# Cas9/guide RNA-based gene-drive dynamics following introduction and introgression into diverse anopheline mosquito genetic backgrounds

**DOI:** 10.1186/s12864-024-10977-w

**Published:** 2024-11-13

**Authors:** Taylor Tushar, Thai Binh Pham, Kiona Parker, Marc Crepeau, Gregory C. Lanzaro, Anthony A. James, Rebeca Carballar-Lejarazú

**Affiliations:** 1 Department of Microbiology & Molecular Genetics, University of California, Irvine, CA 92697-4025 USA; 2 Vector Genetics Laboratory, Department of Pathology, Microbiology and Immunology, School of Veterinary Medicine, University of California, Davis, CA 95616 USA; 3 Department of Molecular Biology & Biochemistry, University of California, Irvine, CA 92697-3900 USA

**Keywords:** Hybrid, Gene conversion, Homology-directed repair, Population replacement, Malaria

## Abstract

**Background:**

Novel technologies are needed to combat anopheline vectors of malaria parasites as the reductions in worldwide disease incidence has stalled in recent years. Gene drive-based approaches utilizing Cas9/guide RNA (gRNA) systems are being developed to suppress anopheline populations or modify them by increasing their refractoriness to the parasites. These systems rely on the successful cleavage of a chromosomal DNA target site followed by homology-directed repair (HDR) in germline cells to bias inheritance of the drive system. An optimal drive system should be highly efficient for HDR-mediated gene conversion with minimal error rates. A gene-drive system, AgNosCd-1, with these attributes has been developed in the *Anopheles gambiae* G3 strain and serves as a framework for further development of population modification strains. To validate AgNosCd-1 as a versatile platform, it must perform well in a variety of genetic backgrounds.

**Results:**

We introduced or introgressed AgNosCd-1 into different genetic backgrounds, three in geographically-diverse *Anopheles gambiae* strains, and one each in an *An. coluzzii* and *An. arabiensis* strain. The overall drive inheritance, determined by presence of a dominant marker gene in the F2 hybrids, far exceeded Mendelian inheritance ratios in all genetic backgrounds that produced viable progeny. Haldane’s rule was confirmed for AgNosCd-1 introgression into the *An. arabiensis* Dongola strain and sterility of the F1 hybrid males prevented production of F2 hybrid offspring. Back-crosses of F1 hybrid females were not performed to keep the experimental design consistent across all the genetic backgrounds and to avoid maternally-generated mutant alleles that might confound the drive dynamics. DNA sequencing of the target site in F1 and F2 mosquitoes with exceptional phenotypes revealed drive system-generated mutations resulting from non-homologous end joining events (NHEJ), which formed at rates similar to AgNosCd-1 in the G3 genetic background and were generated via the same maternal-effect mechanism.

**Conclusions:**

These findings support the conclusion that the AgNosCd-1 drive system is robust and has high drive inheritance and gene conversion efficiency accompanied by low NHEJ mutation rates in diverse *An. gambiae s.l*. laboratory strains.

**Supplementary Information:**

The online version contains supplementary material available at 10.1186/s12864-024-10977-w.

## Background

Gene-drive technologies have the potential to provide novel forms of malaria control by reducing parasite transmission by either suppressing or modifying the anopheline mosquito populations that transmit *Plasmodium spp.* parasites to humans [1]. Proof-of-principle gene-drive lines have been developed in *Anopheles gambiae*, for both population suppression [2–4] and population modification [5, 6] strategies. The existing proof-of-principle gene-drive lines are confined currently to laboratory-based research to evaluate safety and efficacy before semi- or full field trials are considered [7, 8]. In particular, the performance of drive systems in diverse *An. gambiae* populations and whether they can be introgressed into sibling species needs to be evaluated [9, 10]. Evidence that specific gene drive systems will work efficiently in diverse *An. gambiae s.l.* populations will enhance their usefulness [11]. Furthermore, this knowledge is relevant to the development of the target product profiles needed as strains advance through phased testing [7].

Several design features are important for the function of Cas9/gRNA-based gene-drive systems in diverse *An. gambiae s.l.* genetic backgrounds. These include conservation of the chromosomal target site [12] and correct spatial–temporal expression of the Cas9 protein [13]. Moreover, hybrid progeny from gene-drive introductions (moving the systems into different strains of the same species) and introgressions (moving the systems into different species in the complex) must be viable and able to reproduce efficiently to maintain a high level of drive in mixed populations. Haldane’s rule, characterized by the heterogametic sex progeny resulting from hybrid crosses being sterile, applies for some crosses between species within the *An. gambiae* complex and has been observed with hybrids between *An. gambiae* and *An. arabiensis* [14, 15]. However, sterility of the heterogametic sex is not expected following hybridization between *An. gambiae sensu strictu (s.s.)* and *An. coluzzii* because of their relatively recent speciation and still incomplete reproductive isolation, crosses of two genetically-distinct lines of *An. gambiae s.s.* or those with distinct phenotypes such as insecticide resistance [16, 17]. It is unknown whether Cas9/gRNA-based drive systems will affect the reproductive viability of hybrid progeny in ways that deviate from what is expected based on Haldane’s rule for introgressions of wild-type *An. gambiae* sibling species. An introgression of a Cas9/gRNA sex-distortion drive system from *An. gambiae* to *An. arabiensis* has previously been demonstrated [18] and reproductive phenotypes of hybrid progeny did not vary from what has been observed with unmodified mosquitoes [19], providing evidence that there was no unexpected effect on reproductive fitness due to the drive system itself.

Our work investigates the ability of AgNosCd-1, a population modification-based drive system developed in the *An. gambiae* G3 strain [5], to be introduced by reciprocal matings into three diverse *An. gambiae* laboratory strains (KISUMU, NDOKAYO and ZANU) and into two *An. gambiae* sibling species, *An. coluzzii* (MOPTI) and *An. arabiensis* (DONGOLA). Sequencing of the AgNosCd-1 target site in the respective wild-type lines showed a high level of sequence conservation, with only one strain, NDOKAYO, exhibiting a low frequency of alleles containing a single nucleotide polymorphism (SNP) in the target site. The AgNosCd-1 G3 line was used in small cage crosses to introduce and introgress the drive components from both male and female homozygous gene-drive parents. Evidence of inappropriate temporal or spatial (referenced herein as ‘leaky’) Cas9 expression and/or maternal effect was observed in all hybrids but there was no greater effect in any one of the assessed genetic backgrounds compared to the original AgNosCd-1 G3 background. Furthermore, none of the wild-type lines were observed to be more susceptible to these effects when compared to the original AgNosCd-1 G3 line [5, 20]. Analysis of F1 and F2 hybrids allowed us to determine the rate of functional and non-functional mutant allele formation resulting from nonhomologous end-joining (NHEJ) and estimate the rates of drive inheritance amongst the diverse genetic backgrounds. The genetic background for each strain was described using a multi-locus genotyping assay developed to assess hybridization and introgression between *An. coluzzii* and *An. gambiae* in nature [21]. Results then were used to determine whether there was an association between *An. gambiae s.s.* and/or *An. coluzzii* genetic background on NHEJ allele formation or drive inheritance rate in F2 individuals.

## Results

### Molecular analyses of target site-conversion in the diverse wild-type parental lines

**Fig. 1:**
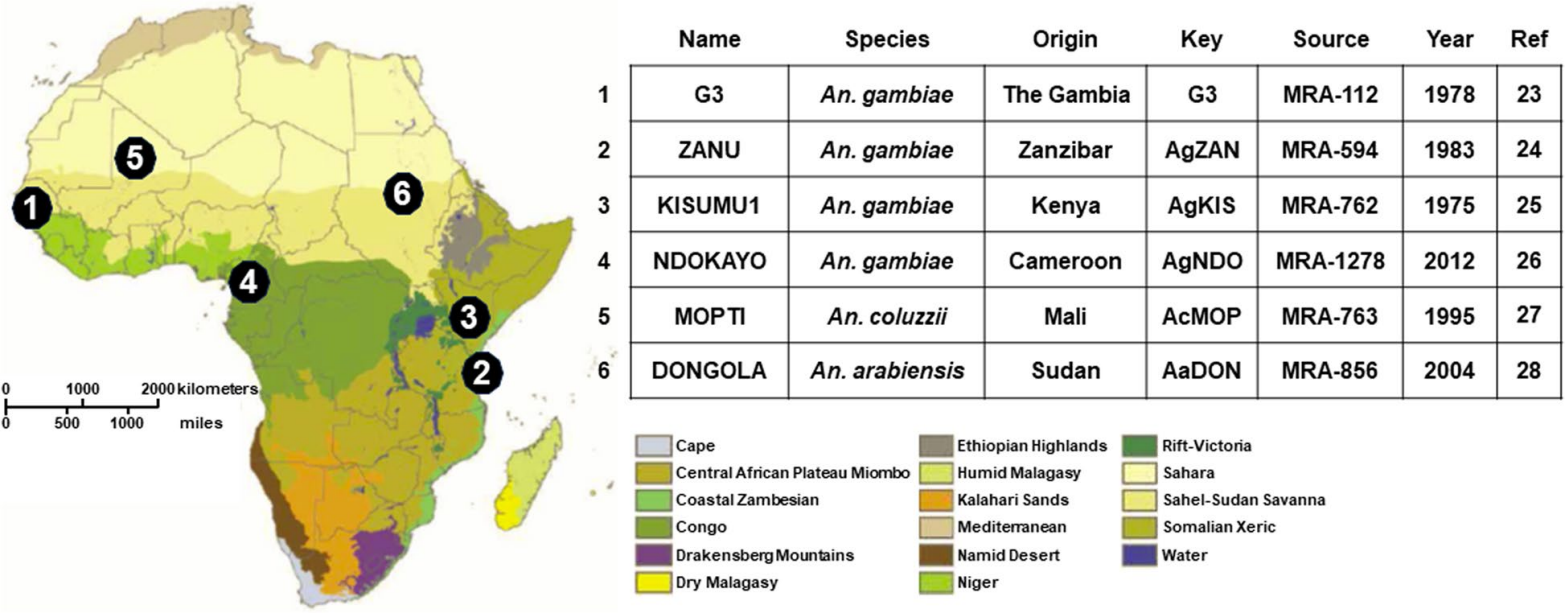
Geographic and ecological diversity of *Anopheles gambaie* and *An. coluzzii *strains. The six parental strains used in this study come from distinct geographic and ecological zones. The numbers refer to the specific strain and are placed over the phytogeographic region from which they were first colonized. The species and place of origin are listed. The key designation refers to the abbreviations used in the text. The source lists the strain number as available from BEI MR4. Year refers to the first available reference. The phytogeographic map was adapted with permission from the 2013 publication ‘A New Map of Standardized Terrestrial Ecosystems of Africa’, copyright American Association of Geographers [23–29]

Homozygous AgNosCd-1 males and females were crossed to respective males and females from *An. gambiae* KISUMU (AgKIS), *An. gambiae* NDOKAYO (AgNDO) and *An. gambiae* ZANU (AgZAN), and *An. coluzzii* MOPTI (AcMOP) and *An. arabiensis* DONGOLA (AaDON) [22] (Fig. 1). DNA from a subset of 50–120 of the parental *An. gambiae*, *An. coluzzii* and *An. arabiensis* wild type adults were sequenced at the AgNosCd-1 gRNA target site in the *cardinal* gene to determine if any sequence variation existed within the diverse genetic backgrounds at the gene drive target site (Figure S1A). The sequences of the target sites were found to contain no polymorphisms in all individuals from all lines except AgNDO (Figure S2A). Some AgNDO individuals had an A → T transversion SNP at the 17th nucleotide in the canonical target site. This specific *cardinal* SNP was documented previously and is known to occur in natural *An. gambiae* populations at estimated frequencies from 0.32 – 0.42 [5]. The overall frequency of this SNP within the AgNDO individuals used here was 0.29 (*N* = 130) and was present in two different haplotypes (Figure S2A-B).

### Small cage experimental crosses

**Fig. 2:**
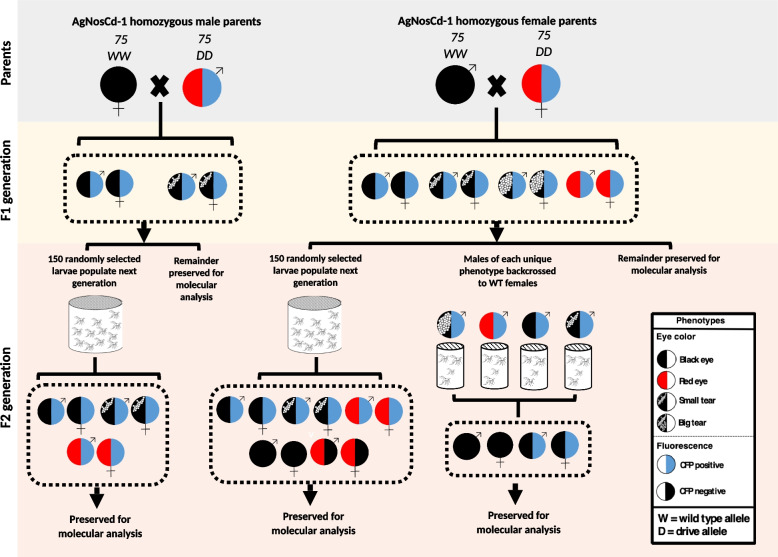
Schematic representations of experimental crosses and analysis. Parental crosses consist of wild-type individuals from each respective introduction or introgression line and homozygous AgNosCd-1 individuals of the opposite sex. All crosses were performed in triplicate and wild-type males and females used in the crosses were preserved for analysis of target site conservation. *F1 generation*: 150 randomly-selected F1 hybrid larvae form the parental crosses were used to populate the next generation (F2) from each male and female lineage. The remaining F1 individuals from male lineages were screened at the pupal stage, reared to adulthood, and preserved for molecular analysis. F1 progeny from female lineages not used in either F1 or F2 generation crosses were screened at the pupal stage, reared to adulthood and preserved for molecular analyses. *F2 generation*: 150 randomly-selected F1 hybrid larvae from the parental crosses were reared, allowed to intercross and used to populate F2 generation. A subset of F1 males representative of each unique phenotype was taken from the female lineage and outcrossed to wild-type females of the respective parental line. All F2 progeny of these F1 phenotype-specific backcrosses were screened as pupae, reared to adulthood and preserved for molecular analysis. The F2 progeny from the F1 next-generation crosses were screened at the pupal stage, reared to adulthood and preserved for molecular analysis

Three replicates of 75 homozygous AgNosCd-1 males or females crossed to 75 wild-type mosquitoes of the opposite sex from each wild-type strain were completed to test the ability of AgNosCd-1 to be introduced or introgressed into diverse *An. gambiae spp.* genetic backgrounds (Fig. 2). All F1 hybrids from each cross were screened as pupae and phenotypes recorded prior to use in subsequent crosses or preservation for molecular analysis. A subset of 150 randomly-selected F1 hybrid progeny of each replicate was allowed to intercross and generate F2 progeny while 15–20 F1 hybrid males from the female lineages were used in phenotype-specific backcrosses. Any remaining F1 hybrid progeny were preserved for molecular analysis. The screening and sequencing of F1 hybrids from male and female gene-drive lineages allowed the determination of the extent of maternal effects and/or leaky Cas9 expression in each unique *An. gambiae spp.* genetic background. The screening and sequencing of F2 hybrids allowed the calculation of the drive conversion rates (efficiency) of the F1 hybrids and an estimate of the prevalence of exceptional phenotypes containing NHEJ alleles that may have the potential to reduce drive efficiency in future generations. A total of 73,397 progeny mosquitoes were scored from the F1 and F2 cage experiments,

### F1 progeny

All (100%) F1 progeny from the experimental crosses were cyan fluorescent protein positive (CFP^+^) consistent with one parent being homozygous for the drive system carrying the dominant marker gene (Table 1). In contrast, differences were seen in the frequencies of eye phenotypes when comparing the progeny from paternal or maternal gene-drive founder lineages. Mosaicism in the eye, in the form of tear- or cardinal-eye coloration, has been observed previously in the progeny resulting from both male and female AgNosCd-1 founder outcrosses in the G3 genetic background [5, 20]. The mosaicism is presumed to be caused by either expression or accumulation of the Cas9 protein outside of the germline or the deposition of Cas9 protein in embryos derived from gene-drive maternal lineages, both of which in the presence of the ubiquitously-expressed gRNAs can cause cleavage and generate NHEJ alleles in somatic and/or germline cells of gene drive progeny [5, 6, 20, 30, 31]. Different *An. gambiae* genetic backgrounds may vary in their susceptibility to leaky Cas9 expression or maternal effects.

**Table 1:** Phenotypes of F1 generation hybrid progeny from AgNosCd-1 experimental crosses to wild-type strains

Parents	Wild-type Strains	Black-eye/ CFP^+^	Small Tear/ CFP^+^	Big Tear/ CFP^+^	Cardinal-eye/ CFP^+^
♂ AgNosCd-1	♀ AgKIS	99.6% (1718/1725)	0.4% (7/1725)	–	–
	♀ AgNDO	99.9% (5777/5780)	0.1% (3/5780)	–	–
	♀ AgZAN	99.3% (2795/2814)	0.7% (19/2814)	–	–
	♀ AcMOP	99.6% (1632/1639)	0.4% (7/1639)	–	–
	♀ AaDON	99.98% (4145/4146)	0.02% (1/4146)	–	–
♀ AgNosCd-1	♂ AgKIS	22.3% (1126/5040)	27.7% (1393/5040)	42.2% (2127/5040)	7.8% (394/5040)
	♂ AgNDO	27.0% (1538/5691)	33.1% (1884/5691)	31.9% (1816/5691)	8.0% (453/5961)
	♂ AgZAN	32.7% (1403/4292)	33.7% (1448/4292)	26.4% (1132/4292)	7.2% (309/4292)
	♂ AcMOP	21.6% (618/2858)	21.7% (619/2858)	45.0% (1288/2858)	11.7% (333/2858)
	♂ AaDON	33.7% (1083/3209)	29.9% (958/3209)	28.5% (916/3209)	7.9% (252/3209)

Table legend. Crosses were performed in three replicates with either 75 homozygous male gene drive parents, ♂ AgNosCd-1, or homozygous female gene drive parents, ♀ AgNosCd-1, and 75 individuals of the opposite sex from each respective wild-type strain. Resulting F1 progeny displayed unique eye phenotypes (black eyes, small or big tear eyes, or cardinal eyes) and all carried the drive system dominant marker, CFP. Numbers in parentheses are the sum of three replicates. –, indicates no individuals with phenotype/genotype were identified. Abbreviation: CFP^+^, cyan fluorescent protein positive

**Fig. 3:**
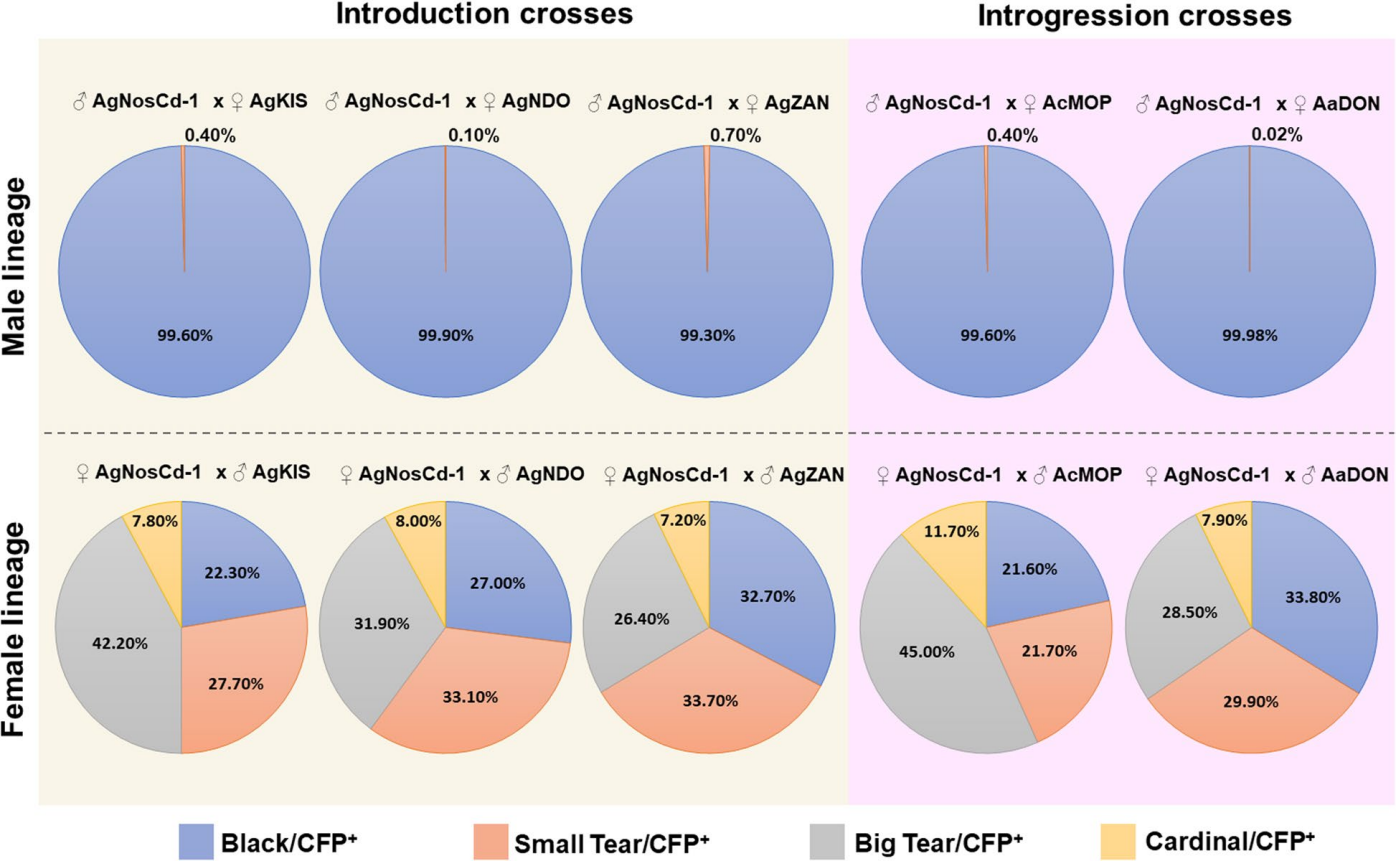
Phenotype proportions observed in F1 hybrids from male and female lineages introduction and introgression outcrosses. AgNosCd-1 male lineages produced a majority of black-eye/CFP^+^ progeny with a small proportion having a small tear/CFP^+^ phenotype. AgNosCd-1 female lineages produced a number of different phenotypes. The greatest proportion of progeny from female crosses had small and big tear phenotypes in all introduction and introgression outcrosses while the smallest proportion of progeny had the cardinal-eye phenotype

Differences in exposure to Cas9 cleavage outside of the germline was monitored by mosaic tear-eye or cardinal-eye phenotypes in the F1 hybrid progeny resulting from AgNosCd-1 male and female lineage outcrosses (Table 1). The average percentages of F1 hybrid progeny with the tear-eye phenotype in the triplicate male AgNosCd-1 introduction crosses was low, 0.4%, 0.1% and 0.7% for AgZAN, AgNDO and AgKIS, respectively (Table 1, Fig. 3). Similarly, AcMOP and AaDON had low respective average percentages, 0.4% and 0.02%, of tear-eye mosquitoes in the replicate male lineage introgression crosses (Table 1 and Fig. 3). Overall, no statistical differences were observed in the rate of tear-eye formation resulting from male AgNosCd-1 outcrosses to diverse *An. gambiae* lines (pairwise t-tests with Bonferroni correction, adjusted *x*-values ≥ 0.87) and the proportion of tear-eye progeny in the introduction and introgression crosses is comparable to that observed in hemizygous AgNosCd-1 male lineage outcrosses [5].

In contrast, the overall proportion of tear-eye individuals in the F1 progeny resulting from female AgNosCd-1 introduction and introgression outcrosses was much greater than that observed for the male outcrosses. These results are consistent with what was observed previously with the AgNosCd-1 (G3) line and provides additional evidence that maternal effects likely contribute more to mosaicism than leaky expression in this specific drive system [5, 20]. The average proportions of F1 hybrid progeny with tear-eye phenotypes (big and small) from the introduction outcrosses were 69.9%, 65.0% and 60.1%, in AgKIS, AgNDO and AgZAN, respectively (Table 1, Fig. 3), and the respective AcMOP and AaDON introgression outcrosses were 66.7% and 58.4% (Table 1, Fig. 3). No statistical differences were observed in the rate of tear-eye formation resulting from AgNosCd-1 female outcrosses to diverse *An. gambiae* lines (pairwise t-tests with Bonferroni correction, adjusted *p*-values ≥ 0.66).

An exceptional cardinal-eye phenotype can also occur in female AgNosCd-1 outcrosses and presumably results from a greater accumulation of NHEJ loss-of-function alleles in the cells that make up the eye in these individuals. Previous work showed that the greater accumulation of NHEJ alleles in these exceptional individuals also can result in the germline transfer to the next generation, which has the potential of causing inhibition of the drive system over time if they accumulate to sufficient frequencies [20]. The exceptional cardinal-eye phenotype was observed in all AgNosCd-1 female introduction and introgression crosses with average percentages in F1 hybrid progeny of 7.8%, 8.0% and 7.2% from AgKIS, AgNDO and AgZAN, respectively, and 11.7% and 7.9% from the respective AcMOP and AaDON introduction outcrosses (Table 1, Fig. 3). No statistical differences were observed in the rate of cardinal-eye formation resulting from AgNosCd-1 female outcrosses to diverse *An. gambiae* lines (pairwise t-tests with Bonferroni correction, adjusted *p*-values ≥ 0.45). The proportions of progeny from female AgNosCd-1 outcrosses to diverse *An. gambiae* lines containing either the tear- or cardinal-eye phenotypes were comparable to the proportions of the tear (pairwise t-tests with Bonferroni correction, adjusted *p*-values ≥ 0.67) and cardinal-eye phenotypes (pairwise t-tests with Bonferroni correction, adjusted *p*-values ≥ 0.085) observed for the original female AgNosCd-1 line outcrosses [20], which supports the conclusion that none of the investigated *An. gambiae* genetic backgrounds were more susceptible or resistant to maternal effects caused by the drive system.

### F2 progeny

The inheritance efficiency of the AgNosCd-1 gene drive system was determined by calculating the proportions of F2 progeny that carried at least one copy of the gene drive (CFP^+^) from an intercross of F1 hybrid parents hemizygous for one drive allele and one allele from the introduced or introgressed wild-type line (Table 2). High drive inheritance was observed in the four F1 hybrid lines that produced progeny, and as expected, the sterility of F1 hybrid males from the AgNosCd-1/AaDON crosses prevented the generation of F2 hybrids. A higher drive inheritance in the F1 hybrid intercrosses from male gene-drive lineages was observed when compared with the female lineages, which is consistent with what was observed in the AgNosCd-1 G3 line [5]. Specifically, the AgKIS, AgNDO, AgZAN and AcMOP F1 hybrid intercrosses from the male gene-drive lineages showed that 100% of the F2 progeny were CFP^+^ indicating that all the progeny inherited at least one copy of the drive system (Table 2). In contrast, a small percentage of the F2 progeny from the female lineages did not inherit a copy of the drive (AgKIS 0.6%, AgNDO 1.2%, AgZAN 0.8% and AcMOP 0.8%). While this resulted in a slightly lower drive inheritance in all genetic backgrounds, strong biased inheritance of the AgNosCd-1 allele was seen with > 98% F2 progeny inheriting at least one copy of the drive allele in all genetic backgrounds, far exceeding expected Mendelian outcomes of 75% drive inheritance in F2 progeny (*χ*^2^ > 1760, *p*-value < 2.2e-16). Overall, the F2 progeny phenotypes from male and female gene-drive lineages provide evidence for the ability of the AgNosCd-1 drive system to initiate cleavage and perform homology-directed repair (HDR) on wild-type alleles from diverse genetic backgrounds.

**Table 2:** Phenotypes of F2 generation hybrid progeny from AgNosCd-1 F1 hybrid intercrosses

F1 hybrids intercross		Black-eye/ CFP^+^	Cardinal-eye/ CFP^+^	Small Tear/ CFP^+^	Black-eye/ CFP^−^	Cardinal-eye/ CFP^−^
Male lineage	AgNosCd-1/ AgKIS	3.7% (189/5036)	96.2% (4843/5036)	0.1% (4/5036)	–	–
	AgNosCd-1/ AgNDO	6.5% (263/4023)	93.4% (3759/4023)	0.1% (1/4023)	–	–
	AgNosCd-1/ AgZAN	1.5% (67/4409)	98.5% (4345/4409)	–	–	–
	AgNosCd-1/ AcMOP	0.3% (11/3795)	99.7% (3784/3795)	–	–	–
Female lineage	AgNosCd-1/ AgKIS	11.8% (540/4567)	87.6% (3999/4567)	–	0.5% (25/4567)	0.1% (3/4567)
	AgNosCd-1/ AgNDO	8.3% (484/5835)	90.5% (5278/5835)	0.03% (2/5835)	1% (57/5835)	0.2% (14/5835)
	AgNosCd-1/ AgZAN	8.3% (295/3570)	90.8% (3243/3570)	0.1% (4/3570)	0.2% (7/3570)	0.6% (21/3570)
	AgNosCd-1/ AcMOP	6.2% (307/4962)	93.0% (4614/4962)	–	0.3% (16/4962)	0.5% (25/4962)

Table legend. Crosses were performed in three replicates with 150 randomly-selected F1 progeny (produced from either a male or female gene-drive parent lineages) allowed to intercross to produce the F2 generation. The resulting F2 progeny displayed unique eye phenotypes (black eyes, small or big tear eyes or cardinal eyes) and presence/absence of the drive system dominant marker, CFP, was determined. Numbers in parentheses are the sum of three replicates. Abbreviations: CFP^+^, cyan fluorescent protein-positive; CFP^−^, cyan fluorescent protein-negative

### Phenotype-specific backcrosses

F1 hybrid males (all CFP^+^) of each unique eye phenotype from female lineages were backcrossed to females of their respective wild-type line to determine whether NHEJ alleles in F1 mosaic hybrids with black-, tear- or cardinal*-*eye phenotypes would affect drive inheritance. A total of 2,416 progeny were scored from all of the crosses. A return to Mendelian inheritance of the drive system was not observed in the backcrosses of the black-eye or tear-eye hybrid phenotypes, both big and small, from any genetic background (Tables S1-S3). Some cohorts of tear-eye phenotypes had lower drive inheritance than others (with a range of 84.9–100% among all genetic backgrounds), however, all the observed drive inheritance rates of tear-eye individuals are comparable to those observed previously for AgNosCd-1 hemizygous individuals from female lineages [5].

The exceptional cardinal-eye phenotypes in AgNDO and AcMOP F1 hybrids showed biased inheritance of the drive allele with 81.1% of AgNDO and 81.2% of AcMOP hybrid progeny inheriting a copy of the drive (Tables S1 and S3). However, the cardinal-eye phenotypes of the AgKIS crosses showed a return to Mendelian inheritance with only 50.8% of progeny inheriting a copy of the drive (Table S2) as shown in previous assessments of the cardinal-eye phenotypes generated by maternal deposition of Cas9/gRNA complexes in the AgNosCd-1 line [20]. These results support the conclusion that the NHEJ alleles generated via maternal effect in AgNDO and AcMOP genetic backgrounds are less likely to be present in germline progenitor cells than in AgKIS or G3 genetic backgrounds. The F1 hybrid phenotype-specific backcrosses from the AgZAN lines did not produce enough progeny to be evaluated and backcrosses of AaDON F1 hybrid males were not viable due to sterility of the F1 hybrid males. These results provide evidence that either the amount of and/or the spatial–temporal action of maternally-deposited Cas9 complexes may differ in different genetic backgrounds.

### Molecular analysis of F1 hybrid progeny from small cage experimental crosses

The target site DNAs of a subset of cardinal-eye, F1 hybrid progeny from all female lineage replicates were sequenced to confirm the genotypes causing loss-of-function mutations at the *cardinal* gene locus (supplemental files 1–5). A minimum of 37 cardinal-eye individuals, representing all three replicates, were sequenced from each introduction or introgression cross. In the majority of crosses, the cardinal-eye individuals (260/263 mosquitoes) were found to contain both a drive allele and at least one loss-of-function NHEJ allele (Table S4A). A number of individual cardinal-eye/CFP^+^ progeny from AgNosCd-1 outcrosses were reported previously to have more than one NHEJ allele along with the drive allele, presumably resulting from different NHEJ alleles that were formed independently during zygote development [20]. Three individuals from one replicate of the AgNosCd-1/AgZAN female lineage crosses carried a drive allele and unidentified NHEJ allele (Figure S3). To determine whether functional NHEJ alleles also were present, a subset of black-eye, F1 hybrid progeny from male and female lineage crosses were sequenced. No evidence of functional NHEJ alleles was discovered in the male lineages from any of the crosses, however, functional NHEJ alleles were recovered in the black-eye progeny from female lineages, consistent with the outcomes of a maternal effect (Table S4B1-B2). The percentage of black-eye progeny with functional NHEJ alleles varied among the genetic backgrounds with AgKIS F1 hybrids having the lowest, (5.0%), followed by AaDON (14.0%), AgZAN (18.6%), AgNDO (22.5%) and AcMOP (31.7%) (Table S4B2).

### Molecular analysis of F2 hybrid progeny from small cage experimental crosses

A majority of the F2 progeny from the F1 hybrid intercrosses had a cardinal-eye phenotype with CFP fluorescence providing evidence that they likely inherited two copies of the drive system, one from each hybrid parent (Table 2). However, a small percentage (0.3–11.8%) of the progeny from each hybrid intercross inherited only one copy of the drive system (black-eye, CFP^+^) and a smaller percentage (0.6–1.2%) of the progeny from the female drive lineages failed to inherit a single copy of the drive system (black- or cardinal-eye, CFP^−^). Sequencing the DNA of a subset of all F2 phenotypes from each replicate (supplemental files 1–5) allowed an estimation of the impact of the various mechanisms by which the drive system could have failed, either through inability of Cas9 to initiate HDR on wild-type alleles or inheritance of NHEJ alleles from their parents.

F1 hybrid intercrosses from male gene-drive lineages provided no evidence of NHEJ alleles affecting drive allele inheritance as no CFP^−^ individuals were recovered (Table 2), and all progeny with a black-eye/CFP^+^ phenotype from male lineages had one drive allele and one wild-type allele (Table S5). Non-functional, potentially drive-resistant alleles are expected to contribute to a cardinal-eye phenotype when homozygous (CFP^−^) or opposite a drive-carrying locus (CFP^+^). F1 hybrid intercrosses from female gene-drive lineages showed evidence of both lack of Cas9-mediated HDR and inheritance of NHEJ alleles causing reduced inheritance of the drive. Progeny with black-eye/CFP^+^ phenotypes from female lineages were a mixed population of individuals hemizygous for the drive allele and a wild-type allele (10–29%) or hemizygous for a drive allele and a functional NHEJ allele (71–90%) (Table S5). The proportions of these wild-type or functional NHEJ alleles in the female lineage F2 progeny varied among the different genetic backgrounds but NHEJ alleles were more predominant (≥ 71%) in all of them.

Analysis of the cardinal-eye/CFP^+^ progeny showed no evidence of non-functional NHEJ alleles in the male lineages indicating that they were all homozygous for the gene-drive system (Table S6). In contrast, there was again a mixed population in the female lineages with individuals homozygous for the drive allele (76.7–93.4%) or hemizygous for a drive allele and non-functional NHEJ allele (6.6–23.3%) (Table S6). The proportion of NHEJ alleles contributing to the cardinal-eye/CFP^+^ phenotype varied among the different genetic backgrounds but the drive allele (≥ 76.7%) was more predominant in all genetic backgrounds than the non-functional NHEJ alleles (≤ 23.3%).

The small percentages of progeny from female lineages that did not inherit any drive allele (black- or cardinal-eye, CFP^−^) showed a mixture of genotypes. Black-eye/CFP^−^ progeny were either hemizygous for NHEJ and wild-type alleles (33–86%) or homozygous for functional NHEJ alleles (14–67%), and only non-functional NHEJ alleles (100%) were found in the cardinal-eye/CFP^−^ phenotypes (Table S7). The NHEJ alleles predominated (≥ 57%) in the individuals without drive alleles, and no individual from any line inherited only wild-type alleles. These data support the conclusion that NHEJ alleles contribute more than non-converted wild-type alleles to the observed reduction in drive inheritance in female lineages and no clear evidence was found of reduced Cas9/gRNA performance on wild-type alleles in any of the genetic backgrounds tested.

To assess whether inheritance of the drive allele in the F2 generation of AgNDO male lineages crosses was impacted by the presence of the target-site SNP, the frequencies of wild-type alleles containing the SNP or canonical wild-type alleles were compared in F2 progeny that inherited one wild-type allele (SNP or canonical) and one copy of the drive system (black-eye/CFP^+^) (Figure S2B). The observed frequencies of these two alleles then were compared to the expected frequencies of the SNP and canonical wild-type alleles based on the frequency of these alleles in their AgNDO female parents. The relative frequency of the SNP allele in the AgNDO parental females that contributed alleles to the AgNosCd-1 male experimental crosses was 0.31 (Figure S2) and the frequency of the SNP allele in black-eye/CFP + F2 progeny was 0.40 (Table S8). Therefore, the SNP alleles and canonical wild-type alleles of the F2 individuals from male lineages that only inherited one copy of the drive system are present with frequencies similar to what was observed in their AgNDO parents. Theoretically, if the SNP alleles impair gRNA recognition and Cas9 cleavage, and therefore HDR-based integration of the drive system, it would be expected that these alleles would be inherited unaltered in subsequent generations and make up a greater frequency of the wild-type alleles in black-eye/CFP^+^ F2 progeny. Based on these observations it is not evident that the SNP present in the AgNDO strain blocks Cas9-mediated DNA cleavage.

### Association of drive dynamics and genetic background

Differences in the rates of NHEJ allele accumulation in the F1 hybrid progeny exist among each of the derived hybrid lines as do differences in overall drive inheritance in the F2 generation. The highest frequencies of NHEJ alleles were found in the AcMOP F1 hybrids (9.3%) followed by AgZAN (7.5%), and AgNDO (7.2%), while the AgKIS F1 hybrids had a little more than half as many (4.3%) NHEJ alleles as the average of the other lines (Table 3). The lower frequency of NHEJ alleles in the F1 AgKIS hybrids likely contributed to this line having the highest frequency of drive alleles in the F2 generation (91.6%). Interestingly, despite the AcMOP line having the highest frequency of NHEJ alleles in the F1 generation, it had the second-highest inheritance of drive alleles in the F2 generation (88.4%). One possible explanation for this higher level of drive inheritance despite the presence of NHEJ alleles is that the NHEJ alleles in the F1 generation were less likely to be present in the germline. This explanation is supported also by the phenotype-specific backcross data that show a higher-than-expected drive inheritance of the cardinal-eye, CFP^+^ phenotype, despite the presence of NHEJ alleles in these individuals (Table S3 and S4A). Overall, the two lines with predominantly *An. coluzzii* genetic backgrounds had higher drive inheritance in the F2 generation compared to the AgNDO and AgZAN lines where the *An. gambiae* genetic background predominates. In both the AgNDO and AgZAN crosses, the frequency of NHEJ alleles in the F1 generation (7.2% and 7.5% respectively), frequency of NHEJ alleles in the F2 generation (14.6% and 14.6% respectively) and drive alleles in the F2 generation (85.2% and 85.4% respectively) were similar, which supports the conclusion that the drive constructs in these two genetic backgrounds perform similarly (Table 3).

**Table 3:** Summary of NHEJ allele frequencies and the genetic composition in F1 and F2 individuals from female drive lineages

Estimates		Strains			
		AgNDO	AgZAN	AgKIS	AcMOP
F1	Blk/CFP^+^ (percentage of individuals with functional NHEJ alleles)	22.5%	18.6%	5.0%	31.7%
	Cd/ CFP^+^ (percentage of individuals with non-functional NHEJ alleles)	100.0%	94.0%*	100.0%	100.0%
	Total NHEJ alleles^1^ (95% CI)	7.2% (0.0001–34.9%)	7.5% (1.1–13.9%)	4.3% (0.0001–8.8%)	9.3% (5.2–13.3%)
	Blk/ CFP^+^ drive inheritance	93.6%	–	82.0%	98.0%
	Cd/ CFP^+^ drive inheritance	81.1%	–	50.8%	81.2%
F2	Total NHEJ alleles^2^ (95% CI)	14.6% (3.4–25.8%)	14.6% (8.2–20.9%)	8.1% (4.7–11.6%)	11.6% (0.0001–30.6%)
	drive alleles^3^ (95% CI)	85.2% (72.9–97.4%)	85.4% (79.0–91.8%)	91.6% (88.7–94.4%)	88.4% (70.5–99.999%)
% genetic background^4^ (*An. coluzzii*/*An. gambiae*)		25.6%/74.4%	33.3%/66.7%	66.7%/33.3%	92.2%/7.8%

Table legend. Non-homologous end joining (NHEJ) allele frequencies expressed as percentages in F1 generations represent alleles that were generated as a result of maternal effect; frequencies were derived from small cage experimental crosses. Blk/CFP^+^ and Cd/CFP^+^ drive inheritance values represent frequency of F2 offspring from each respective phenotype that inherited a copy of the drive; frequencies were derived from phenotype-specific backcrosses. NHEJ allele frequencies in F2 generations represent alleles that were either inherited or generated de novo from F1 hybrid parent intercrosses; frequencies were derived from small cage experimental crosses.

^1^Total proportion calculation represents the (% of Blk/CFP^+^ phenotype in F1 population x % of NHEJ alleles in Blk/CFP^+^ sequenced individuals) + (% of Cd/CFP^+^ phenotype in F1 population x % of NHEJ alleles in Cd/CFP^+^ individuals) mean and confidence intervals derived from 3 replicates.

^2^Total proportion calculation represents the sum of all unique calculations from each phenotype (% of each phenotype in F2 population x % of NHEJ alleles in sequenced individuals of that phenotype) mean and confidence intervals derived from three replicates.

^3^Total proportion calculation represents the sum of all unique calculations from each phenotype (% of each phenotype in F2 population x % of drive alleles in sequenced individuals of that phenotype) mean and confidence intervals derived from three replicates.

^4^Proportion of genotyping markers with a SNP associated with either *An. coluzzii* or *An. gambiae*. *3/50 sequences not identified, likely to be NHEJ alleles but not confirmed. Abbreviations: Blk: wild-type black-eye; CFP^+^: cyan fluorescent protein-positive; Cd: cardinal-eye red eye

### Genetic backgrounds

**Fig. 4:**
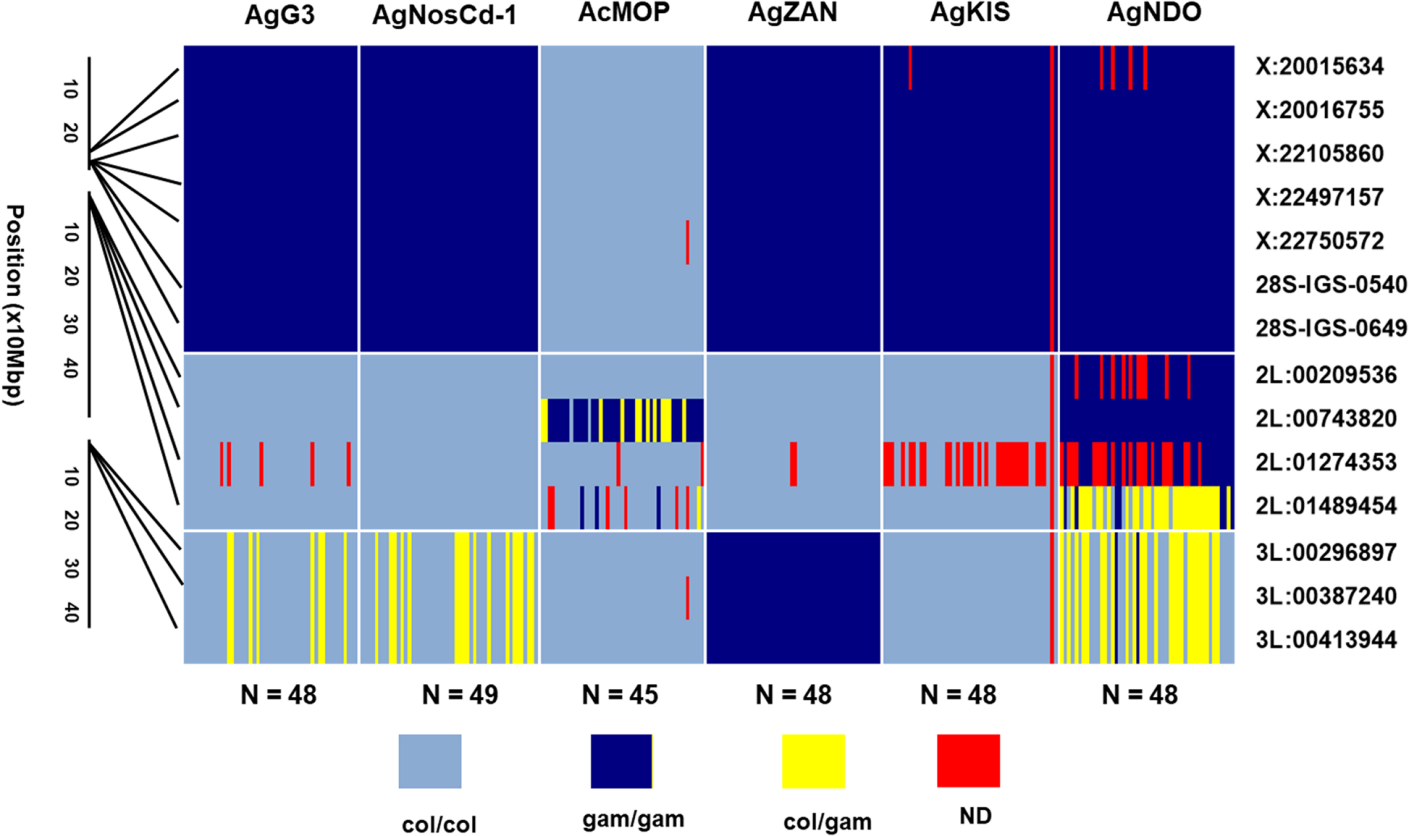
Genotypes at the 14 SNP markers used as part of the DIS assay. Data for the six strains are arranged in blocks subdivided by linkage groups: X = X chromosome, (28S is also on the X), 2L = left arm of chromosome 2, and 3L = left arm of chromosome 3. SNP labels are listed on the right and their locations in the genome are depicted on the left. The light blue color (col/col) represents the *An. coluzzii* genotype, the dark blue (gam/gam) is the *An. gambiae* genotype, yellow (col/gam) are the *An. coluzzii*/*An. gambiae* hybrid heterozygotes, and red (ND) represents missing data. Each column represents individual mosquitoes with genotypes organized in 14 rows. Sample sizes (N) for each strain are presented at the bottom of the Figure. G3 is a long-standing colony that is an *An. coluzzii*/*An. gambiae* hybrid, this strain was engineered to produce the AgNosCd-1 strain. AgNosCd-1 is the parental strain used to generate introduced and introgressed strains by crossing and inbreeding. The *An. gambiae* strains include AgZAN (Zanzibar-Tanzania), AgKIS (Kisumu-Kenya) and the AgNDO (Ndokayo-Cameroon). The AcMOP (N’Gabacoro Droit-Mali) is the *An. coluzzii* strain

The three *An. gambiae* (AgZAN, AgKIS and AgNDO) and one *An. coluzzii* (AcMOP) wild-type strains also were maintained as individual colonies while the crosses with AgNosCd-1 were being performed. The genetic backgrounds of these strains were defined using the “Divergence Island SNP” (DIS) genotyping assay (Fig. 4). This assay utilizes 14 species-specific SNP genotypes distributed across three linkage groups (X, 2L and 3L chromosomes) and was developed to evaluate introgression between *An. coluzzii* and *An. gambiae* [21, 32]. AgNosCd-1 was established initially in G3, which has been reported as a mixed strain resulting from hybridization of *An. gambiae* and *An. coluzzii* (https://www.beiresources.org/Catalog/BEIVectors/MRA-112.aspx). This is reflected in a DIS profile that indicates that G3 has an *An. gambiae* X chromosome profile and 2nd chromosome markers that originate from *An. coluzzii.* Chromosome 3 is polymorphic carrying both *An. coluzzii* and *An. gambiae* markers. Interestingly the *An. gambiae* 3rd chromosome-specific markers are only present as heterozygotes. It is unclear whether this is simply a sampling artifact or indicates the presence of a balanced lethal system in this strain. As expected, the AgNosCd-1 strain has the same DIS profile as G3. The colonization of the AgZAN, AgKIS, AgNDO and AcMOP strains was completed prior to the identification of M and S forms as two unique species and it was assumed in the available documentation that the first three are true-breeding *An. gambiae s.s.* and the last a true-breeding *An. coluzzii* line based on a multiplex-PCR assay used to verify the lines background [33]. Therefore, it was expected a priori that each strain would have homozygous species-specific DIS profiles for all 14 of the previously assigned SNPs [21]. However, the AgKIS DIS profile is most similar to that of the AgNosCd-1 strain with the X chromosome of *An. gambiae* origin and both autosomes fixed for *An. coluzzii* SNPs. Furthermore, whereas AgNosCd-1 is polymorphic for the 3rd chromosome SNPs, AgKIS has only the *An. coluzzii*-specific SNPs. The AgZAN strain has *An. coluzzii* 2nd chromosome markers and *An. gambiae* markers for both the X and 3rd chromosome. AgNDO is the most polymorphic of the strains with the X chromosome fixed for the *An. gambiae* SNPs and polymorphic for 2nd and 3rd chromosomes. Genotype frequencies at the three 3L chromosome polymorphisms are in compliance to the Hardy–Weinberg equilibrium (H-W) (*p* < 0.05), whereas the polymorphic 2L chromosome SNPs have a slight, but statistically significant excess of heterozygotes (supplemental file 6). The AcMOP strain is fixed for the *An. coluzzii* X and 3rd chromosome SNPs but is polymorphic for one of the four 2nd chromosome SNPs (2L:00743820 and 2L:01489454). Genotype frequencies for the 00743820 SNP were consistent with H-W expectations whereas there was a significant deficiency of heterozygotes for the 01489454 SNP (supplemental file 6).

## Discussion

Cas9/gRNA-based gene drives rely on HDR-mediated gene conversion at a specific chromosomal target site identified by a guide RNA (gRNA) where the Cas9 endonuclease must reliably and efficiently cleave the DNA. The target sites selected for existing proof-of-principle drive systems were chosen in part for their highly conserved DNA sequences throughout diverse populations of the *An. gambiae* complex. However, small variations in the form of SNPs exist at low frequencies within these intended target sites [5]. For successful introduction or introgression of the Cas9/gRNA-based gene-drive systems to occur, variations in the target site must not inhibit the ability of the gRNAs to recognize the target locus and direct Cas9-mediated DNA cleavage. Sequencing of the wild-type lines used in the crosses has revealed a high level of conservation of the target site at the *cardinal* locus among the tested anopheline lines. This high-level conservation was expected based on previous analysis of the *cardinal* locus [5], and the sequencing data acquired in this work further supports that analysis. The presence of a target site SNP in the AgNDO line allowed testing of that unique polymorphism for its ability to potentially reduce drive inheritance during introduction of the AgNosCd-1 gene-drive construct. In addition, the frequency of the SNP in the long-colonized AgNDO test line was found to be comparable to what was reported for natural populations, therefore, any changes in drive inheritance might support expectations of how the drive could behave in natural populations. No differences were observed in the initial AgNDO introduction crosses although there were minor differences between the AgNDO hybrids and the progeny of the other introductory crosses at the F2 generation. The percentage of F2 progeny with evidence of two copies of the drive allele (cardinal-eye/CFP^+^) in the F2 progeny of the AgNDO /AgNosCd-1 male test cross lineage was lower (93.4%) than the other male introductory test crosses (96.2 -98.5%). However, this difference was not observed in the F2 generation of AgNDO /AgNosCd-1 female lineage crosses (90.5% cardinal-eye/CFP^+^) when compared to the other introductory test crosses (87.6–93.0% cardinal-eye/CFP^+^). It also was observed that the frequency of the SNP in AgNDO/AgNosCd-1 male lineages crosses did not change much over two generations (0.31 in parental generation, 0.40 in F2 generation). Whether the slight differences in drive inheritance of the AgNDO AgNosCd-1 male introductory crosses are due to the presence of the SNP in small frequencies or some other genetic variation cannot be determined in the scope of these experiments. Further examination of the effect that the SNP has on AgNosCd-1 drive inheritance is needed and efforts are underway to assess drive conversion in iso-lines from the AgNDO colony homozygous for each SNP haplotype and the conserved wild-type allele.

Other potential genetic barriers to successful gene-drive system performance relate to correct temporal and spatial expression of the Cas9 protein. Cas9 expression that is both highly-specific and highly-active across diverse *An. gambiae spp.* genetic backgrounds is desired in a gene-drive system suitable for introduction and introgression. Optimally-designed Cas9 expression should restrict it to the diploid, premeiotic germline tissues and have it expressed in sufficient abundance to ensure high germline gene conversion rates. NHEJ alleles are more likely to arise when Cas9 expression is non-specific and outside the confines of germline cells, and those in the germline can be resistant to further Cas9/gRNA-mediated cleavage causing reduced drive inheritance in subsequent generations [20]. Two different mechanisms have been proposed to lead to the production of NHEJ alleles [30]. The most impactful contributor is the maternal effect resulting from the accumulation of Cas9/gRNA complexes in embryos originating from drive-carrying females, which causes early cleavage of the male-contributed allele and can create NHEJ alleles in both somatic and germline cells that may be more likely to be inherited through the resulting zygote [20, 30]. Temporal and spatial misexpression (‘leakiness’) of a drive-system promoter can result in target site cleavage in the developing zygote that can result in NHEJ alleles, potentially resulting in germline and/or somatic mosaicism [20, 30].

While active and unrestrained Cas9 expression reduces drive inheritance over subsequent generations due to the generation of NHEJ alleles, low Cas9 expression also is not desirable as this has the potential to reduce the rates of HDR-mediated gene conversion in the germline. A very low proportion of wild-type alleles were recovered in the F2 generation amongst all genetic backgrounds (Tables S5-S7) providing evidence that this drive system maintains high levels of Cas9 expression in different genetic backgrounds with high rates of HDR-mediated gene conversion. Ectopic Cas9 activity resulting in maternal effects did have a noticeable impact in the female lineages of all genetic backgrounds but the drive alleles were still inherited at a much higher rate than NHEJ alleles in the F2 generation (Tables S5-S7) similar to what has been observed in the original AgNosCd-1 (G3) strain [5]. F1 progeny showed minor effects of leaky Cas9 expression or paternally-inherited Cas9/gRNA complexes causing mosaicism in male lineages, as has been observed in the AgNosCd-1 parental line [5]. No hybrid line had a greater proportion than any other of mosaic or exceptional cardinal-eye phenotypes in the F1 progeny of female lineages that would indicate a greater susceptibility to maternal effects.

One notable phenotype/genotype combination discovered in the assessment of the maternal-effect phenotypes was the recovery of the three cardinal-eye/CFP^+^ F1 AgZAN hybrids with no evidence of an NHEJ allele. The cardinal-eye individuals resulting from maternal-effect, loss-of-function NHEJ alleles likely result from cleavage of the male-donated wild-type allele in early post-fertilization embryos originating from females carrying the gene-drive system. The physical distance between the male and female chromosomes in the developing embryo is sufficiently large to favor NHEJ over HDR [20, 30]. While we have no previous evidence that this type of early cleavage of the male-contributed wild-type allele results in HDR, these data support the possibility that this may have taken place in a small percentage of the AgZAN F1 progeny. Other possible explanations for the apparent lack of an NHEJ genotype is a failure of the oligonucleotide primer sets to capture and amplify the region. This may have occurred if the target site contained a large deletion or insertion resulting from internal recombination that might disrupt the locus primary structure, and this has been observed in other mosquito gene-drive systems [34].

Further analysis of the phenotypes generated as a result of maternal effects revealed that drive inheritance did vary amongst unique phenotypes and genetic backgrounds. One expectation of the consequences of the exceptional cardinal-eye phenotype based on previous work with AgNosCd-1 was that drive resistant alleles are usually present in the germline of these individuals and drive ability is lost, with inheritance of the drive allele in their progeny returning to expected 1:1 Mendelian ratios [20]. However, in two of the genetic backgrounds, AcMOP and AgNDO, a small mating experiment of exceptional cardinal-eye phenotypes revealed that some drive function remained in these individuals with ~ 80% of their progeny inheriting a copy of the drive. For these genetic backgrounds, maternal-effect related NHEJ events may occur later in embryonic development, after formation of the germline cells. This would reduce the fraction of germline cells carrying alleles resistant to the drive system and thus have a lesser impact on subsequent drive inheritance.

Results derived from work with gene-drive systems in a diverse array of *Drosophila melanogaster* populations found rates of resistance allele formation varying as much as ten-fold in both individuals and populations of different genetic backgrounds [35]. Our data are consistent with these findings and show that diverse *Anopheles gambiae s.l.* genetic backgrounds also exhibit variation in potential resistance allele formation and accumulation. However, given the inability to distinguish NHEJ alleles by unique sex and phenotype combinations with an autosomal drive system, differences in mutant allele formation rates are less straightforward to determine in these mosquitoes than what was possible with the X-linked drive system used in *D. melanogaster* experiments [35]. Other research has shown that a high level of drive inheritance was achieved in two unique *An. gambiae* drive systems (G3 background) upon introduction into three geographically diverse *An. gambiae* strains [36]. Despite heterology around the target sites of the two tested gene-drive lines, no significant differences in drive inheritance were observed in the hybrids of all three strains when compared to each other or the control drive line. Our data complement these findings in that all our introduction crosses showed super-Mendelian inheritance of the AgNosCd-1 drive system, as observed in the original drive line, with little difference in inheritance rates between the hybrid strains.

The utility of AgNosCd-1 and derived gene-drive systems will depend on their ability to move large ‘cargos’ including beneficial genes into target populations at high frequencies. An AgNosCd-1-based system, TP13, was shown to spread a cargo of ~ 15.6 kilobases in length that included the drive system components, dominant marker gene and two anti-parasite effector genes into caged mosquito populations at rates similar to the parental construct [6]. Modeling of this and other parameters showed that this system could have a significant impact of malaria parasite transmission.

The potential of a deleterious impact of gene-drive systems on non-target organisms through horizontal gene transfer (HGT) is a major concern expressed by scientists and communities of stakeholders and potential end-users [37]. The most likely HGT mechanism would be interspecific mating that produced fertile, sexually-reproductive F1 hybrids. It is important to emphasize that the function of the AgNosCd-1 gene-drive system described here is highly likely to be restricted by evolutionary and design considerations to a few closely-related species in the *An. gambiae s.l.* complex. The *An. gambiae* strains AgZAN, AgKIS and AgNDO, in which the gene-drive system is functional, are most closely related to the *An. coluzzii* AcMOP strain, with whom they are estimated to have last shared a common ancestor ~ 0.54 million years ago (mya) [38]. The *An. arabiensis* AaDON strain may be as distant as 1.85 mya from both *An. gambiae* and *An. coluzzii* strains and is already subject to Haldane’s rule producing infertile male F1 hybrids when crossed to the AgNosCd-1 G3 strain, although there is evidence of historic introgression of autosomal chromosomal segments most likely resulting from backcrossing of sufficient numbers of fertile F1 hybrid females [38, 39]. *Anopheles merus*, another member of the complex, also is estimated to have diverged from *An. gambiae*/*An. coluzzii* 1.85 mya, but it shows strong post-mating barriers with other members of the complex [38–41]. We were unable to maintain this species in colony so were not able to test this. Thus, it is possible for the gene-drive system to introgress into species within the *An. gambiae* complex where porous pre- and post-mating barriers have allowed chromosomal segment introgression.

A threshold level for the amount of divergence in insects required for post-mating barriers to emerge is likely to be variable among the tremendous diversity of species across the phylum. Post-mating barriers sufficient to confer hybrid sterility are evident in the genus *Drosophila* following divergences of ~ 0.25 my and it was posited that species in the genus *Anopheles* show more rapid genome evolution and speciation [40, 42]. Two species, *An. christyi* and *An. funestus*, with geographic ranges overlapping those of the *An. gambiae* complex, are not likely targets of AgNosCd-1 introgression [43, 44]. Their respective estimated divergence times of 9 and 20 mya are likely to preclude the function of the AgNosCd-1 control DNA, specifically the *nanos*-driven Cas9 expression, although this should be tested empirically in heterologous transgenesis experiments [38, 39, 45].

## Conclusion

The performance of the core gene drive system AgNosCd-1 was assessed in diverse mosquito genetic backgrounds by analyzing the drive inheritance and resistance allele formation dynamics. AgNosCd-1 showed a high drive inheritance in both males and females and a small number of resistance alleles regardless of the genetic background. These characteristics make AgNosCd-1 a good core drive system to develop further modification/replacement strategies due to its wide potential use in *An. gambiae s.l*.

## Methods

### Mosquito lines and insectary maintenance

The AgNosCd-1 gene-drive system in the *An. gambiae G3* strain genetic background was used for all introduction and introgression experiments [5]. The AgNosCd-1 components include regulatory elements of the *An. gambiae nanos* gene ortholog (*Agnos*) to limit Cas9 transcription to the germline, a U6 promoter-driven gRNA targeting the *cardinal* (*cd*) gene ortholog, and a 3xP3 promoter driving the cyan fluorescent protein (CFP) as a dominant marker (Figure S1A). Three different *An. gambiae* wild-type test strains with distinct geographic origins and colonization histories were used for introduction experiments, ZANU/Zanzibar (AgZAN, MRA-594), KISUMU1/Kenya (AgKIS, MRA-762), and NDOKAYO/Cameroon (AgNDO, MRA-1278) (Fig. 1). Two additional wild-type strains, *An. coluzzii* MOPTI/Mali (AcMOP, MRA-763) and *An. arabiensis* DONGOLA/Sudan (AaDON, MRA-856), also were used for introgression experiments. All wild-type lines were obtained from BEI resources [22] as eggs, reared to adults and allowed to complete two gonotrophic cycles. Adults from the first egg batch were used to maintain the wild-type colony and adults from the second egg batch were separated by sex as pupae and used in experimental crosses.

Adult transgenic and wild-type mosquitoes were maintained at 27°C with 75–80% humidity and a 12-h day/night, 30 min dusk/dawn lighting cycle. Adult females were provided rabbit blood (Colorado Serum Company, CO) through artificial membranes (Hemotek, Inc., Blackburn, UK). Transgenic and wild-type larvae were maintained at 28°C with a 12-h day/night lighting cycle. Larvae were reared in plastic containers (‘pans’, 11″ [7.94 cm]) × 6″ [15.24 cm] × 4″ [10.16 cm]) containing 1 L of distilled water. Approximately 200 first-instar (L1) larvae were placed into each container and provided daily with food (2:1 ground Tetramin fish flakes: Saaf baker’s yeast). Water was changed in the larval rearing containers if it became noticeably foul.

### Small cage introduction and introgression experimental crosses

Three independent small cage replicates containing either 75 homozygous AgNosCd-1 males or females and 75 wild types of the opposite sex of the wild-type test lines were created for each introduction and introgression experimental cross. Males and females from each line were separated by sex as pupae, placed into individual 16-oz (~ 473 cm^3^) containers in groups of 10–12 pupae and kept separately as virgins. When adult males and females were 3–5 days old, males or females from the respective AgNosCd-1 line and wild-type line were combined in a 46-oz cage (1360 cm^3^) and allowed to mate for three days before being offered two consecutive blood meals. Two days after the second blood meal, cages were provided an 8-oz oviposition cup (~ 237 cm^3^) lined with filter paper and filled to 2/3 depth with distilled water. Mosquitoes were allowed to oviposit for three days after which the egg cups were removed and F1 hybrids hatched. After removal of the egg cup, the wild-type males or females used in the experimental crosses were removed from the cage with a mechanical aspirator and frozen at -20°C for molecular analysis. One randomly-selected container of third-instar (L3) F1 larvae was chosen to populate the next generation cage while the other pans were reared to adults and either used in subsequent phenotype-specific experimental crosses or preserved at -20°C for molecular analysis. The next generation cages consisted of ~ 150 F1 hybrid individuals, and the sex and eye phenotype of each individual was determined at the pupal stage prior to being placed in a cage. The F1 hybrid cages were maintained and fed blood in the same manner as described for the initial parental crosses to produce F2 progeny. F2 larvae were hatched as described for the F1 larvae, pupae were screened and sorted based on eye phenotype and sex, kept separate in 16 oz (~ 473 cm^3^) containers and then preserved at -20°C for molecular analysis.

### Screening

All mosquitoes were screened at the pupal stage and kept separately based on eye color, presence/absence of CFP and sex. Both eyes of each individual pupa were examined under a light microscope (Leica MZ6) and sorted into one of the following categories: wild-type pupal eyes with full pigmentation were designated “black”, a pupa lacking a large fragment of pigmentation (> 25%) in at least one eye was designated “big tear”, a pupa with any small fragment of missing pigmentation was designated “small tear”, and a pupa with red pigmentation was designated “cardinal-eye” (Figure S1B). The tear- and cardinal-eye phenotypes have been described previously [5, 20] and are known to represent varying levels of mosaicism resulting from combinations of mutant and wild-type *cardinal* target gene alleles. After screening and sorting for eye color, pupae were examined under a fluorescent microscope (Leica M165FC) for CFP fluorescence and further sorted based on the presence (CFP^+^) or absence (CFP^−^) of fluorescence in the eyes.

### Phenotype-specific backcrosses

A subset of 15–20 male F1 progeny collected from the female lineage of each experimental cross representing each unique eye phenotype was collected and backcrossed to 15–20 females of the corresponding wild-type strain. The phenotype-specific backcrosses were mated, blood-fed and provided an oviposition cup as described for the small cage crosses. The larvae of each phenotype-specific backcross were reared and screened and sorted as pupae based on eye color and CFP fluorescence and individuals of each unique phenotype were collected at the adult stage and preserved at -20°C for molecular analysis.

### DNA Sequencing

Genomic DNA was extracted from individual mosquitoes using the Extract-N-Amp PCR kit (Sigma) following manufacturer-recommended protocols. Three pairs of oligonucleotide primers (Ak5/Ak21, CO159/CO160 and TP179/TP302) (Table S9) were designed to amplify DNA fragments of unique lengths encompassing the gRNA-directed Cas9 cleavage site for identification of potential SNPs in wild-type parental strains and potential mutant alleles in F1 and F2 hybrids. Two additional pairs of oligonucleotide primers (TP180/TP217 for the left junction and TP179/CO94 for the right junction; Table S9) were used to amplify DNA fragments between the *cardinal* gene homology arms and the drive cassette to verify the presence of the gene-drive system. PCR was performed utilizing DreamTaq Polymerase (Thermo Fisher Scientific) following the manufacture-recommended protocol with the annealing temperatures and extension times listed in Table S9. Amplicons were sequenced via Sanger sequencing (Genewiz, USA) and the results were analyzed for potential indels at the gRNA target site (Indigo, https://www.gear-genomics.com/indigo/).

### Genotyping

Six *An. gambiae* mosquito laboratory strain populations were sampled for analysis. These included the two parental strains, G3 (*N* = 48) and AgNosCd-1 (*N* = 49), and AgZAN (*N* = 48), AgKIS (*N* = 48) and AgNDO (*N* = 48). Forty-five (45) samples from the *An. coluzzii* AcMOP strain also were analyzed. DNA was extracted from whole mosquitoes and stored in 80% EtOH using a QIAGEN Biosprint 96 system with QIAGEN blood tissue reagents following established protocols [46–48]. A modified version of the “Divergence Island SNP” (DIS) assay was performed on each sample following established protocols [21, 41]. The DIS assay is a genotyping method for detecting hybridization and introgression between *An. coluzzii* and *An. gambiae* and is based on a multi-locus panel of SNPs determined to be fixed between the two species [32].

## Supplementary Information

Supplementary Material 1.

Supplementary Material 2.

## Data Availability

The datasets generated and/or analyzed during the current study are available in the BankIt repository (BankIt2749631, OR693501-OR695055). Plasmids and mosquito lines are available from the authors on request.
