# Supplementary material for: Cas9/guide RNA-based gene-drive dynamics following introduction and introgression into diverse anopheline mosquito genetic backgrounds
Source: BMC Genomics. 2024 Nov 13;25:1078. doi: 10.1186/s12864-024-10977-w (PMC11558816; doi:10.1186/s12864-024-10977-w)
Supplement: Supplementary file 1 — Supplementary Material 1. [file 12864_2024_10977_MOESM1_ESM.docx]

**Supplemental Files**

**Table S1.** AgNDO F1 hybrid phenotype-specific backcrosses.

**Table S2.** AgKIS F1 hybrid phenotype-specific backcrosses.

**Table S3**. AcMOP F1 hybrid phenotype-specific backcrosses.

**Table S4.** Phenotypes and genotypes in F1 hybrids from experimental crosses from AgNosCd-1 male and female lineages.

**Table S5.** Black-eye, CFP^+^ phenotypes and genotypes in F2 hybrid offspring of introduction and introgression experimental crosses.

**Table S6.** Cardinal-eye, CFP^+^ phenotypes and genotypes in F2 hybrid offspring of introduction and introgression experimental crosses.

**Table S7.** Black and cardinal eye, CFP^-^ genotypes in F2 hybrids from female AgNosCd-1 lineage experimental crosses.

**Table S8.** Sequencing results of black-eye/CFP^+^ F2 offspring from ♂AgNosCd-1 x ♀AgNDO introductory experimental crosses.

**Table S9.** List of oligonucleotide primers.

**Figure S1.** AgNosCd-1 gene drive system and eye phenotypes

**Figure S2.** *cardinal* target-site variation in AgNDO wild-type males and females used in introductory crosses to AgNosCd-1.

**Figure S3.** Unidentified *cardinal* allele in *cd*^-^/CFP^+^ F2 individuals from ♀AgNosCd-1 x ♂AgZAN F1 hybrid intercrosses.

**Sequencing data set.**

|  | Parental phenotypes^*^ | | | |
| --- | --- | --- | --- | --- |
| Phenotypes  Percentage (%)  (total population) | ♂ Blk/CFP^+^  x  ♀ AgNDO | ♂ Small Tear/CFP^+^  x  ♀ AgNDO | ♂ Big Tear/CFP^+^  x  ♀ AgNDO | ♂ Cd/CFP^+^  x  ♀ AgNDO |
| Blk eye/CFP^+^ | 93.6%  (219/234) | 84.9%  (158/186) | 95.7%  (110/115) | 81.1%  (120/148) |
| Small tear/CFP^+^ | --- | --- | --- | --- |
| Big tear/CFP^+^ | --- | --- | --- | --- |
| Blk eye/CFP^-^ | 6.4%  (15/234) | 15.1%  (28/186) | 4.3%  (5/115) | 18.9%  (28/148) |
| *20 males from each F1 hybrid specific phenotype were crossed to 20 WT *An. gambiae* Ndokayo (AgNDO)females. Blk, black eye; Cd, cardinal eye. | | | | |

**Table S1. AgNDO F1 hybrid phenotype-specific backcrosses.** Crosses were performed between twenty F1 AgNDO hybrid males of different phenotypes generated from the female lineage, and twenty wild-type *An. gambiae* AgNDO females. The male hybrid phenotypes outcrossed to wild-type females included: small tear/CFP^+^, black-eye/CFP^+^, big tear/CFP^+^, and cardinal-eye/CFP^+^. The resulting F2 generation consisted of either black-eye/CFP^+^ or black-eye/CFP^-^ progeny and offspring with black-eye/CFP^+^ phenotypes predominated across all phenotype’s specific crosses. ^a^Blk: wild-type black-eye, ^b^CFP^+^: cyan fluorescent protein-positive, ^c^Cd: cardinal-eye red eye, ^d^CFP^-^: cyan fluorescent protein-negative. Frequencies are expressed as percentages with the numbers in parentheses the sums of the replicates.

|  | Parental phenotypes* | | | |
| --- | --- | --- | --- | --- |
| Phenotypes  Percentage (%)  (total population) | ♂ Blk/CFP^+^  x  ♀ AgKIS | ♂ Small Tear/CFP^+^  x  ♀ AgKIS | ♂ Big Tear/CFP^+^  x  ♀ AgKIS | ♂ Cd/CFP^+^  x  ♀ AgKIS |
| Blk eye/CFP^+^ | 82.0%  (196/239) | 98.1%  (266/271) | 100%  (280/280) | 50.8%  (215/423) |
| Small tear/CFP^+^ | --- | 1.5%  (4/271) | --- | --- |
| Big tear/CFP^+^ | --- | --- | --- | --- |
| Blk eye/CFP^-^ | 18.0%  (43/239) | 0.4%  (1/271) | --- | 49.2%  (208/423) |
| *20 males from each F1 hybrid specific phenotype were crossed to 20 WT *An. gambiae* Kisumu (AgKIS) females. Blk, black eye; Cd, cardinal eye. | | | | |

**Table S2. AgKIS F1 hybrid phenotype-specific backcrosses.** Crosses were performed between twenty F1 AgKIS hybrid males of different phenotypes generated from the female lineage, and twenty wild-type An. gambiae AgKIS females. The male hybrid phenotypes outcrossed to wild-type females included: small tear/CFP^+^, black-eye/CFP^+^, big tear/CFP^+^, and cardinal-eye/CFP^+^. The resulting F2 generation consisted of either black-eye/CFP^+^, small tear/CFP^+^ or black-eye/CFP^-^ progeny. Offspring with black-eye/CFP^+^ phenotypes predominated across all phenotype’s specific crosses except for the cardinal-eye crosses in which the proportions of CFP^+^ and CFP^-^ offspring were roughly equal. ^a^Blk: wild-type black-eye, ^b^CFP^+^: cyan fluorescent protein-positive, ^c^Cd: cardinal-eye red eye, ^d^CFP^-^: cyan fluorescent protein-negative. Frequencies are expressed as percentages with the numbers in parentheses the sums of the replicates.

|  | Parental phenotypes* | | | |
| --- | --- | --- | --- | --- |
| Phenotypes  Percentage (%)  (total population) | ♂ Blk/CFP^+^  x  ♀ AcMOP | ♂ Small Tear/CFP^+^  x  ♀ AcMOP | ♂ Big Tear/CFP^+^  x  ♀ AcMOP | ♂ Cd/CFP^+^  x  ♀ AcMOP |
| Blk eye/CFP^+^ | 98%  (481/491) | 97.6%  (124/127) | 97.7%  (130/133) | 81.2%  (147/181) |
| Small tear/CFP^+^ | 0.2%  (1/491) | 0.8%  (1/127) | 2.3%  (3/133) | --- |
| Big tear/CFP^+^ | 0.4%  (2/491) | --- | --- | --- |
| Blk eye/CFP^-^ | 1.4%  (7/491) | 1.6%  (2/127) | --- | 18.8%  (34/181) |
| *15 males from each F1 hybrid specific phenotype were crossed to 20 females WT *An. coluzzii* Mopti (AcMOP). Blk, black eye; Cd, cardinal eye. | | | | |

**Table S3. AcMOP F1 hybrid phenotype-specific backcrosses.** Crosses were performed between fifteen F1 AcMOP hybrid males of different phenotypes generated from the female lineage, and fifteen wild-type *An. coluzzii* AcMOP females. The male hybrid phenotypes outcrossed to wild-type females included: small tear/CFP^+^, black-eye/CFP^+^, big tear/CFP^+^, and cardinal-eye/CFP^+^. The resulting F2 generation consisted of either black-eye/CFP^+^, small tear/CFP^+^, big tear/CFP+, or black-eye/CFP^-^ progeny and offspring with black-eye/CFP^+^ phenotypes predominated across all phenotype’s specific crosses. ^a^Blk: wild-type black-eye, ^b^CFP^+^: cyan fluorescent protein-positive, ^c^Cd: cardinal-eye red eye, ^d^CFP^-^: cyan fluorescent protein-negative. Frequencies are expressed as percentages with the numbers in parentheses the sums of the replicates.

| A. F1 Cardinal-eye offspring, CFP^+^ phenotype | | | |
| --- | --- | --- | --- |
| Introduction or Introgression wild-type lines | F1 cardinal eye phenotypes | Total Sequenced | Genotypes |
| AgKIS | 7.8% | 37 | 100% drive/NHEJ allele |
| AgNDO | 8.0% | 47 | 100% drive/NHEJ allele |
| AgZAN | 7.2% | 50 | 94% drive/NHEJ allele |
| AcMOP | 11.7% | 69 | 100% drive/NHEJ allele |
| AaDON | 7.3% | 60 | 100% drive/NHEJ allele |
| B1. Male gene drive lineage F1 Black eye offspring, CFP^+^ phenotype | | | |
| AgKIS | 99.6% | 60 | 100% drive/WT allele |
| AgNDO | 99.9% | 60 | 100% drive/WT allele |
| AgZAN | 99.3% | 60 | 100% drive/WT allele |
| AcMOP | 99.6% | 60 | 100% drive/WT allele |
| AaDON | 99.97% | 60 | 100% drive/WT allele |
| B2. Female gene drive lineage F1 Black eye offspring, CFP^+^ phenotype | | | |
| AgKIS | 22.3% | 60 | 95.0% drive/WT allele  5.0% drive/NHEJ allele |
| AgNDO | 27.0% | 40 | 77.5% drive/WT allele  22.5% drive/NHEJ allele |
| AgZAN | 32.7% | 59 | 81.4% drive/WT allele  18.6% drive/NHEJ allele |
| AcMOP | 21.6% | 60 | 68.3% drive/WT allele  31.7% drive/NHEJ allele |
| AaDON | 33.8% | 57 | 86.0% drive/WT allele  14.0% drive/NHEJ allele |

**Table S4. Phenotypes and genotypes in F1 hybrids from experimental crosses from AgNosCd-1 male and female lineages.** F1 hybrids were created from three reps of either AgNOSCd-1 homozygous gene-drive males or females out-crossed with wild-types of the opposite sex from each strain: *An. gambiae* Kisumu (AgKIS), *An. gambiae* Ndokayo (AgNDO), *An. gambiae* Zanu (AgZan), *An. coluzzii* Mopti (AcMOP), and *An. arabiensis* Dongola (AaDON). The F1 progeny were screened for unique eye phenotypes and a sample of each phenotype from each rep was sequenced to determine genotypes. A) The F1 cardinal-eye/CFP^+^ offspring, from all female lineage replicates were found to contain one drive allele and one NHEJ allele except for three cardinal eye/CFP^+^ F1 AgZAN hybrids that showed evidence of a drive allele and another unidentifiable allele. B1) Black eye F1 hybrid progeny from all male lineage crosses were found to have one drive allele and one wildtype allele. B2) Black eye F1 hybrid progeny from all female lineage crosses showed evidence of both wild-type and NHEJ alleles along with a drive allele. The quantity of wild-type and NHEJ alleles contained within the black eye hybrid progeny varied amongst the different hybrid strains. ^a^CFP^+^: cyan fluorescent protein-positive, ^b^NHEJ: non-homologous end joining allele, ^c^WT: wild-type allele. Frequencies are expressed as percentages with the numbers in parentheses the sums of the replicates

| Introduction or Introgression  F1 hybrids | F2 black eye phenotypes | Total Sequenced | Genotypes |
| --- | --- | --- | --- |
| Male gene drive lineages | | | |
| AgNosCd-1/AgKIS | 3.7% | 21 | 100% drive/WT allele |
| AgNosCd-1/AgNDO | 6.5% | 25 | 100% drive/WT allele |
| AgNosCd-1/AgZAN | 1.5% | 9 | 100% drive/WT allele |
| AgNosCd-1/AcMOP | 0.3% | 8 | 100% drive/WT allele |
| Female drive lineages | | | |
| AgNosCd-1/AgKIS | 11.8% | 15 | 13% drive/WT allele  87% drive/NHEJ allele |
| AgNosCd-1/AgNDO | 8.3% | 15 | 27% drive/WT allele  73% drive/NHEJ allele |
| AgNosCd-1/AgZAN | 8.3% | 21 | 10% drive/WT allele  90% drive/NHEJ allele |
| AgNosCd-1/AcMOP | 6.2% | 24 | 29% drive/WT allele  71% drive/NHEJ allele |

**Table S5. Black-eye, CFP^+^ phenotypes and genotypes in F2 hybrid offspring of introduction and introgression experimental crosses.** F2 hybrids were created by allowing three replicates of a subset of 150 randomly selected F1 hybrid progeny from each strain from each male and female lineage to intercross. A sample of the black eye/CFP^+^ offspring from each cross, lineage and replicate were then sequenced to determine the F2 offspring’s genotype. Frequencies are expressed as percentages. The male gene drive lineages from all strains were found to have one drive allele and one wild-type allele, no evidence of NHEJ alleles were detected. The female drive lineages were found to have either wild-type or NHEJ alleles along with a drive allele and NHEJ alleles were found to be more frequent than wild-type alleles in all strains. ^a^CFP^+^: cyan fluorescent protein-positive, ^b^WT: wild-type allele, ^c^NHEJ: non-homologous end joining allele, ^1^total number of black eye/CFP^+^ phenotypes divided by total number of offspring (all phenotypes). Frequencies are expressed as percentages.

| Introduction or Introgression F1 hybrids | F2 cardinal/CFP^+^ phenotypes | Total Sequenced | Genotypes |
| --- | --- | --- | --- |
| Male gene drive lineages | | | |
| AgNosCd-1/AgKIS | 96.2% | 60 | 100% drive homozygous |
| AgNosCd-1/AgNDO | 93.4% | 60 | 100% drive homozygous |
| AgNosCd-1/AgZAN | 98.5% | 60 | 100% drive homozygous |
| AgNosCd-1/AcMOP | 99.7% | 60 | 100% drive homozygous |
| Female gene drive lineages | | | |
| AgNosCd-1/AgKIS | 87.6% | 60 | 93.4% drive homozygous  6.6% drive/NHEJ allele |
| AgNosCd-1/AgNDO | 90.5% | 60 | 76.7% drive homozygous  23.3% drive/NHEJ allele |
| AgNosCd-1/AgZAN | 90.8% | 60 | 76.7% drive homozygous  23.3% drive/NHEJ allele |
| AgNosCd-1/AcMOP | 93.0% | 60 | 81.7% drive homozygous  18.3% drive/NHEJ allele |

**Table S6. Cardinal-eye, CFP^+^ phenotypes and genotypes in F2 hybrid offspring of introduction and introgression experimental crosses.** F2 hybrids were created by allowing three replicates of a subset of 150 randomly selected F1 hybrid progeny from each strain from each male and female lineage to intercross. A sample of the cardinal eye/CFP^+^ offspring from each cross, lineage and replicate were then sequenced to determine the F2 offspring’s genotype. The male gene drive lineages from all strains were found to have only drive alleles, no evidence of NHEJ alleles were detected. The female drive lineages were found to have either drive alleles or NHEJ alleles and drive alleles were found to be more frequent than NHEJ alleles in all strains. ^a^CFP^+^: cyan fluorescent protein-positive, ^b^NHEJ: non-homologous end joining allele, ^1^total number of cardinal-eye/CFP^+^ phenotypes divided by total number of offspring (all phenotypes). Frequencies are expressed as percentages with the numbers in parentheses the sums of the replicates

| Introduction or Introgression F1 hybrids | F2 phenotypes | Total  Sequenced | Genotypes |
| --- | --- | --- | --- |
| Black eye/CFP^-^ | | | |
| AgNosCd-1/AgKIS | 0.5% | 15 | 33% WT/NHEJ allele  67% NHEJ/NHEJ allele |
| AgNosCd-1/AgNDO | 1.0% | 21 | 33% WT/NHEJ allele  67% NHEJ/NHEJ allele |
| AgNosCd-1/AgZAN | 0.2% | 7 | 86% WT/NHEJ allele  14% NHEJ/NHEJ allele |
| AgNosCd-1/AcMOP | 0.3% | 8 | 50% WT/NHEJ allele  50% NHEJ/NHEJ allele |
| Cardinal eye/CFP^-^ | | | |
| AgNosCd-1/AgKIS | 0.1% | 3 | 100% NHEJ/NHEJ allele |
| AgNosCd-1/AgNDO | 0.2% | 11 | 100% NHEJ/NHEJ allele |
| AgNosCd-1/AgZAN | 0.6% | 17 | 100% NHEJ/NHEJ allele |
| AgNosCd-1/AcMOP | 0.5% | 16 | 100% NHEJ/NHEJ allele |

**Table S7. Black and cardinal eye, CFP^-^ genotypes in F2 hybrids from female AgNosCd-1 lineage experimental crosses.** F2 hybrids were created by allowing three replicates of a subset of 150 randomly selected F1 hybrid progeny from each strain from each male and female lineage to intercross. A sample of the black eye/CFP^-^ and cardinal eye/CFP^-^ offspring from each cross and replicate from female lineages were then sequenced to determine the F2 offspring’s genotype. The male gene drive lineages were not sequenced as no CFP^-^ individuals were recovered from the F2 generation from any strain. The black eye/CFP^-^ individuals from all strains were found to have both NHEJ alleles and wild-type alleles, NHEJ alleles were more common and no individual from any strain had two copies of a wild-type allele causing the black eye/CFP^-^ phenotype. The cardinal eye/CFP^-^ individuals were found to have only NHEJ alleles in all strains. ^a^CFP^-^: cyan fluorescent protein-negative, ^b^WT: wild-type allele, ^c^NHEJ: non-homologous end joining allele, ^1^total number of black eye/CFP^-^ or cardinal eye/CFP^-^ phenotypes divided by total number of offspring (all phenotypes). Frequencies are expressed as percentages with the numbers in parentheses the sums of the replicates

| Non-drive alleles of F2 (Blk/CFP^+^) offspring from ♂AgNosCd-1 x ♀AgNDO test crosses | | | | |
| --- | --- | --- | --- | --- |
| Frequency | Rep. 1 | Rep. 2 | Rep. 3 | Relative Frequency |
| WT conserved allele | 0.375  (3/8) | 1.0  (5/5) | 0.583  (7/12) | 0.60  (15/25) |
| WT SNP allele (both haplotypes | 0.625  (5/8) | 0.0  (0/5) | 0.417  (5/12) | 0.40  (10/25) |

**Table S8. Sequencing results of black-eye/CFP^+^ F2 offspring from ♂AgNosCd-1 x ♀AgNDO introductory experimental crosses.** To determine whether the presence of a target-site SNP affected the drive inheritance in F2 progeny from male drive lineages, the frequency of canonical WT and WT SNP alleles in the F2 progeny from each replicate was determined. The frequency of these alleles in the F2 offspring could then be compared with the frequency of these alleles in the AgNDO mothers that contributed alleles to the AgNosCd-1 male lineage experimental crosses.

| **Name of primer** | **Sequence (5’– 3’)** | **Annealing Temperature** | **Extension time** | **Amplicon length (bp)** |
| --- | --- | --- | --- | --- |
| Ak5 | GTACTCGTACGGTCGCTCCTTA | 61^o^C | 1 min | 526 |
| Ak21 | TCATTAGTTTCCCGCTGGTC |  |  |  |
| CO159 | GCAAGGCTGTTGTGGTGCCGG | 72^o^C | 2 min | 2192 |
| CO160 | CGGTACCGGCACCGTAGGCACC |  |  |  |
| TP179 | GAAAGTCACCCTCGTCCTACG | 60^o^C | 2 min | 1936 |
| TP302 | GTTATGTCGTGATCGAGAAACTG |  |  |  |
| TP180 | GATTCCGGATCGACGATGCG | 61^o^C | 2 min | 1808 |
| TP217 | GCTTGTTTGAATTGAATTGTCGC |  |  |  |
| CO94 | CCCGAGCACACACTTTCTTGC | 61^o^C | 2 min | 2334 |
| bp: base pairs | | | | |

**Table S9. List of oligonucleotide primers.** Primers used to amplify regions of interest related to both the drive system and its respective target site.

**Figure S1. AgNosCd-1 gene drive system and eye phenotypes.** A) schematic representation of the AgNosCd-1 gene drive and its components. Modified from Carballar-Lejarazú, *et al* [5], B) Cardinal vs Big Tear vs Small Tear phenotypes. Eye phenotypes indicative of maternal-effect and leaky expression present in F1 hybrids. The cardinal-eye phenotype lacks any wild-type pigmentation at the pupal stage, the small tear has a reduction in pigmentation in <25% of the eye due to mosaicism at the *cardinal* gene, the big tear has a loss of pigmentation in ≥25% of the eye due to mosaicism at the *cardinal* gene. The red (cardinal) ovals behind the developing adult eyes are the remnants of the larval eyes. Presence of the CFP dominant marker (CFP^+^) indicates the integration of the AgNosCd-1 gene drive in the mosquito genome.


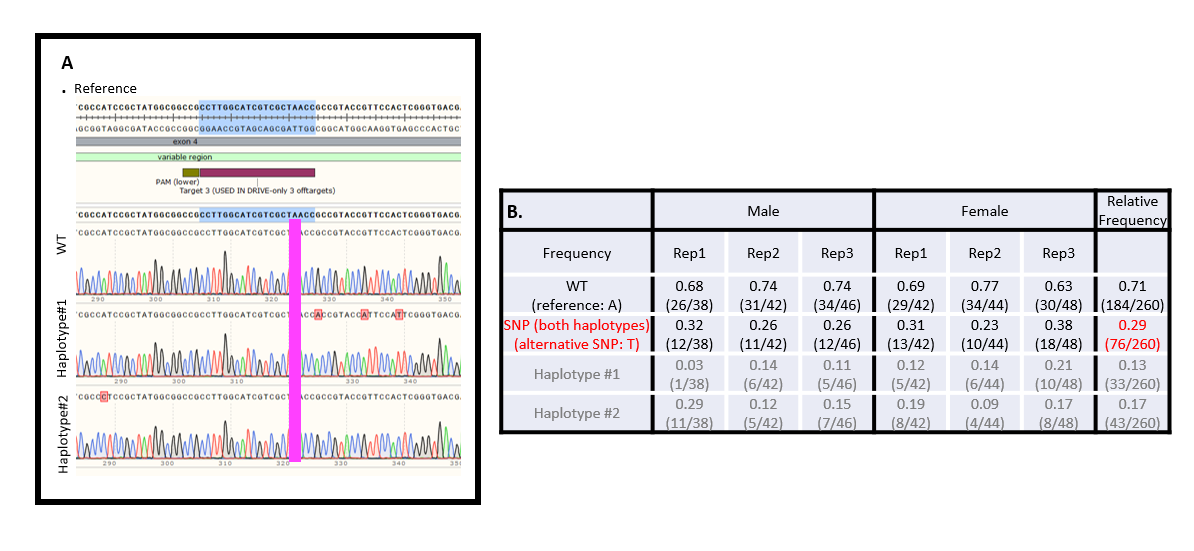


**Figure S2.** ***cardinal* target-site variation in AgNDO wild-type males and females used in introductory crosses to AgNosCd-1.** A. The canonical reference sequence for the *cardinal* gene target site is presented in the top line, the following sequences WT, haplotype #1, and haplotype #2 were all discovered in the AgNDO parental samples. B. The frequencies of WT, haplotype #1 and haplotype #2 alleles discovered in males and females. A relative frequency of the WT alleles and SNP alleles from all three replicates was calculated.


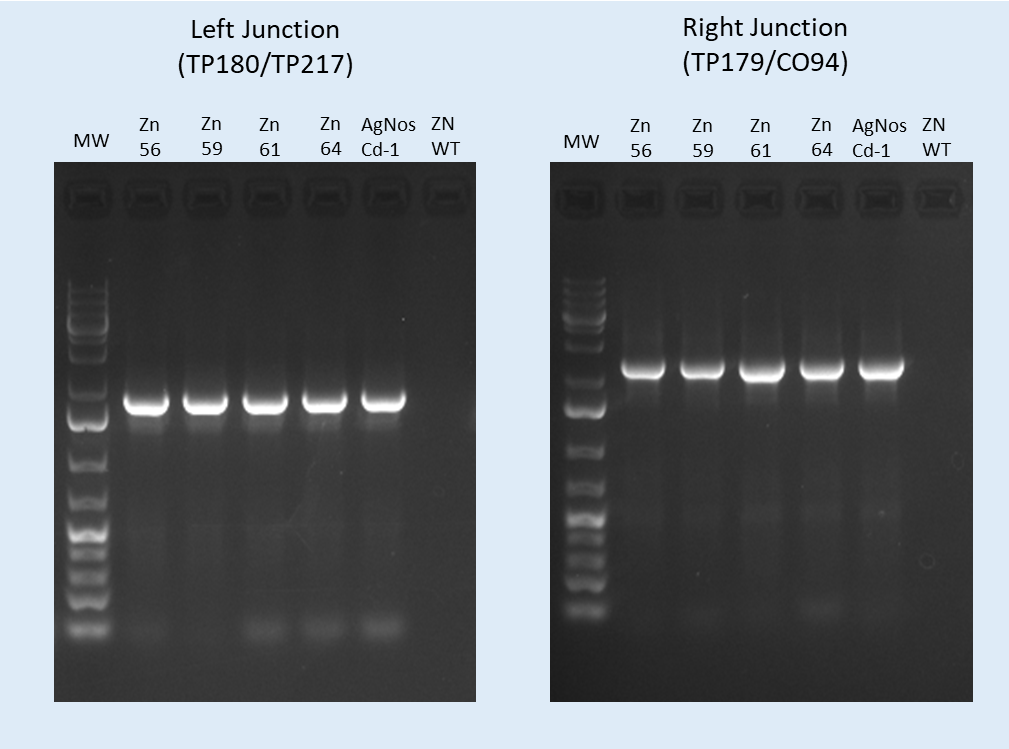
**
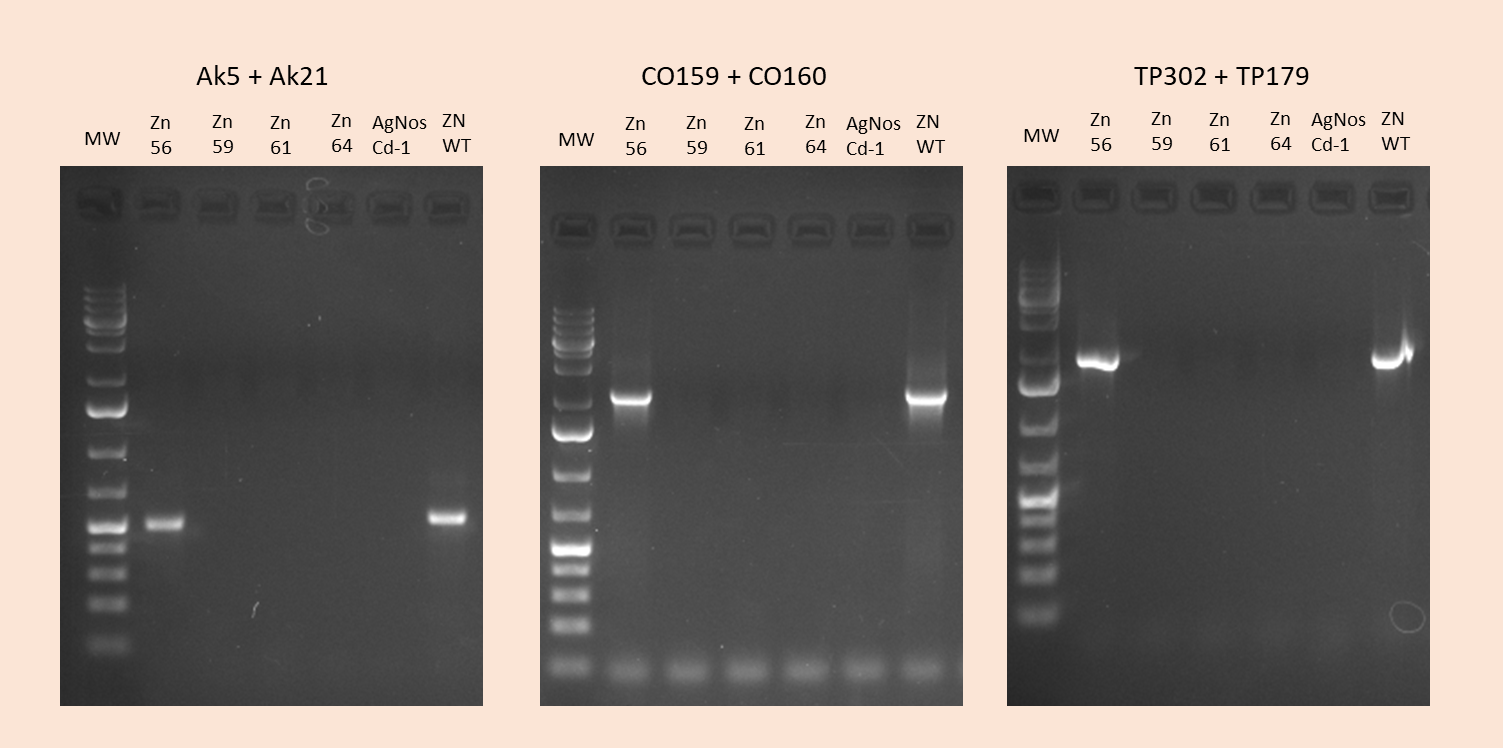
Figure S3**. **Unidentified *cardinal* allele in *cd*^-^/CFP^+^ F2 individuals from ♀AgNosCd-1 x ♂AgZAN F1 hybrid intercrosses.** Three cardinal-eye/CFP^+^ individuals (ZN59, 61 and 64) were verified to contain a drive allele (blue box), whereas the presence of a cardinal allele was not able to be identified with three different pairs of primers (Orange box). A *cd*^-^/CFP^+^ (ZN56) individual from the same cross, an AgNosCd-1 individual and an AgZAN wild-type (ZN WT) individual served as controls.

**Sequencing data set.** Individual mosquitoes were extracted and analyzed separately. Amplicon sequences of wild-type allele or NHEJ allele were uploaded into GenBank.

| **Sample Name** | **Gen Bank ID** | **Parent Cross Description** | **Replicate** | **Generation** | ***Phenotype*** | **Sex** | **Genotype** |
| --- | --- | --- | --- | --- | --- | --- | --- |
|  |  |  |  |  |  |  |  |
| ZN22 | OR693508 | *An. gambiae* Zanu male x AgNosCd-1 female | Rep 1 | F1 | *Cardinal/CFP+* | Female | Drive/NHEJ |
|  | OR693509 |  |  |  |  |  |  |
| ZN23 | OR693510 | *An. gambiae* Zanu male x AgNosCd-1 female | Rep 1 | F1 | *Cardinal/CFP+* | Female | Drive/NHEJ |
|  | OR693511 |  |  |  |  |  |  |
| ZN24 | OR693512 | *An. gambiae* Zanu male x AgNosCd-1 female | Rep 1 | F1 | *Cardinal/CFP+* | Female | Drive/NHEJ |
|  | OR693513 |  |  |  |  |  |  |
| ZN25 | OR693514 | *An. gambiae* Zanu male x AgNosCd-1 female | Rep 1 | F1 | *Cardinal/CFP+* | Female | Drive/NHEJ |
|  | OR693515 |  |  |  |  |  |  |
| ZN26 | OR693516 | *An. gambiae* Zanu male x AgNosCd-1 female | Rep 1 | F1 | *Cardinal/CFP+* | Female | Drive/NHEJ |
|  | OR693517 |  |  |  |  |  |  |
| ZN27 | OR693518 | *An. gambiae* Zanu male x AgNosCd-1 female | Rep 1 | F1 | *Cardinal/CFP+* | Female | Drive/NHEJ |
|  | OR693519 |  |  |  |  |  |  |
| ZN28 | OR693520 | *An. gambiae* Zanu male x AgNosCd-1 female | Rep 1 | F1 | *Cardinal/CFP+* | Female | Drive/NHEJ |
|  | OR693521 |  |  |  |  |  |  |
| ZN29 | OR693522 | *An. gambiae* Zanu male x AgNosCd-1 female | Rep 1 | F1 | *Cardinal/CFP+* | Female | Drive/NHEJ |
|  | OR693523 |  |  |  |  |  |  |
| ZN30 | OR693524 | *An. gambiae* Zanu male x AgNosCd-1 female | Rep 1 | F1 | *Cardinal/CFP+* | Female | Drive/NHEJ |
|  | OR693525 |  |  |  |  |  |  |
| ZN31 | OR693526 | *An. gambiae* Zanu male x AgNosCd-1 female | Rep 1 | F1 | *Cardinal/CFP+* | male | Drive/NHEJ |
|  | OR693527 |  |  |  |  |  |  |
| ZN32 | OR693528 | *An. gambiae* Zanu male x AgNosCd-1 female | Rep 1 | F1 | *Cardinal/CFP+* | Male | Drive/NHEJ |
|  | OR693529 |  |  |  |  |  |  |
| ZN56 | OR693530 | *An. gambiae* Zanu male x AgNosCd-1 female | Rep 1 | F1 | *Cardinal/CFP+* | male | Drive/NHEJ |
| ZN57 | OR693531 | *An. gambiae* Zanu male x AgNosCd-1 female | Rep 1 | F1 | *Cardinal/CFP+* | Male | Drive/NHEJ |
|  | OR693532 |  |  |  |  |  |  |
| ZN58 | OR693533 | *An. gambiae* Zanu male x AgNosCd-1 female | Rep 1 | F1 | *Cardinal/CFP+* | male | Drive/NHEJ |
| ZN59 |  | *An. gambiae* Zanu male x AgNosCd-1 female | Rep 2 | F1 | *Cardinal/CFP+* | Male | N/A |
| ZN60 | OR693534 | *An. gambiae* Zanu male x AgNosCd-1 female | Rep 2 | F1 | *Cardinal/CFP+* | male | Drive/NHEJ |
| ZN61 |  | *An. gambiae* Zanu male x AgNosCd-1 female | Rep 2 | F1 | *Cardinal/CFP+* | Female | N/A |
| ZN62 | OR693535 | *An. gambiae* Zanu male x AgNosCd-1 female | Rep 2 | F1 | *Cardinal/CFP+* | Female | Drive/NHEJ |
|  | OR693536 |  |  |  |  |  |  |
| ZN63 | OR693537 | *An. gambiae* Zanu male x AgNosCd-1 female | Rep 2 | F1 | *Cardinal/CFP+* | Female | Drive/NHEJ |
|  | OR693538 |  |  |  |  |  |  |
| ZN64 |  | *An. gambiae* Zanu male x AgNosCd-1 female | Rep 2 | F1 | *Cardinal/CFP+* | Female | N/A |
| ZN65 | OR693539 | *An. gambiae* Zanu male x AgNosCd-1 female | Rep 2 | F1 | *Cardinal/CFP+* | Female | Drive/NHEJ |
| ZN66 | OR693540 | *An. gambiae* Zanu male x AgNosCd-1 female | Rep 2 | F1 | *Cardinal/CFP+* | Male | Drive/NHEJ |
|  | OR693541 |  |  |  |  |  |  |
| ZN67 | OR693542 | *An. gambiae* Zanu male x AgNosCd-1 female | Rep 2 | F1 | *Cardinal/CFP+* | male | Drive/NHEJ |
|  | OR693543 |  |  |  |  |  |  |
| ZN68 | OR693544 | *An. gambiae* Zanu male x AgNosCd-1 female | Rep 2 | F1 | *Cardinal/CFP+* | Male | Drive/NHEJ |
|  | OR693545 |  |  |  |  |  |  |
| ZN69 | OR693546 | *An. gambiae* Zanu male x AgNosCd-1 female | Rep 2 | F1 | *Cardinal/CFP+* | male | Drive/NHEJ |
|  | OR693547 |  |  |  |  |  |  |
| ZN70 | OR693548 | *An. gambiae* Zanu male x AgNosCd-1 female | Rep 2 | F1 | *Cardinal/CFP+* | Male | Drive/NHEJ |
|  | OR693549 |  |  |  |  |  |  |
| ZN71 | OR693550 | *An. gambiae* Zanu male x AgNosCd-1 female | Rep 2 | F1 | *Cardinal/CFP+* | male | Drive/NHEJ |
|  | OR693551 |  |  |  |  |  |  |
| ZN72 | OR693552 | *An. gambiae* Zanu male x AgNosCd-1 female | Rep 2 | F1 | *Cardinal/CFP+* | Male | Drive/NHEJ |
| ZN73 | OR693553 | *An. gambiae* Zanu male x AgNosCd-1 female | Rep 2 | F1 | *Cardinal/CFP+* | male | Drive/NHEJ |
| ZN74 | OR693554 | *An. gambiae* Zanu male x AgNosCd-1 female | Rep 2 | F1 | *Cardinal/CFP+* | Female | Drive/NHEJ |
| ZN75 | OR693555 | *An. gambiae* Zanu male x AgNosCd-1 female | Rep 2 | F1 | *Cardinal/CFP+* | Female | Drive/NHEJ |
|  | OR693556 |  |  |  |  |  |  |
| ZN76 | OR693557 | *An. gambiae* Zanu male x AgNosCd-1 female | Rep 2 | F1 | *Cardinal/CFP+* | Female | Drive/NHEJ |
|  | OR693558 |  |  |  |  |  |  |
| ZN77 | OR693559 | *An. gambiae* Zanu male x AgNosCd-1 female | Rep 2 | F1 | *Cardinal/CFP+* | Female | Drive/NHEJ |
| ZN78 | OR693560 | *An. gambiae* Zanu male x AgNosCd-1 female | Rep 2 | F1 | *Cardinal/CFP+* | Female | Drive/NHEJ |
| ZN79 | OR693561 | *An. gambiae* Zanu male x AgNosCd-1 female | Rep 2 | F1 | *Cardinal/CFP+* | Female | Drive/NHEJ |
| ZN80 | OR693562 | *An. gambiae* Zanu male x AgNosCd-1 female | Rep 2 | F1 | *Cardinal/CFP+* | Female | Drive/NHEJ |
| ZN81 | OR693563 | *An. gambiae* Zanu male x AgNosCd-1 female | Rep 2 | F1 | *Cardinal/CFP+* | Female | Drive/NHEJ |
| ZN82 | OR693564 | *An. gambiae* Zanu male x AgNosCd-1 female | Rep 2 | F1 | *Cardinal/CFP+* | Female | Drive/NHEJ |
|  | OR693565 |  |  |  |  |  |  |
| ZN83 | OR693566 | *An. gambiae* Zanu male x AgNosCd-1 female | Rep 2 | F1 | *Cardinal/CFP+* | Female | Drive/NHEJ |
| ZN84 | OR693567 | *An. gambiae* Zanu male x AgNosCd-1 female | Rep 2 | F1 | *Cardinal/CFP+* | Female | Drive/NHEJ |
|  | OR693568 |  |  |  |  |  |  |
| ZN123 | OR693602 | *An. gambiae* Zanu male x AgNosCd-1 female | Rep 3 | F1 | *Cardinal/CFP+* | Male | Drive/NHEJ |
|  | OR693603 |  |  |  |  |  |  |
| ZN124 | OR693604 | *An. gambiae* Zanu male x AgNosCd-1 female | Rep 3 | F1 | *Cardinal/CFP+* | Male | Drive/NHEJ |
|  | OR693605 |  |  |  |  |  |  |
| ZN125 | OR693606 | *An. gambiae* Zanu male x AgNosCd-1 female | Rep 3 | F1 | *Cardinal/CFP+* | Female | Drive/NHEJ |
| ZN126 | OR693607 | *An. gambiae* Zanu male x AgNosCd-1 female | Rep 3 | F1 | *Cardinal/CFP+* | Male | Drive/NHEJ |
|  | OR693608 |  |  |  |  |  |  |
| ZN127 | OR693609 | *An. gambiae* Zanu male x AgNosCd-1 female | Rep 3 | F1 | *Cardinal/CFP+* | Female | Drive/NHEJ |
| ZN128 | OR693610 | *An. gambiae* Zanu male x AgNosCd-1 female | Rep 3 | F1 | *Cardinal/CFP+* | Female | Drive/NHEJ |
| ZN129 | OR693611 | *An. gambiae* Zanu male x AgNosCd-1 female | Rep 3 | F1 | *Cardinal/CFP+* | Female | Drive/NHEJ |
|  | OR693612 |  |  |  |  |  |  |
| ZN130 | OR693613 | *An. gambiae* Zanu male x AgNosCd-1 female | Rep 3 | F1 | *Cardinal/CFP+* | Female | Drive/NHEJ |
| ZN131 | OR693614 | *An. gambiae* Zanu male x AgNosCd-1 female | Rep 3 | F1 | *Cardinal/CFP+* | Female | Drive/NHEJ |
|  | OR693615 |  |  |  |  |  |  |
| ZN132 | OR693616 | *An. gambiae* Zanu male x AgNosCd-1 female | Rep 3 | F1 | *Cardinal/CFP+* | Female | Drive/NHEJ |
| ZN421 | OR693651 | *An. gambiae* Zanu male x AgNosCd-1 female | Rep1 | F1 | ***Black****/****CFP+*** | Male | Drive/WT |
| ZN422 | OR693652 | *An. gambiae* Zanu male x AgNosCd-1 female | Rep1 | F1 | ***Black****/****CFP+*** | Male | Drive/WT |
| ZN423 | OR693653 | *An. gambiae* Zanu male x AgNosCd-1 female | Rep1 | F1 | ***Black****/****CFP+*** | Male | Drive/WT |
| ZN424 | OR693654 | *An. gambiae* Zanu male x AgNosCd-1 female | Rep1 | F1 | ***Black****/****CFP+*** | Male | Drive/NHEJ |
| ZN425 | OR693655 | *An. gambiae* Zanu male x AgNosCd-1 female | Rep1 | F1 | ***Black****/****CFP+*** | Male | Drive/WT |
| ZN426 | OR693656 | *An. gambiae* Zanu male x AgNosCd-1 female | Rep1 | F1 | ***Black****/****CFP+*** | Male | Drive/NHEJ |
| ZN427 | OR693657 | *An. gambiae* Zanu male x AgNosCd-1 female | Rep1 | F1 | ***Black****/****CFP+*** | Male | Drive/WT |
| ZN428 | OR693658 | *An. gambiae* Zanu male x AgNosCd-1 female | Rep1 | F1 | ***Black****/****CFP+*** | Male | Drive/WT |
| ZN429 | OR693659 | *An. gambiae* Zanu male x AgNosCd-1 female | Rep1 | F1 | ***Black****/****CFP+*** | Male | Drive/NHEJ |
|  | OR693660 |  |  |  |  |  |  |
| ZN430 | OR693661 | *An. gambiae* Zanu male x AgNosCd-1 female | Rep1 | F1 | ***Black****/****CFP+*** | Male | Drive/WT |
| ZN431 | OR693662 | *An. gambiae* Zanu male x AgNosCd-1 female | Rep1 | F1 | ***Black****/****CFP+*** | Female | Drive/NHEJ |
| ZN432 | OR693663 | *An. gambiae* Zanu male x AgNosCd-1 female | Rep1 | F1 | ***Black****/****CFP+*** | Female | Drive/WT |
| ZN433 | OR693664 | *An. gambiae* Zanu male x AgNosCd-1 female | Rep1 | F1 | ***Black****/****CFP+*** | Female | Drive/WT |
| ZN434 | OR693665 | *An. gambiae* Zanu male x AgNosCd-1 female | Rep1 | F1 | ***Black****/****CFP+*** | Female | Drive/NHEJ |
|  | OR693666 |  |  |  |  |  |  |
| ZN435 | OR693667 | *An. gambiae* Zanu male x AgNosCd-1 female | Rep1 | F1 | ***Black****/****CFP+*** | Female | Drive/WT |
| ZN436 | OR693668 | *An. gambiae* Zanu male x AgNosCd-1 female | Rep1 | F1 | ***Black****/****CFP+*** | Female | Drive/WT |
| ZN437 | OR693669 | *An. gambiae* Zanu male x AgNosCd-1 female | Rep1 | F1 | ***Black****/****CFP+*** | Female | Drive/NHEJ |
| ZN438 | OR693670 | *An. gambiae* Zanu male x AgNosCd-1 female | Rep1 | F1 | ***Black****/****CFP+*** | Female | Drive/WT |
| ZN439 | OR693671 | *An. gambiae* Zanu male x AgNosCd-1 female | Rep1 | F1 | ***Black****/****CFP+*** | Female | Drive/WT |
| ZN440 | OR693672 | *An. gambiae* Zanu male x AgNosCd-1 female | Rep1 | F1 | ***Black****/****CFP+*** | Female | Drive/WT |
| ZN441 | OR693673 | *An. gambiae* Zanu male x AgNosCd-1 female | Rep2 | F1 | ***Black****/****CFP+*** | Male | Drive/WT |
| ZN442 | OR693674 | *An. gambiae* Zanu male x AgNosCd-1 female | Rep2 | F1 | ***Black****/****CFP+*** | Male | Drive/WT |
| ZN443 | OR693675 | *An. gambiae* Zanu male x AgNosCd-1 female | Rep2 | F1 | ***Black****/****CFP+*** | Male | Drive/NHEJ |
|  | OR693676 |  |  |  |  |  |  |
| ZN444 | OR693677 | *An. gambiae* Zanu male x AgNosCd-1 female | Rep2 | F1 | ***Black****/****CFP+*** | Male | Drive/WT |
| ZN446 | OR693678 | *An. gambiae* Zanu male x AgNosCd-1 female | Rep2 | F1 | ***Black****/****CFP+*** | Male | Drive/NHEJ |
| ZN447 | OR693679 | *An. gambiae* Zanu male x AgNosCd-1 female | Rep2 | F1 | ***Black****/****CFP+*** | Male | Drive/WT |
| ZN448 | OR693680 | *An. gambiae* Zanu male x AgNosCd-1 female | Rep2 | F1 | ***Black****/****CFP+*** | Male | Drive/WT |
| ZN449 | OR693681 | *An. gambiae* Zanu male x AgNosCd-1 female | Rep2 | F1 | ***Black****/****CFP+*** | Male | Drive/WT |
| ZN450 | OR693682 | *An. gambiae* Zanu male x AgNosCd-1 female | Rep2 | F1 | ***Black****/****CFP+*** | Male | Drive/WT |
| ZN451 | OR693683 | *An. gambiae* Zanu male x AgNosCd-1 female | Rep2 | F1 | ***Black****/****CFP+*** | Female | Drive/WT |
| ZN452 | OR693684 | *An. gambiae* Zanu male x AgNosCd-1 female | Rep2 | F1 | ***Black****/****CFP+*** | Female | Drive/WT |
| ZN453 | OR693685 | *An. gambiae* Zanu male x AgNosCd-1 female | Rep2 | F1 | ***Black****/****CFP+*** | Female | Drive/WT |
| ZN454 | OR693686 | *An. gambiae* Zanu male x AgNosCd-1 female | Rep2 | F1 | ***Black****/****CFP+*** | Female | Drive/WT |
| ZN455 | OR693687 | *An. gambiae* Zanu male x AgNosCd-1 female | Rep2 | F1 | ***Black****/****CFP+*** | Female | Drive/WT |
| ZN456 | OR693688 | *An. gambiae* Zanu male x AgNosCd-1 female | Rep2 | F1 | ***Black****/****CFP+*** | Female | Drive/WT |
| ZN457 | OR693689 | *An. gambiae* Zanu male x AgNosCd-1 female | Rep2 | F1 | ***Black****/****CFP+*** | Female | Drive/WT |
| ZN458 | OR693690 | *An. gambiae* Zanu male x AgNosCd-1 female | Rep2 | F1 | ***Black****/****CFP+*** | Female | Drive/WT |
| ZN459 | OR693691 | *An. gambiae* Zanu male x AgNosCd-1 female | Rep2 | F1 | ***Black****/****CFP+*** | Female | Drive/WT |
| ZN460 | OR693692 | *An. gambiae* Zanu male x AgNosCd-1 female | Rep2 | F1 | ***Black****/****CFP+*** | Female | Drive/WT |
| ZN461 | OR693693 | *An. gambiae* Zanu male x AgNosCd-1 female | Rep3 | F1 | ***Black****/****CFP+*** | Male | Drive/WT |
| ZN462 | OR693694 | *An. gambiae* Zanu male x AgNosCd-1 female | Rep3 | F1 | ***Black****/****CFP+*** | Male | Drive/WT |
| ZN463 | OR693695 | *An. gambiae* Zanu male x AgNosCd-1 female | Rep3 | F1 | ***Black****/****CFP+*** | Male | Drive/NHEJ |
| ZN464 | OR693696 | *An. gambiae* Zanu male x AgNosCd-1 female | Rep3 | F1 | ***Black****/****CFP+*** | Male | Drive/WT |
| ZN465 | OR693697 | *An. gambiae* Zanu male x AgNosCd-1 female | Rep3 | F1 | ***Black****/****CFP+*** | Male | Drive/NHEJ |
| ZN466 | OR693698 | *An. gambiae* Zanu male x AgNosCd-1 female | Rep3 | F1 | ***Black****/****CFP+*** | Male | Drive/WT |
| ZN467 | OR693699 | *An. gambiae* Zanu male x AgNosCd-1 female | Rep3 | F1 | ***Black****/****CFP+*** | Male | Drive/WT |
| ZN468 | OR693700 | *An. gambiae* Zanu male x AgNosCd-1 female | Rep3 | F1 | ***Black****/****CFP+*** | Male | Drive/WT |
| ZN469 | OR693701 | *An. gambiae* Zanu male x AgNosCd-1 female | Rep3 | F1 | ***Black****/****CFP+*** | Male | Drive/WT |
| ZN470 | OR693702 | *An. gambiae* Zanu male x AgNosCd-1 female | Rep3 | F1 | ***Black****/****CFP+*** | Male | Drive/WT |
| ZN471 | OR693703 | *An. gambiae* Zanu male x AgNosCd-1 female | Rep3 | F1 | ***Black****/****CFP+*** | Female | Drive/WT |
| ZN472 | OR693704 | *An. gambiae* Zanu male x AgNosCd-1 female | Rep3 | F1 | ***Black****/****CFP+*** | Female | Drive/WT |
| ZN473 | OR693705 | *An. gambiae* Zanu male x AgNosCd-1 female | Rep3 | F1 | ***Black****/****CFP+*** | Female | Drive/WT |
| ZN474 | OR693706 | *An. gambiae* Zanu male x AgNosCd-1 female | Rep3 | F1 | ***Black****/****CFP+*** | Female | Drive/WT |
| ZN475 | OR693707 | *An. gambiae* Zanu male x AgNosCd-1 female | Rep3 | F1 | ***Black****/****CFP+*** | Female | Drive/WT |
| ZN476 | OR693708 | *An. gambiae* Zanu male x AgNosCd-1 female | Rep3 | F1 | ***Black****/****CFP+*** | Female | Drive/WT |
| ZN477 | OR693709 | *An. gambiae* Zanu male x AgNosCd-1 female | Rep3 | F1 | ***Black****/****CFP+*** | Female | Drive/WT |
| ZN478 | OR693710 | *An. gambiae* Zanu male x AgNosCd-1 female | Rep3 | F1 | ***Black****/****CFP+*** | Female | Drive/WT |
| ZN479 | OR693711 | *An. gambiae* Zanu male x AgNosCd-1 female | Rep3 | F1 | ***Black****/****CFP+*** | Female | Drive/WT |
| ZN480 | OR693712 | *An. gambiae* Zanu male x AgNosCd-1 female | Rep3 | F1 | ***Black****/****CFP+*** | Female | Drive/WT |
| ZN481 | OR693713 | AgNosCd-1 male x *An. gambiae* Zanu female | Rep1 | F1 | ***Black****/****CFP+*** | Male | Drive/WT |
| ZN482 | OR693714 | AgNosCd-1 male x *An. gambiae* Zanu female | Rep1 | F1 | ***Black****/****CFP+*** | Male | Drive/WT |
| ZN483 | OR693715 | AgNosCd-1 male x *An. gambiae* Zanu female | Rep1 | F1 | ***Black****/****CFP+*** | Male | Drive/WT |
| ZN484 | OR693716 | AgNosCd-1 male x *An. gambiae* Zanu female | Rep1 | F1 | ***Black****/****CFP+*** | Male | Drive/WT |
| ZN485 | OR693717 | AgNosCd-1 male x *An. gambiae* Zanu female | Rep1 | F1 | ***Black****/****CFP+*** | Male | Drive/WT |
| ZN486 | OR693718 | AgNosCd-1 male x *An. gambiae* Zanu female | Rep1 | F1 | ***Black****/****CFP+*** | Male | Drive/WT |
| ZN487 | OR693719 | AgNosCd-1 male x *An. gambiae* Zanu female | Rep1 | F1 | ***Black****/****CFP+*** | Male | Drive/WT |
| ZN488 | OR693720 | AgNosCd-1 male x *An. gambiae* Zanu female | Rep1 | F1 | ***Black****/****CFP+*** | Male | Drive/WT |
| ZN489 | OR693721 | AgNosCd-1 male x *An. gambiae* Zanu female | Rep1 | F1 | ***Black****/****CFP+*** | Male | Drive/WT |
| ZN490 | OR693722 | AgNosCd-1 male x *An. gambiae* Zanu female | Rep1 | F1 | ***Black****/****CFP+*** | Male | Drive/WT |
| ZN491 | OR693723 | AgNosCd-1 male x *An. gambiae* Zanu female | Rep1 | F1 | ***Black****/****CFP+*** | Female | Drive/WT |
| ZN492 | OR693724 | AgNosCd-1 male x *An. gambiae* Zanu female | Rep1 | F1 | ***Black****/****CFP+*** | Female | Drive/WT |
| ZN493 | OR693725 | AgNosCd-1 male x *An. gambiae* Zanu female | Rep1 | F1 | ***Black****/****CFP+*** | Female | Drive/WT |
| ZN494 | OR693726 | AgNosCd-1 male x *An. gambiae* Zanu female | Rep1 | F1 | ***Black****/****CFP+*** | Female | Drive/WT |
| ZN495 | OR693727 | AgNosCd-1 male x *An. gambiae* Zanu female | Rep1 | F1 | ***Black****/****CFP+*** | Female | Drive/WT |
| ZN496 | OR693728 | AgNosCd-1 male x *An. gambiae* Zanu female | Rep1 | F1 | ***Black****/****CFP+*** | Female | Drive/WT |
| ZN497 | OR693729 | AgNosCd-1 male x *An. gambiae* Zanu female | Rep1 | F1 | ***Black****/****CFP+*** | Female | Drive/WT |
| ZN498 | OR693730 | AgNosCd-1 male x *An. gambiae* Zanu female | Rep1 | F1 | ***Black****/****CFP+*** | Female | Drive/WT |
| ZN499 | OR693731 | AgNosCd-1 male x *An. gambiae* Zanu female | Rep1 | F1 | ***Black****/****CFP+*** | Female | Drive/WT |
| ZN500 | OR693732 | AgNosCd-1 male x *An. gambiae* Zanu female | Rep1 | F1 | ***Black****/****CFP+*** | Female | Drive/WT |
| ZN501 | OR693733 | AgNosCd-1 male x *An. gambiae* Zanu female | Rep2 | F1 | ***Black****/****CFP+*** | Male | Drive/WT |
| ZN502 | OR693734 | AgNosCd-1 male x *An. gambiae* Zanu female | Rep2 | F1 | ***Black****/****CFP+*** | Male | Drive/WT |
| ZN503 | OR693735 | AgNosCd-1 male x *An. gambiae* Zanu female | Rep2 | F1 | ***Black****/****CFP+*** | Male | Drive/WT |
| ZN504 | OR693736 | AgNosCd-1 male x *An. gambiae* Zanu female | Rep2 | F1 | ***Black****/****CFP+*** | Male | Drive/WT |
| ZN505 | OR693737 | AgNosCd-1 male x *An. gambiae* Zanu female | Rep2 | F1 | ***Black****/****CFP+*** | Male | Drive/WT |
| ZN506 | OR693738 | AgNosCd-1 male x *An. gambiae* Zanu female | Rep2 | F1 | ***Black****/****CFP+*** | Male | Drive/WT |
| ZN507 | OR693739 | AgNosCd-1 male x *An. gambiae* Zanu female | Rep2 | F1 | ***Black****/****CFP+*** | Male | Drive/WT |
| ZN508 | OR693740 | AgNosCd-1 male x *An. gambiae* Zanu female | Rep2 | F1 | ***Black****/****CFP+*** | Male | Drive/WT |
| ZN509 | OR693741 | AgNosCd-1 male x *An. gambiae* Zanu female | Rep2 | F1 | ***Black****/****CFP+*** | Male | Drive/WT |
| ZN510 | OR693742 | AgNosCd-1 male x *An. gambiae* Zanu female | Rep2 | F1 | ***Black****/****CFP+*** | Male | Drive/WT |
| ZN511 | OR693743 | AgNosCd-1 male x *An. gambiae* Zanu female | Rep2 | F1 | ***Black****/****CFP+*** | Female | Drive/WT |
| ZN512 | OR693744 | AgNosCd-1 male x *An. gambiae* Zanu female | Rep2 | F1 | ***Black****/****CFP+*** | Female | Drive/WT |
| ZN513 | OR693745 | AgNosCd-1 male x *An. gambiae* Zanu female | Rep2 | F1 | ***Black****/****CFP+*** | Female | Drive/WT |
| ZN514 | OR693746 | AgNosCd-1 male x *An. gambiae* Zanu female | Rep2 | F1 | ***Black****/****CFP+*** | Female | Drive/WT |
| ZN515 | OR693747 | AgNosCd-1 male x *An. gambiae* Zanu female | Rep2 | F1 | ***Black****/****CFP+*** | Female | Drive/WT |
| ZN516 | OR693748 | AgNosCd-1 male x *An. gambiae* Zanu female | Rep2 | F1 | ***Black****/****CFP+*** | Female | Drive/WT |
| ZN517 | OR693749 | AgNosCd-1 male x *An. gambiae* Zanu female | Rep2 | F1 | ***Black****/****CFP+*** | Female | Drive/WT |
| ZN518 | OR693750 | AgNosCd-1 male x *An. gambiae* Zanu female | Rep2 | F1 | ***Black****/****CFP+*** | Female | Drive/WT |
| ZN519 | OR693751 | AgNosCd-1 male x *An. gambiae* Zanu female | Rep2 | F1 | ***Black****/****CFP+*** | Female | Drive/WT |
| ZN520 | OR693752 | AgNosCd-1 male x *An. gambiae* Zanu female | Rep2 | F1 | ***Black****/****CFP+*** | Female | Drive/WT |
| ZN521 | OR693753 | AgNosCd-1 male x *An. gambiae* Zanu female | Rep3 | F1 | ***Black****/****CFP+*** | Male | Drive/WT |
| ZN522 | OR693754 | AgNosCd-1 male x *An. gambiae* Zanu female | Rep3 | F1 | ***Black****/****CFP+*** | Male | Drive/WT |
| ZN523 | OR693755 | AgNosCd-1 male x *An. gambiae* Zanu female | Rep3 | F1 | ***Black****/****CFP+*** | Male | Drive/WT |
| ZN524 | OR693756 | AgNosCd-1 male x *An. gambiae* Zanu female | Rep3 | F1 | ***Black****/****CFP+*** | Male | Drive/WT |
| ZN525 | OR693757 | AgNosCd-1 male x *An. gambiae* Zanu female | Rep3 | F1 | ***Black****/****CFP+*** | Male | Drive/WT |
| ZN526 | OR693758 | AgNosCd-1 male x *An. gambiae* Zanu female | Rep3 | F1 | ***Black****/****CFP+*** | Male | Drive/WT |
| ZN527 | OR693759 | AgNosCd-1 male x *An. gambiae* Zanu female | Rep3 | F1 | ***Black****/****CFP+*** | Male | Drive/WT |
| ZN528 | OR693760 | AgNosCd-1 male x *An. gambiae* Zanu female | Rep3 | F1 | ***Black****/****CFP+*** | Male | Drive/WT |
| ZN529 | OR693761 | AgNosCd-1 male x *An. gambiae* Zanu female | Rep3 | F1 | ***Black****/****CFP+*** | Male | Drive/WT |
| ZN530 | OR693762 | AgNosCd-1 male x *An. gambiae* Zanu female | Rep3 | F1 | ***Black****/****CFP+*** | Male | Drive/WT |
| ZN531 | OR693763 | AgNosCd-1 male x *An. gambiae* Zanu female | Rep3 | F1 | ***Black****/****CFP+*** | Female | Drive/WT |
| ZN532 | OR693764 | AgNosCd-1 male x *An. gambiae* Zanu female | Rep3 | F1 | ***Black****/****CFP+*** | Female | Drive/WT |
| ZN533 | OR693765 | AgNosCd-1 male x *An. gambiae* Zanu female | Rep3 | F1 | ***Black****/****CFP+*** | Female | Drive/WT |
| ZN534 | OR693766 | AgNosCd-1 male x *An. gambiae* Zanu female | Rep3 | F1 | ***Black****/****CFP+*** | Female | Drive/WT |
| ZN535 | OR693767 | AgNosCd-1 male x *An. gambiae* Zanu female | Rep3 | F1 | ***Black****/****CFP+*** | Female | Drive/WT |
| ZN536 | OR693768 | AgNosCd-1 male x *An. gambiae* Zanu female | Rep3 | F1 | ***Black****/****CFP+*** | Female | Drive/WT |
| ZN537 | OR693769 | AgNosCd-1 male x *An. gambiae* Zanu female | Rep3 | F1 | ***Black****/****CFP+*** | Female | Drive/WT |
| ZN538 | OR693770 | AgNosCd-1 male x *An. gambiae* Zanu female | Rep3 | F1 | ***Black****/****CFP+*** | Female | Drive/WT |
| ZN539 | OR693771 | AgNosCd-1 male x *An. gambiae* Zanu female | Rep3 | F1 | ***Black****/****CFP+*** | Female | Drive/WT |
| ZN540 | OR693772 | AgNosCd-1 male x *An. gambiae* Zanu female | Rep3 | F1 | ***Black****/****CFP+*** | Female | Drive/WT |
| ZN99 | OR693583 | AgNosCd-1 male/*An. gambiae* Zanu female hybrid intercross | Rep 2 | F2 | ***Black****/****CFP+*** | Male | Drive/WT |
| ZN100 | OR693584 | AgNosCd-1 male/*An. gambiae* Zanu female hybrid intercross | Rep 2 | F2 | ***Black****/****CFP+*** | Male | Drive/WT |
| ZN101 | OR693585 | AgNosCd-1 male/*An. gambiae* Zanu female hybrid intercross | Rep 2 | F2 | ***Black****/****CFP+*** | Female | Drive/WT |
| ZN141 | OR693625 | AgNosCd-1 male/*An. gambiae* Zanu female hybrid intercross | Rep 3 | F2 | ***Black****/****CFP+*** | Female | Drive/WT |
| ZN142 | OR693626 | AgNosCd-1 male/*An. gambiae* Zanu female hybrid intercross | Rep 3 | F2 | ***Black****/****CFP+*** | Male | Drive/WT |
| ZN143 | OR693627 | AgNosCd-1 male/*An. gambiae* Zanu female hybrid intercross | Rep 3 | F2 | ***Black****/****CFP+*** | Male | Drive/WT |
| ZN144 | OR693628 | AgNosCd-1 male/*An. gambiae* Zanu female hybrid intercross | Rep 3 | F2 | ***Black****/****CFP+*** | Female | Drive/WT |
| ZN145 | OR693629 | AgNosCd-1 male/*An. gambiae* Zanu female hybrid intercross | Rep 3 | F2 | ***Black****/****CFP+*** | Female | Drive/WT |
| ZN146 | OR693630 | AgNosCd-1 male/*An. gambiae* Zanu female hybrid intercross | Rep 3 | F2 | ***Black****/****CFP+*** | Male | Drive/WT |
| ZN105 | OR693592 | *An. gambiae* Zanu male/AgNosCd-1 female hybrid intercross | Rep 2 | F2 | ***Black****/****CFP+*** | Female | Drive/NHEJ |
| ZN106 | OR693593 | *An. gambiae* Zanu male/AgNosCd-1 female hybrid intercross | Rep 2 | F2 | ***Black****/****CFP+*** | Female | Drive/NHEJ |
| ZN107 | OR693594 | *An. gambiae* Zanu male/AgNosCd-1 female hybrid intercross | Rep 2 | F2 | ***Black****/****CFP+*** | Female | Drive/NHEJ |
| ZN108 | OR693595 | *An. gambiae* Zanu male/AgNosCd-1 female hybrid intercross | Rep 2 | F2 | ***Black****/****CFP+*** | Female | Drive/NHEJ |
| ZN109 | OR693596 | *An. gambiae* Zanu male/AgNosCd-1 female hybrid intercross | Rep 2 | F2 | ***Black****/****CFP+*** | Female | Drive/NHEJ |
| ZN110 | OR693597 | *An. gambiae* Zanu male/AgNosCd-1 female hybrid intercross | Rep 2 | F2 | ***Black****/****CFP+*** | Female | Drive/NHEJ |
| ZN111 | OR693598 | *An. gambiae* Zanu male/AgNosCd-1 female hybrid intercross | Rep 2 | F2 | ***Black****/****CFP+*** | Male | Drive/NHEJ |
| ZN112 | OR693599 | *An. gambiae* Zanu male/AgNosCd-1 female hybrid intercross | Rep 2 | F2 | ***Black****/****CFP+*** | Male | Drive/NHEJ |
| ZN113 | OR693600 | *An. gambiae* Zanu male/AgNosCd-1 female hybrid intercross | Rep 2 | F2 | ***Black****/****CFP+*** | Male | Drive/NHEJ |
| ZN114 | OR693601 | *An. gambiae* Zanu male/AgNosCd-1 female hybrid intercross | Rep 2 | F2 | ***Black****/****CFP+*** | Male | Drive/NHEJ |
| ZN133 | OR693617 | *An. gambiae* Zanu male/AgNosCd-1 female hybrid intercross | Rep 3 | F2 | ***Black****/****CFP+*** | Female | Drive/NHEJ |
| ZN134 | OR693618 | *An. gambiae* Zanu male/AgNosCd-1 female hybrid intercross | Rep 3 | F2 | ***Black****/****CFP+*** | Female | Drive/NHEJ |
| ZN135 | OR693619 | *An. gambiae* Zanu male/AgNosCd-1 female hybrid intercross | Rep 3 | F2 | ***Black****/****CFP+*** | Male | Drive/NHEJ |
| ZN136 | OR693620 | *An. gambiae* Zanu male/AgNosCd-1 female hybrid intercross | Rep 3 | F2 | ***Black****/****CFP+*** | Male | Drive/NHEJ |
| ZN137 | OR693621 | *An. gambiae* Zanu male/AgNosCd-1 female hybrid intercross | Rep 3 | F2 | ***Black****/****CFP+*** | Male | Drive/NHEJ |
| ZN138 | OR693622 | *An. gambiae* Zanu male/AgNosCd-1 female hybrid intercross | Rep 3 | F2 | ***Black****/****CFP+*** | Male | Drive/NHEJ |
| ZN139 | OR693623 | *An. gambiae* Zanu male/AgNosCd-1 female hybrid intercross | Rep 3 | F2 | ***Black****/****CFP+*** | Male | Drive/NHEJ |
| ZN140 | OR693624 | *An. gambiae* Zanu male/AgNosCd-1 female hybrid intercross | Rep 3 | F2 | ***Black****/****CFP+*** | Male | Drive/NHEJ |
| ZN146 | OR693631 | *An. gambiae* Zanu male/AgNosCd-1 female hybrid intercross | Rep 3 | F2 | ***Black****/****CFP+*** | Male | Drive/NHEJ |
| ZN147 | OR693632 | *An. gambiae* Zanu male/AgNosCd-1 female hybrid intercross | Rep 3 | F2 | ***Black****/****CFP+*** | Female | Drive/WT |
| ZN148 | OR693633 | *An. gambiae* Zanu male/AgNosCd-1 female hybrid intercross | Rep 3 | F2 | ***Black****/****CFP+*** | Female | Drive/WT |
| ZN20 | OR693506 | *An. gambiae* Zanu male/AgNosCd-1 female hybrid intercross | Rep 1 | F2 | ***Black****/CFP-* | Male | WT/NHEJ |
| ZN21 | OR693507 | *An. gambiae* Zanu male/AgNosCd-1 female hybrid intercross | Rep 1 | F2 | ***Black****/CFP-* | Male | WT/NHEJ |
| ZN85 | OR693569 | *An. gambiae* Zanu male/AgNosCd-1 female hybrid intercross | Rep 2 | F2 | ***Black****/CFP-* | Male | NHEJ/NHEJ |
|  | OR693570 |  |  |  |  |  |  |
| ZN86 | OR693571 | *An. gambiae* Zanu male/AgNosCd-1 female hybrid intercross | Rep 2 | F2 | ***Black****/CFP-* | Male | WT/NHEJ |
| ZN87 | OR693572 | *An. gambiae* Zanu male/AgNosCd-1 female hybrid intercross | Rep 2 | F2 | ***Black****/CFP-* | Male | WT/NHEJ |
| ZN88 | OR693573 | *An. gambiae* Zanu male/AgNosCd-1 female hybrid intercross | Rep 2 | F2 | ***Black****/CFP-* | Male | WT/NHEJ |
| ZN89 | OR693574 | *An. gambiae* Zanu male/AgNosCd-1 female hybrid intercross | Rep 2 | F2 | ***Black****/CFP-* | Male | WT/NHEJ |
| ZN17 | OR693501 | *An. gambiae* Zanu male/AgNosCd-1 female hybrid intercross | Rep 1 | F2 | *Cardinal/CFP-* | Male | NHEJ/NHEJ |
|  | OR693502 |  |  |  |  |  |  |
| ZN18 | OR693503 | *An. gambiae* Zanu male/AgNosCd-1 female hybrid intercross | Rep 1 | F2 | *Cardinal/CFP-* | Male | NHEJ/NHEJ |
|  | OR693504 |  |  |  |  |  |  |
| ZN19 | OR693505 | *An. gambiae* Zanu male/AgNosCd-1 female hybrid intercross | Rep 1 | F2 | *Cardinal/CFP-* | Female | NHEJ/NHEJ |
| ZN90 | OR693575 | *An. gambiae* Zanu male/AgNosCd-1 female hybrid intercross | Rep 2 | F2 | *Cardinal/CFP-* | Male | NHEJ/NHEJ |
| ZN91 | OR693576 | *An. gambiae* Zanu male/AgNosCd-1 female hybrid intercross | Rep 2 | F2 | *Cardinal/CFP-* | male | NHEJ/NHEJ |
| ZN92 | OR693577 | *An. gambiae* Zanu male/AgNosCd-1 female hybrid intercross | Rep 2 | F2 | *Cardinal/CFP-* | male | NHEJ/NHEJ |
| ZN93 | OR693578 | *An. gambiae* Zanu male/AgNosCd-1 female hybrid intercross | Rep 2 | F2 | *Cardinal/CFP-* | male | NHEJ/NHEJ |
| ZN95 | OR693579 | *An. gambiae* Zanu male/AgNosCd-1 female hybrid intercross | Rep 2 | F2 | *Cardinal/CFP-* | male | NHEJ/NHEJ |
| ZN96 | OR693580 | *An. gambiae* Zanu male/AgNosCd-1 female hybrid intercross | Rep 2 | F2 | *Cardinal/CFP-* | female | NHEJ/NHEJ |
| ZN97 | OR693581 | *An. gambiae* Zanu male/AgNosCd-1 female hybrid intercross | Rep 2 | F2 | *Cardinal/CFP-* | female | NHEJ/NHEJ |
| ZN98 | OR693582 | *An. gambiae* Zanu male/AgNosCd-1 female hybrid intercross | Rep 2 | F2 | *Cardinal/CFP-* | female | NHEJ/NHEJ |
| ZN102 | OR693586 | *An. gambiae* Zanu male/AgNosCd-1 female hybrid intercross | Rep 2 | F2 | *Cardinal/CFP-* | Male | NHEJ/NHEJ |
|  | OR693587 |  |  |  |  |  |  |
| ZN103 | OR693588 | *An. gambiae* Zanu male/AgNosCd-1 female hybrid intercross | Rep 2 | F2 | *Cardinal/CFP-* | Male | NHEJ/NHEJ |
|  | OR693589 |  |  |  |  |  |  |
| ZN104 | OR693590 | *An. gambiae* Zanu male/AgNosCd-1 female hybrid intercross | Rep 2 | F2 | *Cardinal/CFP-* | Female | NHEJ/NHEJ |
|  | OR693591 |  |  |  |  |  |  |
| ZN149 | OR693634 | *An. gambiae* Zanu male/AgNosCd-1 female hybrid intercross | Rep 3 | F2 | *Cardinal/CFP-* | | NHEJ/NHEJ |
| ZN150 | OR693635 | *An. gambiae* Zanu male/AgNosCd-1 female hybrid intercross | Rep 3 | F2 | *Cardinal/CFP-* | | NHEJ/NHEJ |
| ZN151 | OR693636 | *An. gambiae* Zanu male/AgNosCd-1 female hybrid intercross | Rep 3 | F2 | *Cardinal/CFP-* | | NHEJ/NHEJ |
| ZN301 |  | AgNosCd-1 male/*An. gambiae* Zanu female hybrid intercross | Rep1 | F2 | *Cardinal/CFP+* | Male | Drive/Drive |
| ZN302 |  | AgNosCd-1 male/*An. gambiae* Zanu female hybrid intercross | Rep1 | F2 | *Cardinal/CFP+* | Male | Drive/Drive |
| ZN303 |  | AgNosCd-1 male/*An. gambiae* Zanu female hybrid intercross | Rep1 | F2 | *Cardinal/CFP+* | Male | Drive/Drive |
| ZN304 |  | AgNosCd-1 male/*An. gambiae* Zanu female hybrid intercross | Rep1 | F2 | *Cardinal/CFP+* | Male | Drive/Drive |
| ZN305 |  | AgNosCd-1 male/*An. gambiae* Zanu female hybrid intercross | Rep1 | F2 | *Cardinal/CFP+* | Male | Drive/Drive |
| ZN306 |  | AgNosCd-1 male/*An. gambiae* Zanu female hybrid intercross | Rep1 | F2 | *Cardinal/CFP+* | Male | Drive/Drive |
| ZN307 |  | AgNosCd-1 male/*An. gambiae* Zanu female hybrid intercross | Rep1 | F2 | *Cardinal/CFP+* | Male | Drive/Drive |
| ZN308 |  | AgNosCd-1 male/*An. gambiae* Zanu female hybrid intercross | Rep1 | F2 | *Cardinal/CFP+* | Male | Drive/Drive |
| ZN309 |  | AgNosCd-1 male/*An. gambiae* Zanu female hybrid intercross | Rep1 | F2 | *Cardinal/CFP+* | Male | Drive/Drive |
| ZN310 |  | AgNosCd-1 male/*An. gambiae* Zanu female hybrid intercross | Rep1 | F2 | *Cardinal/CFP+* | Male | Drive/Drive |
| ZN311 |  | AgNosCd-1 male/*An. gambiae* Zanu female hybrid intercross | Rep1 | F2 | *Cardinal/CFP+* | Female | Drive/Drive |
| ZN312 |  | AgNosCd-1 male/*An. gambiae* Zanu female hybrid intercross | Rep1 | F2 | *Cardinal/CFP+* | Female | Drive/Drive |
| ZN313 |  | AgNosCd-1 male/*An. gambiae* Zanu female hybrid intercross | Rep1 | F2 | *Cardinal/CFP+* | Female | Drive/Drive |
| ZN314 |  | AgNosCd-1 male/*An. gambiae* Zanu female hybrid intercross | Rep1 | F2 | *Cardinal/CFP+* | Female | Drive/Drive |
| ZN315 |  | AgNosCd-1 male/*An. gambiae* Zanu female hybrid intercross | Rep1 | F2 | *Cardinal/CFP+* | Female | Drive/Drive |
| ZN316 |  | AgNosCd-1 male/*An. gambiae* Zanu female hybrid intercross | Rep1 | F2 | *Cardinal/CFP+* | Female | Drive/Drive |
| ZN317 |  | AgNosCd-1 male/*An. gambiae* Zanu female hybrid intercross | Rep1 | F2 | *Cardinal/CFP+* | Female | Drive/Drive |
| ZN318 |  | AgNosCd-1 male/*An. gambiae* Zanu female hybrid intercross | Rep1 | F2 | *Cardinal/CFP+* | Female | Drive/Drive |
| ZN319 |  | AgNosCd-1 male/*An. gambiae* Zanu female hybrid intercross | Rep1 | F2 | *Cardinal/CFP+* | Female | Drive/Drive |
| ZN320 |  | AgNosCd-1 male/*An. gambiae* Zanu female hybrid intercross | Rep1 | F2 | *Cardinal/CFP+* | Female | Drive/Drive |
| ZN321 |  | AgNosCd-1 male/*An. gambiae* Zanu female hybrid intercross | Rep2 | F2 | *Cardinal/CFP+* | Male | Drive/Drive |
| ZN322 |  | AgNosCd-1 male/*An. gambiae* Zanu female hybrid intercross | Rep2 | F2 | *Cardinal/CFP+* | Male | Drive/Drive |
| ZN323 |  | AgNosCd-1 male/*An. gambiae* Zanu female hybrid intercross | Rep2 | F2 | *Cardinal/CFP+* | Male | Drive/Drive |
| ZN324 |  | AgNosCd-1 male/*An. gambiae* Zanu female hybrid intercross | Rep2 | F2 | *Cardinal/CFP+* | Male | Drive/Drive |
| ZN325 |  | AgNosCd-1 male/*An. gambiae* Zanu female hybrid intercross | Rep2 | F2 | *Cardinal/CFP+* | Male | Drive/Drive |
| ZN326 |  | AgNosCd-1 male/*An. gambiae* Zanu female hybrid intercross | Rep2 | F2 | *Cardinal/CFP+* | Male | Drive/Drive |
| ZN327 |  | AgNosCd-1 male/*An. gambiae* Zanu female hybrid intercross | Rep2 | F2 | *Cardinal/CFP+* | Male | Drive/Drive |
| ZN328 |  | AgNosCd-1 male/*An. gambiae* Zanu female hybrid intercross | Rep2 | F2 | *Cardinal/CFP+* | Male | Drive/Drive |
| ZN329 |  | AgNosCd-1 male/*An. gambiae* Zanu female hybrid intercross | Rep2 | F2 | *Cardinal/CFP+* | Male | Drive/Drive |
| ZN330 |  | AgNosCd-1 male/*An. gambiae* Zanu female hybrid intercross | Rep2 | F2 | *Cardinal/CFP+* | Male | Drive/Drive |
| ZN331 |  | AgNosCd-1 male/*An. gambiae* Zanu female hybrid intercross | Rep2 | F2 | *Cardinal/CFP+* | Female | Drive/Drive |
| ZN332 |  | AgNosCd-1 male/*An. gambiae* Zanu female hybrid intercross | Rep2 | F2 | *Cardinal/CFP+* | Female | Drive/Drive |
| ZN333 |  | AgNosCd-1 male/*An. gambiae* Zanu female hybrid intercross | Rep2 | F2 | *Cardinal/CFP+* | Female | Drive/Drive |
| ZN334 |  | AgNosCd-1 male/*An. gambiae* Zanu female hybrid intercross | Rep2 | F2 | *Cardinal/CFP+* | Female | Drive/Drive |
| ZN335 |  | AgNosCd-1 male/*An. gambiae* Zanu female hybrid intercross | Rep2 | F2 | *Cardinal/CFP+* | Female | Drive/Drive |
| ZN336 |  | AgNosCd-1 male/*An. gambiae* Zanu female hybrid intercross | Rep2 | F2 | *Cardinal/CFP+* | Female | Drive/Drive |
| ZN337 |  | AgNosCd-1 male/*An. gambiae* Zanu female hybrid intercross | Rep2 | F2 | *Cardinal/CFP+* | Female | Drive/Drive |
| ZN338 |  | AgNosCd-1 male/*An. gambiae* Zanu female hybrid intercross | Rep2 | F2 | *Cardinal/CFP+* | Female | Drive/Drive |
| ZN339 |  | AgNosCd-1 male/*An. gambiae* Zanu female hybrid intercross | Rep2 | F2 | *Cardinal/CFP+* | Female | Drive/Drive |
| ZN340 |  | AgNosCd-1 male/*An. gambiae* Zanu female hybrid intercross | Rep2 | F2 | *Cardinal/CFP+* | Female | Drive/Drive |
| ZN341 |  | AgNosCd-1 male/*An. gambiae* Zanu female hybrid intercross | Rep3 | F2 | *Cardinal/CFP+* | Male | Drive/Drive |
| ZN342 |  | AgNosCd-1 male/*An. gambiae* Zanu female hybrid intercross | Rep3 | F2 | *Cardinal/CFP+* | Male | Drive/Drive |
| ZN343 |  | AgNosCd-1 male/*An. gambiae* Zanu female hybrid intercross | Rep3 | F2 | *Cardinal/CFP+* | Male | Drive/Drive |
| ZN344 |  | AgNosCd-1 male/*An. gambiae* Zanu female hybrid intercross | Rep3 | F2 | *Cardinal/CFP+* | Male | Drive/Drive |
| ZN345 |  | AgNosCd-1 male/*An. gambiae* Zanu female hybrid intercross | Rep3 | F2 | *Cardinal/CFP+* | Male | Drive/Drive |
| ZN346 |  | AgNosCd-1 male/*An. gambiae* Zanu female hybrid intercross | Rep3 | F2 | *Cardinal/CFP+* | Male | Drive/Drive |
| ZN347 |  | AgNosCd-1 male/*An. gambiae* Zanu female hybrid intercross | Rep3 | F2 | *Cardinal/CFP+* | Male | Drive/Drive |
| ZN348 |  | AgNosCd-1 male/*An. gambiae* Zanu female hybrid intercross | Rep3 | F2 | *Cardinal/CFP+* | Male | Drive/Drive |
| ZN349 |  | AgNosCd-1 male/*An. gambiae* Zanu female hybrid intercross | Rep3 | F2 | *Cardinal/CFP+* | Male | Drive/Drive |
| ZN350 |  | AgNosCd-1 male/*An. gambiae* Zanu female hybrid intercross | Rep3 | F2 | *Cardinal/CFP+* | Male | Drive/Drive |
| ZN351 |  | AgNosCd-1 male/*An. gambiae* Zanu female hybrid intercross | Rep3 | F2 | *Cardinal/CFP+* | Female | Drive/Drive |
| ZN352 |  | AgNosCd-1 male/*An. gambiae* Zanu female hybrid intercross | Rep3 | F2 | *Cardinal/CFP+* | Female | Drive/Drive |
| ZN353 |  | AgNosCd-1 male/*An. gambiae* Zanu female hybrid intercross | Rep3 | F2 | *Cardinal/CFP+* | Female | Drive/Drive |
| ZN354 |  | AgNosCd-1 male/*An. gambiae* Zanu female hybrid intercross | Rep3 | F2 | *Cardinal/CFP+* | Female | Drive/Drive |
| ZN355 |  | AgNosCd-1 male/*An. gambiae* Zanu female hybrid intercross | Rep3 | F2 | *Cardinal/CFP+* | Female | Drive/Drive |
| ZN356 |  | AgNosCd-1 male/*An. gambiae* Zanu female hybrid intercross | Rep3 | F2 | *Cardinal/CFP+* | Female | Drive/Drive |
| ZN357 |  | AgNosCd-1 male/*An. gambiae* Zanu female hybrid intercross | Rep3 | F2 | *Cardinal/CFP+* | Female | Drive/Drive |
| ZN358 |  | AgNosCd-1 male/*An. gambiae* Zanu female hybrid intercross | Rep3 | F2 | *Cardinal/CFP+* | Female | Drive/Drive |
| ZN359 |  | AgNosCd-1 male/*An. gambiae* Zanu female hybrid intercross | Rep3 | F2 | *Cardinal/CFP+* | Female | Drive/Drive |
| ZN360 |  | AgNosCd-1 male/*An. gambiae* Zanu female hybrid intercross | Rep3 | F2 | *Cardinal/CFP+* | Female | Drive/Drive |
| ZN361 |  | *An. gambiae* Zanu male/AgNosCd-1 female hybrid intercross | Rep1 | F2 | *Cardinal/CFP+* | Male | Drive/Drive |
| ZN362 |  | *An. gambiae* Zanu male/AgNosCd-1 female hybrid intercross | Rep1 | F2 | *Cardinal/CFP+* | Male | Drive/Drive |
| ZN363 |  | *An. gambiae* Zanu male/AgNosCd-1 female hybrid intercross | Rep1 | F2 | *Cardinal/CFP+* | Male | Drive/Drive |
| ZN364 | OR693637 | *An. gambiae* Zanu male/AgNosCd-1 female hybrid intercross | Rep1 | F2 | *Cardinal/CFP+* | Male | Drive/NHEJ |
| ZN365 |  | *An. gambiae* Zanu male/AgNosCd-1 female hybrid intercross | Rep1 | F2 | *Cardinal/CFP+* | Male | Drive/Drive |
| ZN366 | OR693638 | *An. gambiae* Zanu male/AgNosCd-1 female hybrid intercross | Rep1 | F2 | *Cardinal/CFP+* | Male | Drive/NHEJ |
| ZN367 |  | *An. gambiae* Zanu male/AgNosCd-1 female hybrid intercross | Rep1 | F2 | *Cardinal/CFP+* | Male | Drive/Drive |
| ZN368 | OR693639 | *An. gambiae* Zanu male/AgNosCd-1 female hybrid intercross | Rep1 | F2 | *Cardinal/CFP+* | Male | Drive/NHEJ |
| ZN369 | OR693640 | *An. gambiae* Zanu male/AgNosCd-1 female hybrid intercross | Rep1 | F2 | *Cardinal/CFP+* | Male | Drive/NHEJ |
| ZN370 | OR693641 | *An. gambiae* Zanu male/AgNosCd-1 female hybrid intercross | Rep1 | F2 | *Cardinal/CFP+* | Male | Drive/NHEJ |
| ZN371 | OR693642 | *An. gambiae* Zanu male/AgNosCd-1 female hybrid intercross | Rep1 | F2 | *Cardinal/CFP+* | Female | Drive/NHEJ |
| ZN372 |  | *An. gambiae* Zanu male/AgNosCd-1 female hybrid intercross | Rep1 | F2 | *Cardinal/CFP+* | Female | Drive/Drive |
| ZN373 |  | *An. gambiae* Zanu male/AgNosCd-1 female hybrid intercross | Rep1 | F2 | *Cardinal/CFP+* | Female | Drive/Drive |
| ZN374 |  | *An. gambiae* Zanu male/AgNosCd-1 female hybrid intercross | Rep1 | F2 | *Cardinal/CFP+* | Female | Drive/Drive |
| ZN375 |  | *An. gambiae* Zanu male/AgNosCd-1 female hybrid intercross | Rep1 | F2 | *Cardinal/CFP+* | Female | Drive/Drive |
| ZN376 |  | *An. gambiae* Zanu male/AgNosCd-1 female hybrid intercross | Rep1 | F2 | *Cardinal/CFP+* | Female | Drive/Drive |
| ZN377 |  | *An. gambiae* Zanu male/AgNosCd-1 female hybrid intercross | Rep1 | F2 | *Cardinal/CFP+* | Female | Drive/Drive |
| ZN378 |  | *An. gambiae* Zanu male/AgNosCd-1 female hybrid intercross | Rep1 | F2 | *Cardinal/CFP+* | Female | Drive/Drive |
| ZN379 |  | *An. gambiae* Zanu male/AgNosCd-1 female hybrid intercross | Rep1 | F2 | *Cardinal/CFP+* | Female | Drive/Drive |
| ZN380 |  | *An. gambiae* Zanu male/AgNosCd-1 female hybrid intercross | Rep1 | F2 | *Cardinal/CFP+* | Female | Drive/Drive |
| ZN381 |  | *An. gambiae* Zanu male/AgNosCd-1 female hybrid intercross | Rep2 | F2 | *Cardinal/CFP+* | Male | Drive/Drive |
| ZN382 |  | *An. gambiae* Zanu male/AgNosCd-1 female hybrid intercross | Rep2 | F2 | *Cardinal/CFP+* | Male | Drive/Drive |
| ZN383 |  | *An. gambiae* Zanu male/AgNosCd-1 female hybrid intercross | Rep2 | F2 | *Cardinal/CFP+* | Male | Drive/Drive |
| ZN384 |  | *An. gambiae* Zanu male/AgNosCd-1 female hybrid intercross | Rep2 | F2 | *Cardinal/CFP+* | Male | Drive/Drive |
| ZN385 |  | *An. gambiae* Zanu male/AgNosCd-1 female hybrid intercross | Rep2 | F2 | *Cardinal/CFP+* | Male | Drive/Drive |
| ZN386 |  | *An. gambiae* Zanu male/AgNosCd-1 female hybrid intercross | Rep2 | F2 | *Cardinal/CFP+* | Male | Drive/Drive |
| ZN387 |  | *An. gambiae* Zanu male/AgNosCd-1 female hybrid intercross | Rep2 | F2 | *Cardinal/CFP+* | Male | Drive/Drive |
| ZN388 |  | *An. gambiae* Zanu male/AgNosCd-1 female hybrid intercross | Rep2 | F2 | *Cardinal/CFP+* | Male | Drive/Drive |
| ZN389 |  | *An. gambiae* Zanu male/AgNosCd-1 female hybrid intercross | Rep2 | F2 | *Cardinal/CFP+* | Male | Drive/Drive |
| ZN390 |  | *An. gambiae* Zanu male/AgNosCd-1 female hybrid intercross | Rep2 | F2 | *Cardinal/CFP+* | Male | Drive/Drive |
| ZN391 |  | *An. gambiae* Zanu male/AgNosCd-1 female hybrid intercross | Rep2 | F2 | *Cardinal/CFP+* | Female | Drive/Drive |
| ZN392 | OR693643 | *An. gambiae* Zanu male/AgNosCd-1 female hybrid intercross | Rep2 | F2 | *Cardinal/CFP+* | Female | Drive/NHEJ |
| ZN393 | OR693644 | *An. gambiae* Zanu male/AgNosCd-1 female hybrid intercross | Rep2 | F2 | *Cardinal/CFP+* | Female | Drive/NHEJ |
| ZN394 |  | *An. gambiae* Zanu male/AgNosCd-1 female hybrid intercross | Rep2 | F2 | *Cardinal/CFP+* | Female | Drive/Drive |
| ZN395 |  | *An. gambiae* Zanu male/AgNosCd-1 female hybrid intercross | Rep2 | F2 | *Cardinal/CFP+* | Female | Drive/Drive |
| ZN396 | OR693645 | *An. gambiae* Zanu male/AgNosCd-1 female hybrid intercross | Rep2 | F2 | *Cardinal/CFP+* | Female | Drive/NHEJ |
| ZN397 |  | *An. gambiae* Zanu male/AgNosCd-1 female hybrid intercross | Rep2 | F2 | *Cardinal/CFP+* | Female | Drive/Drive |
| ZN398 |  | *An. gambiae* Zanu male/AgNosCd-1 female hybrid intercross | Rep2 | F2 | *Cardinal/CFP+* | Female | Drive/Drive |
| ZN399 |  | *An. gambiae* Zanu male/AgNosCd-1 female hybrid intercross | Rep2 | F2 | *Cardinal/CFP+* | Female | Drive/Drive |
| ZN400 | OR693646 | *An. gambiae* Zanu male/AgNosCd-1 female hybrid intercross | Rep2 | F2 | *Cardinal/CFP+* | Female | Drive/NHEJ |
| ZN401 |  | *An. gambiae* Zanu male/AgNosCd-1 female hybrid intercross | Rep3 | F2 | *Cardinal/CFP+* | Male | Drive/Drive |
| ZN402 |  | *An. gambiae* Zanu male/AgNosCd-1 female hybrid intercross | Rep3 | F2 | *Cardinal/CFP+* | Male | Drive/Drive |
| ZN403 |  | *An. gambiae* Zanu male/AgNosCd-1 female hybrid intercross | Rep3 | F2 | *Cardinal/CFP+* | Male | Drive/Drive |
| ZN404 |  | *An. gambiae* Zanu male/AgNosCd-1 female hybrid intercross | Rep3 | F2 | *Cardinal/CFP+* | Male | Drive/Drive |
| ZN405 |  | *An. gambiae* Zanu male/AgNosCd-1 female hybrid intercross | Rep3 | F2 | *Cardinal/CFP+* | Male | Drive/Drive |
| ZN406 |  | *An. gambiae* Zanu male/AgNosCd-1 female hybrid intercross | Rep3 | F2 | *Cardinal/CFP+* | Male | Drive/Drive |
| ZN407 | OR693647 | *An. gambiae* Zanu male/AgNosCd-1 female hybrid intercross | Rep3 | F2 | *Cardinal/CFP+* | Male | Drive/NHEJ |
| ZN408 | OR693648 | *An. gambiae* Zanu male/AgNosCd-1 female hybrid intercross | Rep3 | F2 | *Cardinal/CFP+* | Male | Drive/NHEJ |
| ZN409 |  | *An. gambiae* Zanu male/AgNosCd-1 female hybrid intercross | Rep3 | F2 | *Cardinal/CFP+* | Male | Drive/Drive |
| ZN410 |  | *An. gambiae* Zanu male/AgNosCd-1 female hybrid intercross | Rep3 | F2 | *Cardinal/CFP+* | Male | Drive/Drive |
| ZN411 | OR693649 | *An. gambiae* Zanu male/AgNosCd-1 female hybrid intercross | Rep3 | F2 | *Cardinal/CFP+* | Female | Drive/NHEJ |
| ZN412 |  | *An. gambiae* Zanu male/AgNosCd-1 female hybrid intercross | Rep3 | F2 | *Cardinal/CFP+* | Female | Drive/Drive |
| ZN413 |  | *An. gambiae* Zanu male/AgNosCd-1 female hybrid intercross | Rep3 | F2 | *Cardinal/CFP+* | Female | Drive/Drive |
| ZN414 | OR693650 | *An. gambiae* Zanu male/AgNosCd-1 female hybrid intercross | Rep3 | F2 | *Cardinal/CFP+* | Female | Drive/NHEJ |
| ZN415 |  | *An. gambiae* Zanu male/AgNosCd-1 female hybrid intercross | Rep3 | F2 | *Cardinal/CFP+* | Female | Drive/Drive |
| ZN416 |  | *An. gambiae* Zanu male/AgNosCd-1 female hybrid intercross | Rep3 | F2 | *Cardinal/CFP+* | Female | Drive/Drive |
| ZN417 |  | *An. gambiae* Zanu male/AgNosCd-1 female hybrid intercross | Rep3 | F2 | *Cardinal/CFP+* | Female | Drive/Drive |
| ZN418 |  | *An. gambiae* Zanu male/AgNosCd-1 female hybrid intercross | Rep3 | F2 | *Cardinal/CFP+* | Female | Drive/Drive |
| ZN419 |  | *An. gambiae* Zanu male/AgNosCd-1 female hybrid intercross | Rep3 | F2 | *Cardinal/CFP+* | Female | Drive/Drive |
| ZN420 |  | *An. gambiae* Zanu male/AgNosCd-1 female hybrid intercross | Rep3 | F2 | *Cardinal/CFP+* | Female | Drive/Drive |
| KSM85 | OR693773 | *An. gambiae* Kisumu male x AgNosCd-1 female | Rep 1 | F1 | *Cardinal/CFP+* | Female | Drive/NHEJ |
|  | OR693774 |  |  |  |  |  |  |
| KSM86 | OR693775 | *An. gambiae* Kisumu male x AgNosCd-1 female | Rep 1 | F1 | *Cardinal/CFP+* | Female | Drive/NHEJ |
| KSM87 | OR693776 | *An. gambiae* Kisumu male x AgNosCd-1 female | Rep 1 | F1 | *Cardinal/CFP+* | Female | Drive/NHEJ |
|  | OR693777 |  |  |  |  |  |  |
| KSM88 | OR693778 | *An. gambiae* Kisumu male x AgNosCd-1 female | Rep 1 | F1 | *Cardinal/CFP+* | Female | Drive/NHEJ |
|  | OR693779 |  |  |  |  |  |  |
| KSM89 | OR693780 | *An. gambiae* Kisumu male x AgNosCd-1 female | Rep 1 | F1 | *Cardinal/CFP+* | Female | Drive/NHEJ |
| KSM90 | OR693781 | *An. gambiae* Kisumu male x AgNosCd-1 female | Rep 1 | F1 | *Cardinal/CFP+* | Female | Drive/NHEJ |
|  | OR693782 |  |  |  |  |  |  |
| KSM91 | OR693783 | *An. gambiae* Kisumu male x AgNosCd-1 female | Rep 1 | F1 | *Cardinal/CFP+* | Female | Drive/NHEJ |
|  | OR693784 |  |  |  |  |  |  |
| KSM92 | OR693785 | *An. gambiae* Kisumu male x AgNosCd-1 female | Rep 1 | F1 | *Cardinal/CFP+* | Female | Drive/NHEJ |
|  | OR693786 |  |  |  |  |  |  |
| KSM93 | OR693787 | *An. gambiae* Kisumu male x AgNosCd-1 female | Rep 1 | F1 | *Cardinal/CFP+* | Female | Drive/NHEJ |
|  | OR693788 |  |  |  |  |  |  |
| KSM94 | OR693789 | *An. gambiae* Kisumu male x AgNosCd-1 female | Rep 1 | F1 | *Cardinal/CFP+* | Female | Drive/NHEJ |
|  | OR693790 |  |  |  |  |  |  |
| KSM95 | OR693791 | *An. gambiae* Kisumu male x AgNosCd-1 female | Rep 1 | F1 | *Cardinal/CFP+* | male | Drive/NHEJ |
| KSM96 | OR693792 | *An. gambiae* Kisumu male x AgNosCd-1 female | Rep 1 | F1 | *Cardinal/CFP+* | male | Drive/NHEJ |
|  | OR693793 |  |  |  |  |  |  |
| KSM97 | OR693794 | *An. gambiae* Kisumu male x AgNosCd-1 female | Rep 1 | F1 | *Cardinal/CFP+* | male | Drive/NHEJ |
|  | OR693795 |  |  |  |  |  |  |
| KSM98 | OR693796 | *An. gambiae* Kisumu male x AgNosCd-1 female | Rep 1 | F1 | *Cardinal/CFP+* | male | Drive/NHEJ |
|  | OR693797 |  |  |  |  |  |  |
| KSM99 | OR693798 | *An. gambiae* Kisumu male x AgNosCd-1 female | Rep 1 | F1 | *Cardinal/CFP+* | male | Drive/NHEJ |
|  | OR693799 |  |  |  |  |  |  |
| KSM100 | OR693800 | *An. gambiae* Kisumu male x AgNosCd-1 female | Rep 1 | F1 | *Cardinal/CFP+* | male | Drive/NHEJ |
|  | OR693801 |  |  |  |  |  |  |
| KSM101 | OR693802 | *An. gambiae* Kisumu male x AgNosCd-1 female | Rep 1 | F1 | *Cardinal/CFP+* | male | Drive/NHEJ |
|  | OR693803 |  |  |  |  |  |  |
| KSM102 | OR693804 | *An. gambiae* Kisumu male x AgNosCd-1 female | Rep 1 | F1 | *Cardinal/CFP+* | male | Drive/NHEJ |
|  | OR693805 |  |  |  |  |  |  |
| KSM103 | OR693806 | *An. gambiae* Kisumu male x AgNosCd-1 female | Rep 1 | F1 | *Cardinal/CFP+* | male | Drive/NHEJ |
|  | OR693807 |  |  |  |  |  |  |
| KSM104 | OR693808 | *An. gambiae* Kisumu male x AgNosCd-1 female | Rep 1 | F1 | *Cardinal/CFP+* | male | Drive/NHEJ |
|  | OR693809 |  |  |  |  |  |  |
| KSM105 | OR693810 | *An. gambiae* Kisumu male x AgNosCd-1 female | Rep 1 | F1 | *Cardinal/CFP+* | male | Drive/NHEJ |
|  | OR693811 |  |  |  |  |  |  |
| KSM118 | OR693834 | *An. gambiae* Kisumu male x AgNosCd-1 female | Rep 2 | F1 | *Cardinal/CFP+* | Female | Drive/NHEJ |
|  | OR693835 |  |  |  |  |  |  |
| KSM119 | OR693836 | *An. gambiae* Kisumu male x AgNosCd-1 female | Rep 2 | F1 | *Cardinal/CFP+* | Female | Drive/NHEJ |
|  | OR693837 |  |  |  |  |  |  |
| KSM120 | OR693838 | *An. gambiae* Kisumu male x AgNosCd-1 female | Rep 2 | F1 | *Cardinal/CFP+* | Female | Drive/NHEJ |
|  | OR693839 |  |  |  |  |  |  |
| KSM121 | OR693840 | *An. gambiae* Kisumu male x AgNosCd-1 female | Rep 2 | F1 | *Cardinal/CFP+* | Female | Drive/NHEJ |
|  | OR693841 |  |  |  |  |  |  |
| KSM122 | OR693842 | *An. gambiae* Kisumu male x AgNosCd-1 female | Rep 2 | F1 | *Cardinal/CFP+* | male | Drive/NHEJ |
|  | OR693843 |  |  |  |  |  |  |
| KSM123 | OR693844 | *An. gambiae* Kisumu male x AgNosCd-1 female | Rep 2 | F1 | *Cardinal/CFP+* | male | Drive/NHEJ |
|  | OR693845 |  |  |  |  |  |  |
| KSM124 | OR693846 | *An. gambiae* Kisumu male x AgNosCd-1 female | Rep 2 | F1 | *Cardinal/CFP+* | male | Drive/NHEJ |
|  | OR693847 |  |  |  |  |  |  |
| KSM125 | OR693848 | *An. gambiae* Kisumu male x AgNosCd-1 female | Rep 2 | F1 | *Cardinal/CFP+* | male | Drive/NHEJ |
|  | OR693849 |  |  |  |  |  |  |
| KSM170 | OR693870 | *An. gambiae* Kisumu male x AgNosCd-1 female | Rep 3 | F1 | *Cardinal/CFP+* | Female | Drive/NHEJ |
| KSM171 | OR693871 | *An. gambiae* Kisumu male x AgNosCd-1 female | Rep 3 | F1 | *Cardinal/CFP+* | Female | Drive/NHEJ |
|  | OR693872 |  |  |  |  |  |  |
| KSM172 | OR693873 | *An. gambiae* Kisumu male x AgNosCd-1 female | Rep 3 | F1 | *Cardinal/CFP+* | Female | Drive/NHEJ |
| KSM173 | OR693874 | *An. gambiae* Kisumu male x AgNosCd-1 female | Rep 3 | F1 | *Cardinal/CFP+* | Female | Drive/NHEJ |
|  | OR693875 |  |  |  |  |  |  |
| KSM174 | OR693876 | *An. gambiae* Kisumu male x AgNosCd-1 female | Rep 3 | F1 | *Cardinal/CFP+* | male | Drive/NHEJ |
|  | OR693877 |  |  |  |  |  |  |
| KSM175 | OR693878 | *An. gambiae* Kisumu male x AgNosCd-1 female | Rep 3 | F1 | *Cardinal/CFP+* | male | Drive/NHEJ |
|  | OR693879 |  |  |  |  |  |  |
| KSM176 | OR693880 | *An. gambiae* Kisumu male x AgNosCd-1 female | Rep 3 | F1 | *Cardinal/CFP+* | male | Drive/NHEJ |
|  | OR693881 |  |  |  |  |  |  |
| KSM177 | OR693882 | *An. gambiae* Kisumu male x AgNosCd-1 female | Rep 3 | F1 | *Cardinal/CFP+* | male | Drive/NHEJ |
|  | OR693883 |  |  |  |  |  |  |
| KSM421 | OR693912 | *An. gambiae* Kisumu male x AgNosCd-1 female | Rep1 | F1 | ***Black****/****CFP+*** | Male | Drive/WT |
| KSM422 | OR693913 | *An. gambiae* Kisumu male x AgNosCd-1 female | Rep1 | F1 | ***Black****/****CFP+*** | Male | Drive/WT |
| KSM423 | OR693914 | *An. gambiae* Kisumu male x AgNosCd-1 female | Rep1 | F1 | ***Black****/****CFP+*** | Male | Drive/WT |
| KSM424 | OR693915 | *An. gambiae* Kisumu male x AgNosCd-1 female | Rep1 | F1 | ***Black****/****CFP+*** | Male | Drive/WT |
| KSM425 | OR693916 | *An. gambiae* Kisumu male x AgNosCd-1 female | Rep1 | F1 | ***Black****/****CFP+*** | Male | Drive/WT |
| KSM426 | OR693917 | *An. gambiae* Kisumu male x AgNosCd-1 female | Rep1 | F1 | ***Black****/****CFP+*** | Male | Drive/WT |
| KSM427 | OR693918 | *An. gambiae* Kisumu male x AgNosCd-1 female | Rep1 | F1 | ***Black****/****CFP+*** | Male | Drive/WT |
| KSM428 | OR693919 | *An. gambiae* Kisumu male x AgNosCd-1 female | Rep1 | F1 | ***Black****/****CFP+*** | Male | Drive/WT |
| KSM429 | OR693920 | *An. gambiae* Kisumu male x AgNosCd-1 female | Rep1 | F1 | ***Black****/****CFP+*** | Male | Drive/WT |
| KSM430 | OR693921 | *An. gambiae* Kisumu male x AgNosCd-1 female | Rep1 | F1 | ***Black****/****CFP+*** | Male | Drive/WT |
| KSM431 | OR693922 | *An. gambiae* Kisumu male x AgNosCd-1 female | Rep1 | F1 | ***Black****/****CFP+*** | Female | Drive/WT |
| KSM432 | OR693923 | *An. gambiae* Kisumu male x AgNosCd-1 female | Rep1 | F1 | ***Black****/****CFP+*** | Female | Drive/WT |
| KSM433 | OR693924 | *An. gambiae* Kisumu male x AgNosCd-1 female | Rep1 | F1 | ***Black****/****CFP+*** | Female | Drive/WT |
| KSM434 | OR693925 | *An. gambiae* Kisumu male x AgNosCd-1 female | Rep1 | F1 | ***Black****/****CFP+*** | Female | Drive/WT |
| KSM435 | OR693926 | *An. gambiae* Kisumu male x AgNosCd-1 female | Rep1 | F1 | ***Black****/****CFP+*** | Female | Drive/WT |
| KSM436 | OR693927 | *An. gambiae* Kisumu male x AgNosCd-1 female | Rep1 | F1 | ***Black****/****CFP+*** | Female | Drive/WT |
| KSM437 | OR693928 | *An. gambiae* Kisumu male x AgNosCd-1 female | Rep1 | F1 | ***Black****/****CFP+*** | Female | Drive/WT |
| KSM438 | OR693929 | *An. gambiae* Kisumu male x AgNosCd-1 female | Rep1 | F1 | ***Black****/****CFP+*** | Female | Drive/WT |
| KSM439 | OR693930 | *An. gambiae* Kisumu male x AgNosCd-1 female | Rep1 | F1 | ***Black****/****CFP+*** | Female | Drive/WT |
| KSM440 | OR693931 | *An. gambiae* Kisumu male x AgNosCd-1 female | Rep1 | F1 | ***Black****/****CFP+*** | Female | Drive/WT |
| KSM441 | OR693932 | *An. gambiae* Kisumu male x AgNosCd-1 female | Rep2 | F1 | ***Black****/****CFP+*** | Male | Drive/WT |
| KSM442 | OR693933 | *An. gambiae* Kisumu male x AgNosCd-1 female | Rep2 | F1 | ***Black****/****CFP+*** | Male | Drive/WT |
| KSM443 | OR693934 | *An. gambiae* Kisumu male x AgNosCd-1 female | Rep2 | F1 | ***Black****/****CFP+*** | Male | Drive/WT |
| KSM444 | OR693935 | *An. gambiae* Kisumu male x AgNosCd-1 female | Rep2 | F1 | ***Black****/****CFP+*** | Male | Drive/WT |
| KSM445 | OR693936 | *An. gambiae* Kisumu male x AgNosCd-1 female | Rep2 | F1 | ***Black****/****CFP+*** | Male | Drive/WT |
| KSM446 | OR693937 | *An. gambiae* Kisumu male x AgNosCd-1 female | Rep2 | F1 | ***Black****/****CFP+*** | Male | Drive/WT |
| KSM447 | OR693938 | *An. gambiae* Kisumu male x AgNosCd-1 female | Rep2 | F1 | ***Black****/****CFP+*** | Male | Drive/WT |
| KSM448 | OR693939 | *An. gambiae* Kisumu male x AgNosCd-1 female | Rep2 | F1 | ***Black****/****CFP+*** | Male | Drive/WT |
| KSM449 | OR693940 | *An. gambiae* Kisumu male x AgNosCd-1 female | Rep2 | F1 | ***Black****/****CFP+*** | Male | Drive/WT |
| KSM450 | OR693941 | *An. gambiae* Kisumu male x AgNosCd-1 female | Rep2 | F1 | ***Black****/****CFP+*** | Male | Drive/WT |
| KSM451 | OR693942 | *An. gambiae* Kisumu male x AgNosCd-1 female | Rep2 | F1 | ***Black****/****CFP+*** | Female | Drive/WT |
| KSM452 | OR693943 | *An. gambiae* Kisumu male x AgNosCd-1 female | Rep2 | F1 | ***Black****/****CFP+*** | Female | Drive/WT |
| KSM453 | OR693944 | *An. gambiae* Kisumu male x AgNosCd-1 female | Rep2 | F1 | ***Black****/****CFP+*** | Female | Drive/WT |
| KSM454 | OR693945 | *An. gambiae* Kisumu male x AgNosCd-1 female | Rep2 | F1 | ***Black****/****CFP+*** | Female | Drive/WT |
| KSM455 | OR693946 | *An. gambiae* Kisumu male x AgNosCd-1 female | Rep2 | F1 | ***Black****/****CFP+*** | Female | Drive/WT |
| KSM456 | OR693947 | *An. gambiae* Kisumu male x AgNosCd-1 female | Rep2 | F1 | ***Black****/****CFP+*** | Female | Drive/WT |
| KSM457 | OR693948 | *An. gambiae* Kisumu male x AgNosCd-1 female | Rep2 | F1 | ***Black****/****CFP+*** | Female | Drive/WT |
| KSM458 | OR693949 | *An. gambiae* Kisumu male x AgNosCd-1 female | Rep2 | F1 | ***Black****/****CFP+*** | Female | Drive/WT |
| KSM459 | OR693950 | *An. gambiae* Kisumu male x AgNosCd-1 female | Rep2 | F1 | ***Black****/****CFP+*** | Female | Drive/WT |
| KSM460 | OR693951 | *An. gambiae* Kisumu male x AgNosCd-1 female | Rep2 | F1 | ***Black****/****CFP+*** | Female | Drive/WT |
| KSM461 | OR693952 | *An. gambiae* Kisumu male x AgNosCd-1 female | Rep3 | F1 | ***Black****/****CFP+*** | Male | Drive/WT |
| KSM462 | OR693953 | *An. gambiae* Kisumu male x AgNosCd-1 female | Rep3 | F1 | ***Black****/****CFP+*** | Male | Drive/NHEJ |
| KSM463 | OR693954 | *An. gambiae* Kisumu male x AgNosCd-1 female | Rep3 | F1 | ***Black****/****CFP+*** | Male | Drive/WT |
| KSM464 | OR693955 | *An. gambiae* Kisumu male x AgNosCd-1 female | Rep3 | F1 | ***Black****/****CFP+*** | Male | Drive/WT |
| KSM465 | OR693956 | *An. gambiae* Kisumu male x AgNosCd-1 female | Rep3 | F1 | ***Black****/****CFP+*** | Male | Drive/WT |
| KSM466 | OR693957 | *An. gambiae* Kisumu male x AgNosCd-1 female | Rep3 | F1 | ***Black****/****CFP+*** | Male | Drive/WT |
| KSM467 | OR693958 | *An. gambiae* Kisumu male x AgNosCd-1 female | Rep3 | F1 | ***Black****/****CFP+*** | Male | Drive/WT |
| KSM468 | OR693959 | *An. gambiae* Kisumu male x AgNosCd-1 female | Rep3 | F1 | ***Black****/****CFP+*** | Male | Drive/WT |
| KSM469 | OR693960 | *An. gambiae* Kisumu male x AgNosCd-1 female | Rep3 | F1 | ***Black****/****CFP+*** | Male | Drive/WT |
| KSM470 | OR693961 | *An. gambiae* Kisumu male x AgNosCd-1 female | Rep3 | F1 | ***Black****/****CFP+*** | Male | Drive/WT |
| KSM471 | OR693962 | *An. gambiae* Kisumu male x AgNosCd-1 female | Rep3 | F1 | ***Black****/****CFP+*** | Female | Drive/WT |
| KSM472 | OR693963 | *An. gambiae* Kisumu male x AgNosCd-1 female | Rep3 | F1 | ***Black****/****CFP+*** | Female | Drive/WT |
| KSM473 | OR693964 | *An. gambiae* Kisumu male x AgNosCd-1 female | Rep3 | F1 | ***Black****/****CFP+*** | Female | Drive/WT |
| KSM474 | OR693965 | *An. gambiae* Kisumu male x AgNosCd-1 female | Rep3 | F1 | ***Black****/****CFP+*** | Female | Drive/NHEJ |
|  | OR693966 |  |  |  |  |  |  |
| KSM475 | OR693967 | *An. gambiae* Kisumu male x AgNosCd-1 female | Rep3 | F1 | ***Black****/****CFP+*** | Female | Drive/WT |
| KSM476 | OR693968 | *An. gambiae* Kisumu male x AgNosCd-1 female | Rep3 | F1 | ***Black****/****CFP+*** | Female | Drive/WT |
| KSM477 | OR693969 | *An. gambiae* Kisumu male x AgNosCd-1 female | Rep3 | F1 | ***Black****/****CFP+*** | Female | Drive/NHEJ |
| KSM478 | OR693970 | *An. gambiae* Kisumu male x AgNosCd-1 female | Rep3 | F1 | ***Black****/****CFP+*** | Female | Drive/WT |
| KSM479 | OR693971 | *An. gambiae* Kisumu male x AgNosCd-1 female | Rep3 | F1 | ***Black****/****CFP+*** | Female | Drive/WT |
| KSM480 | OR693972 | *An. gambiae* Kisumu male x AgNosCd-1 female | Rep3 | F1 | ***Black****/****CFP+*** | Female | Drive/WT |
| KSM481 | OR693973 | AgNosCd-1 male x *An. gambiae* Kisumu female | Rep1 | F1 | ***Black****/****CFP+*** | Male | Drive/WT |
| KSM482 | OR693974 | AgNosCd-1 male x *An. gambiae* Kisumu female | Rep1 | F1 | ***Black****/****CFP+*** | Male | Drive/WT |
| KSM483 | OR693975 | AgNosCd-1 male x *An. gambiae* Kisumu female | Rep1 | F1 | ***Black****/****CFP+*** | Male | Drive/WT |
| KSM484 | OR693976 | AgNosCd-1 male x *An. gambiae* Kisumu female | Rep1 | F1 | ***Black****/****CFP+*** | Male | Drive/WT |
| KSM485 | OR693977 | AgNosCd-1 male x *An. gambiae* Kisumu female | Rep1 | F1 | ***Black****/****CFP+*** | Male | Drive/WT |
| KSM486 | OR693978 | AgNosCd-1 male x *An. gambiae* Kisumu female | Rep1 | F1 | ***Black****/****CFP+*** | Male | Drive/WT |
| KSM487 | OR693979 | AgNosCd-1 male x *An. gambiae* Kisumu female | Rep1 | F1 | ***Black****/****CFP+*** | Male | Drive/WT |
| KSM488 | OR693980 | AgNosCd-1 male x *An. gambiae* Kisumu female | Rep1 | F1 | ***Black****/****CFP+*** | Male | Drive/WT |
| KSM489 | OR693981 | AgNosCd-1 male x *An. gambiae* Kisumu female | Rep1 | F1 | ***Black****/****CFP+*** | Male | Drive/WT |
| KSM490 | OR693982 | AgNosCd-1 male x *An. gambiae* Kisumu female | Rep1 | F1 | ***Black****/****CFP+*** | Male | Drive/WT |
| KSM491 | OR693983 | AgNosCd-1 male x *An. gambiae* Kisumu female | Rep1 | F1 | ***Black****/****CFP+*** | Female | Drive/WT |
| KSM492 | OR693984 | AgNosCd-1 male x *An. gambiae* Kisumu female | Rep1 | F1 | ***Black****/****CFP+*** | Female | Drive/WT |
| KSM493 | OR693985 | AgNosCd-1 male x *An. gambiae* Kisumu female | Rep1 | F1 | ***Black****/****CFP+*** | Female | Drive/WT |
| KSM494 | OR693986 | AgNosCd-1 male x *An. gambiae* Kisumu female | Rep1 | F1 | ***Black****/****CFP+*** | Female | Drive/WT |
| KSM495 | OR693987 | AgNosCd-1 male x *An. gambiae* Kisumu female | Rep1 | F1 | ***Black****/****CFP+*** | Female | Drive/WT |
| KSM496 | OR693988 | AgNosCd-1 male x *An. gambiae* Kisumu female | Rep1 | F1 | ***Black****/****CFP+*** | Female | Drive/WT |
| KSM497 | OR693989 | AgNosCd-1 male x *An. gambiae* Kisumu female | Rep1 | F1 | ***Black****/****CFP+*** | Female | Drive/WT |
| KSM498 | OR693990 | AgNosCd-1 male x *An. gambiae* Kisumu female | Rep1 | F1 | ***Black****/****CFP+*** | Female | Drive/WT |
| KSM499 | OR693991 | AgNosCd-1 male x *An. gambiae* Kisumu female | Rep1 | F1 | ***Black****/****CFP+*** | Female | Drive/WT |
| KSM500 | OR693992 | AgNosCd-1 male x *An. gambiae* Kisumu female | Rep1 | F1 | ***Black****/****CFP+*** | Female | Drive/WT |
| KSM501 | OR693993 | AgNosCd-1 male x *An. gambiae* Kisumu female | Rep2 | F1 | ***Black****/****CFP+*** | Male | Drive/WT |
| KSM502 | OR693994 | AgNosCd-1 male x *An. gambiae* Kisumu female | Rep2 | F1 | ***Black****/****CFP+*** | Male | Drive/WT |
| KSM503 | OR693995 | AgNosCd-1 male x *An. gambiae* Kisumu female | Rep2 | F1 | ***Black****/****CFP+*** | Male | Drive/WT |
| KSM504 | OR693996 | AgNosCd-1 male x *An. gambiae* Kisumu female | Rep2 | F1 | ***Black****/****CFP+*** | Male | Drive/WT |
| KSM505 | OR693997 | AgNosCd-1 male x *An. gambiae* Kisumu female | Rep2 | F1 | ***Black****/****CFP+*** | Male | Drive/WT |
| KSM506 | OR693998 | AgNosCd-1 male x *An. gambiae* Kisumu female | Rep2 | F1 | ***Black****/****CFP+*** | Male | Drive/WT |
| KSM507 | OR693999 | AgNosCd-1 male x *An. gambiae* Kisumu female | Rep2 | F1 | ***Black****/****CFP+*** | Male | Drive/WT |
| KSM508 | OR694000 | AgNosCd-1 male x *An. gambiae* Kisumu female | Rep2 | F1 | ***Black****/****CFP+*** | Male | Drive/WT |
| KSM509 | OR694001 | AgNosCd-1 male x *An. gambiae* Kisumu female | Rep2 | F1 | ***Black****/****CFP+*** | Male | Drive/WT |
| KSM510 | OR694002 | AgNosCd-1 male x *An. gambiae* Kisumu female | Rep2 | F1 | ***Black****/****CFP+*** | Male | Drive/WT |
| KSM511 | OR694003 | AgNosCd-1 male x *An. gambiae* Kisumu female | Rep2 | F1 | ***Black****/****CFP+*** | Female | Drive/WT |
| KSM512 | OR694004 | AgNosCd-1 male x *An. gambiae* Kisumu female | Rep2 | F1 | ***Black****/****CFP+*** | Female | Drive/WT |
| KSM513 | OR694005 | AgNosCd-1 male x *An. gambiae* Kisumu female | Rep2 | F1 | ***Black****/****CFP+*** | Female | Drive/WT |
| KSM514 | OR694006 | AgNosCd-1 male x *An. gambiae* Kisumu female | Rep2 | F1 | ***Black****/****CFP+*** | Female | Drive/WT |
| KSM515 | OR694007 | AgNosCd-1 male x *An. gambiae* Kisumu female | Rep2 | F1 | ***Black****/****CFP+*** | Female | Drive/WT |
| KSM516 | OR694008 | AgNosCd-1 male x *An. gambiae* Kisumu female | Rep2 | F1 | ***Black****/****CFP+*** | Female | Drive/WT |
| KSM517 | OR694009 | AgNosCd-1 male x *An. gambiae* Kisumu female | Rep2 | F1 | ***Black****/****CFP+*** | Female | Drive/WT |
| KSM518 | OR694010 | AgNosCd-1 male x *An. gambiae* Kisumu female | Rep2 | F1 | ***Black****/****CFP+*** | Female | Drive/WT |
| KSM519 | OR694011 | AgNosCd-1 male x *An. gambiae* Kisumu female | Rep2 | F1 | ***Black****/****CFP+*** | Female | Drive/WT |
| KSM520 | OR694012 | AgNosCd-1 male x *An. gambiae* Kisumu female | Rep2 | F1 | ***Black****/****CFP+*** | Female | Drive/WT |
| KSM521 | OR694013 | AgNosCd-1 male x *An. gambiae* Kisumu female | Rep3 | F1 | ***Black****/****CFP+*** | Male | Drive/WT |
| KSM522 | OR694014 | AgNosCd-1 male x *An. gambiae* Kisumu female | Rep3 | F1 | ***Black****/****CFP+*** | Male | Drive/WT |
| KSM523 | OR694015 | AgNosCd-1 male x *An. gambiae* Kisumu female | Rep3 | F1 | ***Black****/****CFP+*** | Male | Drive/WT |
| KSM524 | OR694016 | AgNosCd-1 male x *An. gambiae* Kisumu female | Rep3 | F1 | ***Black****/****CFP+*** | Male | Drive/WT |
| KSM525 | OR694017 | AgNosCd-1 male x *An. gambiae* Kisumu female | Rep3 | F1 | ***Black****/****CFP+*** | Male | Drive/WT |
| KSM526 | OR694018 | AgNosCd-1 male x *An. gambiae* Kisumu female | Rep3 | F1 | ***Black****/****CFP+*** | Male | Drive/WT |
| KSM527 | OR694019 | AgNosCd-1 male x *An. gambiae* Kisumu female | Rep3 | F1 | ***Black****/****CFP+*** | Male | Drive/WT |
| KSM528 | OR694020 | AgNosCd-1 male x *An. gambiae* Kisumu female | Rep3 | F1 | ***Black****/****CFP+*** | Male | Drive/WT |
| KSM529 | OR694021 | AgNosCd-1 male x *An. gambiae* Kisumu female | Rep3 | F1 | ***Black****/****CFP+*** | Male | Drive/WT |
| KSM530 | OR694022 | AgNosCd-1 male x *An. gambiae* Kisumu female | Rep3 | F1 | ***Black****/****CFP+*** | Male | Drive/WT |
| KSM531 | OR694023 | AgNosCd-1 male x *An. gambiae* Kisumu female | Rep3 | F1 | ***Black****/****CFP+*** | Female | Drive/WT |
| KSM532 | OR694024 | AgNosCd-1 male x *An. gambiae* Kisumu female | Rep3 | F1 | ***Black****/****CFP+*** | Female | Drive/WT |
| KSM533 | OR694025 | AgNosCd-1 male x *An. gambiae* Kisumu female | Rep3 | F1 | ***Black****/****CFP+*** | Female | Drive/WT |
| KSM534 | OR694026 | AgNosCd-1 male x *An. gambiae* Kisumu female | Rep3 | F1 | ***Black****/****CFP+*** | Female | Drive/WT |
| KSM535 | OR694027 | AgNosCd-1 male x *An. gambiae* Kisumu female | Rep3 | F1 | ***Black****/****CFP+*** | Female | Drive/WT |
| KSM536 | OR694028 | AgNosCd-1 male x *An. gambiae* Kisumu female | Rep3 | F1 | ***Black****/****CFP+*** | Female | Drive/WT |
| KSM537 | OR694029 | AgNosCd-1 male x *An. gambiae* Kisumu female | Rep3 | F1 | ***Black****/****CFP+*** | Female | Drive/WT |
| KSM538 | OR694030 | AgNosCd-1 male x *An. gambiae* Kisumu female | Rep3 | F1 | ***Black****/****CFP+*** | Female | Drive/WT |
| KSM539 | OR694031 | AgNosCd-1 male x *An. gambiae* Kisumu female | Rep3 | F1 | ***Black****/****CFP+*** | Female | Drive/WT |
| KSM540 | OR694032 | AgNosCd-1 male x *An. gambiae* Kisumu female | Rep3 | F1 | ***Black****/****CFP+*** | Female | Drive/WT |
| KSM154 | OR693854 | AgNosCd-1 male/*An. gambiae* Kisumu female hybrid intercross | Rep 1 | F2 | ***Black****/****CFP+*** | Male | Drive/WT |
| KSM155 | OR693855 | AgNosCd-1 male/*An. gambiae* Kisumu female hybrid intercross | Rep 1 | F2 | ***Black****/****CFP+*** | Male | Drive/WT |
| KSM156 | OR693856 | AgNosCd-1 male/*An. gambiae* Kisumu female hybrid intercross | Rep 1 | F2 | ***Black****/****CFP+*** | Male | Drive/WT |
| KSM157 | OR693857 | AgNosCd-1 male/*An. gambiae* Kisumu female hybrid intercross | Rep 1 | F2 | ***Black****/****CFP+*** | Male | Drive/WT |
| KSM158 | OR693858 | AgNosCd-1 male/*An. gambiae* Kisumu female hybrid intercross | Rep 3 | F2 | ***Black****/****CFP+*** | Male | Drive/WT |
| KSM159 | OR693859 | AgNosCd-1 male/*An. gambiae* Kisumu female hybrid intercross | Rep 3 | F2 | ***Black****/****CFP+*** | Male | Drive/WT |
| KSM160 | OR693860 | AgNosCd-1 male/*An. gambiae* Kisumu female hybrid intercross | Rep 3 | F2 | ***Black****/****CFP+*** | Male | Drive/WT |
| KSM161 | OR693861 | AgNosCd-1 male/*An. gambiae* Kisumu female hybrid intercross | Rep 3 | F2 | ***Black****/****CFP+*** | Male | Drive/WT |
| KSM162 | OR693862 | AgNosCd-1 male/*An. gambiae* Kisumu female hybrid intercross | Rep 3 | F2 | ***Black****/****CFP+*** | Male | Drive/WT |
| KSM163 | OR693863 | AgNosCd-1 male/*An. gambiae* Kisumu female hybrid intercross | Rep 3 | F2 | ***Black****/****CFP+*** | Male | Drive/WT |
| KSM164 | OR693864 | AgNosCd-1 male/*An. gambiae* Kisumu female hybrid intercross | Rep 3 | F2 | ***Black****/****CFP+*** | Male | Drive/WT |
| KSM165 | OR693865 | AgNosCd-1 male/*An. gambiae* Kisumu female hybrid intercross | Rep 3 | F2 | ***Black****/****CFP+*** | Male | Drive/WT |
| KSM166 | OR693866 | AgNosCd-1 male/*An. gambiae* Kisumu female hybrid intercross | Rep 3 | F2 | ***Black****/****CFP+*** | Female | Drive/WT |
| KSM167 | OR693867 | AgNosCd-1 male/*An. gambiae* Kisumu female hybrid intercross | Rep 3 | F2 | ***Black****/****CFP+*** | Female | Drive/WT |
| KSM168 | OR693868 | AgNosCd-1 male/*An. gambiae* Kisumu female hybrid intercross | Rep 3 | F2 | ***Black****/****CFP+*** | Female | Drive/WT |
| KSM169 | OR693869 | AgNosCd-1 male/*An. gambiae* Kisumu female hybrid intercross | Rep 3 | F2 | ***Black****/****CFP+*** | Female | Drive/WT |
| KSM190 | OR693897 | AgNosCd-1 male/*An. gambiae* Kisumu female hybrid intercross | Rep 2 | F2 | ***Black****/****CFP+*** | Female | Drive/WT |
| KSM191 | OR693898 | AgNosCd-1 male/*An. gambiae* Kisumu female hybrid intercross | Rep 2 | F2 | ***Black****/****CFP+*** | Female | Drive/WT |
| KSM192 | OR693899 | AgNosCd-1 male/*An. gambiae* Kisumu female hybrid intercross | Rep 2 | F2 | ***Black****/****CFP+*** | Female | Drive/WT |
| KSM193 | OR693900 | AgNosCd-1 male/*An. gambiae* Kisumu female hybrid intercross | Rep 2 | F2 | ***Black****/****CFP+*** | Female | Drive/WT |
| KSM194 | OR693901 | AgNosCd-1 male/*An. gambiae* Kisumu female hybrid intercross | Rep 2 | F2 | ***Black****/****CFP+*** | Male | Drive/WT |
| KSM150 | OR693850 | *An. gambiae* Kisumu male/AgNosCd-1 female hybrid intercross | Rep 1 | F2 | ***Black****/****CFP+*** | Male | Drive/NHEJ |
| KSM151 | OR693851 | *An. gambiae* Kisumu male/AgNosCd-1 female hybrid intercross | Rep 1 | F2 | ***Black****/****CFP+*** | Male | Drive/NHEJ |
| KSM152 | OR693852 | *An. gambiae* Kisumu male/AgNosCd-1 female hybrid intercross | Rep 1 | F2 | ***Black****/****CFP+*** | Male | Drive/NHEJ |
| KSM153 | OR693853 | *An. gambiae* Kisumu male/AgNosCd-1 female hybrid intercross | Rep 1 | F2 | ***Black****/****CFP+*** | Male | Drive/WT |
| KSM184 | OR693891 | *An. gambiae* Kisumu male/AgNosCd-1 female hybrid intercross | Rep 3 | F2 | ***Black****/****CFP+*** | Male | Drive/WT |
| KSM185 | OR693892 | *An. gambiae* Kisumu male/AgNosCd-1 female hybrid intercross | Rep 3 | F2 | ***Black****/****CFP+*** | Male | Drive/NHEJ |
| KSM186 | OR693893 | *An. gambiae* Kisumu male/AgNosCd-1 female hybrid intercross | Rep 3 | F2 | ***Black****/****CFP+*** | Male | Drive/NHEJ |
| KSM187 | OR693894 | *An. gambiae* Kisumu male/AgNosCd-1 female hybrid intercross | Rep 3 | F2 | ***Black****/****CFP+*** | Female | Drive/NHEJ |
| KSM188 | OR693895 | *An. gambiae* Kisumu male/AgNosCd-1 female hybrid intercross | Rep 3 | F2 | ***Black****/****CFP+*** | Female | Drive/NHEJ |
| KSM189 | OR693896 | *An. gambiae* Kisumu male/AgNosCd-1 female hybrid intercross | Rep 3 | F2 | ***Black****/****CFP+*** | Female | Drive/NHEJ |
| KSM195 | OR693902 | *An. gambiae* Kisumu male/AgNosCd-1 female hybrid intercross | Rep 2 | F2 | ***Black****/****CFP+*** | Female | Drive/NHEJ |
| KSM196 | OR693903 | *An. gambiae* Kisumu male/AgNosCd-1 female hybrid intercross | Rep 2 | F2 | ***Black****/****CFP+*** | Female | Drive/NHEJ |
| KSM197 | OR693904 | *An. gambiae* Kisumu male/AgNosCd-1 female hybrid intercross | Rep 2 | F2 | ***Black****/****CFP+*** | Female | Drive/NHEJ |
| KSM198 | OR693905 | *An. gambiae* Kisumu male/AgNosCd-1 female hybrid intercross | Rep 2 | F2 | ***Black****/****CFP+*** | Male | Drive/NHEJ |
| KSM199 | OR693906 | *An. gambiae* Kisumu male/AgNosCd-1 female hybrid intercross | Rep 2 | F2 | ***Black****/****CFP+*** | Male | Drive/NHEJ |
| KSM107 | OR693814 | *An. gambiae* Kisumu male/AgNosCd-1 female hybrid intercross | Rep 1 | F2 | ***Black****/CFP-* | Female | NHEJ/NHEJ |
|  | OR693815 |  |  |  |  |  |  |
| KSM108 | OR693816 | *An. gambiae* Kisumu male/AgNosCd-1 female hybrid intercross | Rep 1 | F2 | ***Black****/CFP-* | Female | NHEJ/NHEJ |
|  | OR693817 |  |  |  |  |  |  |
| KSM109 | OR693818 | *An. gambiae* Kisumu male/AgNosCd-1 female hybrid intercross | Rep 1 | F2 | ***Black****/CFP-* | Female | NHEJ/NHEJ |
|  | OR693819 |  |  |  |  |  |  |
| KSM110 | OR693820 | *An. gambiae* Kisumu male/AgNosCd-1 female hybrid intercross | Rep 1 | F2 | ***Black****/CFP-* | Female | NHEJ/NHEJ |
|  | OR693821 |  |  |  |  |  |  |
| KSM111 | OR693822 | *An. gambiae* Kisumu male/AgNosCd-1 female hybrid intercross | Rep 1 | F2 | ***Black****/CFP-* | Female | NHEJ/NHEJ |
|  | OR693823 |  |  |  |  |  |  |
| KSM112 | OR693824 | *An. gambiae* Kisumu male/AgNosCd-1 female hybrid intercross | Rep 1 | F2 | ***Black****/CFP-* | Female | NHEJ/NHEJ |
|  | OR693825 |  |  |  |  |  |  |
| KSM113 | OR693826 | *An. gambiae* Kisumu male/AgNosCd-1 female hybrid intercross | Rep 1 | F2 | ***Black****/CFP-* | Female | NHEJ/NHEJ |
|  | OR693827 |  |  |  |  |  |  |
| KSM114 | OR693828 | *An. gambiae* Kisumu male/AgNosCd-1 female hybrid intercross | Rep 1 | F2 | ***Black****/CFP-* | Male | NHEJ/NHEJ |
|  | OR693829 |  |  |  |  |  |  |
| KSM115 | OR693830 | *An. gambiae* Kisumu male/AgNosCd-1 female hybrid intercross | Rep 1 | F2 | ***Black****/CFP-* | Male | NHEJ/NHEJ |
|  | OR693831 |  |  |  |  |  |  |
| KSM117 | OR693832 | *An. gambiae* Kisumu male/AgNosCd-1 female hybrid intercross | Rep 1 | F2 | ***Black****/CFP-* | Male | NHEJ/NHEJ |
|  | OR693833 |  |  |  |  |  |  |
| KSM179 | OR693886 | *An. gambiae* Kisumu male/AgNosCd-1 female hybrid intercross | Rep 3 | F2 | ***Black****/CFP-* | Male | WT/NHEJ |
| KSM180 | OR693887 | *An. gambiae* Kisumu male/AgNosCd-1 female hybrid intercross | Rep 3 | F2 | ***Black****/CFP-* | Male | WT/NHEJ |
| KSM181 | OR693888 | *An. gambiae* Kisumu male/AgNosCd-1 female hybrid intercross | Rep 3 | F2 | ***Black****/CFP-* | Female | WT/NHEJ |
| KSM182 | OR693889 | *An. gambiae* Kisumu male/AgNosCd-1 female hybrid intercross | Rep 3 | F2 | ***Black****/CFP-* | Female | WT/NHEJ |
| KSM183 | OR693890 | *An. gambiae* Kisumu male/AgNosCd-1 female hybrid intercross | Rep 3 | F2 | ***Black****/CFP-* | Female | WT/NHEJ |
| KSM106 | OR693812 | *An. gambiae* Kisumu male/AgNosCd-1 female hybrid intercross | Rep 1 | F2 | *Cardinal/CFP-* | Female | NHEJ/NHEJ |
|  | OR693813 |  |  |  |  |  |  |
| KSM178 | OR693884 | *An. gambiae* Kisumu male/AgNosCd-1 female hybrid intercross | Rep 3 | F2 | *Cardinal/CFP-* | Male | NHEJ/NHEJ |
|  | OR693885 |  |  |  |  |  |  |
| KSM200 | OR693907 | *An. gambiae* Kisumu male/AgNosCd-1 female hybrid intercross | Rep 2 | F2 | *Cardinal/CFP-* | | NHEJ/NHEJ |
| KSM301 |  | AgNosCd-1 male/*An. gambiae* Kisumu female hybrid intercross | Rep1 | F2 | *Cardinal/CFP+* | Male | Drive/Drive |
| KSM302 |  | AgNosCd-1 male/*An. gambiae* Kisumu female hybrid intercross | Rep1 | F2 | *Cardinal/CFP+* | Male | Drive/Drive |
| KSM303 |  | AgNosCd-1 male/*An. gambiae* Kisumu female hybrid intercross | Rep1 | F2 | *Cardinal/CFP+* | Male | Drive/Drive |
| KSM304 |  | AgNosCd-1 male/*An. gambiae* Kisumu female hybrid intercross | Rep1 | F2 | *Cardinal/CFP+* | Male | Drive/Drive |
| KSM305 |  | AgNosCd-1 male/*An. gambiae* Kisumu female hybrid intercross | Rep1 | F2 | *Cardinal/CFP+* | Male | Drive/Drive |
| KSM306 |  | AgNosCd-1 male/*An. gambiae* Kisumu female hybrid intercross | Rep1 | F2 | *Cardinal/CFP+* | Male | Drive/Drive |
| KSM307 |  | AgNosCd-1 male/*An. gambiae* Kisumu female hybrid intercross | Rep1 | F2 | *Cardinal/CFP+* | Male | Drive/Drive |
| KSM308 |  | AgNosCd-1 male/*An. gambiae* Kisumu female hybrid intercross | Rep1 | F2 | *Cardinal/CFP+* | Male | Drive/Drive |
| KSM309 |  | AgNosCd-1 male/*An. gambiae* Kisumu female hybrid intercross | Rep1 | F2 | *Cardinal/CFP+* | Male | Drive/Drive |
| KSM310 |  | AgNosCd-1 male/*An. gambiae* Kisumu female hybrid intercross | Rep1 | F2 | *Cardinal/CFP+* | Male | Drive/Drive |
| KSM311 |  | AgNosCd-1 male/*An. gambiae* Kisumu female hybrid intercross | Rep1 | F2 | *Cardinal/CFP+* | Female | Drive/Drive |
| KSM312 |  | AgNosCd-1 male/*An. gambiae* Kisumu female hybrid intercross | Rep1 | F2 | *Cardinal/CFP+* | Female | Drive/Drive |
| KSM313 |  | AgNosCd-1 male/*An. gambiae* Kisumu female hybrid intercross | Rep1 | F2 | *Cardinal/CFP+* | Female | Drive/Drive |
| KSM314 |  | AgNosCd-1 male/*An. gambiae* Kisumu female hybrid intercross | Rep1 | F2 | *Cardinal/CFP+* | Female | Drive/Drive |
| KSM315 |  | AgNosCd-1 male/*An. gambiae* Kisumu female hybrid intercross | Rep1 | F2 | *Cardinal/CFP+* | Female | Drive/Drive |
| KSM316 |  | AgNosCd-1 male/*An. gambiae* Kisumu female hybrid intercross | Rep1 | F2 | *Cardinal/CFP+* | Female | Drive/Drive |
| KSM317 |  | AgNosCd-1 male/*An. gambiae* Kisumu female hybrid intercross | Rep1 | F2 | *Cardinal/CFP+* | Female | Drive/Drive |
| KSM318 |  | AgNosCd-1 male/*An. gambiae* Kisumu female hybrid intercross | Rep1 | F2 | *Cardinal/CFP+* | Female | Drive/Drive |
| KSM319 |  | AgNosCd-1 male/*An. gambiae* Kisumu female hybrid intercross | Rep1 | F2 | *Cardinal/CFP+* | Female | Drive/Drive |
| KSM320 |  | AgNosCd-1 male/*An. gambiae* Kisumu female hybrid intercross | Rep1 | F2 | *Cardinal/CFP+* | Female | Drive/Drive |
| KSM321 |  | AgNosCd-1 male/*An. gambiae* Kisumu female hybrid intercross | Rep2 | F2 | *Cardinal/CFP+* | Male | Drive/Drive |
| KSM322 |  | AgNosCd-1 male/*An. gambiae* Kisumu female hybrid intercross | Rep2 | F2 | *Cardinal/CFP+* | Male | Drive/Drive |
| KSM323 |  | AgNosCd-1 male/*An. gambiae* Kisumu female hybrid intercross | Rep2 | F2 | *Cardinal/CFP+* | Male | Drive/Drive |
| KSM324 |  | AgNosCd-1 male/*An. gambiae* Kisumu female hybrid intercross | Rep2 | F2 | *Cardinal/CFP+* | Male | Drive/Drive |
| KSM325 |  | AgNosCd-1 male/*An. gambiae* Kisumu female hybrid intercross | Rep2 | F2 | *Cardinal/CFP+* | Male | Drive/Drive |
| KSM326 |  | AgNosCd-1 male/*An. gambiae* Kisumu female hybrid intercross | Rep2 | F2 | *Cardinal/CFP+* | Male | Drive/Drive |
| KSM327 |  | AgNosCd-1 male/*An. gambiae* Kisumu female hybrid intercross | Rep2 | F2 | *Cardinal/CFP+* | Male | Drive/Drive |
| KSM328 |  | AgNosCd-1 male/*An. gambiae* Kisumu female hybrid intercross | Rep2 | F2 | *Cardinal/CFP+* | Male | Drive/Drive |
| KSM329 |  | AgNosCd-1 male/*An. gambiae* Kisumu female hybrid intercross | Rep2 | F2 | *Cardinal/CFP+* | Male | Drive/Drive |
| KSM330 |  | AgNosCd-1 male/*An. gambiae* Kisumu female hybrid intercross | Rep2 | F2 | *Cardinal/CFP+* | Male | Drive/Drive |
| KSM331 |  | AgNosCd-1 male/*An. gambiae* Kisumu female hybrid intercross | Rep2 | F2 | *Cardinal/CFP+* | Female | Drive/Drive |
| KSM332 |  | AgNosCd-1 male/*An. gambiae* Kisumu female hybrid intercross | Rep2 | F2 | *Cardinal/CFP+* | Female | Drive/Drive |
| KSM333 |  | AgNosCd-1 male/*An. gambiae* Kisumu female hybrid intercross | Rep2 | F2 | *Cardinal/CFP+* | Female | Drive/Drive |
| KSM334 |  | AgNosCd-1 male/*An. gambiae* Kisumu female hybrid intercross | Rep2 | F2 | *Cardinal/CFP+* | Female | Drive/Drive |
| KSM335 |  | AgNosCd-1 male/*An. gambiae* Kisumu female hybrid intercross | Rep2 | F2 | *Cardinal/CFP+* | Female | Drive/Drive |
| KSM336 |  | AgNosCd-1 male/*An. gambiae* Kisumu female hybrid intercross | Rep2 | F2 | *Cardinal/CFP+* | Female | Drive/Drive |
| KSM337 |  | AgNosCd-1 male/*An. gambiae* Kisumu female hybrid intercross | Rep2 | F2 | *Cardinal/CFP+* | Female | Drive/Drive |
| KSM338 |  | AgNosCd-1 male/*An. gambiae* Kisumu female hybrid intercross | Rep2 | F2 | *Cardinal/CFP+* | Female | Drive/Drive |
| KSM339 |  | AgNosCd-1 male/*An. gambiae* Kisumu female hybrid intercross | Rep2 | F2 | *Cardinal/CFP+* | Female | Drive/Drive |
| KSM340 |  | AgNosCd-1 male/*An. gambiae* Kisumu female hybrid intercross | Rep2 | F2 | *Cardinal/CFP+* | Female | Drive/Drive |
| KSM341 |  | AgNosCd-1 male/*An. gambiae* Kisumu female hybrid intercross | Rep3 | F2 | *Cardinal/CFP+* | Male | Drive/Drive |
| KSM342 |  | AgNosCd-1 male/*An. gambiae* Kisumu female hybrid intercross | Rep3 | F2 | *Cardinal/CFP+* | Male | Drive/Drive |
| KSM343 |  | AgNosCd-1 male/*An. gambiae* Kisumu female hybrid intercross | Rep3 | F2 | *Cardinal/CFP+* | Male | Drive/Drive |
| KSM344 |  | AgNosCd-1 male/*An. gambiae* Kisumu female hybrid intercross | Rep3 | F2 | *Cardinal/CFP+* | Male | Drive/Drive |
| KSM345 |  | AgNosCd-1 male/*An. gambiae* Kisumu female hybrid intercross | Rep3 | F2 | *Cardinal/CFP+* | Male | Drive/Drive |
| KSM346 |  | AgNosCd-1 male/*An. gambiae* Kisumu female hybrid intercross | Rep3 | F2 | *Cardinal/CFP+* | Male | Drive/Drive |
| KSM347 |  | AgNosCd-1 male/*An. gambiae* Kisumu female hybrid intercross | Rep3 | F2 | *Cardinal/CFP+* | Male | Drive/Drive |
| KSM348 |  | AgNosCd-1 male/*An. gambiae* Kisumu female hybrid intercross | Rep3 | F2 | *Cardinal/CFP+* | Male | Drive/Drive |
| KSM349 |  | AgNosCd-1 male/*An. gambiae* Kisumu female hybrid intercross | Rep3 | F2 | *Cardinal/CFP+* | Male | Drive/Drive |
| KSM350 |  | AgNosCd-1 male/*An. gambiae* Kisumu female hybrid intercross | Rep3 | F2 | *Cardinal/CFP+* | Male | Drive/Drive |
| KSM351 |  | AgNosCd-1 male/*An. gambiae* Kisumu female hybrid intercross | Rep3 | F2 | *Cardinal/CFP+* | Female | Drive/Drive |
| KSM352 |  | AgNosCd-1 male/*An. gambiae* Kisumu female hybrid intercross | Rep3 | F2 | *Cardinal/CFP+* | Female | Drive/Drive |
| KSM353 |  | AgNosCd-1 male/*An. gambiae* Kisumu female hybrid intercross | Rep3 | F2 | *Cardinal/CFP+* | Female | Drive/Drive |
| KSM354 |  | AgNosCd-1 male/*An. gambiae* Kisumu female hybrid intercross | Rep3 | F2 | *Cardinal/CFP+* | Female | Drive/Drive |
| KSM355 |  | AgNosCd-1 male/*An. gambiae* Kisumu female hybrid intercross | Rep3 | F2 | *Cardinal/CFP+* | Female | Drive/Drive |
| KSM356 |  | AgNosCd-1 male/*An. gambiae* Kisumu female hybrid intercross | Rep3 | F2 | *Cardinal/CFP+* | Female | Drive/Drive |
| KSM357 |  | AgNosCd-1 male/*An. gambiae* Kisumu female hybrid intercross | Rep3 | F2 | *Cardinal/CFP+* | Female | Drive/Drive |
| KSM358 |  | AgNosCd-1 male/*An. gambiae* Kisumu female hybrid intercross | Rep3 | F2 | *Cardinal/CFP+* | Female | Drive/Drive |
| KSM359 |  | AgNosCd-1 male/*An. gambiae* Kisumu female hybrid intercross | Rep3 | F2 | *Cardinal/CFP+* | Female | Drive/Drive |
| KSM360 |  | AgNosCd-1 male/*An. gambiae* Kisumu female hybrid intercross | Rep3 | F2 | *Cardinal/CFP+* | Female | Drive/Drive |
| KSM361 |  | *An. gambiae* Kisumu male/AgNosCd-1 female hybrid intercross | Rep1 | F2 | *Cardinal/CFP+* | Male | Drive/Drive |
| KSM362 |  | *An. gambiae* Kisumu male/AgNosCd-1 female hybrid intercross | Rep1 | F2 | *Cardinal/CFP+* | Male | Drive/Drive |
| KSM363 |  | *An. gambiae* Kisumu male/AgNosCd-1 female hybrid intercross | Rep1 | F2 | *Cardinal/CFP+* | Male | Drive/Drive |
| KSM364 |  | *An. gambiae* Kisumu male/AgNosCd-1 female hybrid intercross | Rep1 | F2 | *Cardinal/CFP+* | Male | Drive/Drive |
| KSM365 |  | *An. gambiae* Kisumu male/AgNosCd-1 female hybrid intercross | Rep1 | F2 | *Cardinal/CFP+* | Male | Drive/Drive |
| KSM366 |  | *An. gambiae* Kisumu male/AgNosCd-1 female hybrid intercross | Rep1 | F2 | *Cardinal/CFP+* | Male | Drive/Drive |
| KSM367 |  | *An. gambiae* Kisumu male/AgNosCd-1 female hybrid intercross | Rep1 | F2 | *Cardinal/CFP+* | Male | Drive/Drive |
| KSM368 |  | *An. gambiae* Kisumu male/AgNosCd-1 female hybrid intercross | Rep1 | F2 | *Cardinal/CFP+* | Male | Drive/Drive |
| KSM369 |  | *An. gambiae* Kisumu male/AgNosCd-1 female hybrid intercross | Rep1 | F2 | *Cardinal/CFP+* | Male | Drive/Drive |
| KSM370 |  | *An. gambiae* Kisumu male/AgNosCd-1 female hybrid intercross | Rep1 | F2 | *Cardinal/CFP+* | Male | Drive/Drive |
| KSM371 |  | *An. gambiae* Kisumu male/AgNosCd-1 female hybrid intercross | Rep1 | F2 | *Cardinal/CFP+* | Female | Drive/Drive |
| KSM372 |  | *An. gambiae* Kisumu male/AgNosCd-1 female hybrid intercross | Rep1 | F2 | *Cardinal/CFP+* | Female | Drive/Drive |
| KSM373 |  | *An. gambiae* Kisumu male/AgNosCd-1 female hybrid intercross | Rep1 | F2 | *Cardinal/CFP+* | Female | Drive/Drive |
| KSM374 |  | *An. gambiae* Kisumu male/AgNosCd-1 female hybrid intercross | Rep1 | F2 | *Cardinal/CFP+* | Female | Drive/Drive |
| KSM375 |  | *An. gambiae* Kisumu male/AgNosCd-1 female hybrid intercross | Rep1 | F2 | *Cardinal/CFP+* | Female | Drive/Drive |
| KSM376 |  | *An. gambiae* Kisumu male/AgNosCd-1 female hybrid intercross | Rep1 | F2 | *Cardinal/CFP+* | Female | Drive/Drive |
| KSM377 | OR693908 | *An. gambiae* Kisumu male/AgNosCd-1 female hybrid intercross | Rep1 | F2 | *Cardinal/CFP+* | Female | Drive/NHEJ |
| KSM378 |  | *An. gambiae* Kisumu male/AgNosCd-1 female hybrid intercross | Rep1 | F2 | *Cardinal/CFP+* | Female | Drive/Drive |
| KSM379 | OR693909 | *An. gambiae* Kisumu male/AgNosCd-1 female hybrid intercross | Rep1 | F2 | *Cardinal/CFP+* | Female | Drive/NHEJ |
| KSM380 |  | *An. gambiae* Kisumu male/AgNosCd-1 female hybrid intercross | Rep1 | F2 | *Cardinal/CFP+* | Female | Drive/Drive |
| KSM381 |  | *An. gambiae* Kisumu male/AgNosCd-1 female hybrid intercross | Rep2 | F2 | *Cardinal/CFP+* | Male | Drive/Drive |
| KSM382 |  | *An. gambiae* Kisumu male/AgNosCd-1 female hybrid intercross | Rep2 | F2 | *Cardinal/CFP+* | Male | Drive/Drive |
| KSM383 |  | *An. gambiae* Kisumu male/AgNosCd-1 female hybrid intercross | Rep2 | F2 | *Cardinal/CFP+* | Male | Drive/Drive |
| KSM384 |  | *An. gambiae* Kisumu male/AgNosCd-1 female hybrid intercross | Rep2 | F2 | *Cardinal/CFP+* | Male | Drive/Drive |
| KSM385 |  | *An. gambiae* Kisumu male/AgNosCd-1 female hybrid intercross | Rep2 | F2 | *Cardinal/CFP+* | Male | Drive/Drive |
| KSM386 |  | *An. gambiae* Kisumu male/AgNosCd-1 female hybrid intercross | Rep2 | F2 | *Cardinal/CFP+* | Male | Drive/Drive |
| KSM387 |  | *An. gambiae* Kisumu male/AgNosCd-1 female hybrid intercross | Rep2 | F2 | *Cardinal/CFP+* | Male | Drive/Drive |
| KSM388 |  | *An. gambiae* Kisumu male/AgNosCd-1 female hybrid intercross | Rep2 | F2 | *Cardinal/CFP+* | Male | Drive/Drive |
| KSM389 |  | *An. gambiae* Kisumu male/AgNosCd-1 female hybrid intercross | Rep2 | F2 | *Cardinal/CFP+* | Male | Drive/Drive |
| KSM390 |  | *An. gambiae* Kisumu male/AgNosCd-1 female hybrid intercross | Rep2 | F2 | *Cardinal/CFP+* | Male | Drive/Drive |
| KSM391 |  | *An. gambiae* Kisumu male/AgNosCd-1 female hybrid intercross | Rep2 | F2 | *Cardinal/CFP+* | Female | Drive/Drive |
| KSM392 |  | *An. gambiae* Kisumu male/AgNosCd-1 female hybrid intercross | Rep2 | F2 | *Cardinal/CFP+* | Female | Drive/Drive |
| KSM393 |  | *An. gambiae* Kisumu male/AgNosCd-1 female hybrid intercross | Rep2 | F2 | *Cardinal/CFP+* | Female | Drive/Drive |
| KSM394 |  | *An. gambiae* Kisumu male/AgNosCd-1 female hybrid intercross | Rep2 | F2 | *Cardinal/CFP+* | Female | Drive/Drive |
| KSM395 |  | *An. gambiae* Kisumu male/AgNosCd-1 female hybrid intercross | Rep2 | F2 | *Cardinal/CFP+* | Female | Drive/Drive |
| KSM396 |  | *An. gambiae* Kisumu male/AgNosCd-1 female hybrid intercross | Rep2 | F2 | *Cardinal/CFP+* | Female | Drive/Drive |
| KSM397 |  | *An. gambiae* Kisumu male/AgNosCd-1 female hybrid intercross | Rep2 | F2 | *Cardinal/CFP+* | Female | Drive/Drive |
| KSM398 |  | *An. gambiae* Kisumu male/AgNosCd-1 female hybrid intercross | Rep2 | F2 | *Cardinal/CFP+* | Female | Drive/Drive |
| KSM399 |  | *An. gambiae* Kisumu male/AgNosCd-1 female hybrid intercross | Rep2 | F2 | *Cardinal/CFP+* | Female | Drive/Drive |
| KSM400 | OR693910 | *An. gambiae* Kisumu male/AgNosCd-1 female hybrid intercross | Rep2 | F2 | *Cardinal/CFP+* | Female | Drive/NHEJ |
| KSM401 |  | *An. gambiae* Kisumu male/AgNosCd-1 female hybrid intercross | Rep3 | F2 | *Cardinal/CFP+* | Male | Drive/Drive |
| KSM402 |  | *An. gambiae* Kisumu male/AgNosCd-1 female hybrid intercross | Rep3 | F2 | *Cardinal/CFP+* | Male | Drive/Drive |
| KSM403 |  | *An. gambiae* Kisumu male/AgNosCd-1 female hybrid intercross | Rep3 | F2 | *Cardinal/CFP+* | Male | Drive/Drive |
| KSM404 |  | *An. gambiae* Kisumu male/AgNosCd-1 female hybrid intercross | Rep3 | F2 | *Cardinal/CFP+* | Male | Drive/Drive |
| KSM405 |  | *An. gambiae* Kisumu male/AgNosCd-1 female hybrid intercross | Rep3 | F2 | *Cardinal/CFP+* | Male | Drive/Drive |
| KSM406 |  | *An. gambiae* Kisumu male/AgNosCd-1 female hybrid intercross | Rep3 | F2 | *Cardinal/CFP+* | Male | Drive/Drive |
| KSM407 |  | *An. gambiae* Kisumu male/AgNosCd-1 female hybrid intercross | Rep3 | F2 | *Cardinal/CFP+* | Male | Drive/Drive |
| KSM408 |  | *An. gambiae* Kisumu male/AgNosCd-1 female hybrid intercross | Rep3 | F2 | *Cardinal/CFP+* | Male | Drive/Drive |
| KSM409 |  | *An. gambiae* Kisumu male/AgNosCd-1 female hybrid intercross | Rep3 | F2 | *Cardinal/CFP+* | Male | Drive/Drive |
| KSM410 |  | *An. gambiae* Kisumu male/AgNosCd-1 female hybrid intercross | Rep3 | F2 | *Cardinal/CFP+* | Male | Drive/Drive |
| KSM411 |  | *An. gambiae* Kisumu male/AgNosCd-1 female hybrid intercross | Rep3 | F2 | *Cardinal/CFP+* | Female | Drive/Drive |
| KSM412 |  | *An. gambiae* Kisumu male/AgNosCd-1 female hybrid intercross | Rep3 | F2 | *Cardinal/CFP+* | Female | Drive/Drive |
| KSM413 |  | *An. gambiae* Kisumu male/AgNosCd-1 female hybrid intercross | Rep3 | F2 | *Cardinal/CFP+* | Female | Drive/Drive |
| KSM414 |  | *An. gambiae* Kisumu male/AgNosCd-1 female hybrid intercross | Rep3 | F2 | *Cardinal/CFP+* | Female | Drive/Drive |
| KSM415 |  | *An. gambiae* Kisumu male/AgNosCd-1 female hybrid intercross | Rep3 | F2 | *Cardinal/CFP+* | Female | Drive/Drive |
| KSM416 |  | *An. gambiae* Kisumu male/AgNosCd-1 female hybrid intercross | Rep3 | F2 | *Cardinal/CFP+* | Female | Drive/Drive |
| KSM417 |  | *An. gambiae* Kisumu male/AgNosCd-1 female hybrid intercross | Rep3 | F2 | *Cardinal/CFP+* | Female | Drive/Drive |
| KSM418 | OR693911 | *An. gambiae* Kisumu male/AgNosCd-1 female hybrid intercross | Rep3 | F2 | *Cardinal/CFP+* | Female | Drive/NHEJ |
| KSM419 |  | *An. gambiae* Kisumu male/AgNosCd-1 female hybrid intercross | Rep3 | F2 | *Cardinal/CFP+* | Female | Drive/Drive |
| KSM420 |  | *An. gambiae* Kisumu male/AgNosCd-1 female hybrid intercross | Rep3 | F2 | *Cardinal/CFP+* | Female | Drive/Drive |
| ND1 | OR694033 | ***An. gambiae* Ndoyako** male x **AgNosCd-1** female | Rep 1 | Parent | ***Black****/CFP-* | Male | WT |
| ND2 | OR694034 | ***An. gambiae* Ndoyako** male x **AgNosCd-1** female | Rep 1 | Parent | ***Black****/CFP-* | Male | WT |
|  | OR694035 |  |  |  |  |  |  |
| ND3 | OR694036 | ***An. gambiae* Ndoyako** male x **AgNosCd-1** female | Rep 1 | Parent | ***Black****/CFP-* | Male | WT |
| ND4 | OR694037 | ***An. gambiae* Ndoyako** male x **AgNosCd-1** female | Rep 1 | Parent | ***Black****/CFP-* | Male | WT |
|  | OR694038 |  |  |  |  |  |  |
| ND5 | OR694039 | ***An. gambiae* Ndoyako** male x **AgNosCd-1** female | Rep 1 | Parent | ***Black****/CFP-* | Male | WT |
| ND6 | OR694040 | ***An. gambiae* Ndoyako** male x **AgNosCd-1** female | Rep 1 | Parent | ***Black****/CFP-* | Male | WT |
| ND7 | OR694041 | ***An. gambiae* Ndoyako** male x **AgNosCd-1** female | Rep 1 | Parent | ***Black****/CFP-* | Male | WT |
|  | OR694042 |  |  |  |  |  |  |
| ND8 | OR694043 | ***An. gambiae* Ndoyako** male x **AgNosCd-1** female | Rep 1 | Parent | ***Black****/CFP-* | Male | WT |
| ND9 | OR694044 | ***An. gambiae* Ndoyako** male x **AgNosCd-1** female | Rep 1 | Parent | ***Black****/CFP-* | Male | WT |
|  | OR694045 |  |  |  |  |  |  |
| ND10 | OR694046 | ***An. gambiae* Ndoyako** male x **AgNosCd-1** female | Rep 1 | Parent | ***Black****/CFP-* | Male | WT |
| ND11 | OR694047 | ***An. gambiae* Ndoyako** male x **AgNosCd-1** female | Rep 1 | Parent | ***Black****/CFP-* | Male | WT |
|  | OR694048 |  |  |  |  |  |  |
| ND12 | OR694049 | ***An. gambiae* Ndoyako** male x **AgNosCd-1** female | Rep 1 | Parent | ***Black****/CFP-* | Male | WT |
|  | OR694050 |  |  |  |  |  |  |
| ND25 | OR694067 | ***An. gambiae* Ndoyako** male x **AgNosCd-1** female | Rep 1 | Parent | ***Black****/CFP-* | Male | WT |
|  | OR694068 |  |  |  |  |  |  |
| ND27 | OR694069 | ***An. gambiae* Ndoyako** male x **AgNosCd-1** female | Rep 1 | Parent | ***Black****/CFP-* | Male | WT |
|  | OR694070 |  |  |  |  |  |  |
| ND28 | OR694071 | ***An. gambiae* Ndoyako** male x **AgNosCd-1** female | Rep 1 | Parent | ***Black****/CFP-* | Male | WT |
|  | OR694072 |  |  |  |  |  |  |
| ND29 | OR694073 | ***An. gambiae* Ndoyako** male x **AgNosCd-1** female | Rep 1 | Parent | ***Black****/CFP-* | Male | WT |
|  | OR694074 |  |  |  |  |  |  |
| ND30 | OR694075 | ***An. gambiae* Ndoyako** male x **AgNosCd-1** female | Rep 1 | Parent | ***Black****/CFP-* | Male | WT |
|  | OR694076 |  |  |  |  |  |  |
| ND31 | OR694077 | ***An. gambiae* Ndoyako** male x **AgNosCd-1** female | Rep 1 | Parent | ***Black****/CFP-* | Male | WT |
|  | OR694078 |  |  |  |  |  |  |
| ND32 | OR694079 | ***An. gambiae* Ndoyako** male x **AgNosCd-1** female | Rep 1 | Parent | ***Black****/CFP-* | Male | WT |
| ND13 | OR694051 | **AgNosCd-1** male x ***An. gambiae* Ndoyako** female | Rep 1 | Parent | ***Black****/CFP-* | Female | WT |
|  | OR694052 |  |  |  |  |  |  |
| ND14 | OR694053 | **AgNosCd-1** male x ***An. gambiae* Ndoyako** female | Rep 1 | Parent | ***Black****/CFP-* | Female | WT |
| ND15 | OR694054 | **AgNosCd-1** male x ***An. gambiae* Ndoyako** female | Rep 1 | Parent | ***Black****/CFP-* | Female | WT |
| ND16 | OR694055 | **AgNosCd-1** male x ***An. gambiae* Ndoyako** female | Rep 1 | Parent | ***Black****/CFP-* | Female | WT |
| ND17 | OR694056 | **AgNosCd-1** male x ***An. gambiae* Ndoyako** female | Rep 1 | Parent | ***Black****/CFP-* | Female | WT |
|  | OR694057 |  |  |  |  |  |  |
| ND18 | OR694058 | **AgNosCd-1** male x ***An. gambiae* Ndoyako** female | Rep 1 | Parent | ***Black****/CFP-* | Female | WT |
|  | OR694059 |  |  |  |  |  | WT |
| ND20 | OR694060 | **AgNosCd-1** male x ***An. gambiae* Ndoyako** female | Rep 1 | Parent | ***Black****/CFP-* | Female | WT |
|  | OR694061 |  |  |  |  |  |  |
| ND21 | OR694062 | **AgNosCd-1** male x ***An. gambiae* Ndoyako** female | Rep 1 | Parent | ***Black****/CFP-* | Female | WT |
| ND22 | OR694063 | **AgNosCd-1** male x ***An. gambiae* Ndoyako** female | Rep 1 | Parent | ***Black****/CFP-* | Female | WT |
| ND23 | OR694064 | **AgNosCd-1** male x ***An. gambiae* Ndoyako** female | Rep 1 | Parent | ***Black****/CFP-* | Female | WT |
|  | OR694065 |  |  |  |  |  |  |
| ND24 | OR694066 | **AgNosCd-1** male x ***An. gambiae* Ndoyako** female | Rep 1 | Parent | ***Black****/CFP-* | Female | WT |
| ND33 | OR694080 | **AgNosCd-1** male x ***An. gambiae* Ndoyako** female | Rep 1 | Parent | ***Black****/CFP-* | Female | WT |
| ND34 | OR694081 | **AgNosCd-1** male x ***An. gambiae* Ndoyako** female | Rep 1 | Parent | ***Black****/CFP-* | Female | WT |
|  | OR694082 |  |  |  |  |  |  |
| ND35 | OR694083 | **AgNosCd-1** male x ***An. gambiae* Ndoyako** female | Rep 1 | Parent | ***Black****/CFP-* | Female | WT |
| ND37 | OR694084 | **AgNosCd-1** male x ***An. gambiae* Ndoyako** female | Rep 1 | Parent | ***Black****/CFP-* | Female | WT |
| ND38 | OR694085 | **AgNosCd-1** male x ***An. gambiae* Ndoyako** female | Rep 1 | Parent | ***Black****/CFP-* | Female | WT |
|  | OR694086 |  |  |  |  |  |  |
| ND39 | OR694087 | **AgNosCd-1** male x ***An. gambiae* Ndoyako** female | Rep 1 | Parent | ***Black****/CFP-* | Female | WT |
|  | OR694088 |  |  |  |  |  |  |
| ND40 | OR694089 | **AgNosCd-1** male x ***An. gambiae* Ndoyako** female | Rep 1 | Parent | ***Black****/CFP-* | Female | WT |
| ND41 | OR694090 | **AgNosCd-1** male x ***An. gambiae* Ndoyako** female | Rep 1 | Parent | ***Black****/CFP-* | Female | WT |
| ND42 | OR694091 | **AgNosCd-1** male x ***An. gambiae* Ndoyako** female | Rep 1 | Parent | ***Black****/CFP-* | Female | WT |
|  | OR694092 |  |  |  |  |  |  |
| ND43 | OR694093 | **AgNosCd-1** male x ***An. gambiae* Ndoyako** female | Rep 1 | Parent | ***Black****/CFP-* | Female | WT |
|  | OR694094 |  |  |  |  |  |  |
| ND67 | OR694120 | ***An. gambiae* Ndoyako** male x **AgNosCd-1** female | Rep 2 | Parent | ***Black****/CFP-* | Male | WT |
| ND68 | OR694121 | ***An. gambiae* Ndoyako** male x **AgNosCd-1** female | Rep 2 | Parent | ***Black****/CFP-* | Male | WT |
| ND69 | OR694122 | ***An. gambiae* Ndoyako** male x **AgNosCd-1** female | Rep 2 | Parent | ***Black****/CFP-* | Male | WT |
|  | OR694123 |  |  |  |  |  |  |
| ND70 | OR694124 | ***An. gambiae* Ndoyako** male x **AgNosCd-1** female | Rep 2 | Parent | ***Black****/CFP-* | Male | WT |
|  | OR694125 |  |  |  |  |  |  |
| ND71 | OR694126 | ***An. gambiae* Ndoyako** male x **AgNosCd-1** female | Rep 2 | Parent | ***Black****/CFP-* | Male | WT |
| ND72 | OR694127 | ***An. gambiae* Ndoyako** male x **AgNosCd-1** female | Rep 2 | Parent | ***Black****/CFP-* | Male | WT |
|  | OR694128 |  |  |  |  |  |  |
| ND73 | OR694129 | ***An. gambiae* Ndoyako** male x **AgNosCd-1** female | Rep 2 | Parent | ***Black****/CFP-* | Male | WT |
|  | OR694130 |  |  |  |  |  |  |
| ND74 | OR694131 | ***An. gambiae* Ndoyako** male x **AgNosCd-1** female | Rep 2 | Parent | ***Black****/CFP-* | Male | WT |
|  | OR694132 |  |  |  |  |  |  |
| ND75 | OR694133 | ***An. gambiae* Ndoyako** male x **AgNosCd-1** female | Rep 2 | Parent | ***Black****/CFP-* | Male | WT |
|  | OR694134 |  |  |  |  |  |  |
| ND76 | OR694135 | ***An. gambiae* Ndoyako** male x **AgNosCd-1** female | Rep 2 | Parent | ***Black****/CFP-* | Male | WT |
| ND77 | OR694136 | ***An. gambiae* Ndoyako** male x **AgNosCd-1** female | Rep 2 | Parent | ***Black****/CFP-* | Male | WT |
|  | OR694137 |  |  |  |  |  |  |
| ND78 | OR694138 | ***An. gambiae* Ndoyako** male x **AgNosCd-1** female | Rep 2 | Parent | ***Black****/CFP-* | Male | WT |
| ND79 | OR694139 | ***An. gambiae* Ndoyako** male x **AgNosCd-1** female | Rep 2 | Parent | ***Black****/CFP-* | Male | WT |
| ND80 | OR694140 | ***An. gambiae* Ndoyako** male x **AgNosCd-1** female | Rep 2 | Parent | ***Black****/CFP-* | Male | WT |
|  | OR694141 |  |  |  |  |  |  |
| ND81 | OR694142 | ***An. gambiae* Ndoyako** male x **AgNosCd-1** female | Rep 2 | Parent | ***Black****/CFP-* | Male | WT |
| ND82 | OR694143 | ***An. gambiae* Ndoyako** male x **AgNosCd-1** female | Rep 2 | Parent | ***Black****/CFP-* | Male | WT |
| ND83 | OR694144 | ***An. gambiae* Ndoyako** male x **AgNosCd-1** female | Rep 2 | Parent | ***Black****/CFP-* | Male | WT |
| ND84 | OR694145 | ***An. gambiae* Ndoyako** male x **AgNosCd-1** female | Rep 2 | Parent | ***Black****/CFP-* | Male | WT |
|  | OR694146 |  |  |  |  |  |  |
| ND85 | OR694147 | ***An. gambiae* Ndoyako** male x **AgNosCd-1** female | Rep 2 | Parent | ***Black****/CFP-* | Male | WT |
|  | OR694148 |  |  |  |  |  |  |
| ND86 | OR694149 | ***An. gambiae* Ndoyako** male x **AgNosCd-1** female | Rep 2 | Parent | ***Black****/CFP-* | Male | WT |
|  | OR694150 |  |  |  |  |  |  |
| ND87 | OR694151 | ***An. gambiae* Ndoyako** male x **AgNosCd-1** female | Rep 2 | Parent | ***Black****/CFP-* | Male | WT |
| ND45 | OR694095 | **AgNosCd-1** male x ***An. gambiae* Ndoyako** female | Rep 2 | Parent | ***Black****/CFP-* | Female | WT |
| ND46 | OR694096 | **AgNosCd-1** male x ***An. gambiae* Ndoyako** female | Rep 2 | Parent | ***Black****/CFP-* | Female | WT |
|  | OR694097 |  |  |  |  |  |  |
| ND47 | OR694098 | **AgNosCd-1** male x ***An. gambiae* Ndoyako** female | Rep 2 | Parent | ***Black****/CFP-* | Female | WT |
| ND48 | OR694099 | **AgNosCd-1** male x ***An. gambiae* Ndoyako** female | Rep 2 | Parent | ***Black****/CFP-* | Female | WT |
| ND49 | OR694100 | **AgNosCd-1** male x ***An. gambiae* Ndoyako** female | Rep 2 | Parent | ***Black****/CFP-* | Female | WT |
|  | OR694101 |  |  |  |  |  |  |
| ND50 | OR694102 | **AgNosCd-1** male x ***An. gambiae* Ndoyako** female | Rep 2 | Parent | ***Black****/CFP-* | Female | WT |
| ND51 | OR694103 | **AgNosCd-1** male x ***An. gambiae* Ndoyako** female | Rep 2 | Parent | ***Black****/CFP-* | Female | WT |
| ND53 | OR694104 | **AgNosCd-1** male x ***An. gambiae* Ndoyako** female | Rep 2 | Parent | ***Black****/CFP-* | Female | WT |
| ND55 | OR694105 | **AgNosCd-1** male x ***An. gambiae* Ndoyako** female | Rep 2 | Parent | ***Black****/CFP-* | Female | WT |
|  | OR694106 |  |  |  |  |  |  |
| ND56 | OR694107 | **AgNosCd-1** male x ***An. gambiae* Ndoyako** female | Rep 2 | Parent | ***Black****/CFP-* | Female | WT |
| ND57 | OR694108 | **AgNosCd-1** male x ***An. gambiae* Ndoyako** female | Rep 2 | Parent | ***Black****/CFP-* | Female | WT |
|  | OR694109 |  |  |  |  |  |  |
| ND58 | OR694110 | **AgNosCd-1** male x ***An. gambiae* Ndoyako** female | Rep 2 | Parent | ***Black****/CFP-* | Female | WT |
| ND59 | OR694111 | **AgNosCd-1** male x ***An. gambiae* Ndoyako** female | Rep 2 | Parent | ***Black****/CFP-* | Female | WT |
|  | OR694112 |  |  |  |  |  |  |
| ND61 | OR694113 | **AgNosCd-1** male x ***An. gambiae* Ndoyako** female | Rep 2 | Parent | ***Black****/CFP-* | Female | WT |
| ND62 | OR694114 | **AgNosCd-1** male x ***An. gambiae* Ndoyako** female | Rep 2 | Parent | ***Black****/CFP-* | Female | WT |
| ND63 | OR694115 | **AgNosCd-1** male x ***An. gambiae* Ndoyako** female | Rep 2 | Parent | ***Black****/CFP-* | Female | WT |
| ND64 | OR694116 | **AgNosCd-1** male x ***An. gambiae* Ndoyako** female | Rep 2 | Parent | ***Black****/CFP-* | Female | WT |
| ND65 | OR694117 | **AgNosCd-1** male x ***An. gambiae* Ndoyako** female | Rep 2 | Parent | ***Black****/CFP-* | Female | WT |
| ND66 | OR694118 | **AgNosCd-1** male x ***An. gambiae* Ndoyako** female | Rep 2 | Parent | ***Black****/CFP-* | Female | WT |
|  | OR694119 |  |  |  |  |  |  |
| ND89 | OR694152 | **AgNosCd-1** male x ***An. gambiae* Ndoyako** female | Rep 2 | Parent | ***Black****/CFP-* | Female | WT |
|  | OR694153 |  |  |  |  |  |  |
| ND90 | OR694154 | **AgNosCd-1** male x ***An. gambiae* Ndoyako** female | Rep 2 | Parent | ***Black****/CFP-* | Female | WT |
|  | OR694155 |  |  |  |  |  |  |
| ND91 | OR694156 | **AgNosCd-1** male x ***An. gambiae* Ndoyako** female | Rep 2 | Parent | ***Black****/CFP-* | Female | WT |
|  | OR694157 |  |  |  |  |  | WT |
| ND151 | OR694260 | ***An. gambiae* Ndoyako** male x **AgNosCd-1** female | Rep 3 | Parent | ***Black****/CFP-* | Male | WT |
| ND152 | OR694261 | ***An. gambiae* Ndoyako** male x **AgNosCd-1** female | Rep 3 | Parent | ***Black****/CFP-* | Male | WT |
| ND153 | OR694262 | ***An. gambiae* Ndoyako** male x **AgNosCd-1** female | Rep 3 | Parent | ***Black****/CFP-* | Male | WT |
|  | OR694263 |  |  |  |  |  |  |
| ND154 | OR694264 | ***An. gambiae* Ndoyako** male x **AgNosCd-1** female | Rep 3 | Parent | ***Black****/CFP-* | Male | WT |
|  | OR694265 |  |  |  |  |  |  |
| ND155 | OR694266 | ***An. gambiae* Ndoyako** male x **AgNosCd-1** female | Rep 3 | Parent | ***Black****/CFP-* | Male | WT |
| ND156 | OR694267 | ***An. gambiae* Ndoyako** male x **AgNosCd-1** female | Rep 3 | Parent | ***Black****/CFP-* | Male | WT |
|  | OR694268 |  |  |  |  |  |  |
| ND157 | OR694269 | ***An. gambiae* Ndoyako** male x **AgNosCd-1** female | Rep 3 | Parent | ***Black****/CFP-* | Male | WT |
| ND158 | OR694270 | ***An. gambiae* Ndoyako** male x **AgNosCd-1** female | Rep 3 | Parent | ***Black****/CFP-* | Male | WT |
|  | OR694271 |  |  |  |  |  |  |
| ND159 | OR694272 | ***An. gambiae* Ndoyako** male x **AgNosCd-1** female | Rep 3 | Parent | ***Black****/CFP-* | Male | WT |
| ND160 | OR694273 | ***An. gambiae* Ndoyako** male x **AgNosCd-1** female | Rep 3 | Parent | ***Black****/CFP-* | Male | WT |
| ND161 | OR694274 | ***An. gambiae* Ndoyako** male x **AgNosCd-1** female | Rep 3 | Parent | ***Black****/CFP-* | Male | WT |
|  | OR694275 |  |  |  |  |  |  |
| ND162 | OR694276 | ***An. gambiae* Ndoyako** male x **AgNosCd-1** female | Rep 3 | Parent | ***Black****/CFP-* | Male | WT |
| ND163 | OR694277 | ***An. gambiae* Ndoyako** male x **AgNosCd-1** female | Rep 3 | Parent | ***Black****/CFP-* | Male | WT |
| ND164 | OR694278 | ***An. gambiae* Ndoyako** male x **AgNosCd-1** female | Rep 3 | Parent | ***Black****/CFP-* | Male | WT |
| ND165 | OR694279 | ***An. gambiae* Ndoyako** male x **AgNosCd-1** female | Rep 3 | Parent | ***Black****/CFP-* | Male | WT |
| ND166 | OR694280 | ***An. gambiae* Ndoyako** male x **AgNosCd-1** female | Rep 3 | Parent | ***Black****/CFP-* | Male | WT |
| ND167 | OR694281 | ***An. gambiae* Ndoyako** male x **AgNosCd-1** female | Rep 3 | Parent | ***Black****/CFP-* | Male | WT |
|  | OR694282 |  |  |  |  |  |  |
| ND168 | OR694283 | ***An. gambiae* Ndoyako** male x **AgNosCd-1** female | Rep 3 | Parent | ***Black****/CFP-* | Male | WT |
| ND169 | OR694284 | ***An. gambiae* Ndoyako** male x **AgNosCd-1** female | Rep 3 | Parent | ***Black****/CFP-* | Male | WT |
|  | OR694285 |  |  |  |  |  |  |
| ND170 | OR694286 | ***An. gambiae* Ndoyako** male x **AgNosCd-1** female | Rep 3 | Parent | ***Black****/CFP-* | Male | WT |
| ND171 | OR694287 | ***An. gambiae* Ndoyako** male x **AgNosCd-1** female | Rep 3 | Parent | ***Black****/CFP-* | Male | WT |
|  | OR694288 |  |  |  |  |  |  |
| ND172 | OR694289 | ***An. gambiae* Ndoyako** male x **AgNosCd-1** female | Rep 3 | Parent | ***Black****/CFP-* | Male | WT |
| ND174 | OR694290 | ***An. gambiae* Ndoyako** male x **AgNosCd-1** female | Rep 3 | Parent | ***Black****/CFP-* | Male | WT |
| ND175 | OR694291 | **AgNosCd-1** male x ***An. gambiae* Ndoyako** female | Rep 3 | Parent | ***Black****/CFP-* | Female | WT |
| ND176 | OR694292 | **AgNosCd-1** male x ***An. gambiae* Ndoyako** female | Rep 3 | Parent | ***Black****/CFP-* | Female | WT |
|  | OR694293 |  |  |  |  |  |  |
| ND177 | OR694294 | **AgNosCd-1** male x ***An. gambiae* Ndoyako** female | Rep 3 | Parent | ***Black****/CFP-* | Female | WT |
| ND178 | OR694295 | **AgNosCd-1** male x ***An. gambiae* Ndoyako** female | Rep 3 | Parent | ***Black****/CFP-* | Female | WT |
|  | OR694296 |  |  |  |  |  |  |
| ND179 | OR694297 | **AgNosCd-1** male x ***An. gambiae* Ndoyako** female | Rep 3 | Parent | ***Black****/CFP-* | Female | WT |
|  | OR694298 |  |  |  |  |  |  |
| ND180 | OR694299 | **AgNosCd-1** male x ***An. gambiae* Ndoyako** female | Rep 3 | Parent | ***Black****/CFP-* | Female | WT |
|  | OR694300 |  |  |  |  |  |  |
| ND181 | OR694301 | **AgNosCd-1** male x ***An. gambiae* Ndoyako** female | Rep 8 | Parent | ***Black****/CFP-* | Female | WT |
| ND182 | OR694302 | **AgNosCd-1** male x ***An. gambiae* Ndoyako** female | Rep 3 | Parent | ***Black****/CFP-* | Female | WT |
|  | OR694303 |  |  |  |  |  |  |
| ND183 | OR694304 | **AgNosCd-1** male x ***An. gambiae* Ndoyako** female | Rep 3 | Parent | ***Black****/CFP-* | Female | WT |
|  | OR694305 |  |  |  |  |  |  |
| ND184 | OR694306 | **AgNosCd-1** male x ***An. gambiae* Ndoyako** female | Rep 3 | Parent | ***Black****/CFP-* | Female | WT |
| ND185 | OR694307 | **AgNosCd-1** male x ***An. gambiae* Ndoyako** female | Rep 3 | Parent | ***Black****/CFP-* | Female | WT |
| ND186 | OR694308 | **AgNosCd-1** male x ***An. gambiae* Ndoyako** female | Rep 3 | Parent | ***Black****/CFP-* | Female | WT |
| ND187 | OR694309 | **AgNosCd-1** male x ***An. gambiae* Ndoyako** female | Rep 3 | Parent | ***Black****/CFP-* | Female | WT |
|  | OR694310 |  |  |  |  |  |  |
| ND188 | OR694311 | **AgNosCd-1** male x ***An. gambiae* Ndoyako** female | Rep 3 | Parent | ***Black****/CFP-* | Female | WT |
| ND189 | OR694312 | **AgNosCd-1** male x ***An. gambiae* Ndoyako** female | Rep 3 | Parent | ***Black****/CFP-* | Female | WT |
|  | OR694313 |  |  |  |  |  |  |
| ND190 | OR694314 | **AgNosCd-1** male x ***An. gambiae* Ndoyako** female | Rep 3 | Parent | ***Black****/CFP-* | Female | WT |
|  | OR694315 |  |  |  |  |  |  |
| ND191 | OR694316 | **AgNosCd-1** male x ***An. gambiae* Ndoyako** female | Rep 3 | Parent | ***Black****/CFP-* | Female | WT |
| ND192 | OR694317 | **AgNosCd-1** male x ***An. gambiae* Ndoyako** female | Rep 3 | Parent | ***Black****/CFP-* | Female | WT |
|  | OR694318 |  |  |  |  |  |  |
| ND193 | OR694319 | **AgNosCd-1** male x ***An. gambiae* Ndoyako** female | Rep 3 | Parent | ***Black****/CFP-* | Female | WT |
| ND194 | OR694320 | **AgNosCd-1** male x ***An. gambiae* Ndoyako** female | Rep 3 | Parent | ***Black****/CFP-* | Female | WT |
|  | OR694321 |  |  |  |  |  |  |
| ND195 | OR694322 | **AgNosCd-1** male x ***An. gambiae* Ndoyako** female | Rep 3 | Parent | ***Black****/CFP-* | Female | WT |
|  | OR694323 |  |  |  |  |  |  |
| ND196 | OR694324 | **AgNosCd-1** male x ***An. gambiae* Ndoyako** female | Rep 3 | Parent | ***Black****/CFP-* | Female | WT |
| ND197 | OR694325 | **AgNosCd-1** male x ***An. gambiae* Ndoyako** female | Rep 3 | Parent | ***Black****/CFP-* | Female | WT |
| ND198 | OR694326 | **AgNosCd-1** male x ***An. gambiae* Ndoyako** female | Rep 3 | Parent | ***Black****/CFP-* | Female | WT |
|  | OR694327 |  |  |  |  |  |  |
| ND93 | OR694158 | ***An. gambiae* Ndoyako** male x **AgNosCd-1** female | Rep 1 | F1 | *Cardinal/CFP+* | male | Drive/NHEJ |
| ND94 | OR694159 | ***An. gambiae* Ndoyako** male x **AgNosCd-1** female | Rep 1 | F1 | *Cardinal/CFP+* | male | Drive/NHEJ |
|  | OR694160 |  |  |  |  |  |  |
| ND95 | OR694161 | ***An. gambiae* Ndoyako** male x **AgNosCd-1** female | Rep 1 | F1 | *Cardinal/CFP+* | male | Drive/NHEJ |
|  | OR694162 |  |  |  |  |  |  |
| ND96 | OR694163 | ***An. gambiae* Ndoyako** male x **AgNosCd-1** female | Rep 1 | F1 | *Cardinal/CFP+* | male | Drive/NHEJ |
|  | OR694164 |  |  |  |  |  |  |
| ND97 | OR694165 | ***An. gambiae* Ndoyako** male x **AgNosCd-1** female | Rep 1 | F1 | *Cardinal/CFP+* | male | Drive/NHEJ |
|  | OR694166 |  |  |  |  |  |  |
| ND98 | OR694167 | ***An. gambiae* Ndoyako** male x **AgNosCd-1** female | Rep 1 | F1 | *Cardinal/CFP+* | male | Drive/NHEJ |
|  | OR694168 |  |  |  |  |  |  |
| ND99 | OR694169 | ***An. gambiae* Ndoyako** male x **AgNosCd-1** female | Rep 1 | F1 | *Cardinal/CFP+* | male | Drive/NHEJ |
|  | OR694170 |  |  |  |  |  |  |
| ND100 | OR694171 | ***An. gambiae* Ndoyako** male x **AgNosCd-1** female | Rep 1 | F1 | *Cardinal/CFP+* | male | Drive/NHEJ |
| ND101 | OR694172 | ***An. gambiae* Ndoyako** male x **AgNosCd-1** female | Rep 1 | F1 | *Cardinal/CFP+* | male | Drive/NHEJ |
| ND102 | OR694173 | ***An. gambiae* Ndoyako** male x **AgNosCd-1** female | Rep 1 | F1 | *Cardinal/CFP+* | female | Drive/NHEJ |
|  | OR694174 |  |  |  |  |  |  |
| ND103 | OR694175 | ***An. gambiae* Ndoyako** male x **AgNosCd-1** female | Rep 1 | F1 | *Cardinal/CFP+* | female | Drive/NHEJ |
| ND104 | OR694176 | ***An. gambiae* Ndoyako** male x **AgNosCd-1** female | Rep 1 | F1 | *Cardinal/CFP+* | female | Drive/NHEJ |
|  | OR694177 |  |  |  |  |  |  |
| ND105 | OR694178 | ***An. gambiae* Ndoyako** male x **AgNosCd-1** female | Rep 1 | F1 | *Cardinal/CFP+* | female | Drive/NHEJ |
|  | OR694179 |  |  |  |  |  |  |
| ND106 | OR694180 | ***An. gambiae* Ndoyako** male x **AgNosCd-1** female | Rep 1 | F1 | *Cardinal/CFP+* | female | Drive/NHEJ |
|  | OR694181 |  |  |  |  |  |  |
| ND107 | OR694182 | ***An. gambiae* Ndoyako** male x **AgNosCd-1** female | Rep 1 | F1 | *Cardinal/CFP+* | female | Drive/NHEJ |
|  | OR694183 |  |  |  |  |  |  |
| ND108 | OR694184 | ***An. gambiae* Ndoyako** male x **AgNosCd-1** female | Rep 1 | F1 | *Cardinal/CFP+* | female | Drive/NHEJ |
|  | OR694185 |  |  |  |  |  |  |
| ND109 | OR694186 | ***An. gambiae* Ndoyako** male x **AgNosCd-1** female | Rep 1 | F1 | *Cardinal/CFP+* | female | Drive/NHEJ |
|  | OR694187 |  |  |  |  |  |  |
| ND110 | OR694188 | ***An. gambiae* Ndoyako** male x **AgNosCd-1** female | Rep 1 | F1 | *Cardinal/CFP+* | female | Drive/NHEJ |
|  | OR694189 |  |  |  |  |  |  |
| ND111 | OR694190 | ***An. gambiae* Ndoyako** male x **AgNosCd-1** female | Rep 1 | F1 | *Cardinal/CFP+* | female | Drive/NHEJ |
|  | OR694191 |  |  |  |  |  |  |
| ND112 | OR694192 | ***An. gambiae* Ndoyako** male x **AgNosCd-1** female | Rep 1 | F1 | *Cardinal/CFP+* | female | Drive/NHEJ |
|  | OR694193 |  |  |  |  |  |  |
| ND113 | OR694194 | ***An. gambiae* Ndoyako** male x **AgNosCd-1** female | Rep 2 | F1 | *Cardinal/CFP+* | female | Drive/NHEJ |
|  | OR694195 |  |  |  |  |  |  |
| ND114 | OR694196 | ***An. gambiae* Ndoyako** male x **AgNosCd-1** female | Rep 2 | F1 | *Cardinal/CFP+* | female | Drive/NHEJ |
| ND115 | OR694197 | ***An. gambiae* Ndoyako** male x **AgNosCd-1** female | Rep 2 | F1 | *Cardinal/CFP+* | female | Drive/NHEJ |
|  | OR694198 |  |  |  |  |  |  |
| ND116 | OR694199 | ***An. gambiae* Ndoyako** male x **AgNosCd-1** female | Rep 2 | F1 | *Cardinal/CFP+* | female | Drive/NHEJ |
| ND117 | OR694200 | ***An. gambiae* Ndoyako** male x **AgNosCd-1** female | Rep 2 | F1 | *Cardinal/CFP+* | female | Drive/NHEJ |
| ND118 | OR694201 | ***An. gambiae* Ndoyako** male x **AgNosCd-1** female | Rep 2 | F1 | *Cardinal/CFP+* | female | Drive/NHEJ |
|  | OR694202 |  |  |  |  |  |  |
| ND119 | OR694203 | ***An. gambiae* Ndoyako** male x **AgNosCd-1** female | Rep 2 | F1 | *Cardinal/CFP+* | female | Drive/NHEJ |
|  | OR694204 |  |  |  |  |  |  |
| ND120 | OR694205 | ***An. gambiae* Ndoyako** male x **AgNosCd-1** female | Rep 2 | F1 | *Cardinal/CFP+* | female | Drive/NHEJ |
|  | OR694206 |  |  |  |  |  |  |
| ND121 | OR694207 | ***An. gambiae* Ndoyako** male x **AgNosCd-1** female | Rep 2 | F1 | *Cardinal/CFP+* | female | Drive/NHEJ |
|  | OR694208 |  |  |  |  |  |  |
| ND122 | OR694209 | ***An. gambiae* Ndoyako** male x **AgNosCd-1** female | Rep 2 | F1 | *Cardinal/CFP+* | female | Drive/NHEJ |
|  | OR694210 |  |  |  |  |  |  |
| ND123 | OR694211 | ***An. gambiae* Ndoyako** male x **AgNosCd-1** female | Rep 2 | F1 | *Cardinal/CFP+* | female | Drive/NHEJ |
|  | OR694212 |  |  |  |  |  |  |
| ND124 | OR694213 | ***An. gambiae* Ndoyako** male x **AgNosCd-1** female | Rep 2 | F1 | *Cardinal/CFP+* | female | Drive/NHEJ |
|  | OR694214 |  |  |  |  |  |  |
| ND125 | OR694215 | ***An. gambiae* Ndoyako** male x **AgNosCd-1** female | Rep 2 | F1 | *Cardinal/CFP+* | female | Drive/NHEJ |
|  | OR694216 |  |  |  |  |  |  |
| ND126 | OR694217 | ***An. gambiae* Ndoyako** male x **AgNosCd-1** female | Rep 2 | F1 | *Cardinal/CFP+* | male | Drive/NHEJ |
|  | OR694218 |  |  |  |  |  |  |
| ND127 | OR694219 | ***An. gambiae* Ndoyako** male x **AgNosCd-1** female | Rep 2 | F1 | *Cardinal/CFP+* | male | Drive/NHEJ |
| ND128 | OR694220 | ***An. gambiae* Ndoyako** male x **AgNosCd-1** female | Rep 2 | F1 | *Cardinal/CFP+* | male | Drive/NHEJ |
| ND129 | OR694221 | ***An. gambiae* Ndoyako** male x **AgNosCd-1** female | Rep 2 | F1 | *Cardinal/CFP+* | male | Drive/NHEJ |
| ND130 | OR694222 | ***An. gambiae* Ndoyako** male x **AgNosCd-1** female | Rep 2 | F1 | *Cardinal/CFP+* | male | Drive/NHEJ |
|  | OR694223 |  |  |  |  |  |  |
| ND131 | OR694224 | ***An. gambiae* Ndoyako** male x **AgNosCd-1** female | Rep 2 | F1 | *Cardinal/CFP+* | male | Drive/NHEJ |
|  | OR694225 |  |  |  |  |  |  |
| ND237 | OR694352 | ***An. gambiae* Ndoyako** male x **AgNosCd-1** female | Rep 3 | F1 | *Cardinal/CFP+* | male | Drive/NHEJ |
| ND238 | OR694353 | ***An. gambiae* Ndoyako** male x **AgNosCd-1** female | Rep 3 | F1 | *Cardinal/CFP+* | male | Drive/NHEJ |
| ND239 | OR694354 | ***An. gambiae* Ndoyako** male x **AgNosCd-1** female | Rep 3 | F1 | *Cardinal/CFP+* | male | Drive/NHEJ |
| ND240 | OR694355 | ***An. gambiae* Ndoyako** male x **AgNosCd-1** female | Rep 3 | F1 | *Cardinal/CFP+* | male | Drive/NHEJ |
|  | OR694356 |  |  |  |  |  |  |
| ND241 | OR694357 | ***An. gambiae* Ndoyako** male x **AgNosCd-1** female | Rep 3 | F1 | *Cardinal/CFP+* | female | Drive/NHEJ |
|  | OR694358 |  |  |  |  |  |  |
| ND242 | OR694359 | ***An. gambiae* Ndoyako** male x **AgNosCd-1** female | Rep 3 | F1 | *Cardinal/CFP+* | female | Drive/NHEJ |
|  | OR694360 |  |  |  |  |  |  |
| ND243 | OR694361 | ***An. gambiae* Ndoyako** male x **AgNosCd-1** female | Rep 3 | F1 | *Cardinal/CFP+* | female | Drive/NHEJ |
|  | OR694362 |  |  |  |  |  |  |
| ND244 | OR694363 | ***An. gambiae* Ndoyako** male x **AgNosCd-1** female | Rep 3 | F1 | *Cardinal/CFP+* | female | Drive/NHEJ |
|  | OR694364 |  |  |  |  |  |  |
| ND421 | OR694420 | ***An. gambiae* Ndoyako** male x **AgNosCd-1** female | Rep1 | F1 | ***Black****/****CFP+*** | Male | Drive/NHEJ |
| ND422 | OR694421 | ***An. gambiae* Ndoyako** male x **AgNosCd-1** female | Rep1 | F1 | ***Black****/****CFP+*** | Male | Drive/WT |
| ND423 | OR694422 | ***An. gambiae* Ndoyako** male x **AgNosCd-1** female | Rep1 | F1 | ***Black****/****CFP+*** | Male | Drive/WT |
| ND424 | OR694423 | ***An. gambiae* Ndoyako** male x **AgNosCd-1** female | Rep1 | F1 | ***Black****/****CFP+*** | Male | Drive/WT |
| ND425 | OR694424 | ***An. gambiae* Ndoyako** male x **AgNosCd-1** female | Rep1 | F1 | ***Black****/****CFP+*** | Male | Drive/WT |
| ND426 | OR694425 | ***An. gambiae* Ndoyako** male x **AgNosCd-1** female | Rep1 | F1 | ***Black****/****CFP+*** | Male | Drive/WT |
| ND427 | OR694426 | ***An. gambiae* Ndoyako** male x **AgNosCd-1** female | Rep1 | F1 | ***Black****/****CFP+*** | Male | Drive/NHEJ |
|  | OR694427 |  |  |  |  |  |  |
| ND428 | OR694428 | ***An. gambiae* Ndoyako** male x **AgNosCd-1** female | Rep1 | F1 | ***Black****/****CFP+*** | Male | Drive/WT |
| ND429 | OR694429 | ***An. gambiae* Ndoyako** male x **AgNosCd-1** female | Rep1 | F1 | ***Black****/****CFP+*** | Male | Drive/WT |
| ND430 | OR694430 | ***An. gambiae* Ndoyako** male x **AgNosCd-1** female | Rep1 | F1 | ***Black****/****CFP+*** | Male | Drive/WT |
| ND431 | OR694431 | ***An. gambiae* Ndoyako** male x **AgNosCd-1** female | Rep1 | F1 | ***Black****/****CFP+*** | Female | Drive/WT |
| ND432 | OR694432 | ***An. gambiae* Ndoyako** male x **AgNosCd-1** female | Rep1 | F1 | ***Black****/****CFP+*** | Female | Drive/WT |
| ND433 | OR694433 | ***An. gambiae* Ndoyako** male x **AgNosCd-1** female | Rep1 | F1 | ***Black****/****CFP+*** | Female | Drive/WT |
| ND434 | OR694434 | ***An. gambiae* Ndoyako** male x **AgNosCd-1** female | Rep1 | F1 | ***Black****/****CFP+*** | Female | Drive/WT |
| ND435 | OR694435 | ***An. gambiae* Ndoyako** male x **AgNosCd-1** female | Rep1 | F1 | ***Black****/****CFP+*** | Female | Drive/WT |
| ND436 | OR694436 | ***An. gambiae* Ndoyako** male x **AgNosCd-1** female | Rep1 | F1 | ***Black****/****CFP+*** | Female | Drive/WT |
| ND437 | OR694437 | ***An. gambiae* Ndoyako** male x **AgNosCd-1** female | Rep1 | F1 | ***Black****/****CFP+*** | Female | Drive/WT |
| ND438 | OR694438 | ***An. gambiae* Ndoyako** male x **AgNosCd-1** female | Rep1 | F1 | ***Black****/****CFP+*** | Female | Drive/WT |
| ND439 | OR694439 | ***An. gambiae* Ndoyako** male x **AgNosCd-1** female | Rep1 | F1 | ***Black****/****CFP+*** | Female | Drive/WT |
| ND440 | OR694440 | ***An. gambiae* Ndoyako** male x **AgNosCd-1** female | Rep1 | F1 | ***Black****/****CFP+*** | Female | Drive/WT |
| ND441 | OR694441 | ***An. gambiae* Ndoyako** male x **AgNosCd-1** female | Rep3 | F1 | ***Black****/****CFP+*** | Male | Drive/WT |
| ND442 | OR694442 | ***An. gambiae* Ndoyako** male x **AgNosCd-1** female | Rep3 | F1 | ***Black****/****CFP+*** | Male | Drive/NHEJ |
|  | OR694443 |  |  |  |  |  |  |
| ND443 | OR694444 | ***An. gambiae* Ndoyako** male x **AgNosCd-1** female | Rep3 | F1 | ***Black****/****CFP+*** | Male | Drive/WT |
| ND444 | OR694445 | ***An. gambiae* Ndoyako** male x **AgNosCd-1** female | Rep3 | F1 | ***Black****/****CFP+*** | Male | Drive/WT |
| ND445 | OR694446 | ***An. gambiae* Ndoyako** male x **AgNosCd-1** female | Rep3 | F1 | ***Black****/****CFP+*** | Male | Drive/WT |
| ND446 | OR694447 | ***An. gambiae* Ndoyako** male x **AgNosCd-1** female | Rep3 | F1 | ***Black****/****CFP+*** | Male | Drive/NHEJ |
|  | OR694448 |  |  |  |  |  |  |
| ND447 | OR694449 | ***An. gambiae* Ndoyako** male x **AgNosCd-1** female | Rep3 | F1 | ***Black****/****CFP+*** | Male | Drive/WT |
| ND448 | OR694450 | ***An. gambiae* Ndoyako** male x **AgNosCd-1** female | Rep3 | F1 | ***Black****/****CFP+*** | Male | Drive/WT/NHEJ |
|  | OR694451 |  |  |  |  |  |  |
| ND449 | OR694452 | ***An. gambiae* Ndoyako** male x **AgNosCd-1** female | Rep3 | F1 | ***Black****/****CFP+*** | Male | Drive/WT |
| ND450 | OR694453 | ***An. gambiae* Ndoyako** male x **AgNosCd-1** female | Rep3 | F1 | ***Black****/****CFP+*** | Male | Drive/WT |
| ND451 | OR694454 | ***An. gambiae* Ndoyako** male x **AgNosCd-1** female | Rep3 | F1 | ***Black****/****CFP+*** | Female | Drive/WT |
| ND452 | OR694455 | ***An. gambiae* Ndoyako** male x **AgNosCd-1** female | Rep3 | F1 | ***Black****/****CFP+*** | Female | Drive/NHEJ |
|  | OR694456 |  |  |  |  |  |  |
| ND453 | OR694457 | ***An. gambiae* Ndoyako** male x **AgNosCd-1** female | Rep3 | F1 | ***Black****/****CFP+*** | Female | Drive/WT |
| ND454 | OR694458 | ***An. gambiae* Ndoyako** male x **AgNosCd-1** female | Rep3 | F1 | ***Black****/****CFP+*** | Female | Drive/WT |
| ND455 | OR694459 | ***An. gambiae* Ndoyako** male x **AgNosCd-1** female | Rep3 | F1 | ***Black****/****CFP+*** | Female | Drive/NHEJ |
| ND456 | OR694460 | ***An. gambiae* Ndoyako** male x **AgNosCd-1** female | Rep3 | F1 | ***Black****/****CFP+*** | Female | Drive/NHEJ |
| ND457 | OR694461 | ***An. gambiae* Ndoyako** male x **AgNosCd-1** female | Rep3 | F1 | ***Black****/****CFP+*** | Female | Drive/WT |
| ND458 | OR694462 | ***An. gambiae* Ndoyako** male x **AgNosCd-1** female | Rep3 | F1 | ***Black****/****CFP+*** | Female | Drive/WT |
| ND459 | OR694463 | ***An. gambiae* Ndoyako** male x **AgNosCd-1** female | Rep3 | F1 | ***Black****/****CFP+*** | Female | Drive/WT |
| ND460 | OR694464 | ***An. gambiae* Ndoyako** male x **AgNosCd-1** female | Rep3 | F1 | ***Black****/****CFP+*** | Female | Drive/NHEJ |
|  | OR694465 |  |  |  |  |  |  |
| ND461 | OR694466 | **AgNosCd-1** male x ***An. gambiae* Ndoyako** female | Rep1 | F1 | ***Black****/****CFP+*** | Male | Drive/WT |
| ND462 | OR694467 | **AgNosCd-1** male x ***An. gambiae* Ndoyako** female | Rep1 | F1 | ***Black****/****CFP+*** | Male | Drive/WT |
| ND463 | OR694468 | **AgNosCd-1** male x ***An. gambiae* Ndoyako** female | Rep1 | F1 | ***Black****/****CFP+*** | Male | Drive/WT |
| ND464 | OR694469 | **AgNosCd-1** male x ***An. gambiae* Ndoyako** female | Rep1 | F1 | ***Black****/****CFP+*** | Male | Drive/WT |
| ND465 | OR694470 | **AgNosCd-1** male x ***An. gambiae* Ndoyako** female | Rep1 | F1 | ***Black****/****CFP+*** | Male | Drive/WT |
| ND466 | OR694471 | **AgNosCd-1** male x ***An. gambiae* Ndoyako** female | Rep1 | F1 | ***Black****/****CFP+*** | Male | Drive/WT |
| ND467 | OR694472 | **AgNosCd-1** male x ***An. gambiae* Ndoyako** female | Rep1 | F1 | ***Black****/****CFP+*** | Male | Drive/WT |
| ND468 | OR694473 | **AgNosCd-1** male x ***An. gambiae* Ndoyako** female | Rep1 | F1 | ***Black****/****CFP+*** | Male | Drive/WT |
| ND469 | OR694474 | **AgNosCd-1** male x ***An. gambiae* Ndoyako** female | Rep1 | F1 | ***Black****/****CFP+*** | Male | Drive/WT |
| ND470 | OR694475 | **AgNosCd-1** male x ***An. gambiae* Ndoyako** female | Rep1 | F1 | ***Black****/****CFP+*** | Male | Drive/WT |
| ND471 | OR694476 | **AgNosCd-1** male x ***An. gambiae* Ndoyako** female | Rep1 | F1 | ***Black****/****CFP+*** | Female | Drive/WT |
| ND472 | OR694477 | **AgNosCd-1** male x ***An. gambiae* Ndoyako** female | Rep1 | F1 | ***Black****/****CFP+*** | Female | Drive/WT |
| ND473 | OR694478 | **AgNosCd-1** male x ***An. gambiae* Ndoyako** female | Rep1 | F1 | ***Black****/****CFP+*** | Female | Drive/WT |
| ND474 | OR694479 | **AgNosCd-1** male x ***An. gambiae* Ndoyako** female | Rep1 | F1 | ***Black****/****CFP+*** | Female | Drive/WT |
| ND475 | OR694480 | **AgNosCd-1** male x ***An. gambiae* Ndoyako** female | Rep1 | F1 | ***Black****/****CFP+*** | Female | Drive/WT |
| ND476 | OR694481 | **AgNosCd-1** male x ***An. gambiae* Ndoyako** female | Rep1 | F1 | ***Black****/****CFP+*** | Female | Drive/WT |
| ND477 | OR694482 | **AgNosCd-1** male x ***An. gambiae* Ndoyako** female | Rep1 | F1 | ***Black****/****CFP+*** | Female | Drive/WT |
| ND478 | OR694483 | **AgNosCd-1** male x ***An. gambiae* Ndoyako** female | Rep1 | F1 | ***Black****/****CFP+*** | Female | Drive/WT |
| ND479 | OR694484 | **AgNosCd-1** male x ***An. gambiae* Ndoyako** female | Rep1 | F1 | ***Black****/****CFP+*** | Female | Drive/WT |
| ND480 | OR694485 | **AgNosCd-1** male x ***An. gambiae* Ndoyako** female | Rep1 | F1 | ***Black****/****CFP+*** | Female | Drive/WT |
| ND481 | OR694486 | **AgNosCd-1** male x ***An. gambiae* Ndoyako** female | Rep2 | F1 | ***Black****/****CFP+*** | Male | Drive/WT |
| ND482 | OR694487 | **AgNosCd-1** male x ***An. gambiae* Ndoyako** female | Rep2 | F1 | ***Black****/****CFP+*** | Male | Drive/WT |
| ND483 | OR694488 | **AgNosCd-1** male x ***An. gambiae* Ndoyako** female | Rep2 | F1 | ***Black****/****CFP+*** | Male | Drive/WT |
| ND484 | OR694489 | **AgNosCd-1** male x ***An. gambiae* Ndoyako** female | Rep2 | F1 | ***Black****/****CFP+*** | Male | Drive/WT |
| ND485 | OR694490 | **AgNosCd-1** male x ***An. gambiae* Ndoyako** female | Rep2 | F1 | ***Black****/****CFP+*** | Male | Drive/WT |
| ND486 | OR694491 | **AgNosCd-1** male x ***An. gambiae* Ndoyako** female | Rep2 | F1 | ***Black****/****CFP+*** | Male | Drive/WT |
| ND487 | OR694492 | **AgNosCd-1** male x ***An. gambiae* Ndoyako** female | Rep2 | F1 | ***Black****/****CFP+*** | Male | Drive/WT |
| ND488 | OR694493 | **AgNosCd-1** male x ***An. gambiae* Ndoyako** female | Rep2 | F1 | ***Black****/****CFP+*** | Male | Drive/WT |
| ND489 | OR694494 | **AgNosCd-1** male x ***An. gambiae* Ndoyako** female | Rep2 | F1 | ***Black****/****CFP+*** | Male | Drive/WT |
| ND490 | OR694495 | **AgNosCd-1** male x ***An. gambiae* Ndoyako** female | Rep2 | F1 | ***Black****/****CFP+*** | Male | Drive/WT |
| ND491 | OR694496 | **AgNosCd-1** male x ***An. gambiae* Ndoyako** female | Rep2 | F1 | ***Black****/****CFP+*** | Female | Drive/WT |
| ND492 | OR694497 | **AgNosCd-1** male x ***An. gambiae* Ndoyako** female | Rep2 | F1 | ***Black****/****CFP+*** | Female | Drive/WT |
| ND493 | OR694498 | **AgNosCd-1** male x ***An. gambiae* Ndoyako** female | Rep2 | F1 | ***Black****/****CFP+*** | Female | Drive/WT |
| ND494 | OR694499 | **AgNosCd-1** male x ***An. gambiae* Ndoyako** female | Rep2 | F1 | ***Black****/****CFP+*** | Female | Drive/WT |
| ND495 | OR694500 | **AgNosCd-1** male x ***An. gambiae* Ndoyako** female | Rep2 | F1 | ***Black****/****CFP+*** | Female | Drive/WT |
| ND496 | OR694501 | **AgNosCd-1** male x ***An. gambiae* Ndoyako** female | Rep2 | F1 | ***Black****/****CFP+*** | Female | Drive/WT |
| ND497 | OR694502 | **AgNosCd-1** male x ***An. gambiae* Ndoyako** female | Rep2 | F1 | ***Black****/****CFP+*** | Female | Drive/WT |
| ND498 | OR694503 | **AgNosCd-1** male x ***An. gambiae* Ndoyako** female | Rep2 | F1 | ***Black****/****CFP+*** | Female | Drive/WT |
| ND499 | OR694504 | **AgNosCd-1** male x ***An. gambiae* Ndoyako** female | Rep2 | F1 | ***Black****/****CFP+*** | Female | Drive/WT |
| ND500 | OR694505 | **AgNosCd-1** male x ***An. gambiae* Ndoyako** female | Rep2 | F1 | ***Black****/****CFP+*** | Female | Drive/WT |
| ND501 | OR694506 | **AgNosCd-1** male x ***An. gambiae* Ndoyako** female | Rep3 | F1 | ***Black****/****CFP+*** | Male | Drive/WT |
| ND502 | OR694507 | **AgNosCd-1** male x ***An. gambiae* Ndoyako** female | Rep3 | F1 | ***Black****/****CFP+*** | Male | Drive/WT |
| ND503 | OR694508 | **AgNosCd-1** male x ***An. gambiae* Ndoyako** female | Rep3 | F1 | ***Black****/****CFP+*** | Male | Drive/WT |
| ND504 | OR694509 | **AgNosCd-1** male x ***An. gambiae* Ndoyako** female | Rep3 | F1 | ***Black****/****CFP+*** | Male | Drive/WT |
| ND505 | OR694510 | **AgNosCd-1** male x ***An. gambiae* Ndoyako** female | Rep3 | F1 | ***Black****/****CFP+*** | Male | Drive/WT |
| ND506 | OR694511 | **AgNosCd-1** male x ***An. gambiae* Ndoyako** female | Rep3 | F1 | ***Black****/****CFP+*** | Male | Drive/WT |
| ND507 | OR694512 | **AgNosCd-1** male x ***An. gambiae* Ndoyako** female | Rep3 | F1 | ***Black****/****CFP+*** | Male | Drive/WT |
| ND508 | OR694513 | **AgNosCd-1** male x ***An. gambiae* Ndoyako** female | Rep3 | F1 | ***Black****/****CFP+*** | Male | Drive/WT |
| ND509 | OR694514 | **AgNosCd-1** male x ***An. gambiae* Ndoyako** female | Rep3 | F1 | ***Black****/****CFP+*** | Male | Drive/WT |
| ND510 | OR694515 | **AgNosCd-1** male x ***An. gambiae* Ndoyako** female | Rep3 | F1 | ***Black****/****CFP+*** | Male | Drive/WT |
| ND511 | OR694516 | **AgNosCd-1** male x ***An. gambiae* Ndoyako** female | Rep3 | F1 | ***Black****/****CFP+*** | Female | Drive/WT |
| ND512 | OR694517 | **AgNosCd-1** male x ***An. gambiae* Ndoyako** female | Rep3 | F1 | ***Black****/****CFP+*** | Female | Drive/WT |
| ND513 | OR694518 | **AgNosCd-1** male x ***An. gambiae* Ndoyako** female | Rep3 | F1 | ***Black****/****CFP+*** | Female | Drive/WT |
| ND514 | OR694519 | **AgNosCd-1** male x ***An. gambiae* Ndoyako** female | Rep3 | F1 | ***Black****/****CFP+*** | Female | Drive/WT |
| ND515 | OR694520 | **AgNosCd-1** male x ***An. gambiae* Ndoyako** female | Rep3 | F1 | ***Black****/****CFP+*** | Female | Drive/WT |
| ND516 | OR694521 | **AgNosCd-1** male x ***An. gambiae* Ndoyako** female | Rep3 | F1 | ***Black****/****CFP+*** | Female | Drive/WT |
| ND517 | OR694522 | **AgNosCd-1** male x ***An. gambiae* Ndoyako** female | Rep3 | F1 | ***Black****/****CFP+*** | Female | Drive/WT |
| ND518 | OR694523 | **AgNosCd-1** male x ***An. gambiae* Ndoyako** female | Rep3 | F1 | ***Black****/****CFP+*** | Female | Drive/WT |
| ND519 | OR694524 | **AgNosCd-1** male x ***An. gambiae* Ndoyako** female | Rep3 | F1 | ***Black****/****CFP+*** | Female | Drive/WT |
| ND520 | OR694525 | **AgNosCd-1** male x ***An. gambiae* Ndoyako** female | Rep3 | F1 | ***Black****/****CFP+*** | Female | Drive/WT |
| ND199 | OR694328 | **AgNosCd-1** male/***An. gambiae* Ndoyako** female hybrid intercross | Rep 1 | F2 | ***Black****/****CFP+*** | Male | Drive/WT |
| ND200 | OR694329 | **AgNosCd-1** male/***An. gambiae* Ndoyako** female hybrid intercross | Rep 1 | F2 | ***Black****/****CFP+*** | Male | Drive/WT |
| ND201 | OR694330 | **AgNosCd-1** male/***An. gambiae* Ndoyako** female hybrid intercross | Rep 1 | F2 | ***Black****/****CFP+*** | Male | Drive/WT |
| ND202 | OR694331 | **AgNosCd-1** male/***An. gambiae* Ndoyako** female hybrid intercross | Rep 1 | F2 | ***Black****/****CFP+*** | Male | Drive/WT |
| ND203 | OR694332 | **AgNosCd-1** male/***An. gambiae* Ndoyako** female hybrid intercross | Rep 1 | F2 | ***Black****/****CFP+*** | Female | Drive/WT |
| ND204 | OR694333 | **AgNosCd-1** male/***An. gambiae* Ndoyako** female hybrid intercross | Rep 1 | F2 | ***Black****/****CFP+*** | Female | Drive/WT |
| ND205 | OR694334 | **AgNosCd-1** male/***An. gambiae* Ndoyako** female hybrid intercross | Rep 1 | F2 | ***Black****/****CFP+*** | Female | Drive/WT |
| ND206 | OR694335 | **AgNosCd-1** male/***An. gambiae* Ndoyako** female hybrid intercross | Rep 1 | F2 | ***Black****/****CFP+*** | Female | Drive/WT |
| ND257 | OR694393 | **AgNosCd-1** male/***An. gambiae* Ndoyako** female hybrid intercross | Rep 2 | F2 | ***Black****/****CFP+*** | Female | Drive/WT |
| ND258 | OR694394 | **AgNosCd-1** male/***An. gambiae* Ndoyako** female hybrid intercross | Rep 2 | F2 | ***Black****/****CFP+*** | Female | Drive/WT |
| ND259 | OR694395 | **AgNosCd-1** male/***An. gambiae* Ndoyako** female hybrid intercross | Rep 2 | F2 | ***Black****/****CFP+*** | Male | Drive/WT |
| ND260 | OR694396 | **AgNosCd-1** male/***An. gambiae* Ndoyako** female hybrid intercross | Rep 2 | F2 | ***Black****/****CFP+*** | Male | Drive/WT |
| ND261 | OR694397 | **AgNosCd-1** male/***An. gambiae* Ndoyako** female hybrid intercross | Rep 2 | F2 | ***Black****/****CFP+*** | Male | Drive/WT |
| ND225 | OR694340 | **AgNosCd-1** male/***An. gambiae* Ndoyako** female hybrid intercross | Rep 3 | F2 | ***Black****/****CFP+*** | Male | Drive/WT |
| ND226 | OR694341 | **AgNosCd-1** male/***An. gambiae* Ndoyako** female hybrid intercross | Rep 3 | F2 | ***Black****/****CFP+*** | Male | Drive/WT |
| ND227 | OR694342 | **AgNosCd-1** male/***An. gambiae* Ndoyako** female hybrid intercross | Rep 3 | F2 | ***Black****/****CFP+*** | Male | Drive/WT |
| ND228 | OR694343 | **AgNosCd-1** male/***An. gambiae* Ndoyako** female hybrid intercross | Rep 3 | F2 | ***Black****/****CFP+*** | Male | Drive/WT |
| ND229 | OR694344 | **AgNosCd-1** male/***An. gambiae* Ndoyako** female hybrid intercross | Rep 3 | F2 | ***Black****/****CFP+*** | Male | Drive/WT |
| ND230 | OR694345 | **AgNosCd-1** male/***An. gambiae* Ndoyako** female hybrid intercross | Rep 3 | F2 | ***Black****/****CFP+*** | Male | Drive/WT |
| ND231 | OR694346 | **AgNosCd-1** male/***An. gambiae* Ndoyako** female hybrid intercross | Rep 3 | F2 | ***Black****/****CFP+*** | Female | Drive/WT |
| ND232 | OR694347 | **AgNosCd-1** male/***An. gambiae* Ndoyako** female hybrid intercross | Rep 3 | F2 | ***Black****/****CFP+*** | Female | Drive/WT |
| ND233 | OR694348 | **AgNosCd-1** male/***An. gambiae* Ndoyako** female hybrid intercross | Rep 3 | F2 | ***Black****/****CFP+*** | Female | Drive/WT |
| ND234 | OR694349 | **AgNosCd-1** male/***An. gambiae* Ndoyako** female hybrid intercross | Rep 3 | F2 | ***Black****/****CFP+*** | Female | Drive/WT |
| ND235 | OR694350 | **AgNosCd-1** male/***An. gambiae* Ndoyako** female hybrid intercross | Rep 3 | F2 | ***Black****/****CFP+*** | Female | Drive/WT |
| ND236 | OR694351 | **AgNosCd-1** male/***An. gambiae* Ndoyako** female hybrid intercross | Rep 3 | F2 | ***Black****/****CFP+*** | Female | Drive/WT |
| ND207 | OR694336 | ***An. gambiae* Ndoyako** male/**AgNosCd-1** female hybrid intercross | Rep 1 | F2 | ***Black****/****CFP+*** | Male | Drive/NHEJ |
| ND208 | OR694337 | ***An. gambiae* Ndoyako** male/**AgNosCd-1** female hybrid intercross | Rep 1 | F2 | ***Black****/****CFP+*** | Male | Drive/NHEJ |
| ND209 | OR694338 | ***An. gambiae* Ndoyako** male/**AgNosCd-1** female hybrid intercross | Rep 1 | F2 | ***Black****/****CFP+*** | Female | Drive/WT |
| ND210 | OR694339 | ***An. gambiae* Ndoyako** male/**AgNosCd-1** female hybrid intercross | Rep 1 | F2 | ***Black****/****CFP+*** | Female | Drive/NHEJ |
| ND262 | OR694398 | ***An. gambiae* Ndoyako** male/**AgNosCd-1** female hybrid intercross | Rep 2 | F2 | ***Black****/****CFP+*** | Male | Drive/NHEJ |
| ND263 | OR694399 | ***An. gambiae* Ndoyako** male/**AgNosCd-1** female hybrid intercross | Rep 2 | F2 | ***Black****/****CFP+*** | Male | Drive/NHEJ |
| ND264 | OR694400 | ***An. gambiae* Ndoyako** male/**AgNosCd-1** female hybrid intercross | Rep 2 | F2 | ***Black****/****CFP+*** | Female | Drive/NHEJ |
| ND265 | OR694401 | ***An. gambiae* Ndoyako** male/**AgNosCd-1** female hybrid intercross | Rep 2 | F2 | ***Black****/****CFP+*** | Female | Drive/NHEJ |
| ND266 | OR694402 | ***An. gambiae* Ndoyako** male/**AgNosCd-1** female hybrid intercross | Rep 2 | F2 | ***Black****/****CFP+*** | Female | Drive/NHEJ |
| ND251 | OR694387 | ***An. gambiae* Ndoyako** male/**AgNosCd-1** female hybrid intercross | Rep 3 | F2 | ***Black****/****CFP+*** | Male | Drive/NHEJ |
| ND252 | OR694388 | ***An. gambiae* Ndoyako** male/**AgNosCd-1** female hybrid intercross | Rep 3 | F2 | ***Black****/****CFP+*** | Female | Drive/NHEJ |
| ND253 | OR694389 | ***An. gambiae* Ndoyako** male/**AgNosCd-1** female hybrid intercross | Rep 3 | F2 | ***Black****/****CFP+*** | Female | Drive/WT |
| ND254 | OR694390 | ***An. gambiae* Ndoyako** male/**AgNosCd-1** female hybrid intercross | Rep 3 | F2 | ***Black****/****CFP+*** | Female | Drive/NHEJ |
| ND255 | OR694391 | ***An. gambiae* Ndoyako** male/**AgNosCd-1** female hybrid intercross | Rep 3 | F2 | ***Black****/****CFP+*** | Female | Drive/WT |
| ND256 | OR694392 | ***An. gambiae* Ndoyako** male/**AgNosCd-1** female hybrid intercross | Rep 3 | F2 | ***Black****/****CFP+*** | Female | Drive/WT |
| ND141 | OR694244 | ***An. gambiae* Ndoyako** male/**AgNosCd-1** female hybrid intercross | Rep 1 | F2 | ***Black****/CFP-* | Male | NHEJ/NHEJ |
|  | OR694245 |  |  |  |  |  |  |
| ND142 | OR694246 | ***An. gambiae* Ndoyako** male/**AgNosCd-1** female hybrid intercross | Rep 1 | F2 | ***Black****/CFP-* | Male | WT/NHEJ |
| ND143 | OR694247 | ***An. gambiae* Ndoyako** male/**AgNosCd-1** female hybrid intercross | Rep 1 | F2 | ***Black****/CFP-* | Male | WT/NHEJ |
| ND144 | OR694248 | ***An. gambiae* Ndoyako** male/**AgNosCd-1** female hybrid intercross | Rep 1 | F2 | ***Black****/CFP-* | Male | NHEJ/NHEJ |
|  | OR694249 |  |  |  |  |  |  |
| ND145 | OR694250 | ***An. gambiae* Ndoyako** male/**AgNosCd-1** female hybrid intercross | Rep 1 | F2 | ***Black****/CFP-* | Male | WT/NHEJ |
| ND146 | OR694251 | ***An. gambiae* Ndoyako** male/**AgNosCd-1** female hybrid intercross | Rep 1 | F2 | ***Black****/CFP-* | Female | NHEJ/NHEJ |
|  | OR694252 |  |  |  |  |  |  |
| ND147 | OR694253 | ***An. gambiae* Ndoyako** male/**AgNosCd-1** female hybrid intercross | Rep 1 | F2 | ***Black****/CFP-* | Female | WT/NHEJ |
| ND148 | OR694254 | ***An. gambiae* Ndoyako** male/**AgNosCd-1** female hybrid intercross | Rep 1 | F2 | ***Black****/CFP-* | Female | NHEJ/NHEJ |
|  | OR694255 |  |  |  |  |  |  |
| ND149 | OR694256 | ***An. gambiae* Ndoyako** male/**AgNosCd-1** female hybrid intercross | Rep 1 | F2 | ***Black****/CFP-* | Female | NHEJ/NHEJ |
|  | OR694257 |  |  |  |  |  |  |
| ND150 | OR694258 | ***An. gambiae* Ndoyako** male/**AgNosCd-1** female hybrid intercross | Rep 1 | F2 | ***Black****/CFP-* | Female | NHEJ/NHEJ |
|  | OR694259 |  |  |  |  |  |  |
| ND267 | OR694365 | ***An. gambiae* Ndoyako** male/**AgNosCd-1** female hybrid intercross | Rep 2 | F2 | ***Black****/CFP-* | Female | NHEJ/NHEJ |
|  | OR694366 |  |  |  |  |  |  |
| ND268 | OR694367 | ***An. gambiae* Ndoyako** male/**AgNosCd-1** female hybrid intercross | Rep 2 | F2 | ***Black****/CFP-* | Female | NHEJ/NHEJ |
|  | OR694368 |  |  |  |  |  |  |
| ND269 | OR694369 | ***An. gambiae* Ndoyako** male/**AgNosCd-1** female hybrid intercross | Rep 2 | F2 | ***Black****/CFP-* | Female | NHEJ/NHEJ |
|  | OR694370 |  |  |  |  |  |  |
| ND270 | OR694371 | ***An. gambiae* Ndoyako** male/**AgNosCd-1** female hybrid intercross | Rep 2 | F2 | ***Black****/CFP-* | Male | NHEJ/NHEJ |
|  | OR694372 |  |  |  |  |  |  |
| ND271 | OR694373 | ***An. gambiae* Ndoyako** male/**AgNosCd-1** female hybrid intercross | Rep 2 | F2 | ***Black****/CFP-* | Male | NHEJ/NHEJ |
|  | OR694374 |  |  |  |  |  |  |
| ND245 | OR694375 | ***An. gambiae* Ndoyako** male/**AgNosCd-1** female hybrid intercross | Rep 3 | F2 | ***Black****/CFP-* | Male | WT/NHEJ |
|  | OR694376 |  |  |  |  |  |  |
| ND246 | OR694377 | ***An. gambiae* Ndoyako** male/**AgNosCd-1** female hybrid intercross | Rep 3 | F2 | ***Black****/CFP-* | Male | NHEJ/NHEJ |
|  | OR694378 |  |  |  |  |  |  |
| ND247 | OR694379 | ***An. gambiae* Ndoyako** male/**AgNosCd-1** female hybrid intercross | Rep 3 | F2 | ***Black****/CFP-* | Male | WT/NHEJ |
|  | OR694380 |  |  |  |  |  |  |
| ND248 | OR694381 | ***An. gambiae* Ndoyako** male/**AgNosCd-1** female hybrid intercross | Rep 3 | F2 | ***Black****/CFP-* | Female | NHEJ/NHEJ |
|  | OR694382 |  |  |  |  |  |  |
| ND249 | OR694383 | ***An. gambiae* Ndoyako** male/**AgNosCd-1** female hybrid intercross | Rep 3 | F2 | ***Black****/CFP-* | Female | NHEJ/NHEJ |
|  | OR694384 |  |  |  |  |  |  |
| ND250 | OR694385 | ***An. gambiae* Ndoyako** male/**AgNosCd-1** female hybrid intercross | Rep 3 | F2 | ***Black****/CFP-* | Female | WT/NHEJ |
|  | OR694386 |  |  |  |  |  |  |
| ND132 | OR694226 | ***An. gambiae* Ndoyako** male/**AgNosCd-1** female hybrid intercross | Rep 1 | F2 | *Cardinal/CFP-* | Male | NHEJ/NHEJ |
|  | OR694227 |  |  |  |  |  |  |
| ND133 | OR694228 | ***An. gambiae* Ndoyako** male/**AgNosCd-1** female hybrid intercross | Rep 1 | F2 | *Cardinal/CFP-* | Male | NHEJ/NHEJ |
|  | OR694229 |  |  |  |  |  |  |
| ND134 | OR694230 | ***An. gambiae* Ndoyako** male/**AgNosCd-1** female hybrid intercross | Rep 1 | F2 | *Cardinal/CFP-* | Male | NHEJ/NHEJ |
|  | OR694231 |  |  |  |  |  |  |
| ND135 | OR694232 | ***An. gambiae* Ndoyako** male/**AgNosCd-1** female hybrid intercross | Rep 1 | F2 | *Cardinal/CFP-* | Male | NHEJ/NHEJ |
|  | OR694233 |  |  |  |  |  |  |
| ND136 | OR694234 | ***An. gambiae* Ndoyako** male/**AgNosCd-1** female hybrid intercross | Rep 1 | F2 | *Cardinal/CFP-* | Male | NHEJ/NHEJ |
|  | OR694235 |  |  |  |  |  |  |
| ND137 | OR694236 | ***An. gambiae* Ndoyako** male/**AgNosCd-1** female hybrid intercross | Rep 1 | F2 | *Cardinal/CFP-* | Female | NHEJ/NHEJ |
|  | OR694237 |  |  |  |  |  |  |
| ND138 | OR694238 | ***An. gambiae* Ndoyako** male/**AgNosCd-1** female hybrid intercross | Rep 1 | F2 | *Cardinal/CFP-* | Female | NHEJ/NHEJ |
|  | OR694239 |  |  |  |  |  |  |
| ND139 | OR694240 | ***An. gambiae* Ndoyako** male/**AgNosCd-1** female hybrid intercross | Rep 1 | F2 | *Cardinal/CFP-* | Female | NHEJ/NHEJ |
|  | OR694241 |  |  |  |  |  |  |
| ND140 | OR694242 | ***An. gambiae* Ndoyako** male/**AgNosCd-1** female hybrid intercross | Rep 1 | F2 | *Cardinal/CFP-* | Female | NHEJ/NHEJ |
|  | OR694243 |  |  |  |  |  |  |
| ND272 | OR694403 | ***An. gambiae* Ndoyako** male/**AgNosCd-1** female hybrid intercross | Rep 2 | F2 | *Cardinal/CFP-* | Female | NHEJ/NHEJ |
| ND273 | OR694404 | ***An. gambiae* Ndoyako** male/**AgNosCd-1** female hybrid intercross | Rep 2 | F2 | *Cardinal/CFP-* | Male | NHEJ/NHEJ |
| ND301 |  | **AgNosCd-1** male/***An. gambiae* Ndoyako** female hybrid intercross | Rep1 | F2 | *Cardinal/CFP+* | Male | Drive/Drive |
| ND302 |  | **AgNosCd-1** male/***An. gambiae* Ndoyako** female hybrid intercross | Rep1 | F2 | *Cardinal/CFP+* | Male | Drive/Drive |
| ND303 |  | **AgNosCd-1** male/***An. gambiae* Ndoyako** female hybrid intercross | Rep1 | F2 | *Cardinal/CFP+* | Male | Drive/Drive |
| ND304 |  | **AgNosCd-1** male/***An. gambiae* Ndoyako** female hybrid intercross | Rep1 | F2 | *Cardinal/CFP+* | Male | Drive/Drive |
| ND305 |  | **AgNosCd-1** male/***An. gambiae* Ndoyako** female hybrid intercross | Rep1 | F2 | *Cardinal/CFP+* | Male | Drive/Drive |
| ND306 |  | **AgNosCd-1** male/***An. gambiae* Ndoyako** female hybrid intercross | Rep1 | F2 | *Cardinal/CFP+* | Male | Drive/Drive |
| ND307 |  | **AgNosCd-1** male/***An. gambiae* Ndoyako** female hybrid intercross | Rep1 | F2 | *Cardinal/CFP+* | Male | Drive/Drive |
| ND308 |  | **AgNosCd-1** male/***An. gambiae* Ndoyako** female hybrid intercross | Rep1 | F2 | *Cardinal/CFP+* | Male | Drive/Drive |
| ND309 |  | **AgNosCd-1** male/***An. gambiae* Ndoyako** female hybrid intercross | Rep1 | F2 | *Cardinal/CFP+* | Male | Drive/Drive |
| ND310 |  | **AgNosCd-1** male/***An. gambiae* Ndoyako** female hybrid intercross | Rep1 | F2 | *Cardinal/CFP+* | Male | Drive/Drive |
| ND311 |  | **AgNosCd-1** male/***An. gambiae* Ndoyako** female hybrid intercross | Rep1 | F2 | *Cardinal/CFP+* | Female | Drive/Drive |
| ND312 |  | **AgNosCd-1** male/***An. gambiae* Ndoyako** female hybrid intercross | Rep1 | F2 | *Cardinal/CFP+* | Female | Drive/Drive |
| ND313 |  | **AgNosCd-1** male/***An. gambiae* Ndoyako** female hybrid intercross | Rep1 | F2 | *Cardinal/CFP+* | Female | Drive/Drive |
| ND314 |  | **AgNosCd-1** male/***An. gambiae* Ndoyako** female hybrid intercross | Rep1 | F2 | *Cardinal/CFP+* | Female | Drive/Drive |
| ND315 |  | **AgNosCd-1** male/***An. gambiae* Ndoyako** female hybrid intercross | Rep1 | F2 | *Cardinal/CFP+* | Female | Drive/Drive |
| ND316 |  | **AgNosCd-1** male/***An. gambiae* Ndoyako** female hybrid intercross | Rep1 | F2 | *Cardinal/CFP+* | Female | Drive/Drive |
| ND317 |  | **AgNosCd-1** male/***An. gambiae* Ndoyako** female hybrid intercross | Rep1 | F2 | *Cardinal/CFP+* | Female | Drive/Drive |
| ND318 |  | **AgNosCd-1** male/***An. gambiae* Ndoyako** female hybrid intercross | Rep1 | F2 | *Cardinal/CFP+* | Female | Drive/Drive |
| ND319 |  | **AgNosCd-1** male/***An. gambiae* Ndoyako** female hybrid intercross | Rep1 | F2 | *Cardinal/CFP+* | Female | Drive/Drive |
| ND320 |  | **AgNosCd-1** male/***An. gambiae* Ndoyako** female hybrid intercross | Rep1 | F2 | *Cardinal/CFP+* | Female | Drive/Drive |
| ND321 |  | **AgNosCd-1** male/***An. gambiae* Ndoyako** female hybrid intercross | Rep2 | F2 | *Cardinal/CFP+* | Male | Drive/Drive |
| ND322 |  | **AgNosCd-1** male/***An. gambiae* Ndoyako** female hybrid intercross | Rep2 | F2 | *Cardinal/CFP+* | Male | Drive/Drive |
| ND323 |  | **AgNosCd-1** male/***An. gambiae* Ndoyako** female hybrid intercross | Rep2 | F2 | *Cardinal/CFP+* | Male | Drive/Drive |
| ND324 |  | **AgNosCd-1** male/***An. gambiae* Ndoyako** female hybrid intercross | Rep2 | F2 | *Cardinal/CFP+* | Male | Drive/Drive |
| ND325 |  | **AgNosCd-1** male/***An. gambiae* Ndoyako** female hybrid intercross | Rep2 | F2 | *Cardinal/CFP+* | Male | Drive/Drive |
| ND326 |  | **AgNosCd-1** male/***An. gambiae* Ndoyako** female hybrid intercross | Rep2 | F2 | *Cardinal/CFP+* | Male | Drive/Drive |
| ND327 |  | **AgNosCd-1** male/***An. gambiae* Ndoyako** female hybrid intercross | Rep2 | F2 | *Cardinal/CFP+* | Male | Drive/Drive |
| ND328 |  | **AgNosCd-1** male/***An. gambiae* Ndoyako** female hybrid intercross | Rep2 | F2 | *Cardinal/CFP+* | Male | Drive/Drive |
| ND329 |  | **AgNosCd-1** male/***An. gambiae* Ndoyako** female hybrid intercross | Rep2 | F2 | *Cardinal/CFP+* | Male | Drive/Drive |
| ND330 |  | **AgNosCd-1** male/***An. gambiae* Ndoyako** female hybrid intercross | Rep2 | F2 | *Cardinal/CFP+* | Male | Drive/Drive |
| ND331 |  | **AgNosCd-1** male/***An. gambiae* Ndoyako** female hybrid intercross | Rep2 | F2 | *Cardinal/CFP+* | Female | Drive/Drive |
| ND332 |  | **AgNosCd-1** male/***An. gambiae* Ndoyako** female hybrid intercross | Rep2 | F2 | *Cardinal/CFP+* | Female | Drive/Drive |
| ND333 |  | **AgNosCd-1** male/***An. gambiae* Ndoyako** female hybrid intercross | Rep2 | F2 | *Cardinal/CFP+* | Female | Drive/Drive |
| ND334 |  | **AgNosCd-1** male/***An. gambiae* Ndoyako** female hybrid intercross | Rep2 | F2 | *Cardinal/CFP+* | Female | Drive/Drive |
| ND335 |  | **AgNosCd-1** male/***An. gambiae* Ndoyako** female hybrid intercross | Rep2 | F2 | *Cardinal/CFP+* | Female | Drive/Drive |
| ND336 |  | **AgNosCd-1** male/***An. gambiae* Ndoyako** female hybrid intercross | Rep2 | F2 | *Cardinal/CFP+* | Female | Drive/Drive |
| ND337 |  | **AgNosCd-1** male/***An. gambiae* Ndoyako** female hybrid intercross | Rep2 | F2 | *Cardinal/CFP+* | Female | Drive/Drive |
| ND338 |  | **AgNosCd-1** male/***An. gambiae* Ndoyako** female hybrid intercross | Rep2 | F2 | *Cardinal/CFP+* | Female | Drive/Drive |
| ND339 |  | **AgNosCd-1** male/***An. gambiae* Ndoyako** female hybrid intercross | Rep2 | F2 | *Cardinal/CFP+* | Female | Drive/Drive |
| ND340 |  | **AgNosCd-1** male/***An. gambiae* Ndoyako** female hybrid intercross | Rep2 | F2 | *Cardinal/CFP+* | Female | Drive/Drive |
| ND341 |  | **AgNosCd-1** male/***An. gambiae* Ndoyako** female hybrid intercross | Rep3 | F2 | *Cardinal/CFP+* | Male | Drive/Drive |
| ND342 |  | **AgNosCd-1** male/***An. gambiae* Ndoyako** female hybrid intercross | Rep3 | F2 | *Cardinal/CFP+* | Male | Drive/Drive |
| ND343 |  | **AgNosCd-1** male/***An. gambiae* Ndoyako** female hybrid intercross | Rep3 | F2 | *Cardinal/CFP+* | Male | Drive/Drive |
| ND344 |  | **AgNosCd-1** male/***An. gambiae* Ndoyako** female hybrid intercross | Rep3 | F2 | *Cardinal/CFP+* | Male | Drive/Drive |
| ND345 |  | **AgNosCd-1** male/***An. gambiae* Ndoyako** female hybrid intercross | Rep3 | F2 | *Cardinal/CFP+* | Male | Drive/Drive |
| ND346 |  | **AgNosCd-1** male/***An. gambiae* Ndoyako** female hybrid intercross | Rep3 | F2 | *Cardinal/CFP+* | Male | Drive/Drive |
| ND347 |  | **AgNosCd-1** male/***An. gambiae* Ndoyako** female hybrid intercross | Rep3 | F2 | *Cardinal/CFP+* | Male | Drive/Drive |
| ND348 |  | **AgNosCd-1** male/***An. gambiae* Ndoyako** female hybrid intercross | Rep3 | F2 | *Cardinal/CFP+* | Male | Drive/Drive |
| ND349 |  | **AgNosCd-1** male/***An. gambiae* Ndoyako** female hybrid intercross | Rep3 | F2 | *Cardinal/CFP+* | Male | Drive/Drive |
| ND350 |  | **AgNosCd-1** male/***An. gambiae* Ndoyako** female hybrid intercross | Rep3 | F2 | *Cardinal/CFP+* | Male | Drive/Drive |
| ND351 |  | **AgNosCd-1** male/***An. gambiae* Ndoyako** female hybrid intercross | Rep3 | F2 | *Cardinal/CFP+* | Female | Drive/Drive |
| ND352 |  | **AgNosCd-1** male/***An. gambiae* Ndoyako** female hybrid intercross | Rep3 | F2 | *Cardinal/CFP+* | Female | Drive/Drive |
| ND353 |  | **AgNosCd-1** male/***An. gambiae* Ndoyako** female hybrid intercross | Rep3 | F2 | *Cardinal/CFP+* | Female | Drive/Drive |
| ND354 |  | **AgNosCd-1** male/***An. gambiae* Ndoyako** female hybrid intercross | Rep3 | F2 | *Cardinal/CFP+* | Female | Drive/Drive |
| ND355 |  | **AgNosCd-1** male/***An. gambiae* Ndoyako** female hybrid intercross | Rep3 | F2 | *Cardinal/CFP+* | Female | Drive/Drive |
| ND356 |  | **AgNosCd-1** male/***An. gambiae* Ndoyako** female hybrid intercross | Rep3 | F2 | *Cardinal/CFP+* | Female | Drive/Drive |
| ND357 |  | **AgNosCd-1** male/***An. gambiae* Ndoyako** female hybrid intercross | Rep3 | F2 | *Cardinal/CFP+* | Female | Drive/Drive |
| ND358 |  | **AgNosCd-1** male/***An. gambiae* Ndoyako** female hybrid intercross | Rep3 | F2 | *Cardinal/CFP+* | Female | Drive/Drive |
| ND359 |  | **AgNosCd-1** male/***An. gambiae* Ndoyako** female hybrid intercross | Rep3 | F2 | *Cardinal/CFP+* | Female | Drive/Drive |
| ND360 |  | **AgNosCd-1** male/***An. gambiae* Ndoyako** female hybrid intercross | Rep3 | F2 | *Cardinal/CFP+* | Female | Drive/Drive |
| ND361 |  | ***An. gambiae* Ndoyako** male/**AgNosCd-1** female hybrid intercross | Rep1 | F2 | *Cardinal/CFP+* | Male | Drive/Drive |
| ND362 |  | ***An. gambiae* Ndoyako** male/**AgNosCd-1** female hybrid intercross | Rep1 | F2 | *Cardinal/CFP+* | Male | Drive/Drive |
| ND363 |  | ***An. gambiae* Ndoyako** male/**AgNosCd-1** female hybrid intercross | Rep1 | F2 | *Cardinal/CFP+* | Male | Drive/Drive |
| ND364 | OR694405 | ***An. gambiae* Ndoyako** male/**AgNosCd-1** female hybrid intercross | Rep1 | F2 | *Cardinal/CFP+* | Male | Drive/NHEJ |
| ND365 |  | ***An. gambiae* Ndoyako** male/**AgNosCd-1** female hybrid intercross | Rep1 | F2 | *Cardinal/CFP+* | Male | Drive/Drive |
| ND366 |  | ***An. gambiae* Ndoyako** male/**AgNosCd-1** female hybrid intercross | Rep1 | F2 | *Cardinal/CFP+* | Male | Drive/Drive |
| ND367 |  | ***An. gambiae* Ndoyako** male/**AgNosCd-1** female hybrid intercross | Rep1 | F2 | *Cardinal/CFP+* | Male | Drive/Drive |
| ND368 |  | ***An. gambiae* Ndoyako** male/**AgNosCd-1** female hybrid intercross | Rep1 | F2 | *Cardinal/CFP+* | Male | Drive/Drive |
| ND369 | OR694406 | ***An. gambiae* Ndoyako** male/**AgNosCd-1** female hybrid intercross | Rep1 | F2 | *Cardinal/CFP+* | Male | Drive/NHEJ |
| ND370 | OR694407 | ***An. gambiae* Ndoyako** male/**AgNosCd-1** female hybrid intercross | Rep1 | F2 | *Cardinal/CFP+* | Male | Drive/NHEJ |
| ND371 |  | ***An. gambiae* Ndoyako** male/**AgNosCd-1** female hybrid intercross | Rep1 | F2 | *Cardinal/CFP+* | Female | Drive/Drive |
| ND372 |  | ***An. gambiae* Ndoyako** male/**AgNosCd-1** female hybrid intercross | Rep1 | F2 | *Cardinal/CFP+* | Female | Drive/Drive |
| ND373 |  | ***An. gambiae* Ndoyako** male/**AgNosCd-1** female hybrid intercross | Rep1 | F2 | *Cardinal/CFP+* | Female | Drive/Drive |
| ND374 |  | ***An. gambiae* Ndoyako** male/**AgNosCd-1** female hybrid intercross | Rep1 | F2 | *Cardinal/CFP+* | Female | Drive/Drive |
| ND375 |  | ***An. gambiae* Ndoyako** male/**AgNosCd-1** female hybrid intercross | Rep1 | F2 | *Cardinal/CFP+* | Female | Drive/Drive |
| ND376 |  | ***An. gambiae* Ndoyako** male/**AgNosCd-1** female hybrid intercross | Rep1 | F2 | *Cardinal/CFP+* | Female | Drive/Drive |
| ND377 | OR694408 | ***An. gambiae* Ndoyako** male/**AgNosCd-1** female hybrid intercross | Rep1 | F2 | *Cardinal/CFP+* | Female | Drive/NHEJ |
| ND378 |  | ***An. gambiae* Ndoyako** male/**AgNosCd-1** female hybrid intercross | Rep1 | F2 | *Cardinal/CFP+* | Female | Drive/Drive |
| ND379 | OR694409 | ***An. gambiae* Ndoyako** male/**AgNosCd-1** female hybrid intercross | Rep1 | F2 | *Cardinal/CFP+* | Female | Drive/NHEJ |
| ND380 | OR694410 | ***An. gambiae* Ndoyako** male/**AgNosCd-1** female hybrid intercross | Rep1 | F2 | *Cardinal/CFP+* | Female | Drive/NHEJ |
| ND381 |  | ***An. gambiae* Ndoyako** male/**AgNosCd-1** female hybrid intercross | Rep2 | F2 | *Cardinal/CFP+* | Male | Drive/Drive |
| ND382 |  | ***An. gambiae* Ndoyako** male/**AgNosCd-1** female hybrid intercross | Rep2 | F2 | *Cardinal/CFP+* | Male | Drive/Drive |
| ND383 |  | ***An. gambiae* Ndoyako** male/**AgNosCd-1** female hybrid intercross | Rep2 | F2 | *Cardinal/CFP+* | Male | Drive/Drive |
| ND384 |  | ***An. gambiae* Ndoyako** male/**AgNosCd-1** female hybrid intercross | Rep2 | F2 | *Cardinal/CFP+* | Male | Drive/Drive |
| ND385 |  | ***An. gambiae* Ndoyako** male/**AgNosCd-1** female hybrid intercross | Rep2 | F2 | *Cardinal/CFP+* | Male | Drive/Drive |
| ND386 |  | ***An. gambiae* Ndoyako** male/**AgNosCd-1** female hybrid intercross | Rep2 | F2 | *Cardinal/CFP+* | Male | Drive/Drive |
| ND387 |  | ***An. gambiae* Ndoyako** male/**AgNosCd-1** female hybrid intercross | Rep2 | F2 | *Cardinal/CFP+* | Male | Drive/Drive |
| ND388 |  | ***An. gambiae* Ndoyako** male/**AgNosCd-1** female hybrid intercross | Rep2 | F2 | *Cardinal/CFP+* | Male | Drive/Drive |
| ND389 |  | ***An. gambiae* Ndoyako** male/**AgNosCd-1** female hybrid intercross | Rep2 | F2 | *Cardinal/CFP+* | Male | Drive/Drive |
| ND390 | OR694411 | ***An. gambiae* Ndoyako** male/**AgNosCd-1** female hybrid intercross | Rep2 | F2 | *Cardinal/CFP+* | Male | Drive/NHEJ |
| ND391 | OR694412 | ***An. gambiae* Ndoyako** male/**AgNosCd-1** female hybrid intercross | Rep2 | F2 | *Cardinal/CFP+* | Female | Drive/NHEJ |
| ND392 |  | ***An. gambiae* Ndoyako** male/**AgNosCd-1** female hybrid intercross | Rep2 | F2 | *Cardinal/CFP+* | Female | Drive/Drive |
| ND393 |  | ***An. gambiae* Ndoyako** male/**AgNosCd-1** female hybrid intercross | Rep2 | F2 | *Cardinal/CFP+* | Female | Drive/Drive |
| ND394 |  | ***An. gambiae* Ndoyako** male/**AgNosCd-1** female hybrid intercross | Rep2 | F2 | *Cardinal/CFP+* | Female | Drive/Drive |
| ND395 |  | ***An. gambiae* Ndoyako** male/**AgNosCd-1** female hybrid intercross | Rep2 | F2 | *Cardinal/CFP+* | Female | Drive/Drive |
| ND396 |  | ***An. gambiae* Ndoyako** male/**AgNosCd-1** female hybrid intercross | Rep2 | F2 | *Cardinal/CFP+* | Female | Drive/Drive |
| ND397 |  | ***An. gambiae* Ndoyako** male/**AgNosCd-1** female hybrid intercross | Rep2 | F2 | *Cardinal/CFP+* | Female | Drive/Drive |
| ND398 |  | ***An. gambiae* Ndoyako** male/**AgNosCd-1** female hybrid intercross | Rep2 | F2 | *Cardinal/CFP+* | Female | Drive/Drive |
| ND399 |  | ***An. gambiae* Ndoyako** male/**AgNosCd-1** female hybrid intercross | Rep2 | F2 | *Cardinal/CFP+* | Female | Drive/Drive |
| ND400 |  | ***An. gambiae* Ndoyako** male/**AgNosCd-1** female hybrid intercross | Rep2 | F2 | *Cardinal/CFP+* | Female | Drive/Drive |
| ND401 |  | ***An. gambiae* Ndoyako** male/**AgNosCd-1** female hybrid intercross | Rep3 | F2 | *Cardinal/CFP+* | Male | Drive/Drive |
| ND402 |  | ***An. gambiae* Ndoyako** male/**AgNosCd-1** female hybrid intercross | Rep3 | F2 | *Cardinal/CFP+* | Male | Drive/Drive |
| ND403 |  | ***An. gambiae* Ndoyako** male/**AgNosCd-1** female hybrid intercross | Rep3 | F2 | *Cardinal/CFP+* | Male | Drive/Drive |
| ND404 | OR694413 | ***An. gambiae* Ndoyako** male/**AgNosCd-1** female hybrid intercross | Rep3 | F2 | *Cardinal/CFP+* | Male | Drive/NHEJ |
| ND405 |  | ***An. gambiae* Ndoyako** male/**AgNosCd-1** female hybrid intercross | Rep3 | F2 | *Cardinal/CFP+* | Male | Drive/Drive |
| ND406 |  | ***An. gambiae* Ndoyako** male/**AgNosCd-1** female hybrid intercross | Rep3 | F2 | *Cardinal/CFP+* | Male | Drive/Drive |
| ND407 |  | ***An. gambiae* Ndoyako** male/**AgNosCd-1** female hybrid intercross | Rep3 | F2 | *Cardinal/CFP+* | Male | Drive/Drive |
| ND408 |  | ***An. gambiae* Ndoyako** male/**AgNosCd-1** female hybrid intercross | Rep3 | F2 | *Cardinal/CFP+* | Male | Drive/Drive |
| ND409 |  | ***An. gambiae* Ndoyako** male/**AgNosCd-1** female hybrid intercross | Rep3 | F2 | *Cardinal/CFP+* | Male | Drive/Drive |
| ND410 |  | ***An. gambiae* Ndoyako** male/**AgNosCd-1** female hybrid intercross | Rep3 | F2 | *Cardinal/CFP+* | Male | Drive/Drive |
| ND411 |  | ***An. gambiae* Ndoyako** male/**AgNosCd-1** female hybrid intercross | Rep3 | F2 | *Cardinal/CFP+* | Female | Drive/Drive |
| ND412 | OR694414 | ***An. gambiae* Ndoyako** male/**AgNosCd-1** female hybrid intercross | Rep3 | F2 | *Cardinal/CFP+* | Female | Drive/NHEJ |
| ND413 | OR694415 | ***An. gambiae* Ndoyako** male/**AgNosCd-1** female hybrid intercross | Rep3 | F2 | *Cardinal/CFP+* | Female | Drive/NHEJ |
| ND414 |  | ***An. gambiae* Ndoyako** male/**AgNosCd-1** female hybrid intercross | Rep3 | F2 | *Cardinal/CFP+* | Female | Drive/Drive |
| ND415 |  | ***An. gambiae* Ndoyako** male/**AgNosCd-1** female hybrid intercross | Rep3 | F2 | *Cardinal/CFP+* | Female | Drive/Drive |
| ND416 | OR694416 | ***An. gambiae* Ndoyako** male/**AgNosCd-1** female hybrid intercross | Rep3 | F2 | *Cardinal/CFP+* | Female | Drive/Drive |
| ND417 | OR694417 | ***An. gambiae* Ndoyako** male/**AgNosCd-1** female hybrid intercross | Rep3 | F2 | *Cardinal/CFP+* | Female | Drive/NHEJ |
|  | OR694418 |  |  |  |  |  |  |
| ND418 |  | ***An. gambiae* Ndoyako** male/**AgNosCd-1** female hybrid intercross | Rep3 | F2 | *Cardinal/CFP+* | Female | Drive/Drive |
| ND419 | OR694419 | ***An. gambiae* Ndoyako** male/**AgNosCd-1** female hybrid intercross | Rep3 | F2 | *Cardinal/CFP+* | Female | Drive/NHEJ |
| ND420 |  | ***An. gambiae* Ndoyako** male/**AgNosCd-1** female hybrid intercross | Rep3 | F2 | *Cardinal/CFP+* | Female | Drive/Drive |
| MT122 | OR694526 | *An. coluzzii* Mopti male x AgNosCd-1 female | Rep 3 | F1 | *Cardinal/CFP+* | Female | Drive/NHEJ |
|  | OR694527 |  |  |  |  |  |  |
| MT123 | OR694528 | *An. coluzzii* Mopti male x AgNosCd-1 female | Rep 3 | F1 | *Cardinal/CFP+* | Female | Drive/NHEJ |
| MT124 | OR694529 | *An. coluzzii* Mopti male x AgNosCd-1 female | Rep 3 | F1 | *Cardinal/CFP+* | Female | Drive/NHEJ |
| MT125 | OR694530 | *An. coluzzii* Mopti male x AgNosCd-1 female | Rep 3 | F1 | *Cardinal/CFP+* | Female | Drive/NHEJ |
|  | OR694531 |  |  |  |  |  |  |
| MT126 | OR694532 | *An. coluzzii* Mopti male x AgNosCd-1 female | Rep 3 | F1 | *Cardinal/CFP+* | Female | Drive/NHEJ |
|  | OR694533 |  |  |  |  |  |  |
| MT127 | OR694534 | *An. coluzzii* Mopti male x AgNosCd-1 female | Rep 3 | F1 | *Cardinal/CFP+* | Female | Drive/NHEJ |
| MT128 | OR694535 | *An. coluzzii* Mopti male x AgNosCd-1 female | Rep 3 | F1 | *Cardinal/CFP+* | Female | Drive/NHEJ |
|  | OR694536 |  |  |  |  |  |  |
| MT129 | OR694537 | *An. coluzzii* Mopti male x AgNosCd-1 female | Rep 3 | F1 | *Cardinal/CFP+* | Female | Drive/NHEJ |
|  | OR694538 |  |  |  |  |  |  |
| MT130 | OR694539 | *An. coluzzii* Mopti male x AgNosCd-1 female | Rep 3 | F1 | *Cardinal/CFP+* | Female | Drive/NHEJ |
| MT131 | OR694540 | *An. coluzzii* Mopti male x AgNosCd-1 female | Rep 3 | F1 | *Cardinal/CFP+* | Female | Drive/NHEJ |
|  | OR694541 |  |  |  |  |  |  |
| MT132 | OR694542 | *An. coluzzii* Mopti male x AgNosCd-1 female | Rep 3 | F1 | *Cardinal/CFP+* | Female | Drive/NHEJ |
| MT133 | OR694543 | *An. coluzzii* Mopti male x AgNosCd-1 female | Rep 3 | F1 | *Cardinal/CFP+* | Female | Drive/NHEJ |
|  | OR694544 |  |  |  |  |  |  |
| MT134 | OR694545 | *An. coluzzii* Mopti male x AgNosCd-1 female | Rep 2 | F1 | *Cardinal/CFP+* | Female | Drive/NHEJ |
|  | OR694546 |  |  |  |  |  |  |
| MT135 | OR694547 | *An. coluzzii* Mopti male x AgNosCd-1 female | Rep 2 | F1 | *Cardinal/CFP+* | Female | Drive/NHEJ |
|  | OR694548 |  |  |  |  |  |  |
| MT136 | OR694549 | *An. coluzzii* Mopti male x AgNosCd-1 female | Rep 2 | F1 | *Cardinal/CFP+* | Female | Drive/NHEJ |
|  | OR694550 |  |  |  |  |  |  |
| MT137 | OR694551 | *An. coluzzii* Mopti male x AgNosCd-1 female | Rep 2 | F1 | *Cardinal/CFP+* | Female | Drive/NHEJ |
| MT138 | OR694552 | *An. coluzzii* Mopti male x AgNosCd-1 female | Rep 2 | F1 | *Cardinal/CFP+* | Female | Drive/NHEJ |
| MT139 | OR694553 | *An. coluzzii* Mopti male x AgNosCd-1 female | Rep 2 | F1 | *Cardinal/CFP+* | Female | Drive/NHEJ |
| MT140 | OR694554 | *An. coluzzii* Mopti male x AgNosCd-1 female | Rep 2 | F1 | *Cardinal/CFP+* | Female | Drive/NHEJ |
| MT141 | OR694555 | *An. coluzzii* Mopti male x AgNosCd-1 female | Rep 2 | F1 | *Cardinal/CFP+* | Female | Drive/NHEJ |
|  | OR694556 |  |  |  |  |  |  |
| MT142 | OR694557 | *An. coluzzii* Mopti male x AgNosCd-1 female | Rep 2 | F1 | *Cardinal/CFP+* | Female | Drive/NHEJ |
| MT143 | OR694558 | *An. coluzzii* Mopti male x AgNosCd-1 female | Rep 2 | F1 | *Cardinal/CFP+* | Female | Drive/NHEJ |
|  | OR694559 |  |  |  |  |  |  |
| MT144 | OR694560 | *An. coluzzii* Mopti male x AgNosCd-1 female | Rep 2 | F1 | *Cardinal/CFP+* | Female | Drive/NHEJ |
| MT145 | OR694561 | *An. coluzzii* Mopti male x AgNosCd-1 female | Rep 2 | F1 | *Cardinal/CFP+* | Female | Drive/NHEJ |
|  | OR694562 |  |  |  |  |  |  |
| MT146 | OR694563 | *An. coluzzii* Mopti male x AgNosCd-1 female | Rep 1 | F1 | *Cardinal/CFP+* | Female | Drive/NHEJ |
| MT147 | OR694564 | *An. coluzzii* Mopti male x AgNosCd-1 female | Rep 1 | F1 | *Cardinal/CFP+* | Female | Drive/NHEJ |
| MT148 | OR694565 | *An. coluzzii* Mopti male x AgNosCd-1 female | Rep 1 | F1 | *Cardinal/CFP+* | Female | Drive/NHEJ |
|  | OR694566 |  |  |  |  |  |  |
| MT149 | OR694567 | *An. coluzzii* Mopti male x AgNosCd-1 female | Rep 1 | F1 | *Cardinal/CFP+* | Female | Drive/NHEJ |
| MT150 | OR694568 | *An. coluzzii* Mopti male x AgNosCd-1 female | Rep 1 | F1 | *Cardinal/CFP+* | Female | Drive/NHEJ |
|  | OR694569 |  |  |  |  |  |  |
| MT151 | OR694570 | *An. coluzzii* Mopti male x AgNosCd-1 female | Rep 1 | F1 | *Cardinal/CFP+* | Female | Drive/NHEJ |
|  | OR694571 |  |  |  |  |  |  |
| MT152 | OR694572 | *An. coluzzii* Mopti male x AgNosCd-1 female | Rep 1 | F1 | *Cardinal/CFP+* | Female | Drive/NHEJ |
|  | OR694573 |  |  |  |  |  |  |
| MT153 | OR694574 | *An. coluzzii* Mopti male x AgNosCd-1 female | Rep 1 | F1 | *Cardinal/CFP+* | Female | Drive/NHEJ |
| MT154 | OR694575 | *An. coluzzii* Mopti male x AgNosCd-1 female | Rep 1 | F1 | *Cardinal/CFP+* | Female | Drive/NHEJ |
| MT155 | OR694576 | *An. coluzzii* Mopti male x AgNosCd-1 female | Rep 2 | F1 | *Cardinal/CFP+* | Female | Drive/NHEJ |
|  | OR694577 |  |  |  |  |  |  |
| MT156 | OR694578 | *An. coluzzii* Mopti male x AgNosCd-1 female | Rep 2 | F1 | *Cardinal/CFP+* | Female | Drive/NHEJ |
| MT157 | OR694579 | *An. coluzzii* Mopti male x AgNosCd-1 female | Rep 2 | F1 | *Cardinal/CFP+* | Female | Drive/NHEJ |
|  | OR694580 |  |  |  |  |  |  |
| MT158 | OR694581 | *An. coluzzii* Mopti male x AgNosCd-1 female | Rep 2 | F1 | *Cardinal/CFP+* | Female | Drive/NHEJ |
| MT159 | OR694582 | *An. coluzzii* Mopti male x AgNosCd-1 female | Rep 2 | F1 | *Cardinal/CFP+* | Female | Drive/NHEJ |
|  | OR694583 |  |  |  |  |  |  |
| MT160 | OR694584 | *An. coluzzii* Mopti male x AgNosCd-1 female | Rep 2 | F1 | *Cardinal/CFP+* | Female | Drive/NHEJ |
| MT161 | OR694585 | *An. coluzzii* Mopti male x AgNosCd-1 female | Rep 2 | F1 | *Cardinal/CFP+* | Female | Drive/NHEJ |
|  | OR694586 |  |  |  |  |  |  |
| MT162 | OR694587 | *An. coluzzii* Mopti male x AgNosCd-1 female | Rep 2 | F1 | *Cardinal/CFP+* | Female | Drive/NHEJ |
| MT163 | OR694588 | *An. coluzzii* Mopti male x AgNosCd-1 female | Rep 2 | F1 | *Cardinal/CFP+* | Female | Drive/NHEJ |
| MT164 | OR694589 | *An. coluzzii* Mopti male x AgNosCd-1 female | Rep 3 | F1 | *Cardinal/CFP+* | Female | Drive/NHEJ |
|  | OR694590 |  |  |  |  |  |  |
| MT165 | OR694591 | *An. coluzzii* Mopti male x AgNosCd-1 female | Rep 3 | F1 | *Cardinal/CFP+* | Female | Drive/NHEJ |
| MT166 | OR694592 | *An. coluzzii* Mopti male x AgNosCd-1 female | Rep 3 | F1 | *Cardinal/CFP+* | Female | Drive/NHEJ |
|  | OR694593 |  |  |  |  |  |  |
| MT167 | OR694594 | *An. coluzzii* Mopti male x AgNosCd-1 female | Rep 3 | F1 | *Cardinal/CFP+* | Female | Drive/NHEJ |
|  | OR694595 |  |  |  |  |  |  |
| MT168 | OR694596 | *An. coluzzii* Mopti male x AgNosCd-1 female | Rep 3 | F1 | *Cardinal/CFP+* | Female | Drive/NHEJ |
| MT169 | OR694597 | *An. coluzzii* Mopti male x AgNosCd-1 female | Rep 3 | F1 | *Cardinal/CFP+* | Female | Drive/NHEJ |
|  | OR694598 |  |  |  |  |  |  |
| MT170 | OR694599 | *An. coluzzii* Mopti male x AgNosCd-1 female | Rep 3 | F1 | *Cardinal/CFP+* | Female | Drive/NHEJ |
| MT171 | OR694600 | *An. coluzzii* Mopti male x AgNosCd-1 female | Rep 3 | F1 | *Cardinal/CFP+* | Female | Drive/NHEJ |
| MT172 | OR694601 | *An. coluzzii* Mopti male x AgNosCd-1 female | Rep 3 | F1 | *Cardinal/CFP+* | Female | Drive/NHEJ |
|  | OR694602 |  |  |  |  |  |  |
| MT173 | OR694603 | *An. coluzzii* Mopti male x AgNosCd-1 female | Rep 3 | F1 | *Cardinal/CFP+* | Female | Drive/NHEJ |
| MT174 | OR694604 | *An. coluzzii* Mopti male x AgNosCd-1 female | Rep 3 | F1 | *Cardinal/CFP+* | Female | Drive/NHEJ |
|  | OR694605 |  |  |  |  |  |  |
| MT175 | OR694606 | *An. coluzzii* Mopti male x AgNosCd-1 female | Rep 3 | F1 | *Cardinal/CFP+* | Female | Drive/NHEJ |
|  | OR694607 |  |  |  |  |  |  |
| MT176 | OR694608 | *An. coluzzii* Mopti male x AgNosCd-1 female | Rep 3 | F1 | *Cardinal/CFP+* | Female | Drive/NHEJ |
|  | OR694609 |  |  |  |  |  |  |
| MT177 | OR694610 | *An. coluzzii* Mopti male x AgNosCd-1 female | Rep 3 | F1 | *Cardinal/CFP+* | Female | Drive/NHEJ |
| MT178 | OR694611 | *An. coluzzii* Mopti male x AgNosCd-1 female | Rep 1 | F1 | *Cardinal/CFP+* | Male | Drive/NHEJ |
|  | OR694612 |  |  |  |  |  |  |
| MT179 | OR694613 | *An. coluzzii* Mopti male x AgNosCd-1 female | Rep 1 | F1 | *Cardinal/CFP+* | Male | Drive/NHEJ |
|  | OR694614 |  |  |  |  |  |  |
| MT180 | OR694615 | *An. coluzzii* Mopti male x AgNosCd-1 female | Rep 1 | F1 | *Cardinal/CFP+* | Male | Drive/NHEJ |
| MT181 | OR694616 | *An. coluzzii* Mopti male x AgNosCd-1 female | Rep 1 | F1 | *Cardinal/CFP+* | Male | Drive/NHEJ |
|  | OR694617 |  |  |  |  |  |  |
| MT182 | OR694618 | *An. coluzzii* Mopti male x AgNosCd-1 female | Rep 1 | F1 | *Cardinal/CFP+* | Male | Drive/NHEJ |
| MT183 | OR694619 | *An. coluzzii* Mopti male x AgNosCd-1 female | Rep 2 | F1 | *Cardinal/CFP+* | Male | Drive/NHEJ |
| MT184 | OR694620 | *An. coluzzii* Mopti male x AgNosCd-1 female | Rep 2 | F1 | *Cardinal/CFP+* | Male | Drive/NHEJ |
|  | OR694621 |  |  |  |  |  |  |
| MT185 | OR694622 | *An. coluzzii* Mopti male x AgNosCd-1 female | Rep 2 | F1 | *Cardinal/CFP+* | Male | Drive/NHEJ |
| MT186 | OR694623 | *An. coluzzii* Mopti male x AgNosCd-1 female | Rep 2 | F1 | *Cardinal/CFP+* | Male | Drive/NHEJ |
|  | OR694624 |  |  |  |  |  |  |
| MT187 | OR694625 | *An. coluzzii* Mopti male x AgNosCd-1 female | Rep 2 | F1 | *Cardinal/CFP+* | Male | Drive/NHEJ |
| MT188 | OR694626 | *An. coluzzii* Mopti male x AgNosCd-1 female | Rep 3 | F1 | *Cardinal/CFP+* | Male | Drive/NHEJ |
|  | OR694627 |  |  |  |  |  |  |
| MT189 | OR694628 | *An. coluzzii* Mopti male x AgNosCd-1 female | Rep 3 | F1 | *Cardinal/CFP+* | Male | Drive/NHEJ |
| MT190 | OR694629 | *An. coluzzii* Mopti male x AgNosCd-1 female | Rep 3 | F1 | *Cardinal/CFP+* | Male | Drive/NHEJ |
|  | OR694630 |  |  |  |  |  |  |
| MT421 | OR694718 | *An. coluzzii* Mopti male x AgNosCd-1 female | Rep1 | F1 | ***Black****/****CFP+*** | Male | Drive/NHEJ |
| MT422 | OR694719 | *An. coluzzii* Mopti male x AgNosCd-1 female | Rep1 | F1 | ***Black****/****CFP+*** | Male | Drive/WT |
| MT423 | OR694720 | *An. coluzzii* Mopti male x AgNosCd-1 female | Rep1 | F1 | ***Black****/****CFP+*** | Male | Drive/WT |
| MT424 | OR694721 | *An. coluzzii* Mopti male x AgNosCd-1 female | Rep1 | F1 | ***Black****/****CFP+*** | Male | Drive/NHEJ |
|  | OR694722 |  |  |  |  |  |  |
| MT425 | OR694723 | *An. coluzzii* Mopti male x AgNosCd-1 female | Rep1 | F1 | ***Black****/****CFP+*** | Male | Drive/WT |
| MT426 | OR694724 | *An. coluzzii* Mopti male x AgNosCd-1 female | Rep1 | F1 | ***Black****/****CFP+*** | Male | Drive/WT |
| MT427 | OR694725 | *An. coluzzii* Mopti male x AgNosCd-1 female | Rep1 | F1 | ***Black****/****CFP+*** | Male | Drive/WT |
| MT428 | OR694726 | *An. coluzzii* Mopti male x AgNosCd-1 female | Rep1 | F1 | ***Black****/****CFP+*** | Male | Drive/WT |
| MT429 | OR694727 | *An. coluzzii* Mopti male x AgNosCd-1 female | Rep1 | F1 | ***Black****/****CFP+*** | Male | Drive/WT |
| MT430 | OR694728 | *An. coluzzii* Mopti male x AgNosCd-1 female | Rep1 | F1 | ***Black****/****CFP+*** | Male | Drive/NHEJ |
|  | OR694729 |  |  |  |  |  |  |
| MT431 | OR694730 | *An. coluzzii* Mopti male x AgNosCd-1 female | Rep1 | F1 | ***Black****/****CFP+*** | Female | Drive/NHEJ |
|  | OR694731 |  |  |  |  |  |  |
| MT432 | OR694732 | *An. coluzzii* Mopti male x AgNosCd-1 female | Rep1 | F1 | ***Black****/****CFP+*** | Female | Drive/NHEJ |
|  | OR694733 |  |  |  |  |  |  |
| MT433 | OR694734 | *An. coluzzii* Mopti male x AgNosCd-1 female | Rep1 | F1 | ***Black****/****CFP+*** | Female | Drive/WT |
| MT434 | OR694735 | *An. coluzzii* Mopti male x AgNosCd-1 female | Rep1 | F1 | ***Black****/****CFP+*** | Female | Drive/NHEJ |
|  | OR694736 |  |  |  |  |  |  |
| MT435 | OR694737 | *An. coluzzii* Mopti male x AgNosCd-1 female | Rep1 | F1 | ***Black****/****CFP+*** | Female | Drive/NHEJ |
|  | OR694738 |  |  |  |  |  |  |
| MT436 | OR694739 | *An. coluzzii* Mopti male x AgNosCd-1 female | Rep1 | F1 | ***Black****/****CFP+*** | Female | Drive/WT |
| MT437 | OR694740 | *An. coluzzii* Mopti male x AgNosCd-1 female | Rep1 | F1 | ***Black****/****CFP+*** | Female | Drive/WT |
| MT438 | OR694741 | *An. coluzzii* Mopti male x AgNosCd-1 female | Rep1 | F1 | ***Black****/****CFP+*** | Female | Drive/WT |
| MT439 | OR694742 | *An. coluzzii* Mopti male x AgNosCd-1 female | Rep1 | F1 | ***Black****/****CFP+*** | Female | Drive/WT |
| MT440 | OR694743 | *An. coluzzii* Mopti male x AgNosCd-1 female | Rep1 | F1 | ***Black****/****CFP+*** | Female | Drive/WT |
| MT441 | OR694744 | *An. coluzzii* Mopti male x AgNosCd-1 female | Rep2 | F1 | ***Black****/****CFP+*** | Male | Drive/NHEJ |
|  | OR694745 |  |  |  |  |  |  |
| MT442 | OR694746 | *An. coluzzii* Mopti male x AgNosCd-1 female | Rep2 | F1 | ***Black****/****CFP+*** | Male | Drive/WT |
| MT443 | OR694747 | *An. coluzzii* Mopti male x AgNosCd-1 female | Rep2 | F1 | ***Black****/****CFP+*** | Male | Drive/WT |
| MT444 | OR694748 | *An. coluzzii* Mopti male x AgNosCd-1 female | Rep2 | F1 | ***Black****/****CFP+*** | Male | Drive/WT |
| MT445 | OR694749 | *An. coluzzii* Mopti male x AgNosCd-1 female | Rep2 | F1 | ***Black****/****CFP+*** | Male | Drive/NHEJ |
| MT446 | OR694750 | *An. coluzzii* Mopti male x AgNosCd-1 female | Rep2 | F1 | ***Black****/****CFP+*** | Male | Drive/WT |
| MT447 | OR694751 | *An. coluzzii* Mopti male x AgNosCd-1 female | Rep2 | F1 | ***Black****/****CFP+*** | Male | Drive/NHEJ |
|  | OR694752 |  |  |  |  |  |  |
| MT448 | OR694753 | *An. coluzzii* Mopti male x AgNosCd-1 female | Rep2 | F1 | ***Black****/****CFP+*** | Male | Drive/WT |
| MT449 | OR694754 | *An. coluzzii* Mopti male x AgNosCd-1 female | Rep2 | F1 | ***Black****/****CFP+*** | Male | Drive/NHEJ |
| MT450 | OR694755 | *An. coluzzii* Mopti male x AgNosCd-1 female | Rep2 | F1 | ***Black****/****CFP+*** | Male | Drive/NHEJ |
|  | OR694756 |  |  |  |  |  |  |
| MT451 | OR694757 | *An. coluzzii* Mopti male x AgNosCd-1 female | Rep2 | F1 | ***Black****/****CFP+*** | Female | Drive/WT |
| MT452 | OR694758 | *An. coluzzii* Mopti male x AgNosCd-1 female | Rep2 | F1 | ***Black****/****CFP+*** | Female | Drive/WT |
| MT453 | OR694759 | *An. coluzzii* Mopti male x AgNosCd-1 female | Rep2 | F1 | ***Black****/****CFP+*** | Female | Drive/NHEJ |
| MT454 | OR694760 | *An. coluzzii* Mopti male x AgNosCd-1 female | Rep2 | F1 | ***Black****/****CFP+*** | Female | Drive/WT |
| MT455 | OR694761 | *An. coluzzii* Mopti male x AgNosCd-1 female | Rep2 | F1 | ***Black****/****CFP+*** | Female | Drive/WT |
| MT456 | OR694762 | *An. coluzzii* Mopti male x AgNosCd-1 female | Rep2 | F1 | ***Black****/****CFP+*** | Female | Drive/NHEJ |
| MT457 | OR694763 | *An. coluzzii* Mopti male x AgNosCd-1 female | Rep2 | F1 | ***Black****/****CFP+*** | Female | Drive/WT |
| MT458 | OR694764 | *An. coluzzii* Mopti male x AgNosCd-1 female | Rep2 | F1 | ***Black****/****CFP+*** | Female | Drive/WT |
| MT459 | OR694765 | *An. coluzzii* Mopti male x AgNosCd-1 female | Rep2 | F1 | ***Black****/****CFP+*** | Female | Drive/WT |
| MT460 | OR694766 | *An. coluzzii* Mopti male x AgNosCd-1 female | Rep2 | F1 | ***Black****/****CFP+*** | Female | Drive/WT |
| MT461 | OR694767 | *An. coluzzii* Mopti male x AgNosCd-1 female | Rep3 | F1 | ***Black****/****CFP+*** | Male | Drive/WT |
| MT462 | OR694768 | *An. coluzzii* Mopti male x AgNosCd-1 female | Rep3 | F1 | ***Black****/****CFP+*** | Male | Drive/NHEJ |
|  | OR694769 |  |  |  |  |  |  |
| MT463 | OR694770 | *An. coluzzii* Mopti male x AgNosCd-1 female | Rep3 | F1 | ***Black****/****CFP+*** | Male | Drive/WT |
| MT464 | OR694771 | *An. coluzzii* Mopti male x AgNosCd-1 female | Rep3 | F1 | ***Black****/****CFP+*** | Male | Drive/WT |
| MT465 | OR694772 | *An. coluzzii* Mopti male x AgNosCd-1 female | Rep3 | F1 | ***Black****/****CFP+*** | Male | Drive/WT |
| MT466 | OR694773 | *An. coluzzii* Mopti male x AgNosCd-1 female | Rep3 | F1 | ***Black****/****CFP+*** | Male | Drive/WT |
| MT467 | OR694774 | *An. coluzzii* Mopti male x AgNosCd-1 female | Rep3 | F1 | ***Black****/****CFP+*** | Male | Drive/WT |
| MT468 | OR694775 | *An. coluzzii* Mopti male x AgNosCd-1 female | Rep3 | F1 | ***Black****/****CFP+*** | Male | Drive/WT |
| MT469 | OR694776 | *An. coluzzii* Mopti male x AgNosCd-1 female | Rep3 | F1 | ***Black****/****CFP+*** | Male | Drive/NHEJ |
| MT470 | OR694777 | *An. coluzzii* Mopti male x AgNosCd-1 female | Rep3 | F1 | ***Black****/****CFP+*** | Male | Drive/WT |
| MT471 | OR694778 | *An. coluzzii* Mopti male x AgNosCd-1 female | Rep3 | F1 | ***Black****/****CFP+*** | Female | Drive/WT |
| MT472 | OR694779 | *An. coluzzii* Mopti male x AgNosCd-1 female | Rep3 | F1 | ***Black****/****CFP+*** | Female | Drive/WT |
| MT473 | OR694780 | *An. coluzzii* Mopti male x AgNosCd-1 female | Rep3 | F1 | ***Black****/****CFP+*** | Female | Drive/WT |
| MT474 | OR694781 | *An. coluzzii* Mopti male x AgNosCd-1 female | Rep3 | F1 | ***Black****/****CFP+*** | Female | Drive/WT |
| MT475 | OR694782 | *An. coluzzii* Mopti male x AgNosCd-1 female | Rep3 | F1 | ***Black****/****CFP+*** | Female | Drive/WT |
| MT476 | OR694783 | *An. coluzzii* Mopti male x AgNosCd-1 female | Rep3 | F1 | ***Black****/****CFP+*** | Female | Drive/NHEJ |
| MT477 | OR694784 | *An. coluzzii* Mopti male x AgNosCd-1 female | Rep3 | F1 | ***Black****/****CFP+*** | Female | Drive/WT |
| MT478 | OR694785 | *An. coluzzii* Mopti male x AgNosCd-1 female | Rep3 | F1 | ***Black****/****CFP+*** | Female | Drive/WT |
| MT479 | OR694786 | *An. coluzzii* Mopti male x AgNosCd-1 female | Rep3 | F1 | ***Black****/****CFP+*** | Female | Drive/NHEJ |
| MT480 | OR694787 | *An. coluzzii* Mopti male x AgNosCd-1 female | Rep3 | F1 | ***Black****/****CFP+*** | Female | Drive/NHEJ |
| MT481 | OR694788 | AgNosCd-1 male x *An. coluzzii* Mopti female | Rep1 | F1 | ***Black****/****CFP+*** | Male | Drive/WT |
| MT482 | OR694789 | AgNosCd-1 male x *An. coluzzii* Mopti female | Rep1 | F1 | ***Black****/****CFP+*** | Male | Drive/WT |
| MT483 | OR694790 | AgNosCd-1 male x *An. coluzzii* Mopti female | Rep1 | F1 | ***Black****/****CFP+*** | Male | Drive/WT |
| MT484 | OR694791 | AgNosCd-1 male x *An. coluzzii* Mopti female | Rep1 | F1 | ***Black****/****CFP+*** | Male | Drive/WT |
| MT485 | OR694792 | AgNosCd-1 male x *An. coluzzii* Mopti female | Rep1 | F1 | ***Black****/****CFP+*** | Male | Drive/WT |
| MT486 | OR694793 | AgNosCd-1 male x *An. coluzzii* Mopti female | Rep1 | F1 | ***Black****/****CFP+*** | Male | Drive/WT |
| MT487 | OR694794 | AgNosCd-1 male x *An. coluzzii* Mopti female | Rep1 | F1 | ***Black****/****CFP+*** | Male | Drive/WT |
| MT488 | OR694795 | AgNosCd-1 male x *An. coluzzii* Mopti female | Rep1 | F1 | ***Black****/****CFP+*** | Male | Drive/WT |
| MT489 | OR694796 | AgNosCd-1 male x *An. coluzzii* Mopti female | Rep1 | F1 | ***Black****/****CFP+*** | Male | Drive/WT |
| MT490 | OR694797 | AgNosCd-1 male x *An. coluzzii* Mopti female | Rep1 | F1 | ***Black****/****CFP+*** | Male | Drive/WT |
| MT491 | OR694798 | AgNosCd-1 male x *An. coluzzii* Mopti female | Rep1 | F1 | ***Black****/****CFP+*** | Female | Drive/WT |
| MT492 | OR694799 | AgNosCd-1 male x *An. coluzzii* Mopti female | Rep1 | F1 | ***Black****/****CFP+*** | Female | Drive/WT |
| MT493 | OR694800 | AgNosCd-1 male x *An. coluzzii* Mopti female | Rep1 | F1 | ***Black****/****CFP+*** | Female | Drive/WT |
| MT494 | OR694801 | AgNosCd-1 male x *An. coluzzii* Mopti female | Rep1 | F1 | ***Black****/****CFP+*** | Female | Drive/WT |
| MT495 | OR694802 | AgNosCd-1 male x *An. coluzzii* Mopti female | Rep1 | F1 | ***Black****/****CFP+*** | Female | Drive/WT |
| MT496 | OR694803 | AgNosCd-1 male x *An. coluzzii* Mopti female | Rep1 | F1 | ***Black****/****CFP+*** | Female | Drive/WT |
| MT497 | OR694804 | AgNosCd-1 male x *An. coluzzii* Mopti female | Rep1 | F1 | ***Black****/****CFP+*** | Female | Drive/WT |
| MT498 | OR694805 | AgNosCd-1 male x *An. coluzzii* Mopti female | Rep1 | F1 | ***Black****/****CFP+*** | Female | Drive/WT |
| MT499 | OR694806 | AgNosCd-1 male x *An. coluzzii* Mopti female | Rep1 | F1 | ***Black****/****CFP+*** | Female | Drive/WT |
| MT500 | OR694807 | AgNosCd-1 male x *An. coluzzii* Mopti female | Rep1 | F1 | ***Black****/****CFP+*** | Female | Drive/WT |
| MT501 | OR694808 | AgNosCd-1 male x *An. coluzzii* Mopti female | Rep2 | F1 | ***Black****/****CFP+*** | Male | Drive/WT |
| MT502 | OR694809 | AgNosCd-1 male x *An. coluzzii* Mopti female | Rep2 | F1 | ***Black****/****CFP+*** | Male | Drive/WT |
| MT503 | OR694810 | AgNosCd-1 male x *An. coluzzii* Mopti female | Rep2 | F1 | ***Black****/****CFP+*** | Male | Drive/WT |
| MT504 | OR694811 | AgNosCd-1 male x *An. coluzzii* Mopti female | Rep2 | F1 | ***Black****/****CFP+*** | Male | Drive/WT |
| MT505 | OR694812 | AgNosCd-1 male x *An. coluzzii* Mopti female | Rep2 | F1 | ***Black****/****CFP+*** | Male | Drive/WT |
| MT506 | OR694813 | AgNosCd-1 male x *An. coluzzii* Mopti female | Rep2 | F1 | ***Black****/****CFP+*** | Male | Drive/WT |
| MT507 | OR694814 | AgNosCd-1 male x *An. coluzzii* Mopti female | Rep2 | F1 | ***Black****/****CFP+*** | Male | Drive/WT |
| MT508 | OR694815 | AgNosCd-1 male x *An. coluzzii* Mopti female | Rep2 | F1 | ***Black****/****CFP+*** | Male | Drive/WT |
| MT509 | OR694816 | AgNosCd-1 male x *An. coluzzii* Mopti female | Rep2 | F1 | ***Black****/****CFP+*** | Male | Drive/WT |
| MT510 | OR694817 | AgNosCd-1 male x *An. coluzzii* Mopti female | Rep2 | F1 | ***Black****/****CFP+*** | Male | Drive/WT |
| MT511 | OR694818 | AgNosCd-1 male x *An. coluzzii* Mopti female | Rep2 | F1 | ***Black****/****CFP+*** | Female | Drive/WT |
| MT512 | OR694819 | AgNosCd-1 male x *An. coluzzii* Mopti female | Rep2 | F1 | ***Black****/****CFP+*** | Female | Drive/WT |
| MT513 | OR694820 | AgNosCd-1 male x *An. coluzzii* Mopti female | Rep2 | F1 | ***Black****/****CFP+*** | Female | Drive/WT |
| MT514 | OR694821 | AgNosCd-1 male x *An. coluzzii* Mopti female | Rep2 | F1 | ***Black****/****CFP+*** | Female | Drive/WT |
| MT515 | OR694822 | AgNosCd-1 male x *An. coluzzii* Mopti female | Rep2 | F1 | ***Black****/****CFP+*** | Female | Drive/WT |
| MT516 | OR694823 | AgNosCd-1 male x *An. coluzzii* Mopti female | Rep2 | F1 | ***Black****/****CFP+*** | Female | Drive/WT |
| MT517 | OR694824 | AgNosCd-1 male x *An. coluzzii* Mopti female | Rep2 | F1 | ***Black****/****CFP+*** | Female | Drive/WT |
| MT518 | OR694825 | AgNosCd-1 male x *An. coluzzii* Mopti female | Rep2 | F1 | ***Black****/****CFP+*** | Female | Drive/WT |
| MT519 | OR694826 | AgNosCd-1 male x *An. coluzzii* Mopti female | Rep2 | F1 | ***Black****/****CFP+*** | Female | Drive/WT |
| MT520 | OR694827 | AgNosCd-1 male x *An. coluzzii* Mopti female | Rep2 | F1 | ***Black****/****CFP+*** | Female | Drive/WT |
| MT521 | OR694828 | AgNosCd-1 male x *An. coluzzii* Mopti female | Rep3 | F1 | ***Black****/****CFP+*** | Male | Drive/WT |
| MT522 | OR694829 | AgNosCd-1 male x *An. coluzzii* Mopti female | Rep3 | F1 | ***Black****/****CFP+*** | Male | Drive/WT |
| MT523 | OR694830 | AgNosCd-1 male x *An. coluzzii* Mopti female | Rep3 | F1 | ***Black****/****CFP+*** | Male | Drive/WT |
| MT524 | OR694831 | AgNosCd-1 male x *An. coluzzii* Mopti female | Rep3 | F1 | ***Black****/****CFP+*** | Male | Drive/WT |
| MT525 | OR694832 | AgNosCd-1 male x *An. coluzzii* Mopti female | Rep3 | F1 | ***Black****/****CFP+*** | Male | Drive/WT |
| MT526 | OR694833 | AgNosCd-1 male x *An. coluzzii* Mopti female | Rep3 | F1 | ***Black****/****CFP+*** | Male | Drive/WT |
| MT527 | OR694834 | AgNosCd-1 male x *An. coluzzii* Mopti female | Rep3 | F1 | ***Black****/****CFP+*** | Male | Drive/WT |
| MT528 | OR694835 | AgNosCd-1 male x *An. coluzzii* Mopti female | Rep3 | F1 | ***Black****/****CFP+*** | Male | Drive/WT |
| MT529 | OR694836 | AgNosCd-1 male x *An. coluzzii* Mopti female | Rep3 | F1 | ***Black****/****CFP+*** | Male | Drive/WT |
| MT530 | OR694837 | AgNosCd-1 male x *An. coluzzii* Mopti female | Rep3 | F1 | ***Black****/****CFP+*** | Male | Drive/WT |
| MT531 | OR694838 | AgNosCd-1 male x *An. coluzzii* Mopti female | Rep3 | F1 | ***Black****/****CFP+*** | Female | Drive/WT |
| MT532 | OR694839 | AgNosCd-1 male x *An. coluzzii* Mopti female | Rep3 | F1 | ***Black****/****CFP+*** | Female | Drive/WT |
| MT533 | OR694840 | AgNosCd-1 male x *An. coluzzii* Mopti female | Rep3 | F1 | ***Black****/****CFP+*** | Female | Drive/WT |
| MT534 | OR694841 | AgNosCd-1 male x *An. coluzzii* Mopti female | Rep3 | F1 | ***Black****/****CFP+*** | Female | Drive/WT |
| MT535 | OR694842 | AgNosCd-1 male x *An. coluzzii* Mopti female | Rep3 | F1 | ***Black****/****CFP+*** | Female | Drive/WT |
| MT536 | OR694843 | AgNosCd-1 male x *An. coluzzii* Mopti female | Rep3 | F1 | ***Black****/****CFP+*** | Female | Drive/WT |
| MT537 | OR694844 | AgNosCd-1 male x *An. coluzzii* Mopti female | Rep3 | F1 | ***Black****/****CFP+*** | Female | Drive/WT |
| MT538 | OR694845 | AgNosCd-1 male x *An. coluzzii* Mopti female | Rep3 | F1 | ***Black****/****CFP+*** | Female | Drive/WT |
| MT539 | OR694846 | AgNosCd-1 male x *An. coluzzii* Mopti female | Rep3 | F1 | ***Black****/****CFP+*** | Female | Drive/WT |
| MT540 | OR694847 | AgNosCd-1 male x *An. coluzzii* Mopti female | Rep3 | F1 | ***Black****/****CFP+*** | Female | Drive/WT |
| MT265 | OR694699 | AgNosCd-1 male/*An. coluzzii* Mopti female hybrid Intercross | Rep3 | F2 | ***Black****/****CFP+*** | Male | Drive/WT |
| MT266 | OR694700 | AgNosCd-1 male/*An. coluzzii* Mopti female hybrid Intercross | Rep3 | F2 | ***Black****/****CFP+*** | Male | Drive/WT |
| MT267 | OR694701 | AgNosCd-1 male/*An. coluzzii* Mopti female hybrid Intercross | Rep3 | F2 | ***Black****/****CFP+*** | Male | Drive/WT |
| MT268 | OR694702 | AgNosCd-1 male/*An. coluzzii* Mopti female hybrid Intercross | Rep2 | F2 | ***Black****/****CFP+*** | Male | Drive/WT |
| MT269 | OR694703 | AgNosCd-1 male/*An. coluzzii* Mopti female hybrid Intercross | Rep2 | F2 | ***Black****/****CFP+*** | Male | Drive/WT |
| MT270 | OR694704 | AgNosCd-1 male/*An. coluzzii* Mopti female hybrid Intercross | Rep2 | F2 | ***Black****/****CFP+*** | Female | Drive/WT |
| MT271 | OR694705 | AgNosCd-1 male/*An. coluzzii* Mopti female hybrid Intercross | Rep2 | F2 | ***Black****/****CFP+*** | Female | Drive/WT |
| MT272 | OR694706 | AgNosCd-1 male/*An. coluzzii* Mopti female hybrid Intercross | Rep1 | F2 | ***Black****/****CFP+*** | Female | Drive/WT |
| MT241 | OR694675 | *An. coluzzii* Mopti male/AgNosCd-1 female hybrid Intercross | Rep1 | F2 | ***Black****/****CFP+*** | Male | Drive/NHEJ |
| MT242 | OR694676 | *An. coluzzii* Mopti male/AgNosCd-1 female hybrid Intercross | Rep1 | F2 | ***Black****/****CFP+*** | Male | Drive/WT |
| MT243 | OR694677 | *An. coluzzii* Mopti male/AgNosCd-1 female hybrid Intercross | Rep1 | F2 | ***Black****/****CFP+*** | Male | Drive/WT |
| MT244 | OR694678 | *An. coluzzii* Mopti male/AgNosCd-1 female hybrid Intercross | Rep1 | F2 | ***Black****/****CFP+*** | Male | Drive/WT |
| MT245 | OR694679 | *An. coluzzii* Mopti male/AgNosCd-1 female hybrid Intercross | Rep1 | F2 | ***Black****/****CFP+*** | Female | Drive/NHEJ |
| MT246 | OR694680 | *An. coluzzii* Mopti male/AgNosCd-1 female hybrid Intercross | Rep1 | F2 | ***Black****/****CFP+*** | Female | Drive/WT |
| MT247 | OR694681 | *An. coluzzii* Mopti male/AgNosCd-1 female hybrid Intercross | Rep1 | F2 | ***Black****/****CFP+*** | Female | Drive/NHEJ |
| MT248 | OR694682 | *An. coluzzii* Mopti male/AgNosCd-1 female hybrid Intercross | Rep1 | F2 | ***Black****/****CFP+*** | Female | Drive/NHEJ |
| MT249 | OR694683 | *An. coluzzii* Mopti male/AgNosCd-1 female hybrid Intercross | Rep2 | F2 | ***Black****/****CFP+*** | Male | Drive/NHEJ |
| MT250 | OR694684 | *An. coluzzii* Mopti male/AgNosCd-1 female hybrid Intercross | Rep2 | F2 | ***Black****/****CFP+*** | Male | Drive/NHEJ |
| MT251 | OR694685 | *An. coluzzii* Mopti male/AgNosCd-1 female hybrid Intercross | Rep2 | F2 | ***Black****/****CFP+*** | Female | Drive/NHEJ |
| MT252 | OR694686 | *An. coluzzii* Mopti male/AgNosCd-1 female hybrid Intercross | Rep2 | F2 | ***Black****/****CFP+*** | Female | Drive/NHEJ |
| MT253 | OR694687 | *An. coluzzii* Mopti male/AgNosCd-1 female hybrid Intercross | Rep2 | F2 | ***Black****/****CFP+*** | Female | Drive/WT |
| MT254 | OR694688 | *An. coluzzii* Mopti male/AgNosCd-1 female hybrid Intercross | Rep2 | F2 | ***Black****/****CFP+*** | Female | Drive/NHEJ |
| MT255 | OR694689 | *An. coluzzii* Mopti male/AgNosCd-1 female hybrid Intercross | Rep2 | F2 | ***Black****/****CFP+*** | Female | Drive/NHEJ |
| MT256 | OR694690 | *An. coluzzii* Mopti male/AgNosCd-1 female hybrid Intercross | Rep2 | F2 | ***Black****/****CFP+*** | Female | Drive/NHEJ |
| MT257 | OR694691 | *An. coluzzii* Mopti male/AgNosCd-1 female hybrid Intercross | Rep3 | F2 | ***Black****/****CFP+*** | Male | Drive/WT |
| MT258 | OR694692 | *An. coluzzii* Mopti male/AgNosCd-1 female hybrid Intercross | Rep3 | F2 | ***Black****/****CFP+*** | Female | Drive/NHEJ |
| MT259 | OR694693 | *An. coluzzii* Mopti male/AgNosCd-1 female hybrid Intercross | Rep3 | F2 | ***Black****/****CFP+*** | Female | Drive/WT |
| MT260 | OR694694 | *An. coluzzii* Mopti male/AgNosCd-1 female hybrid Intercross | Rep3 | F2 | ***Black****/****CFP+*** | Female | Drive/NHEJ |
| MT261 | OR694695 | *An. coluzzii* Mopti male/AgNosCd-1 female hybrid Intercross | Rep3 | F2 | ***Black****/****CFP+*** | Female | Drive/NHEJ |
| MT262 | OR694696 | *An. coluzzii* Mopti male/AgNosCd-1 female hybrid Intercross | Rep3 | F2 | ***Black****/****CFP+*** | Female | Drive/NHEJ |
| MT263 | OR694697 | *An. coluzzii* Mopti male/AgNosCd-1 female hybrid Intercross | Rep3 | F2 | ***Black****/****CFP+*** | Female | Drive/NHEJ |
| MT264 | OR694698 | *An. coluzzii* Mopti male/AgNosCd-1 female hybrid Intercross | Rep3 | F2 | ***Black****/****CFP+*** | Female | Drive/NHEJ |
| MT215 | OR694631 | *An. coluzzii* Mopti male/AgNosCd-1 female hybrid Intercross | Rep2 | F2 | ***Black****/CFP-* | Female | WT/NHEJ |
| MT216 | OR694632 | *An. coluzzii* Mopti male/AgNosCd-1 female hybrid Intercross | Rep2 | F2 | ***Black****/CFP-* | Female | WT/NHEJ |
| MT217 | OR694633 | *An. coluzzii* Mopti male/AgNosCd-1 female hybrid Intercross | Rep2 | F2 | ***Black****/CFP-* | Male | WT/NHEJ |
| MT218 | OR694634 | *An. coluzzii* Mopti male/AgNosCd-1 female hybrid Intercross | Rep2 | F2 | ***Black****/CFP-* | Male | WT/NHEJ |
| MT219 | OR694635 | *An. coluzzii* Mopti male/AgNosCd-1 female hybrid Intercross | Rep3 | F2 | ***Black****/CFP-* | Male | NHEJ/NHEJ |
|  | OR694636 |  |  |  |  |  |  |
| MT220 | OR694637 | *An. coluzzii* Mopti male/AgNosCd-1 female hybrid Intercross | Rep3 | F2 | ***Black****/CFP-* | Male | NHEJ/NHEJ |
|  | OR694638 |  |  |  |  |  |  |
| MT221 | OR694639 | *An. coluzzii* Mopti male/AgNosCd-1 female hybrid Intercross | Rep3 | F2 | ***Black****/CFP-* | Female | NHEJ/NHEJ |
|  | OR694640 |  |  |  |  |  |  |
| MT222 | OR694641 | *An. coluzzii* Mopti male/AgNosCd-1 female hybrid Intercross | Rep3 | F2 | ***Black****/CFP-* | Female | NHEJ/NHEJ |
|  | OR694642 |  |  |  |  |  |  |
| MT223 | OR694643 | *An. coluzzii* Mopti male/AgNosCd-1 female hybrid Intercross | Rep2 | F2 | *Cardinal/CFP-* | Female | NHEJ/NHEJ |
|  | OR694644 |  |  |  |  |  |  |
| MT224 | OR694645 | *An. coluzzii* Mopti male/AgNosCd-1 female hybrid Intercross | Rep2 | F2 | *Cardinal/CFP-* | Female | NHEJ/NHEJ |
|  | OR694646 |  |  |  |  |  |  |
| MT225 | OR694647 | *An. coluzzii* Mopti male/AgNosCd-1 female hybrid Intercross | Rep2 | F2 | *Cardinal/CFP-* | Male | NHEJ/NHEJ |
|  | OR694648 |  |  |  |  |  |  |
| MT226 | OR694649 | *An. coluzzii* Mopti male/AgNosCd-1 female hybrid Intercross | Rep2 | F2 | *Cardinal/CFP-* | Female | NHEJ/NHEJ |
|  | OR694650 |  |  |  |  |  |  |
| MT227 | OR694651 | *An. coluzzii* Mopti male/AgNosCd-1 female hybrid Intercross | Rep2 | F2 | *Cardinal/CFP-* | Female | NHEJ/NHEJ |
|  | OR694652 |  |  |  |  |  |  |
| MT228 | OR694653 | *An. coluzzii* Mopti male/AgNosCd-1 female hybrid Intercross | Rep2 | F2 | *Cardinal/CFP-* | Male | NHEJ/NHEJ |
|  | OR694654 |  |  |  |  |  |  |
| MT229 | OR694655 | *An. coluzzii* Mopti male/AgNosCd-1 female hybrid Intercross | Rep2 | F2 | *Cardinal/CFP-* | Female | NHEJ/NHEJ |
|  | OR694656 |  |  |  |  |  |  |
| MT230 | OR694657 | *An. coluzzii* Mopti male/AgNosCd-1 female hybrid Intercross | Rep3 | F2 | *Cardinal/CFP-* | Female | NHEJ/NHEJ |
|  | OR694658 |  |  |  |  |  |  |
| MT231 | OR694659 | *An. coluzzii* Mopti male/AgNosCd-1 female hybrid Intercross | Rep3 | F2 | *Cardinal/CFP-* | Female | NHEJ/NHEJ |
|  | OR694660 |  |  |  |  |  |  |
| MT232 | OR694661 | *An. coluzzii* Mopti male/AgNosCd-1 female hybrid Intercross | Rep3 | F2 | *Cardinal/CFP-* | Female | NHEJ/NHEJ |
|  | OR694662 |  |  |  |  |  |  |
| MT233 | OR694663 | *An. coluzzii* Mopti male/AgNosCd-1 female hybrid Intercross | Rep3 | F2 | *Cardinal/CFP-* | Female | NHEJ/NHEJ |
|  | OR694664 |  |  |  |  |  |  |
| MT234 | OR694665 | *An. coluzzii* Mopti male/AgNosCd-1 female hybrid Intercross | Rep3 | F2 | *Cardinal/CFP-* | Female | NHEJ/NHEJ |
|  | OR694666 |  |  |  |  |  |  |
| MT235 | OR694667 | *An. coluzzii* Mopti male/AgNosCd-1 female hybrid Intercross | Rep3 | F2 | *Cardinal/CFP-* | Female | NHEJ/NHEJ |
|  | OR694668 |  |  |  |  |  |  |
| MT236 | OR694669 | *An. coluzzii* Mopti male/AgNosCd-1 female hybrid Intercross | Rep3 | F2 | *Cardinal/CFP-* | Female | NHEJ/NHEJ |
|  | OR694670 |  |  |  |  |  |  |
| MT237 | OR694671 | *An. coluzzii* Mopti male/AgNosCd-1 female hybrid Intercross | Rep3 | F2 | *Cardinal/CFP-* | Female | NHEJ/NHEJ |
|  | OR694672 |  |  |  |  |  |  |
| MT238 | OR694673 | *An. coluzzii* Mopti male/AgNosCd-1 female hybrid Intercross | Rep3 | F2 | *Cardinal/CFP-* | Male | NHEJ/NHEJ |
|  | OR694674 |  |  |  |  |  |  |
| MT301 |  | AgNosCd-1 male/*An. coluzzii* Mopti female hybrid Intercross | Rep1 | F2 | *Cardinal/CFP+* | Male | Drive/Drive |
| MT302 |  | AgNosCd-1 male/*An. coluzzii* Mopti female hybrid Intercross | Rep1 | F2 | *Cardinal/CFP+* | Male | Drive/Drive |
| MT303 |  | AgNosCd-1 male/*An. coluzzii* Mopti female hybrid Intercross | Rep1 | F2 | *Cardinal/CFP+* | Male | Drive/Drive |
| MT304 |  | AgNosCd-1 male/*An. coluzzii* Mopti female hybrid Intercross | Rep1 | F2 | *Cardinal/CFP+* | Male | Drive/Drive |
| MT305 |  | AgNosCd-1 male/*An. coluzzii* Mopti female hybrid Intercross | Rep1 | F2 | *Cardinal/CFP+* | Male | Drive/Drive |
| MT306 |  | AgNosCd-1 male/*An. coluzzii* Mopti female hybrid Intercross | Rep1 | F2 | *Cardinal/CFP+* | Male | Drive/Drive |
| MT307 |  | AgNosCd-1 male/*An. coluzzii* Mopti female hybrid Intercross | Rep1 | F2 | *Cardinal/CFP+* | Male | Drive/Drive |
| MT308 |  | AgNosCd-1 male/*An. coluzzii* Mopti female hybrid Intercross | Rep1 | F2 | *Cardinal/CFP+* | Male | Drive/Drive |
| MT309 |  | AgNosCd-1 male/*An. coluzzii* Mopti female hybrid Intercross | Rep1 | F2 | *Cardinal/CFP+* | Male | Drive/Drive |
| MT310 |  | AgNosCd-1 male/*An. coluzzii* Mopti female hybrid Intercross | Rep1 | F2 | *Cardinal/CFP+* | Male | Drive/Drive |
| MT311 |  | AgNosCd-1 male/*An. coluzzii* Mopti female hybrid Intercross | Rep1 | F2 | *Cardinal/CFP+* | Female | Drive/Drive |
| MT312 |  | AgNosCd-1 male/*An. coluzzii* Mopti female hybrid Intercross | Rep1 | F2 | *Cardinal/CFP+* | Female | Drive/Drive |
| MT313 |  | AgNosCd-1 male/*An. coluzzii* Mopti female hybrid Intercross | Rep1 | F2 | *Cardinal/CFP+* | Female | Drive/Drive |
| MT314 |  | AgNosCd-1 male/*An. coluzzii* Mopti female hybrid Intercross | Rep1 | F2 | *Cardinal/CFP+* | Female | Drive/Drive |
| MT315 |  | AgNosCd-1 male/*An. coluzzii* Mopti female hybrid Intercross | Rep1 | F2 | *Cardinal/CFP+* | Female | Drive/Drive |
| MT316 |  | AgNosCd-1 male/*An. coluzzii* Mopti female hybrid Intercross | Rep1 | F2 | *Cardinal/CFP+* | Female | Drive/Drive |
| MT317 |  | AgNosCd-1 male/*An. coluzzii* Mopti female hybrid Intercross | Rep1 | F2 | *Cardinal/CFP+* | Female | Drive/Drive |
| MT318 |  | AgNosCd-1 male/*An. coluzzii* Mopti female hybrid Intercross | Rep1 | F2 | *Cardinal/CFP+* | Female | Drive/Drive |
| MT319 |  | AgNosCd-1 male/*An. coluzzii* Mopti female hybrid Intercross | Rep1 | F2 | *Cardinal/CFP+* | Female | Drive/Drive |
| MT320 |  | AgNosCd-1 male/*An. coluzzii* Mopti female hybrid Intercross | Rep1 | F2 | *Cardinal/CFP+* | Female | Drive/Drive |
| MT321 |  | AgNosCd-1 male/*An. coluzzii* Mopti female hybrid Intercross | Rep2 | F2 | *Cardinal/CFP+* | Male | Drive/Drive |
| MT322 |  | AgNosCd-1 male/*An. coluzzii* Mopti female hybrid Intercross | Rep2 | F2 | *Cardinal/CFP+* | Male | Drive/Drive |
| MT323 |  | AgNosCd-1 male/*An. coluzzii* Mopti female hybrid Intercross | Rep2 | F2 | *Cardinal/CFP+* | Male | Drive/Drive |
| MT324 |  | AgNosCd-1 male/*An. coluzzii* Mopti female hybrid Intercross | Rep2 | F2 | *Cardinal/CFP+* | Male | Drive/Drive |
| MT325 |  | AgNosCd-1 male/*An. coluzzii* Mopti female hybrid Intercross | Rep2 | F2 | *Cardinal/CFP+* | Male | Drive/Drive |
| MT326 |  | AgNosCd-1 male/*An. coluzzii* Mopti female hybrid Intercross | Rep2 | F2 | *Cardinal/CFP+* | Male | Drive/Drive |
| MT327 |  | AgNosCd-1 male/*An. coluzzii* Mopti female hybrid Intercross | Rep2 | F2 | *Cardinal/CFP+* | Male | Drive/Drive |
| MT328 |  | AgNosCd-1 male/*An. coluzzii* Mopti female hybrid Intercross | Rep2 | F2 | *Cardinal/CFP+* | Male | Drive/Drive |
| MT329 |  | AgNosCd-1 male/*An. coluzzii* Mopti female hybrid Intercross | Rep2 | F2 | *Cardinal/CFP+* | Male | Drive/Drive |
| MT330 |  | AgNosCd-1 male/*An. coluzzii* Mopti female hybrid Intercross | Rep2 | F2 | *Cardinal/CFP+* | Male | Drive/Drive |
| MT331 |  | AgNosCd-1 male/*An. coluzzii* Mopti female hybrid Intercross | Rep2 | F2 | *Cardinal/CFP+* | Female | Drive/Drive |
| MT332 |  | AgNosCd-1 male/*An. coluzzii* Mopti female hybrid Intercross | Rep2 | F2 | *Cardinal/CFP+* | Female | Drive/Drive |
| MT333 |  | AgNosCd-1 male/*An. coluzzii* Mopti female hybrid Intercross | Rep2 | F2 | *Cardinal/CFP+* | Female | Drive/Drive |
| MT334 |  | AgNosCd-1 male/*An. coluzzii* Mopti female hybrid Intercross | Rep2 | F2 | *Cardinal/CFP+* | Female | Drive/Drive |
| MT335 |  | AgNosCd-1 male/*An. coluzzii* Mopti female hybrid Intercross | Rep2 | F2 | *Cardinal/CFP+* | Female | Drive/Drive |
| MT336 |  | AgNosCd-1 male/*An. coluzzii* Mopti female hybrid Intercross | Rep2 | F2 | *Cardinal/CFP+* | Female | Drive/Drive |
| MT337 |  | AgNosCd-1 male/*An. coluzzii* Mopti female hybrid Intercross | Rep2 | F2 | *Cardinal/CFP+* | Female | Drive/Drive |
| MT338 |  | AgNosCd-1 male/*An. coluzzii* Mopti female hybrid Intercross | Rep2 | F2 | *Cardinal/CFP+* | Female | Drive/Drive |
| MT339 |  | AgNosCd-1 male/*An. coluzzii* Mopti female hybrid Intercross | Rep2 | F2 | *Cardinal/CFP+* | Female | Drive/Drive |
| MT340 |  | AgNosCd-1 male/*An. coluzzii* Mopti female hybrid Intercross | Rep2 | F2 | *Cardinal/CFP+* | Female | Drive/Drive |
| MT341 |  | AgNosCd-1 male/*An. coluzzii* Mopti female hybrid Intercross | Rep3 | F2 | *Cardinal/CFP+* | Male | Drive/Drive |
| MT342 |  | AgNosCd-1 male/*An. coluzzii* Mopti female hybrid Intercross | Rep3 | F2 | *Cardinal/CFP+* | Male | Drive/Drive |
| MT343 |  | AgNosCd-1 male/*An. coluzzii* Mopti female hybrid Intercross | Rep3 | F2 | *Cardinal/CFP+* | Male | Drive/Drive |
| MT344 |  | AgNosCd-1 male/*An. coluzzii* Mopti female hybrid Intercross | Rep3 | F2 | *Cardinal/CFP+* | Male | Drive/Drive |
| MT345 |  | AgNosCd-1 male/*An. coluzzii* Mopti female hybrid Intercross | Rep3 | F2 | *Cardinal/CFP+* | Male | Drive/Drive |
| MT346 |  | AgNosCd-1 male/*An. coluzzii* Mopti female hybrid Intercross | Rep3 | F2 | *Cardinal/CFP+* | Male | Drive/Drive |
| MT347 |  | AgNosCd-1 male/*An. coluzzii* Mopti female hybrid Intercross | Rep3 | F2 | *Cardinal/CFP+* | Male | Drive/Drive |
| MT348 |  | AgNosCd-1 male/*An. coluzzii* Mopti female hybrid Intercross | Rep3 | F2 | *Cardinal/CFP+* | Male | Drive/Drive |
| MT349 |  | AgNosCd-1 male/*An. coluzzii* Mopti female hybrid Intercross | Rep3 | F2 | *Cardinal/CFP+* | Male | Drive/Drive |
| MT350 |  | AgNosCd-1 male/*An. coluzzii* Mopti female hybrid Intercross | Rep3 | F2 | *Cardinal/CFP+* | Male | Drive/Drive |
| MT351 |  | AgNosCd-1 male/*An. coluzzii* Mopti female hybrid Intercross | Rep3 | F2 | *Cardinal/CFP+* | Female | Drive/Drive |
| MT352 |  | AgNosCd-1 male/*An. coluzzii* Mopti female hybrid Intercross | Rep3 | F2 | *Cardinal/CFP+* | Female | Drive/Drive |
| MT353 |  | AgNosCd-1 male/*An. coluzzii* Mopti female hybrid Intercross | Rep3 | F2 | *Cardinal/CFP+* | Female | Drive/Drive |
| MT354 |  | AgNosCd-1 male/*An. coluzzii* Mopti female hybrid Intercross | Rep3 | F2 | *Cardinal/CFP+* | Female | Drive/Drive |
| MT355 |  | AgNosCd-1 male/*An. coluzzii* Mopti female hybrid Intercross | Rep3 | F2 | *Cardinal/CFP+* | Female | Drive/Drive |
| MT356 |  | AgNosCd-1 male/*An. coluzzii* Mopti female hybrid Intercross | Rep3 | F2 | *Cardinal/CFP+* | Female | Drive/Drive |
| MT357 |  | AgNosCd-1 male/*An. coluzzii* Mopti female hybrid Intercross | Rep3 | F2 | *Cardinal/CFP+* | Female | Drive/Drive |
| MT358 |  | AgNosCd-1 male/*An. coluzzii* Mopti female hybrid Intercross | Rep3 | F2 | *Cardinal/CFP+* | Female | Drive/Drive |
| MT359 |  | AgNosCd-1 male/*An. coluzzii* Mopti female hybrid Intercross | Rep3 | F2 | *Cardinal/CFP+* | Female | Drive/Drive |
| MT360 |  | AgNosCd-1 male/*An. coluzzii* Mopti female hybrid Intercross | Rep3 | F2 | *Cardinal/CFP+* | Female | Drive/Drive |
| MT361 |  | *An. coluzzii* Mopti male/AgNosCd-1 female hybrid Intercross | Rep1 | F2 | *Cardinal/CFP+* | Male | Drive/Drive |
| MT362 |  | *An. coluzzii* Mopti male/AgNosCd-1 female hybrid Intercross | Rep1 | F2 | *Cardinal/CFP+* | Male | Drive/Drive |
| MT363 |  | *An. coluzzii* Mopti male/AgNosCd-1 female hybrid Intercross | Rep1 | F2 | *Cardinal/CFP+* | Male | Drive/Drive |
| MT364 |  | *An. coluzzii* Mopti male/AgNosCd-1 female hybrid Intercross | Rep1 | F2 | *Cardinal/CFP+* | Male | Drive/Drive |
| MT365 |  | *An. coluzzii* Mopti male/AgNosCd-1 female hybrid Intercross | Rep1 | F2 | *Cardinal/CFP+* | Male | Drive/Drive |
| MT366 |  | *An. coluzzii* Mopti male/AgNosCd-1 female hybrid Intercross | Rep1 | F2 | *Cardinal/CFP+* | Male | Drive/Drive |
| MT367 | OR694707 | *An. coluzzii* Mopti male/AgNosCd-1 female hybrid Intercross | Rep1 | F2 | *Cardinal/CFP+* | Male | Drive/NHEJ |
| MT368 | OR694708 | *An. coluzzii* Mopti male/AgNosCd-1 female hybrid Intercross | Rep1 | F2 | *Cardinal/CFP+* | Male | Drive/NHEJ |
| MT369 | OR694709 | *An. coluzzii* Mopti male/AgNosCd-1 female hybrid Intercross | Rep1 | F2 | *Cardinal/CFP+* | Male | Drive/NHEJ |
| MT370 |  | *An. coluzzii* Mopti male/AgNosCd-1 female hybrid Intercross | Rep1 | F2 | *Cardinal/CFP+* | Male | Drive/Drive |
| MT371 |  | *An. coluzzii* Mopti male/AgNosCd-1 female hybrid Intercross | Rep1 | F2 | *Cardinal/CFP+* | Female | Drive/Drive |
| MT372 |  | *An. coluzzii* Mopti male/AgNosCd-1 female hybrid Intercross | Rep1 | F2 | *Cardinal/CFP+* | Female | Drive/Drive |
| MT373 |  | *An. coluzzii* Mopti male/AgNosCd-1 female hybrid Intercross | Rep1 | F2 | *Cardinal/CFP+* | Female | Drive/Drive |
| MT374 |  | *An. coluzzii* Mopti male/AgNosCd-1 female hybrid Intercross | Rep1 | F2 | *Cardinal/CFP+* | Female | Drive/Drive |
| MT375 |  | *An. coluzzii* Mopti male/AgNosCd-1 female hybrid Intercross | Rep1 | F2 | *Cardinal/CFP+* | Female | Drive/Drive |
| MT376 |  | *An. coluzzii* Mopti male/AgNosCd-1 female hybrid Intercross | Rep1 | F2 | *Cardinal/CFP+* | Female | Drive/Drive |
| MT377 |  | *An. coluzzii* Mopti male/AgNosCd-1 female hybrid Intercross | Rep1 | F2 | *Cardinal/CFP+* | Female | Drive/Drive |
| MT378 |  | *An. coluzzii* Mopti male/AgNosCd-1 female hybrid Intercross | Rep1 | F2 | *Cardinal/CFP+* | Female | Drive/Drive |
| MT379 |  | *An. coluzzii* Mopti male/AgNosCd-1 female hybrid Intercross | Rep1 | F2 | *Cardinal/CFP+* | Female | Drive/Drive |
| MT380 |  | *An. coluzzii* Mopti male/AgNosCd-1 female hybrid Intercross | Rep1 | F2 | *Cardinal/CFP+* | Female | Drive/Drive |
| MT381 |  | *An. coluzzii* Mopti male/AgNosCd-1 female hybrid Intercross | Rep2 | F2 | *Cardinal/CFP+* | Male | Drive/Drive |
| MT382 |  | *An. coluzzii* Mopti male/AgNosCd-1 female hybrid Intercross | Rep2 | F2 | *Cardinal/CFP+* | Male | Drive/Drive |
| MT383 |  | *An. coluzzii* Mopti male/AgNosCd-1 female hybrid Intercross | Rep2 | F2 | *Cardinal/CFP+* | Male | Drive/Drive |
| MT384 |  | *An. coluzzii* Mopti male/AgNosCd-1 female hybrid Intercross | Rep2 | F2 | *Cardinal/CFP+* | Male | Drive/Drive |
| MT385 |  | *An. coluzzii* Mopti male/AgNosCd-1 female hybrid Intercross | Rep2 | F2 | *Cardinal/CFP+* | Male | Drive/Drive |
| MT386 |  | *An. coluzzii* Mopti male/AgNosCd-1 female hybrid Intercross | Rep2 | F2 | *Cardinal/CFP+* | Male | Drive/Drive |
| MT387 |  | *An. coluzzii* Mopti male/AgNosCd-1 female hybrid Intercross | Rep2 | F2 | *Cardinal/CFP+* | Male | Drive/Drive |
| MT388 |  | *An. coluzzii* Mopti male/AgNosCd-1 female hybrid Intercross | Rep2 | F2 | *Cardinal/CFP+* | Male | Drive/Drive |
| MT389 |  | *An. coluzzii* Mopti male/AgNosCd-1 female hybrid Intercross | Rep2 | F2 | *Cardinal/CFP+* | Male | Drive/Drive |
| MT390 |  | *An. coluzzii* Mopti male/AgNosCd-1 female hybrid Intercross | Rep2 | F2 | *Cardinal/CFP+* | Male | Drive/Drive |
| MT391 |  | *An. coluzzii* Mopti male/AgNosCd-1 female hybrid Intercross | Rep2 | F2 | *Cardinal/CFP+* | Female | Drive/Drive |
| MT392 |  | *An. coluzzii* Mopti male/AgNosCd-1 female hybrid Intercross | Rep2 | F2 | *Cardinal/CFP+* | Female | Drive/Drive |
| MT393 |  | *An. coluzzii* Mopti male/AgNosCd-1 female hybrid Intercross | Rep2 | F2 | *Cardinal/CFP+* | Female | Drive/Drive |
| MT394 | OR694710 | *An. coluzzii* Mopti male/AgNosCd-1 female hybrid Intercross | Rep2 | F2 | *Cardinal/CFP+* | Female | Drive/NHEJ |
| MT395 |  | *An. coluzzii* Mopti male/AgNosCd-1 female hybrid Intercross | Rep2 | F2 | *Cardinal/CFP+* | Female | Drive/Drive |
| MT396 |  | *An. coluzzii* Mopti male/AgNosCd-1 female hybrid Intercross | Rep2 | F2 | *Cardinal/CFP+* | Female | Drive/Drive |
| MT397 |  | *An. coluzzii* Mopti male/AgNosCd-1 female hybrid Intercross | Rep2 | F2 | *Cardinal/CFP+* | Female | Drive/Drive |
| MT398 |  | *An. coluzzii* Mopti male/AgNosCd-1 female hybrid Intercross | Rep2 | F2 | *Cardinal/CFP+* | Female | Drive/Drive |
| MT399 |  | *An. coluzzii* Mopti male/AgNosCd-1 female hybrid Intercross | Rep2 | F2 | *Cardinal/CFP+* | Female | Drive/Drive |
| MT400 |  | *An. coluzzii* Mopti male/AgNosCd-1 female hybrid Intercross | Rep2 | F2 | *Cardinal/CFP+* | Female | Drive/Drive |
| MT401 |  | *An. coluzzii* Mopti male/AgNosCd-1 female hybrid Intercross | Rep3 | F2 | *Cardinal/CFP+* | Male | Drive/Drive |
| MT402 |  | *An. coluzzii* Mopti male/AgNosCd-1 female hybrid Intercross | Rep3 | F2 | *Cardinal/CFP+* | Male | Drive/Drive |
| MT403 |  | *An. coluzzii* Mopti male/AgNosCd-1 female hybrid Intercross | Rep3 | F2 | *Cardinal/CFP+* | Male | Drive/Drive |
| MT404 |  | *An. coluzzii* Mopti male/AgNosCd-1 female hybrid Intercross | Rep3 | F2 | *Cardinal/CFP+* | Male | Drive/Drive |
| MT405 | OR694711 | *An. coluzzii* Mopti male/AgNosCd-1 female hybrid Intercross | Rep3 | F2 | *Cardinal/CFP+* | Male | Drive/NHEJ |
| MT406 |  | *An. coluzzii* Mopti male/AgNosCd-1 female hybrid Intercross | Rep3 | F2 | *Cardinal/CFP+* | Male | Drive/Drive |
| MT407 |  | *An. coluzzii* Mopti male/AgNosCd-1 female hybrid Intercross | Rep3 | F2 | *Cardinal/CFP+* | Male | Drive/Drive |
| MT408 |  | *An. coluzzii* Mopti male/AgNosCd-1 female hybrid Intercross | Rep3 | F2 | *Cardinal/CFP+* | Male | Drive/Drive |
| MT409 |  | *An. coluzzii* Mopti male/AgNosCd-1 female hybrid Intercross | Rep3 | F2 | *Cardinal/CFP+* | Male | Drive/Drive |
| MT410 |  | *An. coluzzii* Mopti male/AgNosCd-1 female hybrid Intercross | Rep3 | F2 | *Cardinal/CFP+* | Male | Drive/Drive |
| MT411 |  | *An. coluzzii* Mopti male/AgNosCd-1 female hybrid Intercross | Rep3 | F2 | *Cardinal/CFP+* | Female | Drive/Drive |
| MT412 | OR694712 | *An. coluzzii* Mopti male/AgNosCd-1 female hybrid Intercross | Rep3 | F2 | *Cardinal/CFP+* | Female | Drive/NHEJ |
| MT413 | OR694713 | *An. coluzzii* Mopti male/AgNosCd-1 female hybrid Intercross | Rep3 | F2 | *Cardinal/CFP+* | Female | Drive/NHEJ |
| MT414 | OR694714 | *An. coluzzii* Mopti male/AgNosCd-1 female hybrid Intercross | Rep3 | F2 | *Cardinal/CFP+* | Female | Drive/Drive |
| MT415 | OR694715 | *An. coluzzii* Mopti male/AgNosCd-1 female hybrid Intercross | Rep3 | F2 | *Cardinal/CFP+* | Female | Drive/NHEJ |
| MT416 |  | *An. coluzzii* Mopti male/AgNosCd-1 female hybrid Intercross | Rep3 | F2 | *Cardinal/CFP+* | Female | Drive/NHEJ |
| MT417 |  | *An. coluzzii* Mopti male/AgNosCd-1 female hybrid Intercross | Rep3 | F2 | *Cardinal/CFP+* | Female | Drive/Drive |
| MT418 | OR694716 | *An. coluzzii* Mopti male/AgNosCd-1 female hybrid Intercross | Rep3 | F2 | *Cardinal/CFP+* | Female | Drive/NHEJ |
| MT419 |  | *An. coluzzii* Mopti male/AgNosCd-1 female hybrid Intercross | Rep3 | F2 | *Cardinal/CFP+* | Female | Drive/Drive |
| MT420 | OR694717 | *An. coluzzii* Mopti male/AgNosCd-1 female hybrid Intercross | Rep3 | F2 | *Cardinal/CFP+* | Female | Drive/NHEJ |
| DGL41 | OR694848 | ***An. arabiensis* Dongola** male x **AgNosCd-1** female | Rep 1 | F1 | *Cardinal/CFP+* | Male | Drive/NHEJ |
| DGL42 | OR694849 | ***An. arabiensis* Dongola** male x **AgNosCd-1** female | Rep 1 | F1 | *Cardinal/CFP+* | Male | Drive/NHEJ |
|  | OR694850 |  |  |  |  |  |  |
| DGL43 | OR694851 | ***An. arabiensis* Dongola** male x **AgNosCd-1** female | Rep 1 | F1 | *Cardinal/CFP+* | Male | Drive/NHEJ |
| DGL44 | OR694852 | ***An. arabiensis* Dongola** male x **AgNosCd-1** female | Rep 1 | F1 | *Cardinal/CFP+* | Male | Drive/NHEJ |
|  | OR694853 |  |  |  |  |  |  |
| DGL45 | OR694854 | ***An. arabiensis* Dongola** male x **AgNosCd-1** female | Rep 1 | F1 | *Cardinal/CFP+* | Male | Drive/NHEJ |
|  | OR694855 |  |  |  |  |  |  |
| DGL46 | OR694856 | ***An. arabiensis* Dongola** male x **AgNosCd-1** female | Rep 1 | F1 | *Cardinal/CFP+* | Female | Drive/NHEJ |
| DGL47 | OR694857 | ***An. arabiensis* Dongola** male x **AgNosCd-1** female | Rep 1 | F1 | *Cardinal/CFP+* | Female | Drive/NHEJ |
| DGL48 | OR694858 | ***An. arabiensis* Dongola** male x **AgNosCd-1** female | Rep 1 | F1 | *Cardinal/CFP+* | Female | Drive/NHEJ |
| DGL49 | OR694859 | ***An. arabiensis* Dongola** male x **AgNosCd-1** female | Rep 1 | F1 | *Cardinal/CFP+* | Female | Drive/NHEJ |
| DGL50 | OR694860 | ***An. arabiensis* Dongola** male x **AgNosCd-1** female | Rep 1 | F1 | *Cardinal/CFP+* | Female | Drive/NHEJ |
|  | OR694861 |  |  |  |  |  |  |
| DGL51 | OR694862 | ***An. arabiensis* Dongola** male x **AgNosCd-1** female | Rep 1 | F1 | *Cardinal/CFP+* | Female | Drive/NHEJ |
| DGL52 | OR694863 | ***An. arabiensis* Dongola** male x **AgNosCd-1** female | Rep 1 | F1 | *Cardinal/CFP+* | Female | Drive/NHEJ |
| DGL53 | OR694864 | ***An. arabiensis* Dongola** male x **AgNosCd-1** female | Rep 1 | F1 | *Cardinal/CFP+* | Female | Drive/NHEJ |
|  | OR694865 |  |  |  |  |  |  |
| DGL54 | OR694866 | ***An. arabiensis* Dongola** male x **AgNosCd-1** female | Rep 1 | F1 | *Cardinal/CFP+* | Female | Drive/NHEJ |
| DGL55 | OR694867 | ***An. arabiensis* Dongola** male x **AgNosCd-1** female | Rep 1 | F1 | *Cardinal/CFP+* | Female | Drive/NHEJ |
| DGL56 | OR694868 | ***An. arabiensis* Dongola** male x **AgNosCd-1** female | Rep 1 | F1 | *Cardinal/CFP+* | Female | Drive/NHEJ |
| DGL57 | OR694869 | ***An. arabiensis* Dongola** male x **AgNosCd-1** female | Rep 1 | F1 | *Cardinal/CFP+* | Female | Drive/NHEJ |
|  | OR694870 |  |  |  |  |  |  |
| DGL58 | OR694871 | ***An. arabiensis* Dongola** male x **AgNosCd-1** female | Rep 1 | F1 | *Cardinal/CFP+* | Female | Drive/NHEJ |
| DGL59 | OR694872 | ***An. arabiensis* Dongola** male x **AgNosCd-1** female | Rep 1 | F1 | *Cardinal/CFP+* | Male | Drive/NHEJ |
| DGL60 | OR694873 | ***An. arabiensis* Dongola** male x **AgNosCd-1** female | Rep 1 | F1 | *Cardinal/CFP+* | Male | Drive/NHEJ |
|  | OR694874 |  |  |  |  |  |  |
| DGL61 | OR694875 | ***An. arabiensis* Dongola** male x **AgNosCd-1** female | Rep 1 | F1 | *Cardinal/CFP+* | Male | Drive/NHEJ |
| DGL62 | OR694876 | ***An. arabiensis* Dongola** male x **AgNosCd-1** female | Rep 1 | F1 | *Cardinal/CFP+* | Male | Drive/NHEJ |
|  | OR694877 |  |  |  |  |  |  |
| DGL103 | OR694878 | ***An. arabiensis* Dongola** male x **AgNosCd-1** female | Rep 2 | F1 | *Cardinal/CFP+* | Male | Drive/NHEJ |
|  | OR694879 |  |  |  |  |  |  |
| DGL104 | OR694880 | ***An. arabiensis* Dongola** male x **AgNosCd-1** female | Rep 2 | F1 | *Cardinal/CFP+* | Male | Drive/NHEJ |
| DGL105 | OR694881 | ***An. arabiensis* Dongola** male x **AgNosCd-1** female | Rep 2 | F1 | *Cardinal/CFP+* | Male | Drive/NHEJ |
|  | OR694882 |  |  |  |  |  |  |
| DGL106 | OR694883 | ***An. arabiensis* Dongola** male x **AgNosCd-1** female | Rep 2 | F1 | *Cardinal/CFP+* | Male | Drive/NHEJ |
|  | OR694884 |  |  |  |  |  |  |
| DGL107 | OR694885 | ***An. arabiensis* Dongola** male x **AgNosCd-1** female | Rep 2 | F1 | *Cardinal/CFP+* | Male | Drive/NHEJ |
|  | OR694886 |  |  |  |  |  |  |
| DGL108 | OR694887 | ***An. arabiensis* Dongola** male x **AgNosCd-1** female | Rep 2 | F1 | *Cardinal/CFP+* | Male | Drive/NHEJ |
| DGL109 | OR694888 | ***An. arabiensis* Dongola** male x **AgNosCd-1** female | Rep 2 | F1 | *Cardinal/CFP+* | Male | Drive/NHEJ |
| DGL110 | OR694889 | ***An. arabiensis* Dongola** male x **AgNosCd-1** female | Rep 2 | F1 | *Cardinal/CFP+* | Male | Drive/NHEJ |
|  | OR694890 |  |  |  |  |  |  |
| DGL111 | OR694891 | ***An. arabiensis* Dongola** male x **AgNosCd-1** female | Rep 2 | F1 | *Cardinal/CFP+* | Female | Drive/NHEJ |
|  | OR694892 |  |  |  |  |  |  |
| DGL112 | OR694893 | ***An. arabiensis* Dongola** male x **AgNosCd-1** female | Rep 2 | F1 | *Cardinal/CFP+* | Female | Drive/NHEJ |
|  | OR694894 |  |  |  |  |  |  |
| DGL113 | OR694895 | ***An. arabiensis* Dongola** male x **AgNosCd-1** female | Rep 2 | F1 | *Cardinal/CFP+* | Female | Drive/NHEJ |
|  | OR694896 |  |  |  |  |  |  |
| DGL114 | OR694897 | ***An. arabiensis* Dongola** male x **AgNosCd-1** female | Rep 2 | F1 | *Cardinal/CFP+* | Female | Drive/NHEJ |
| DGL115 | OR694898 | ***An. arabiensis* Dongola** male x **AgNosCd-1** female | Rep 2 | F1 | *Cardinal/CFP+* | Female | Drive/NHEJ |
|  | OR694899 |  |  |  |  |  |  |
| DGL116 | OR694900 | ***An. arabiensis* Dongola** male x **AgNosCd-1** female | Rep 2 | F1 | *Cardinal/CFP+* | Female | Drive/NHEJ |
|  | OR694901 |  |  |  |  |  |  |
| DGL117 | OR694902 | ***An. arabiensis* Dongola** male x **AgNosCd-1** female | Rep 2 | F1 | *Cardinal/CFP+* | Female | Drive/NHEJ |
|  | OR694903 |  |  |  |  |  |  |
| DGL118 | OR694904 | ***An. arabiensis* Dongola** male x **AgNosCd-1** female | Rep 2 | F1 | *Cardinal/CFP+* | Female | Drive/NHEJ |
| DGL119 | OR694905 | ***An. arabiensis* Dongola** male x **AgNosCd-1** female | Rep 2 | F1 | *Cardinal/CFP+* | Female | Drive/NHEJ |
|  | OR694906 |  |  |  |  |  |  |
| DGL120 | OR694907 | ***An. arabiensis* Dongola** male x **AgNosCd-1** female | Rep 2 | F1 | *Cardinal/CFP+* | Female | Drive/NHEJ |
|  | OR694908 |  |  |  |  |  |  |
| DGL121 | OR694909 | ***An. arabiensis* Dongola** male x **AgNosCd-1** female | Rep 2 | F1 | *Cardinal/CFP+* | Female | Drive/NHEJ |
|  | OR694910 |  |  |  |  |  |  |
| DGL122 | OR694911 | ***An. arabiensis* Dongola** male x **AgNosCd-1** female | Rep 2 | F1 | *Cardinal/CFP+* | Female | Drive/NHEJ |
|  | OR694912 |  |  |  |  |  |  |
| DGL123 | OR694913 | ***An. arabiensis* Dongola** male x **AgNosCd-1** female | Rep 2 | F1 | *Cardinal/CFP+* | Female | Drive/NHEJ |
| DGL154 | OR694914 | ***An. arabiensis* Dongola** male x **AgNosCd-1** female | Rep 3 | F1 | *Cardinal/CFP+* | Male | Drive/NHEJ |
| DGL155 | OR694915 | ***An. arabiensis* Dongola** male x **AgNosCd-1** female | Rep 3 | F1 | *Cardinal/CFP+* | Male | Drive/NHEJ |
| DGL156 | OR694916 | ***An. arabiensis* Dongola** male x **AgNosCd-1** female | Rep 3 | F1 | *Cardinal/CFP+* | Male | Drive/NHEJ |
|  | OR694917 |  |  |  |  |  |  |
| DGL157 | OR694918 | ***An. arabiensis* Dongola** male x **AgNosCd-1** female | Rep 3 | F1 | *Cardinal/CFP+* | Male | Drive/NHEJ |
|  | OR694919 |  |  |  |  |  |  |
| DGL158 | OR694920 | ***An. arabiensis* Dongola** male x **AgNosCd-1** female | Rep 3 | F1 | *Cardinal/CFP+* | Male | Drive/NHEJ |
| DGL159 | OR694921 | ***An. arabiensis* Dongola** male x **AgNosCd-1** female | Rep 3 | F1 | *Cardinal/CFP+* | Male | Drive/NHEJ |
| DGL160 | OR694922 | ***An. arabiensis* Dongola** male x **AgNosCd-1** female | Rep 3 | F1 | *Cardinal/CFP+* | Male | Drive/NHEJ |
|  | OR694923 |  |  |  |  |  |  |
| DGL161 | OR694924 | ***An. arabiensis* Dongola** male x **AgNosCd-1** female | Rep 3 | F1 | *Cardinal/CFP+* | Male | Drive/NHEJ |
| DGL162 | OR694925 | ***An. arabiensis* Dongola** male x **AgNosCd-1** female | Rep 3 | F1 | *Cardinal/CFP+* | Male | Drive/NHEJ |
|  | OR694926 |  |  |  |  |  |  |
| DGL163 | OR694927 | ***An. arabiensis* Dongola** male x **AgNosCd-1** female | Rep 3 | F1 | *Cardinal/CFP+* | Male | Drive/NHEJ |
| DGL164 | OR694928 | ***An. arabiensis* Dongola** male x **AgNosCd-1** female | Rep 3 | F1 | *Cardinal/CFP+* | Female | Drive/NHEJ |
| DGL165 | OR694929 | ***An. arabiensis* Dongola** male x **AgNosCd-1** female | Rep 3 | F1 | *Cardinal/CFP+* | Female | Drive/NHEJ |
|  | OR694930 |  |  |  |  |  |  |
| DGL166 | OR694931 | ***An. arabiensis* Dongola** male x **AgNosCd-1** female | Rep 3 | F1 | *Cardinal/CFP+* | Female |  |
| DGL167 | OR694932 | ***An. arabiensis* Dongola** male x **AgNosCd-1** female | Rep 3 | F1 | *Cardinal/CFP+* | Female | Drive/NHEJ |
| DGL168 | OR694933 | ***An. arabiensis* Dongola** male x **AgNosCd-1** female | Rep 3 | F1 | *Cardinal/CFP+* | Female | Drive/NHEJ |
|  | OR694934 |  |  |  |  |  |  |
| DGL169 | OR694935 | ***An. arabiensis* Dongola** male x **AgNosCd-1** female | Rep 3 | F1 | *Cardinal/CFP+* | Female | Drive/NHEJ |
| DGL170 | OR694936 | ***An. arabiensis* Dongola** male x **AgNosCd-1** female | Rep 3 | F1 | *Cardinal/CFP+* | Female | Drive/NHEJ |
| DGL301 | OR694937 | ***An. arabiensis* Dongola** male x **AgNosCd-1** female | Rep1 | F1 | ***Black****/****CFP+*** | Male | Drive/WT |
| DGL302 | OR694938 | ***An. arabiensis* Dongola** male x **AgNosCd-1** female | Rep1 | F1 | ***Black****/****CFP+*** | Male | Drive/WT |
| DGL303 | OR694939 | ***An. arabiensis* Dongola** male x **AgNosCd-1** female | Rep1 | F1 | ***Black****/****CFP+*** | Male | Drive/WT |
| DGL304 | OR694940 | ***An. arabiensis* Dongola** male x **AgNosCd-1** female | Rep1 | F1 | ***Black****/****CFP+*** | Male | Drive/NHEJ |
|  | OR694941 |  |  |  |  |  |  |
| DGL305 | OR694942 | ***An. arabiensis* Dongola** male x **AgNosCd-1** female | Rep1 | F1 | ***Black****/****CFP+*** | Male | Drive/WT |
| DGL306 | OR694943 | ***An. arabiensis* Dongola** male x **AgNosCd-1** female | Rep1 | F1 | ***Black****/****CFP+*** | Male | Drive/WT |
| DGL307 | OR694944 | ***An. arabiensis* Dongola** male x **AgNosCd-1** female | Rep1 | F1 | ***Black****/****CFP+*** | Male | Drive/WT |
| DGL308 | OR694945 | ***An. arabiensis* Dongola** male x **AgNosCd-1** female | Rep1 | F1 | ***Black****/****CFP+*** | Male | Drive/WT |
| DGL309 | OR694946 | ***An. arabiensis* Dongola** male x **AgNosCd-1** female | Rep1 | F1 | ***Black****/****CFP+*** | Male | Drive/WT |
| DGL310 | OR694947 | ***An. arabiensis* Dongola** male x **AgNosCd-1** female | Rep1 | F1 | ***Black****/****CFP+*** | Male | Drive/WT |
| DGL311 | OR694948 | ***An. arabiensis* Dongola** male x **AgNosCd-1** female | Rep1 | F1 | ***Black****/****CFP+*** | Female | Drive/WT |
| DGL312 | OR694949 | ***An. arabiensis* Dongola** male x **AgNosCd-1** female | Rep1 | F1 | ***Black****/****CFP+*** | Female | Drive/WT |
| DGL313 | OR694950 | ***An. arabiensis* Dongola** male x **AgNosCd-1** female | Rep1 | F1 | ***Black****/****CFP+*** | Female | Drive/WT |
| DGL314 | OR694951 | ***An. arabiensis* Dongola** male x **AgNosCd-1** female | Rep1 | F1 | ***Black****/****CFP+*** | Female | Drive/WT |
| DGL315 | OR694952 | ***An. arabiensis* Dongola** male x **AgNosCd-1** female | Rep1 | F1 | ***Black****/****CFP+*** | Female | Drive/WT |
| DGL316 | OR694953 | ***An. arabiensis* Dongola** male x **AgNosCd-1** female | Rep1 | F1 | ***Black****/****CFP+*** | Female | Drive/WT |
| DGL317 | OR694954 | ***An. arabiensis* Dongola** male x **AgNosCd-1** female | Rep1 | F1 | ***Black****/****CFP+*** | Female | Drive/WT |
| DGL318 | OR694955 | ***An. arabiensis* Dongola** male x **AgNosCd-1** female | Rep1 | F1 | ***Black****/****CFP+*** | Female | Drive/WT |
| DGL319 | OR694956 | ***An. arabiensis* Dongola** male x **AgNosCd-1** female | Rep1 | F1 | ***Black****/****CFP+*** | Female | Drive/WT |
| DGL320 | OR694957 | ***An. arabiensis* Dongola** male x **AgNosCd-1** female | Rep1 | F1 | ***Black****/****CFP+*** | Female | Drive/WT |
| DGL321 | OR694958 | ***An. arabiensis* Dongola** male x **AgNosCd-1** female | Rep2 | F1 | ***Black****/****CFP+*** | Male | Drive/NHEJ |
| DGL322 | OR694959 | ***An. arabiensis* Dongola** male x **AgNosCd-1** female | Rep2 | F1 | ***Black****/****CFP+*** | Male | Drive/WT |
| DGL323 | OR694960 | ***An. arabiensis* Dongola** male x **AgNosCd-1** female | Rep2 | F1 | ***Black****/****CFP+*** | Male | Drive/NHEJ |
|  | OR694961 |  |  |  |  |  |  |
| DGL324 | OR694962 | ***An. arabiensis* Dongola** male x **AgNosCd-1** female | Rep2 | F1 | ***Black****/****CFP+*** | Male | Drive/WT |
| DGL325 | OR694963 | ***An. arabiensis* Dongola** male x **AgNosCd-1** female | Rep2 | F1 | ***Black****/****CFP+*** | Male | Drive/WT |
| DGL326 | OR694964 | ***An. arabiensis* Dongola** male x **AgNosCd-1** female | Rep2 | F1 | ***Black****/****CFP+*** | Male | Drive/WT |
| DGL327 | OR694965 | ***An. arabiensis* Dongola** male x **AgNosCd-1** female | Rep2 | F1 | ***Black****/****CFP+*** | Male | Drive/WT |
| DGL328 | OR694966 | ***An. arabiensis* Dongola** male x **AgNosCd-1** female | Rep2 | F1 | ***Black****/****CFP+*** | Male | Drive/NHEJ |
| DGL329 | OR694967 | ***An. arabiensis* Dongola** male x **AgNosCd-1** female | Rep2 | F1 | ***Black****/****CFP+*** | Male | Drive/WT |
| DGL330 | OR694968 | ***An. arabiensis* Dongola** male x **AgNosCd-1** female | Rep2 | F1 | ***Black****/****CFP+*** | Male | Drive/WT |
| DGL331 | OR694969 | ***An. arabiensis* Dongola** male x **AgNosCd-1** female | Rep2 | F1 | ***Black****/****CFP+*** | Female | Drive/WT |
| DGL332 | OR694970 | ***An. arabiensis* Dongola** male x **AgNosCd-1** female | Rep2 | F1 | ***Black****/****CFP+*** | Female | Drive/NHEJ |
| DGL333 | OR694971 | ***An. arabiensis* Dongola** male x **AgNosCd-1** female | Rep2 | F1 | ***Black****/****CFP+*** | Female | Drive/WT |
| DGL334 | OR694972 | ***An. arabiensis* Dongola** male x **AgNosCd-1** female | Rep2 | F1 | ***Black****/****CFP+*** | Female | Drive/WT |
| DGL335 | OR694973 | ***An. arabiensis* Dongola** male x **AgNosCd-1** female | Rep2 | F1 | ***Black****/****CFP+*** | Female | Drive/WT |
| DGL336 | OR694974 | ***An. arabiensis* Dongola** male x **AgNosCd-1** female | Rep2 | F1 | ***Black****/****CFP+*** | Female | Drive/WT |
| DGL341 | OR694975 | ***An. arabiensis* Dongola** male x **AgNosCd-1** female | Rep3 | F1 | ***Black****/****CFP+*** | Male | Drive/WT |
| DGL342 | OR694976 | ***An. arabiensis* Dongola** male x **AgNosCd-1** female | Rep3 | F1 | ***Black****/****CFP+*** | Male | Drive/WT |
| DGL343 | OR694977 | ***An. arabiensis* Dongola** male x **AgNosCd-1** female | Rep3 | F1 | ***Black****/****CFP+*** | Male | Drive/WT |
| DGL344 | OR694978 | ***An. arabiensis* Dongola** male x **AgNosCd-1** female | Rep3 | F1 | ***Black****/****CFP+*** | Male | Drive/WT |
| DGL345 | OR694979 | ***An. arabiensis* Dongola** male x **AgNosCd-1** female | Rep3 | F1 | ***Black****/****CFP+*** | Male | Drive/NHEJ |
|  | OR694980 |  |  |  |  |  |  |
| DGL346 | OR694981 | ***An. arabiensis* Dongola** male x **AgNosCd-1** female | Rep3 | F1 | ***Black****/****CFP+*** | Male | Drive/WT |
| DGL347 | OR694982 | ***An. arabiensis* Dongola** male x **AgNosCd-1** female | Rep3 | F1 | ***Black****/****CFP+*** | Male | Drive/WT |
| DGL348 | OR694983 | ***An. arabiensis* Dongola** male x **AgNosCd-1** female | Rep3 | F1 | ***Black****/****CFP+*** | Male | Drive/WT |
| DGL349 | OR694984 | ***An. arabiensis* Dongola** male x **AgNosCd-1** female | Rep3 | F1 | ***Black****/****CFP+*** | Male | Drive/NHEJ |
| DGL350 | OR694985 | ***An. arabiensis* Dongola** male x **AgNosCd-1** female | Rep3 | F1 | ***Black****/****CFP+*** | Male | Drive/WT |
| DGL351 | OR694986 | ***An. arabiensis* Dongola** male x **AgNosCd-1** female | Rep3 | F1 | ***Black****/****CFP+*** | Female | Drive/WT |
| DGL352 | OR694987 | ***An. arabiensis* Dongola** male x **AgNosCd-1** female | Rep3 | F1 | ***Black****/****CFP+*** | Female | Drive/WT |
| DGL353 | OR694988 | ***An. arabiensis* Dongola** male x **AgNosCd-1** female | Rep3 | F1 | ***Black****/****CFP+*** | Female | Drive/NHEJ |
| DGL354 | OR694989 | ***An. arabiensis* Dongola** male x **AgNosCd-1** female | Rep3 | F1 | ***Black****/****CFP+*** | Female | Drive/WT |
| DGL355 | OR694990 | ***An. arabiensis* Dongola** male x **AgNosCd-1** female | Rep3 | F1 | ***Black****/****CFP+*** | Female | Drive/WT |
| DGL356 | OR694991 | ***An. arabiensis* Dongola** male x **AgNosCd-1** female | Rep3 | F1 | ***Black****/****CFP+*** | Female | Drive/WT |
| DGL357 | OR694992 | ***An. arabiensis* Dongola** male x **AgNosCd-1** female | Rep3 | F1 | ***Black****/****CFP+*** | Female | Drive/WT |
| DGL358 | OR694993 | ***An. arabiensis* Dongola** male x **AgNosCd-1** female | Rep3 | F1 | ***Black****/****CFP+*** | Female | Drive/WT |
| DGL359 | OR694994 | ***An. arabiensis* Dongola** male x **AgNosCd-1** female | Rep3 | F1 | ***Black****/****CFP+*** | Female | Drive/WT |
| DGL360 | OR694995 | ***An. arabiensis* Dongola** male x **AgNosCd-1** female | Rep3 | F1 | ***Black****/****CFP+*** | Female | Drive/WT |
| DGL361 | OR694996 | **AgNosCd-1** male x ***An. arabiensis*** **Dongola** female | Rep1 | F1 | ***Black****/****CFP+*** | Male | Drive/WT |
| DGL362 | OR694997 | **AgNosCd-1** male x ***An. arabiensis*** **Dongola** female | Rep1 | F1 | ***Black****/****CFP+*** | Male | Drive/WT |
| DGL363 | OR694998 | **AgNosCd-1** male x ***An. arabiensis*** **Dongola** female | Rep1 | F1 | ***Black****/****CFP+*** | Male | Drive/WT |
| DGL364 | OR694999 | **AgNosCd-1** male x ***An. arabiensis*** **Dongola** female | Rep1 | F1 | ***Black****/****CFP+*** | Male | Drive/WT |
| DGL365 | OR695000 | **AgNosCd-1** male x ***An. arabiensis*** **Dongola** female | Rep1 | F1 | ***Black****/****CFP+*** | Male | Drive/WT |
| DGL366 | OR695001 | **AgNosCd-1** male x ***An. arabiensis*** **Dongola** female | Rep1 | F1 | ***Black****/****CFP+*** | Male | Drive/WT |
| DGL367 | OR695002 | **AgNosCd-1** male x ***An. arabiensis*** **Dongola** female | Rep1 | F1 | ***Black****/****CFP+*** | Male | Drive/WT |
| DGL368 | OR695003 | **AgNosCd-1** male x ***An. arabiensis*** **Dongola** female | Rep1 | F1 | ***Black****/****CFP+*** | Male | Drive/WT |
| DGL369 | OR695004 | **AgNosCd-1** male x ***An. arabiensis*** **Dongola** female | Rep1 | F1 | ***Black****/****CFP+*** | Male | Drive/WT |
| DGL370 | OR695005 | **AgNosCd-1** male x ***An. arabiensis*** **Dongola** female | Rep1 | F1 | ***Black****/****CFP+*** | Male | Drive/WT |
| DGL371 | OR695006 | **AgNosCd-1** male x ***An. arabiensis*** **Dongola** female | Rep1 | F1 | ***Black****/****CFP+*** | Female | Drive/WT |
| DGL372 | OR695007 | **AgNosCd-1** male x ***An. arabiensis*** **Dongola** female | Rep1 | F1 | ***Black****/****CFP+*** | Female | Drive/WT |
| DGL373 | OR695008 | **AgNosCd-1** male x ***An. arabiensis*** **Dongola** female | Rep1 | F1 | ***Black****/****CFP+*** | Female | Drive/WT |
| DGL374 | OR695009 | **AgNosCd-1** male x ***An. arabiensis*** **Dongola** female | Rep1 | F1 | ***Black****/****CFP+*** | Female | Drive/WT |
| DGL375 | OR695010 | **AgNosCd-1** male x ***An. arabiensis*** **Dongola** female | Rep1 | F1 | ***Black****/****CFP+*** | Female | Drive/WT |
| DGL376 | OR695011 | **AgNosCd-1** male x ***An. arabiensis*** **Dongola** female | Rep1 | F1 | ***Black****/****CFP+*** | Female | Drive/WT |
| DGL377 | OR695012 | **AgNosCd-1** male x ***An. arabiensis*** **Dongola** female | Rep1 | F1 | ***Black****/****CFP+*** | Female | Drive/WT |
| DGL378 | OR695013 | **AgNosCd-1** male x ***An. arabiensis*** **Dongola** female | Rep1 | F1 | ***Black****/****CFP+*** | Female | Drive/WT |
| DGL379 | OR695014 | **AgNosCd-1** male x ***An. arabiensis*** **Dongola** female | Rep1 | F1 | ***Black****/****CFP+*** | Female | Drive/WT |
| DGL380 | OR695015 | **AgNosCd-1** male x ***An. arabiensis*** **Dongola** female | Rep1 | F1 | ***Black****/****CFP+*** | Female | Drive/WT |
| DGL381 | OR695016 | **AgNosCd-1** male x ***An. arabiensis*** **Dongola** female | Rep2 | F1 | ***Black****/****CFP+*** | Male | Drive/WT |
| DGL382 | OR695017 | **AgNosCd-1** male x ***An. arabiensis*** **Dongola** female | Rep2 | F1 | ***Black****/****CFP+*** | Male | Drive/WT |
| DGL383 | OR695018 | **AgNosCd-1** male x ***An. arabiensis*** **Dongola** female | Rep2 | F1 | ***Black****/****CFP+*** | Male | Drive/WT |
| DGL384 | OR695019 | **AgNosCd-1** male x ***An. arabiensis*** **Dongola** female | Rep2 | F1 | ***Black****/****CFP+*** | Male | Drive/WT |
| DGL385 | OR695020 | **AgNosCd-1** male x ***An. arabiensis*** **Dongola** female | Rep2 | F1 | ***Black****/****CFP+*** | Male | Drive/WT |
| DGL386 | OR695021 | **AgNosCd-1** male x ***An. arabiensis*** **Dongola** female | Rep2 | F1 | ***Black****/****CFP+*** | Male | Drive/WT |
| DGL387 | OR695022 | **AgNosCd-1** male x ***An. arabiensis*** **Dongola** female | Rep2 | F1 | ***Black****/****CFP+*** | Male | Drive/WT |
| DGL388 | OR695023 | **AgNosCd-1** male x ***An. arabiensis*** **Dongola** female | Rep2 | F1 | ***Black****/****CFP+*** | Male | Drive/WT |
| DGL389 | OR695024 | **AgNosCd-1** male x ***An. arabiensis*** **Dongola** female | Rep2 | F1 | ***Black****/****CFP+*** | Male | Drive/WT |
| DGL390 | OR695025 | **AgNosCd-1** male x ***An. arabiensis*** **Dongola** female | Rep2 | F1 | ***Black****/****CFP+*** | Male | Drive/WT |
| DGL391 | OR695026 | **AgNosCd-1** male x ***An. arabiensis*** **Dongola** female | Rep2 | F1 | ***Black****/****CFP+*** | Female | Drive/WT |
| DGL392 | OR695027 | **AgNosCd-1** male x ***An. arabiensis*** **Dongola** female | Rep2 | F1 | ***Black****/****CFP+*** | Female | Drive/WT |
| DGL393 | OR695028 | **AgNosCd-1** male x ***An. arabiensis*** **Dongola** female | Rep2 | F1 | ***Black****/****CFP+*** | Female | Drive/WT |
| DGL394 | OR695029 | **AgNosCd-1** male x ***An. arabiensis*** **Dongola** female | Rep2 | F1 | ***Black****/****CFP+*** | Female | Drive/WT |
| DGL395 | OR695030 | **AgNosCd-1** male x ***An. arabiensis*** **Dongola** female | Rep2 | F1 | ***Black****/****CFP+*** | Female | Drive/WT |
| DGL396 | OR695031 | **AgNosCd-1** male x ***An. arabiensis*** **Dongola** female | Rep2 | F1 | ***Black****/****CFP+*** | Female | Drive/WT |
| DGL397 | OR695032 | **AgNosCd-1** male x ***An. arabiensis*** **Dongola** female | Rep2 | F1 | ***Black****/****CFP+*** | Female | Drive/WT |
| DGL398 | OR695033 | **AgNosCd-1** male x ***An. arabiensis*** **Dongola** female | Rep2 | F1 | ***Black****/****CFP+*** | Female | Drive/WT |
| DGL399 | OR695034 | **AgNosCd-1** male x ***An. arabiensis*** **Dongola** female | Rep2 | F1 | ***Black****/****CFP+*** | Female | Drive/WT |
| DGL400 | OR695035 | **AgNosCd-1** male x ***An. arabiensis*** **Dongola** female | Rep2 | F1 | ***Black****/****CFP+*** | Female | Drive/WT |
| DGL401 | OR695036 | **AgNosCd-1** male x ***An. arabiensis*** **Dongola** female | Rep3 | F1 | ***Black****/****CFP+*** | Male | Drive/WT |
| DGL402 | OR695037 | **AgNosCd-1** male x ***An. arabiensis*** **Dongola** female | Rep3 | F1 | ***Black****/****CFP+*** | Male | Drive/WT |
| DGL403 | OR695038 | **AgNosCd-1** male x ***An. arabiensis*** **Dongola** female | Rep3 | F1 | ***Black****/****CFP+*** | Male | Drive/WT |
| DGL404 | OR695039 | **AgNosCd-1** male x ***An. arabiensis*** **Dongola** female | Rep3 | F1 | ***Black****/****CFP+*** | Male | Drive/WT |
| DGL405 | OR695040 | **AgNosCd-1** male x ***An. arabiensis*** **Dongola** female | Rep3 | F1 | ***Black****/****CFP+*** | Male | Drive/WT |
| DGL406 | OR695041 | **AgNosCd-1** male x ***An. arabiensis*** **Dongola** female | Rep3 | F1 | ***Black****/****CFP+*** | Male | Drive/WT |
| DGL407 | OR695042 | **AgNosCd-1** male x ***An. arabiensis*** **Dongola** female | Rep3 | F1 | ***Black****/****CFP+*** | Male | Drive/WT |
| DGL408 | OR695043 | **AgNosCd-1** male x ***An. arabiensis*** **Dongola** female | Rep3 | F1 | ***Black****/****CFP+*** | Male | Drive/WT |
| DGL409 | OR695044 | **AgNosCd-1** male x ***An. arabiensis*** **Dongola** female | Rep3 | F1 | ***Black****/****CFP+*** | Male | Drive/WT |
| DGL410 | OR695045 | **AgNosCd-1** male x ***An. arabiensis*** **Dongola** female | Rep3 | F1 | ***Black****/****CFP+*** | Male | Drive/WT |
| DGL411 | OR695046 | **AgNosCd-1** male x ***An. arabiensis*** **Dongola** female | Rep3 | F1 | ***Black****/****CFP+*** | Female | Drive/WT |
| DGL412 | OR695047 | **AgNosCd-1** male x ***An. arabiensis*** **Dongola** female | Rep3 | F1 | ***Black****/****CFP+*** | Female | Drive/WT |
| DGL413 | OR695048 | **AgNosCd-1** male x ***An. arabiensis*** **Dongola** female | Rep3 | F1 | ***Black****/****CFP+*** | Female | Drive/WT |
| DGL414 | OR695049 | **AgNosCd-1** male x ***An. arabiensis*** **Dongola** female | Rep3 | F1 | ***Black****/****CFP+*** | Female | Drive/WT |
| DGL415 | OR695050 | **AgNosCd-1** male x ***An. arabiensis*** **Dongola** female | Rep3 | F1 | ***Black****/****CFP+*** | Female | Drive/WT |
| DGL416 | OR695051 | **AgNosCd-1** male x ***An. arabiensis*** **Dongola** female | Rep3 | F1 | ***Black****/****CFP+*** | Female | Drive/WT |
| DGL417 | OR695052 | **AgNosCd-1** male x ***An. arabiensis*** **Dongola** female | Rep3 | F1 | ***Black****/****CFP+*** | Female | Drive/WT |
| DGL418 | OR695053 | **AgNosCd-1** male x ***An. arabiensis*** **Dongola** female | Rep3 | F1 | ***Black****/****CFP+*** | Female | Drive/WT |
| DGL419 | OR695054 | **AgNosCd-1** male x ***An. arabiensis*** **Dongola** female | Rep3 | F1 | ***Black****/****CFP+*** | Female | Drive/WT |
| DGL420 | OR695055 | **AgNosCd-1** male x ***An. arabiensis*** **Dongola** female | Rep3 | F1 | ***Black****/****CFP+*** | Female | Drive/WT |
